# Supplementary material for: Passerini-type reaction of boronic acids enables α-hydroxyketones synthesis
Source: Nat Commun. 2021 Jan 19;12:441. doi: 10.1038/s41467-020-20727-7 (PMC7815879; doi:10.1038/s41467-020-20727-7)
Supplement: Supplementary file 1 — Supplementary Information [file 41467_2020_20727_MOESM1_ESM.pdf]

Supplementary Information

Passerini-Type Reaction of Boronic Acids Enables *α*-Hydroxyketones Synthesis

Kai Yang, Feng Zhang, Tongchang Fang, Chaokun Li, Wangyang Li, and Qiuling Song*

Key Laboratory of Molecule Synthesis and Function Discovery, Fujian Province University, College of Chemistry at Fuzhou University, Fuzhou, Fujian, 350108 (China)

E-mail: qsong@hqu.edu.cn

Table of contents

1 General information 2

2 General procedure A for the synthesis of α-hydroxyketones from alkylaldehydes, isocyanides and arylboronic acids or alkenylboronic acids 2

3 General procedure B for the synthesis of *α*-hydroxyketones from arylaldehydes, isocyanides and arylboronic acids 2

4 General procedure C for the synthesis of α-hydroxyketones from aldehydes, isocyanides and alkynyl trifluoroborate salt 2

4 Gram-scale synthesis of 5e and 5f 29

5 Transformations of α-hydroxyketone 29

6 Synthetic applications 32

7 NMR spectra 36

8 Supplementary references 119

1 General information

Unless otherwise noted, materials obtained from commercial suppliers were used without further purification, and most starting materials were purchased from Energy Chemical, Bidepharm and Adamas. Flash column chromatography was performed over silica gel (200-300 mesh). ^{1}H NMR and ^{13}C NMR spectra were recorded at ambient temperature using Bruker 400M and JEOL 500M spectrometers, chemical shifts (in ppm) were referenced to $CDCl_3$ (δ = 7.26 ppm) as internal standards. ^{13}C NMR spectra were obtained by using the same NMR spectrometers and were calibrated with $CDCl_3$ (δ = 77.0 ppm). Data for ^{1}H NMR are recorded as following abbreviations: multiplicity (s = singlet, d = doublet, t = triplet, q = quarter, m = multiplet), coupling constant (*J*, Hz). High resolution mass spectroscopy (HRMS) analysis was performed at an Exactive Plus (Thermo Scientific). IR analysis was performed at an NICOLET iS50 FT-IR (Thermo Scientific).

2 General procedure A for the synthesis of α-hydroxyketones from alkylaldehydes, isocyanides and arylboronic acids or alkenylboronic acids

In air, a 10 mL schlenk tube was charged with arylboronic acids (0.36 mmol, 1.8 equiv). The tube was evacuated and filled with argon for three cycles. Then, chloroform (0.7 mL), pH = 8 buffer (0.3 mL), alkylaldehydes (0.20 mmol, 1 equiv), tertbutyl isocyanide (34 μl, 0.30 mmol, 1.5 equiv) were added under argon. The reaction was allowed to stir at corresponding temperature for 24 hours. Upon completion, proper amount of silica gel was added to the reaction mixture. After removal of the solvent, the crude reaction mixture was purified on silica gel (petroleum ether and ethyl acetate) to afford the desired products.

3 General procedure B for the synthesis of *α*-hydroxyketones from arylaldehydes, isocyanides and arylboronic acids

In air, a 10 mL schlenk tube was charged with arylboronic acids (0.36 mmol, 1.8 equiv). The tube was evacuated and filled with argon for three cycles. Then, dichloromethane (0.7 mL), pH = 8 buffer (0.3 mL), arylaldehydes (0.20 mmol, 1 equiv), cyclohexyl isocyanide (37 μl, 0.30 mmol, 1.5 equiv) were added under argon. The reaction was allowed to stir at room temperature for 24 hours. Upon completion, proper amount of silica gel was added to the reaction mixture. After removal of the solvent, the crude reaction mixture was purified on silica gel (petroleum ether and ethyl acetate) to afford the desired products.

4 General procedure C for the synthesis of α-hydroxyketones from aldehydes, isocyanides and alkynyl trifluoroborate salt

In air, a 10 mL schlenk tube was charged with alkynyl trifluoroborate salt (0.60 mmol, 3 equiv) and $Sc(OTf)_3$ (30.0 mg, 0.06 mmol, 0.3 equiv). The tube was evacuated and filled with argon for three cycles. Then, THF (1.5

mL), aldehydes (0.20 mmol, 1 equiv), and tertbutyl isocyanide (57 μl, 0.50 mmol, 2.5 equiv) were added under argon. The reaction was allowed to stir at room temperature for 12 hours. Upon completion, proper amount of silica gel was added to the reaction mixture. After removal of the solvent, the crude reaction mixture was purified on silica gel (petroleum ether and ethyl acetate) to afford the desired products.

2-hydroxy-1-(4-methoxyphenyl)-4-phenylbutan-1-one (4a)

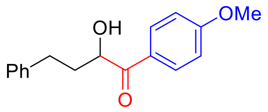

4a

The general procedure A was followed by using 3-phenylpropanal (27.0 mg, 0.20 mmol, 1 equiv), tertbutyl isocyanide (34 μl, 0.30 mmol, 1.5 equiv) and (4-methoxyphenyl)boronic acid (55.0 mg, 0.36 mmol, 1.8 equiv) to afford 43.7 mg (81%) of the product **4a** as a colorless oil. TLC (petroleum ether: ethyl acetate, 84:16 v/v): R_f = 0.30, the ratio of eluents (petroleum ether: ethyl acetate, v/v): 90:10. **^{1}H NMR (400 MHz, CDCl$_3$)** δ 7.77 (d, *J* = 7.3 Hz, 2H), 7.32-7.26 (m, 2H), 7.23-7.16 (m, 3H), 6.91 (d, *J* = 7.3 Hz, 2H), 4.98 (t, *J* = 7.4 Hz, 1H), 3.87 (s, 3H), 3.81 (d, *J* = 5.7 Hz, 1H), 2.89-2.76 (m, 2H), 2.19-2.11 (m, 1H), 1.86-1.79 (m, 1H). **^{13}C NMR (151 MHz, CDCl$_3$)** δ 200.2, 164.1, 141.2, 130.9, 128.6, 128.4, 126.1, 126.1, 114.0, 71.7, 55.5, 38.0, 31.3. **IR (neat):** 3471, 1670 cm^{-1}. **HRMS (ESI)** calcd for $C_{17}H_{18}O_3$ [M+H]$^+$: 271.1329, found: 271.1327.

2-hydroxy-1-(4-methoxyphenyl)-4-(4-nitrophenyl)butan-1-one (4b)

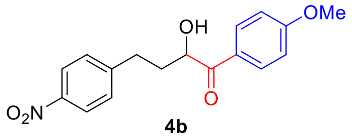

4b

The general procedure A was followed by using 3-(4-nitrophenyl)propanal (35.8 mg, 0.20 mmol, 1 equiv), tertbutyl isocyanide (34 μl, 0.3 mmol, 1.5 equiv) and (4-methoxyphenyl)boronic acid (55.0 mg, 0.36 mmol, 1.8 equiv) to afford 41.6 mg (66%) of the product **4b** as a yellow oil. TLC (petroleum ether: ethyl acetate, 84:16 v/v): R_f = 0.10, the ratio of eluents (petroleum ether: ethyl acetate, v/v): 80:20. **^{1}H NMR (400 MHz, CDCl$_3$)** δ 8.12 (d, *J* = 7.5 Hz, 2H), 7.79 (d, *J* = 7.5 Hz, 2H), 7.32 (d, *J* = 7.6 Hz, 2H), 6.94 (d, *J* = 7.5 Hz, 2H), 4.99 (s, 1H), 3.88 (s, 3H), 3.01-2.91 (m, 1H), 2.89-2.78 (m, 1H), 2.25-2.12 (m, 1H), 1.93-1.80 (m, 1H). **^{13}C NMR (101 MHz, CDCl$_3$)** δ 199.6, 164.3, 149.1, 146.5, 130.8, 129.4, 126.0, 123.7, 114.2, 71.4, 55.6, 37.2, 31.0. **IR (neat):** 3463, 1670 cm^{-1}. **HRMS (ESI)** calcd for $C_{17}H_{17}NO_5$ [M+H]$^+$: 316.1179, found:316.1173.

2-hydroxy-1-(4-methoxyphenyl)-4-(4-(trifluoromethyl)phenyl)butan-1-one (4c)

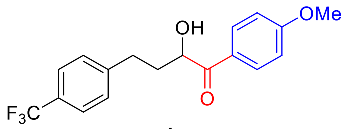

4c

The general procedure A was followed by using 3-(4-(trifluoromethyl)phenyl)propanal (40.5 mg, 0.20 mmol, 1 equiv), tertbutyl isocyanide (34 μl, 0.3 mmol, 1.5 equiv) and (4-methoxyphenyl)boronic acid (55.0 mg, 0.36 mmol, 1.8 equiv) to afford 57.5 mg (85%) of the product **4c** as a colorless oil. TLC (petroleum ether: ethyl acetate, 84:16 v/v): R_f = 0.35, the

ratio of eluents (petroleum ether: ethyl acetate, v/v): 90:10. **^{1}H NMR (400 MHz, CDCl$_3$)** δ 7.77 (d, *J* = 7.9 Hz, 2H), 7.51 (d, *J* = 7.6 Hz, 2H), 7.27 (d, *J* = 8.7 Hz, 2H), 6.92 (d, *J* = 7.9 Hz, 2H), 4.98 (s, 1H), 3.86 (d, *J* = 8.2 Hz, 4H), 2.95-2.75 (m, 2H), 2.23-2.09 (m, 1H), 1.91-1.77 (m, 1H). **^{13}C NMR (101 MHz, CDCl$_3$)** δ 199.8, 164.3, 145.4, 130.8, 128.9, 126.1, 125.32 (q, *J* = 3.5 Hz), 114.1, 71.6, 55.6, 37.6, 31.0. **IR (neat):** 3391, 1673 cm^{-1}. **HRMS (ESI)** calcd for $C_{18}H_{17}F_3O_3$ [M+H]$^+$:339.1203, found:339.1204.

2-hydroxy-1-(4-methoxyphenyl)-4-(5-methylfuran-2-yl)butan-1-one (4d)

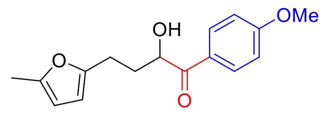

4d

The general procedure A was followed by using 3-(5-methylfuran-2-yl)propanal (27.6 mg, 0.20 mmol, 1 equiv), tertbutyl isocyanide (34 μl, 0.3 mmol, 1.5 equiv) and (4-methoxyphenyl)boronic acid (55.0 mg, 0.36 mmol, 1.8 equiv) to afford 49.3 mg (90%) of the product **4d** as a light yellow oil. TLC (petroleum ether: ethyl acetate, 84:16 v/v): R_f = 0.25, the ratio of eluents (petroleum ether: ethyl acetate, v/v): 90:10. **^{1}H NMR (400 MHz, CDCl$_3$)** δ 7.82 (d, *J* = 7.8 Hz, 2H), 6.93 (d, *J* = 7.8 Hz, 2H), 5.88 (d, *J* = 24.2 Hz, 2H), 4.99 (s, 1H), 3.87 (s, 3H), 3.80 (d, *J* = 5.9 Hz, 1H), 2.91-2.68 (m, 2H), 2.23 (s, 3H), 2.22-2.15 (m, 1H), 1.86-1.72 (m, 1H). **^{13}C NMR (101 MHz, CDCl$_3$)** δ 200.0, 164.1, 152.8, 150.4, 130.9, 126.1, 114.0, 106.5, 105.9, 71.7, 55.5, 34.8, 23.8, 13.4. **IR (neat):** 3466, 1670 cm^{-1}. **HRMS (ESI)** calcd for $C_{16}H_{18}O_4$ [M+H]$^+$: 275.1278, found: 275.1279.

2-hydroxy-1-(4-methoxyphenyl)hexan-1-one(4e)

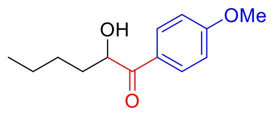

4e

The general procedure A was followed by using pentanal (17.2 mg, 0.20 mmol, 1 equiv), tertbutyl isocyanide (34 μl, 0.3 mmol, 1.5 equiv) and (4-methoxyphenyl)boronic acid (55.0 mg, 0.36 mmol, 1.8 equiv) to afford 25.3 mg (57%) of the product **4e** as a colorless oil. TLC (petroleum ether: ethyl acetate, 84:16 v/v): R_f = 0.35, the ratio of eluents (petroleum ether: ethyl acetate, v/v): 90:10. **^{1}H NMR (400 MHz, CDCl$_3$)** δ 7.90 (d, *J* = 7.9 Hz, 2H), 6.97 (d, *J* = 8.0 Hz, 2H), 5.01 (s, 1H), 3.88 (s, 3H), 3.75 (d, *J* = 5.8 Hz, 1H), 1.93-1.77 (m, 1H), 1.58-1.43 (m, 2H), 1.37-1.23 (m, 3H), 0.86 (t, *J* = 6.5 Hz, 3H). **^{13}C NMR (151 MHz, CDCl$_3$)** δ 200.4, 164.1, 130.8, 126.4, 114.1, 72.7, 55.5, 36.0, 27.1, 22.5, 13.9. **IR (neat):** 3483, 1670 cm^{-1}. **HRMS (ESI)** calcd for $C_{13}H_{18}O_3$[M+H]$^+$: 223.1329, found:223.1332.

2-hydroxy-1-(4-methoxyphenyl)heptan-1-one (4f)

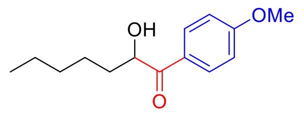

4f

The general procedure A was followed by using hexanal (20.0 mg, 0.20 mmol, 1 equiv), tertbutyl isocyanide (34 μl, 0.3 mmol, 1.5 equiv) and (4-methoxyphenyl)boronic acid (55.0 mg, 0.36 mmol, 1.8 equiv) to afford 32.0 mg (68%) of the product **4f** as a colorless oil. TLC (petroleum ether: ethyl

acetate, 84:16 v/v): R_f = 0.30, the ratio of eluents (petroleum ether: ethyl acetate, v/v): 90:10. **^{1}H NMR (400 MHz, $CDCl_3$)** δ 7.90 (d, *J* = 7.2 Hz, 2H), 6.97 (d, *J* = 7.2 Hz, 2H), 5.01 (s, 1H), 3.88 (s, 3H), 3.74 (d, *J* = 5.8 Hz, 1H), 1.83 (dd, *J* = 18.3, 10.6 Hz, 1H), 1.59-1.45 (m, 2H), 1.38-1.20 (m, 5H), 0.86 (t, *J* = 5.6 Hz, 3H). **^{13}C NMR (151 MHz, $CDCl_3$)** δ 200.4, 164.1, 130.9, 126.4, 114.1, 72.7, 55.5, 36.2, 31.6, 24.6, 22.5, 14.0. **IR (neat):** 3415, 1671 cm^{-1}. **HRMS (ESI)** calcd for $C_{14}H_{20}O_3$ $[M+H]^+$: 237.1485, found: 237.1489.

2-hydroxy-1-(4-methoxyphenyl)undecan-1-one (4g)

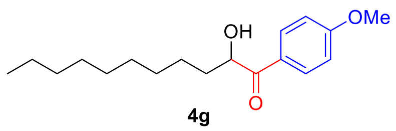

4g

The general procedure A was followed by using decanal (31.2 mg, 0.20 mmol, 1 equiv), tertbutyl isocyanide (34 µl, 0.3 mmol, 1.5 equiv) and (4-methoxyphenyl)boronic acid (55.0 mg, 0.36 mmol, 1.8 equiv) to afford 34.5 mg (59%) of the product **4g** as a colorless oil. TLC (petroleum ether: ethyl acetate, 84:16 v/v): R_f = 0.50, the ratio of eluents (petroleum ether: ethyl acetate, v/v): 95:5. **^{1}H NMR (400 MHz, $CDCl_3$)** δ 7.90 (d, *J* = 7.5 Hz, 2H), 6.97 (d, *J* = 7.5 Hz, 2H), 5.01 (s, 1H), 3.88 (s, 3H), 3.75 (d, *J* = 6.1 Hz, 1H), 1.90-1.75 (m, 1H), 1.57-1.45 (m, 2H), 1.34-1.20 (m, 13H), 0.86 (t, *J* = 6.3 Hz, 3H). **^{13}C NMR (101 MHz, $CDCl_3$)** δ 200.4, 164.1, 130.9, 126.4, 114.0, 72.7, 55.5, 36.3, 31.8, 29.5, 29.4, 29.4, 29.2, 24.9, 22.6, 14.1. **IR (neat):** 3351, 1683 cm^{-1}. **HRMS (ESI)** calcd for $C_{18}H_{28}O_3[M+H]^+$: 293.2111, found:293.2112.

ethyl 5-hydroxy-6-(4-methoxyphenyl)-6-oxohexanoate (4h)

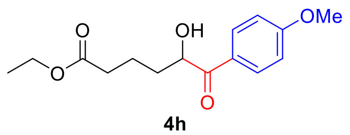

4h

The general procedure A was followed by using ethyl 5-oxopentanoate (29.0 mg, 0.20 mmol, 1 equiv), tertbutyl isocyanide (34 µl, 0.3 mmol, 1.5 equiv) and (4-methoxyphenyl)boronic acid (55.0 mg, 0.36 mmol, 1.8 equiv) to afford 38.1 mg (68%) of the product **4h** as a colorless oil. TLC (petroleum ether: ethyl acetate, 84:16 v/v): R_f = 0.15, the ratio of eluents (petroleum ether: ethyl acetate, v/v): 85:15. **^{1}H NMR (400 MHz, $CDCl_3$)** δ 7.89 (d, *J* = 8.0 Hz, 2H), 6.95 (d, *J* = 8.1 Hz, 2H), 5.01 (s, 1H), 4.07 (dd, *J* = 13.8, 6.7 Hz, 2H), 3.87 (s, 3H), 3.77 (d, *J* = 6.2 Hz, 1H), 2.32 (t, *J* = 6.6 Hz, 2H), 1.99-1.65 (m, 3H), 1.62-1.45 (m, 1H), 1.19 (t, *J* = 7.0 Hz, 3H). **^{13}C NMR (101 MHz, $CDCl_3$)** δ 199.9, 173.2, 164.2, 130.9, 126.2, 114.1, 72.3, 60.3, 55.5, 35.4, 33.8, 20.7, 14.2. **IR (neat):** 3463, 1727, 1670 cm^{-1}. **HRMS (ESI)** calcd for $C_{15}H_{21}O_5$ $[M+H]^+$: 281.1384, found: 281.1389.

3-(adamantan-1-yl)-2-hydroxy-1-(4-methoxyphenyl)propan-1-one (4i)

The general procedure A was followed by using 2-(adamantan-1-yl)acetaldehyde (35.6 mg, 0.20 mmol, 1 equiv), tertbutyl isocyanide (34 µl, 0.3 mmol, 1.5 equiv) and (4-methoxyphenyl)boronic acid (55.0 mg, 0.36 mmol, 1.8 equiv) to afford 26.4 mg (42%) of the product **4i** as a light yellow oil. TLC (petroleum ether: ethyl acetate, 84:16

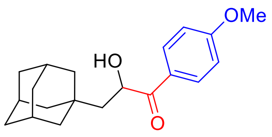

4i

v/v): R_f = 0.50, the ratio of eluents (petroleum ether: ethyl acetate, v/v): 95:5. **^{1}H NMR (400 MHz, $CDCl_3$)** δ 7.89 (d, *J* = 7.4 Hz, 2H), 6.97 (d, *J* = 7.4 Hz, 2H), 5.22-5.10 (m, 1H), 3.88 (s, 3H), 3.72 (d, *J* = 6.8 Hz, 1H), 2.01 (s, 3H), 1.75-1.64 (m, 12H), 1.29-1.12 (m, 2H). **^{13}C NMR (101 MHz, $CDCl_3$)** δ 201.2, 164.0, 131.0, 126.1, 114.0, 69.6, 55.5, 50.6, 42.9, 37.0, 33.3, 28.7. **IR (neat):** 3440, 1663 cm^{-1}. **HRMS (ESI)** calcd for $C_{20}H_{26}O_3$ $[M+H]^+$: 315.1955, found: 315.1956.

3-(benzyloxy)-2-hydroxy-1-(4-methoxyphenyl)propan-1-one (4j)

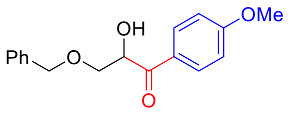

4j

The general procedure A was followed by using 2-(benzyloxy)acetaldehyde (30.0 mg, 0.20 mmol, 1 equiv), tertbutyl isocyanide (34 μl, 0.20 mmol, 1.5 equiv) and (4-methoxyphenyl)boronic acid (55.0 mg, 0.36 mmol, 1.8 equiv) to afford 37.8 mg (66%) of the product **4j** as a colorless oil. TLC (petroleum ether: ethyl acetate, 84:16 v/v): R_f = 0.30, the ratio of eluents (petroleum ether: ethyl acetate, v/v): 90:10. **^{1}H NMR (400 MHz, $CDCl_3$)** δ 7.93 (d, *J* = 8.1 Hz, 2H), 7.33-7.23 (m, 3H), 7.22-7.13 (s, 2H), 6.96 (d, *J* = 8.1 Hz, 2H), 5.19 (s, 1H), 4.52 (q, *J* = 12.2 Hz, 2H), 4.08 (s, 1H), 3.90 (s, 3H), 3.86-3.72 (m, 2H). **^{13}C NMR (101 MHz, $CDCl_3$)** δ 197.6, 164.1, 137.6, 130.9, 128.2, 127.5, 127.5, 126.6, 114.0, 73.2, 72.8, 55.5. **IR (neat):** 3461, 1665 cm^{-1}. **HRMS (ESI)** calcd for $C_{17}H_{18}O_4$ $[M+H]^+$: 287.1278, found: 287.1274.

2-hydroxy-1-(4-methoxyphenyl)-3-methylbutan-1-one (4k)

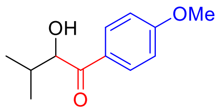

4k

The general procedure A was followed by using isobutyraldehyde (14.5 mg, 0.20 mmol, 1 equiv), tertbutyl isocyanide (34 μl, 0.3 mmol, 1.5 equiv) and (4-methoxyphenyl)boronic acid (55.0 mg, 0.36 mmol, 1.8 equiv) to afford 25.4 mg (61%) of the product **4k** as a colorless oil. TLC (petroleum ether: ethyl acetate, 84:16 v/v): R_f = 0.40, the ratio of eluents (petroleum ether: ethyl acetate, v/v): 93:7. **^{1}H NMR (400 MHz, $CDCl_3$)** δ 7.89 (d, *J* = 7.6 Hz, 2H), 6.96 (d, *J* = 7.6 Hz, 2H), 4.99-4.80 (m, 1H), 3.88 (s, 3H), 3.65 (d, *J* = 6.3 Hz, 1H), 2.18-2.02 (m, 1H), 1.16 (d, *J* = 6.6 Hz, 3H), 0.65 (d, *J* = 6.4 Hz, 3H). **^{13}C NMR (151 MHz, $CDCl_3$)** δ 200.4, 164.1, 130.8, 126.8, 114.0, 55.5, 33.0, 20.2, 14.3. **IR (neat):** 3421, 1668 cm^{-1}. **HRMS (ESI)** calcd for $C_{17}H_{18}O_3$ $[M+H]^+$: 209.1172, found: 209.1178.

2-hydroxy-1-(4-methoxyphenyl)-3,7-dimethyloct-6-en-1-one (4l), dr = 1.13:1

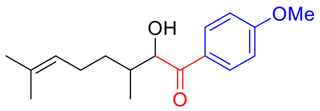

4l

The general procedure A was followed by using 2,6-dimethylhept-5-enal (28.0 mg, 0.20 mmol, 1 equiv), tertbutyl isocyanide (34 μl, 0.3 mmol, 1.5 equiv) and (4-methoxyphenyl)boronic acid (55.0 mg, 0.36 mmol, 1.8 equiv) to afford 24.3 mg (44%) of the product **4l** as a colorless oil. TLC (petroleum ether:

ethyl acetate, 84:16 v/v): R_f = 0.40, 0.48, the ratio of eluents (petroleum ether: ethyl acetate, v/v): 95:5. **^{1}H NMR (400 MHz, $CDCl_3$)** δ 7.88 (d, *J* = 7.4 Hz, 2H), 7.01-6.91 (m, 2H), 5.17 (t, *J* = 7.5 Hz, 0.53*1H), 5.02 (d, *J* = 6.1 Hz, 0.53*1H), 4.94-4.89 (m, 0.47*1H), 4.79 (t, *J* = 7.0 Hz, 0.47*1H), 3.89 (s, 3H), 3.67 (t, *J* = 4.7 Hz, 1H), 2.28-2.09 (m, 1H), 2.05-1.80 (m, 2H), 1.74 (s, 0.47*3H), 1.67 (s, 0.53*3H), 1.52 (s, 0.47*3H), 1.46 (s, 0.53*3H), 1.39-1.27 (m, 1H), 1.16 (d, *J* = 6.6 Hz, 0.47*3H), 1.12-0.96 (m, 1H), 0.62 (d, *J* = 6.7 Hz, 0.53*3H). **^{13}C NMR (151 MHz, $CDCl_3$)** δ 200.6, 164.0, 131.8, 131.6, 130.8, 127.0, 126.6, 124.5, 124.1, 113.9, 77.5, 74.6, 55.5, 37.6, 36.9, 34.0, 29.1, 25.8, 25.6, 25.5, 25.3, 17.8, 17.5, 17.3, 12.5. **IR (neat):** 3469, 1667 cm^{-1}. **HRMS (ESI)** calcd for $C_{17}H_{24}O_3$ $[M+H]^+$: 277.1798, found: 277.1805.

2-cyclopropyl-2-hydroxy-1-(p-tolyl)ethan-1-one (4m)

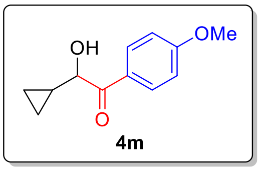

The general procedure A was followed by using cyclopropanecarbaldehyde (14.0 mg, 0.20 mmol, 1 equiv), tertbutyl isocyanide (34 μl, 0.3 mmol, 1.5 equiv) and (4-methoxyphenyl)boronic acid (55.0 mg, 0.36 mmol, 1.8 equiv) to afford 18.5 mg (45%) of the product **4m** as a colorless oil. TLC (petroleum ether: ethyl acetate, 84:16 v/v): R_f = 0.30, the ratio of eluents (petroleum ether: ethyl acetate, v/v): 90:10. **^{1}H NMR (400 MHz, $CDCl_3$)** δ 7.97 (d, *J* = 8.3 Hz, 2H), 6.99 (d, *J* = 8.4 Hz, 2H), 4.91 (t, *J* = 5.6 Hz, 1H), 3.89 (s, 3H), 3.59 (d, *J* = 6.8 Hz, 1H), 1.14-0.99 (m, 1H), 0.70-0.57 (m, 1H), 0.54-0.32 (m, 3H). **^{13}C NMR (151 MHz, $CDCl_3$)** δ 199.6, 164.2, 131.2, 126.7, 114.0, 71.9, 55.5, 15.5, 2.5. **IR (neat):** 3468, 1670 cm^{-1}. **HRMS (ESI)** calcd for $C_{12}H_{14}O_2[M+H]^+$: 191.1067, found:191.1069.

2-hydroxy-1-(4-methoxyphenyl)-2-(2-phenylcyclopropyl)ethan-1-one (4n) dr = 1.38:1

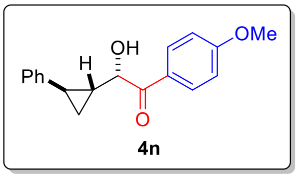

The general procedure A was followed by using 2-phenylcyclopropane-1-carbaldehyde (29.2 mg, 0.20 mmol, 1 equiv), tertbutyl isocyanide (34 μl, 0.3 mmol, 1.5 equiv) and (4-methoxyphenyl)boronic acid (55.0 mg, 0.36 mmol, 1.8 equiv) to afford 32.1 mg (57%) of the product **4n** as a light yellow oil. TLC (petroleum ether: ethyl acetate, 84:16 v/v): R_f = 0.30, 0.35, the ratio of eluents (petroleum ether: ethyl acetate, v/v): 90:10. ^{1}H NMR (400 MHz, $CDCl_3$) δ 8.05-7.94 (m, 2H), 7.26-7.20 (m, 1H), 7.19-7.04 (m, 2H), 7.03-6.90 (m, 4H), 5.21 (s, 0.58*1H), 5.05 (t, *J* = 5.5 Hz, 0.42*1H), 3.89 (s, 3H), 3.78-3.71 (m, 1H), 2.29-2.22 (m, 0.58*1H), 2.10-2.02 (m, 0.42*1H), 1.41-1.33 (m, 1.48*1H), 1.12-1.06 (m, 0.58*1H), 0.99-0.92 (m, 0.42*1H), 0.83-0.77 (m, 0.58*1H). **^{13}C NMR (101 MHz, $CDCl_3$)** δ 199.0, 198.9, 164.3. 164.2, 142.3, 141.8, 131.2, 131.1, 128.3, 128.2, 126.6, 126.4, 126.3, 125.9, 125.7, 125.6, 114.2, 114.1, 71.6, 70.5, 55.5, 27.0, 26.5, 19.9, 19.0, 12.4, 10.1. **IR (neat):** 3449, 1668 cm^{-1}. **HRMS (ESI)** calcd for $C_{18}H_{18}O_3[M+H]^+$: 283.1329, found:283.1324.

2-cyclohexyl-2-hydroxy-1-(4-methoxyphenyl)ethan-1-one (4o)

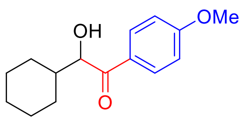

4o

The general procedure A was followed by using cyclohexanecarbaldehyde (22.5 mg, 0.20 mmol, 1 equiv), tertbutyl isocyanide (34 μl, 0.3 mmol, 1.5 equiv) and (4-methoxyphenyl)boronic acid (55.0 mg, 0.36 mmol, 1.8 equiv) to afford 34.2 mg (69%) of the product **4o** as a colorless oil. TLC (petroleum ether: ethyl acetate, 84:16 v/v): R_f = 0.40, the ratio of eluents (petroleum ether: ethyl acetate, v/v): 90:10. **^{1}H NMR (400 MHz, $CDCl_3$)** δ 7.89 (d, *J* = 7.7 Hz, 2H), 6.96 (d, *J* = 7.7 Hz, 2H), 4.87 (d, *J* = 6.4 Hz, 1H), 3.88 (s, 3H), 3.65 (d, *J* = 6.5 Hz, 1H), 1.85-1.71 (m, 3H), 1.67-1.49 (m, 3H), 1.27-1.19 (m, 1H), 1.16-0.96 (m, 4H). **^{13}C NMR (101 MHz, $CDCl_3$)** δ 200.3, 164.1, 130.8, 126.9, 114.0, 76.8, 55.5, 42.9, 30.3, 26.5, 25.9, 25.8, 24.8. **IR (neat):** 3476, 1667 cm^{-1}. **HRMS (ESI)** calcd for $C_{15}H_{20}O_3[M+H]^+$: 249.1485, found:249.1484.

methyl 4-(1-hydroxy-2-(4-methoxyphenyl)-2-oxoethyl)cyclohexane-1-carboxylate (4p)

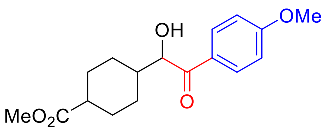

4p

The general procedure A was followed by using methyl 4-formylcyclohexane-1-carboxylate (34.0 mg, 0.20 mmol, 1 equiv), tertbutyl isocyanide (34 μl, 0.3 mmol, 1.5 equiv) and (4-methoxyphenyl)boronic acid (55.0 mg, 0.36 mmol, 1.8 equiv) to afford 46.5 mg (76%) of the product **4p** as a colorless oil. TLC (petroleum ether: ethyl acetate, 84:16 v/v): R_f = 0.20, the ratio of eluents (petroleum ether: ethyl acetate, v/v): 80:20. **^{1}H NMR (400 MHz, $CDCl_3$)** δ 7.88 (d, *J* = 8.0 Hz, 2H), 6.96 (d, *J* = 7.9 Hz, 2H), 4.89 (d, *J* = 6.0 Hz, 1H), 3.87 (s, 3H), 3.69 (d, *J* = 6.1 Hz, 1H), 3.61 (s, 3H), 2.19 (t, *J* = 9.9 Hz, 1H), 2.03 (d, *J* = 11.5 Hz, 1H), 1.89 (d, *J* = 9.0 Hz, 2H), 1.76 (d, *J* = 18.6 Hz, 1H), 1.61 (dd, *J* = 25.4, 12.7 Hz, 1H), 1.41 (dd, *J* = 25.4, 12.9 Hz, 1H), 1.27-1.16 (m, 3H).**^{13}C NMR (101 MHz, $CDCl_3$)** δ 199.9, 176.1, 164.17, 130.8, 126.6, 114.1, 76.1, 55.5, 51.4, 42.5, 41.9, 29.0, 28.8, 28.2, 23.6. **IR (neat):** 3473, 1727, 1667 cm^{-1}. **HRMS (ESI)** calcd for $C_{17}H_{22}O_5$ $[M+H]^+$: 307.1540, found: 307.1541.

tert-butyl 4-(1-hydroxy-2-(4-methoxyphenyl)-2-oxoethyl)piperidine-1-carboxylate (4q)

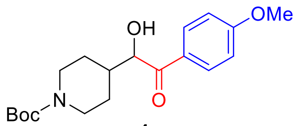

4q

The general procedure A was followed by using tert-butyl 4-formylpiperidine-1-carboxylate (42.6 mg, 0.20 mmol, 1 equiv), tertbutyl isocyanide (34 μl, 0.3 mmol, 1.5 equiv) and (4-methoxyphenyl)boronic acid (55.0 mg, 0.36 mmol, 1.8 equiv) to afford 55.8 mg (80%) of the product **4q** as a colorless oil. TLC (petroleum ether: ethyl acetate, 80:20 v/v): R_f = 0.10, the ratio of eluents (petroleum ether: ethyl acetate, v/v): 70:30. **^{1}H NMR (400 MHz, $CDCl_3$)** δ 7.88 (d, *J* = 7.8 Hz, 2H), 6.97 (d, *J* = 7.7 Hz, 2H), 4.91 (s, 1H), 4.28-3.94 (m, 2H), 3.88 (s, 3H), 3.67 (s, 1H), 2.72-2.55 (m, 1H), 2.52-2.35 (m, 1H), 1.90-1.80 (m, 1H), 1.72-1.65 (m, 2H), 1.41 (s, 9H), 1.36-1.23 (m, 2H), 1.14-1.05 (m, 1H). **^{13}C NMR (101 MHz, $CDCl_3$)** δ 199.5,

164.3, 154.6, 130.8, 126.7, 114.2, 79.3, 75.5, 55.5, 41.3, 29.0, 28.4, 24.3. **IR (neat):** 3465, 1672 cm^{-1}. **HRMS (ESI)** calcd for $C_{19}H_{2}NO_{5}$ $[M+H]^+$: 350.1962, found: 350.1966.

2-hydroxy-1-(4-methoxyphenyl)-2-(1-phenylcyclobutyl)ethan-1-one (4r)

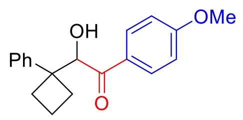

4r

The general procedure A was followed by using 1-phenylcyclobutane-1-carbaldehyde (32.0 mg, 0.20 mmol, 1 equiv), tertbutyl isocyanide (34 µl, 0.3 mmol, 1.5 equiv) and (4-methoxyphenyl)boronic acid (55.0 mg, 0.36 mmol, 1.8 equiv) to afford 32.0 mg (54%) of the product **4r** as a colorless oil. TLC (petroleum ether: ethyl acetate, 84:16 v/v): R_f = 0.35, the ratio of eluents (petroleum ether: ethyl acetate, v/v): 90:10. **^{1}H NMR (400 MHz, CDCl$_3$)** δ 7.57 (d, *J* = 7.4 Hz, 2H), 7.21-7.05 (m, 3H), 6.99 (d, *J* = 7.2 Hz, 2H), 6.80 (d, *J* = 7.4 Hz, 2H), 5.22 (d, *J* = 8.1 Hz, 1H), 3.84 (s, 3H), 3.67 (d, *J* = 8.3 Hz, 1H), 2.73-2.51 (m, 2H), 2.41-2.15 (m, 2H), 2.11-1.92 (m, 1H), 1.87-1.70 (m, 1H). **^{13}C NMR (151 MHz, CDCl$_3$)** δ 200.0, 163.6, 144.7, 130.8, 129.5, 127.6, 127.1, 126.1, 113.5, 77.5, 55.4, 51.4, 31.4, 29.9, 16.3. **IR (neat):** 3486, 1664 cm^{-1}. **HRMS (ESI)** calcd for $C_{19}H_{20}O_{3}$ $[M+H]^+$: 297.1485, found: 297.1488.

2-hydroxy-1-(4-methoxyphenyl)ethan-1-one (4s)[1]

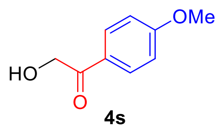

4s

The general procedure A was followed by using paraformaldehyde (150.0 mg, 5.0 mmol, 1 equiv), tertbutyl isocyanide (775 µl, 7.5 mmol, 1.5 equiv) and (4-methoxyphenyl)boronic acid (1.36 g, 9.0 mmol, 1.8 equiv) to afford 315 mg (38%) of the product **4s** as a white solid. mp: 102-105 °C (lit.[1] 103-105 °C). TLC (petroleum ether: ethyl acetate, 84:16 v/v): R_f = 0.20, the ratio of eluents (petroleum ether: ethyl acetate): 85:15. **^{1}H NMR (400 MHz, CDCl$_3$)** δ 7.90 (d, *J* = 7.3 Hz, 2H), 6.97 (d, *J* = 7.3 Hz, 2H), 4.82 (s, 2H), 3.88 (s, 3H), 3.57 (s, 1H).**^{13}C NMR (101 MHz, CDCl$_3$)** δ 196.7, 164.4, 123.0, 126.4, 114.16, 65.0, 55.5. **IR (neat):** 3382, 1671 cm^{-1}.

2-hydroxy-1-(4-methoxyphenyl)-2-phenylethan-1-one (4t)

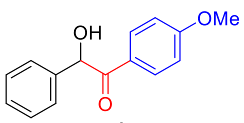

4t

The general procedure B was followed by using benzaldehyde (21.0 mg, 0.20 mmol, 1 equiv), cyclohexyl isocyanide (37 µl, 0.3 mmol, 1.5 equiv) and (4-methoxyphenyl)boronic acid (55.0 mg, 0.36 mmol, 1.8 equiv) to afford 34.0 mg (70%) of the product **4t** as a colorless oil. TLC (petroleum ether: ethyl acetate, 84:16 v/v): R_f = 0.20, the ratio of eluents (petroleum ether: ethyl acetate, v/v): 85:15. **^{1}H NMR (400 MHz, CDCl$_3$)** δ 7.91 (d, *J* = 7.8 Hz, 2H), 7.35-7.24 (m, 5H), 6.86 (d, *J* = 7.9 Hz, 2H), 5.89 (d, *J* = 4.1 Hz, 1H), 4.65 (d, *J* = 5.3 Hz, 1H), 3.81 (s, 3H). **^{13}C NMR (101 MHz, CDCl$_3$)** δ 197.1, 164.0, 139.6, 131.5, 129.0, 128.4, 127.7, 126.2, 113.9, 75.7, 55.4. **IR (neat):** 3477, 1661 cm^{-1}. **HRMS (ESI)** calcd for $C_{15}H_{15}O_{3}$ $[M+H]^+$: 243.1016, found: 243.1012.

2-hydroxy-1-(4-methoxyphenyl)-2-(4-(methylthio)phenyl)ethan-1-one (4u)

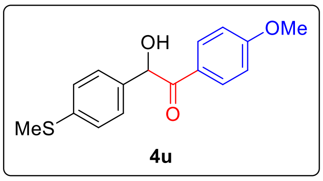

The general procedure B was followed by using 4-(methylthio)benzaldehyde (31.0 mg, 0.20 mmol, 1 equiv), cyclohexyl isocyanide (37 µl, 0.3 mmol, 1.5 equiv) and (4-methoxyphenyl)boronic acid (55.0 mg, 0.36 mmol, 1.8 equiv) to afford 26.0 mg (45%) of the product **4u** as a light yellow oil. TLC (petroleum ether: ethyl acetate, 84:16 v/v): R_f = 0.20, the ratio of eluents (petroleum ether: ethyl acetate, v/v): 85:15. **^{1}H NMR (400 MHz, $CDCl_3$)** δ 7.89 (d, *J* = 8.5 Hz, 2H), 7.24 (d, *J* = 9.4 Hz, 2H), 7.18 (d, *J* = 8.2 Hz, 2H), 6.86 (d, *J* = 7.9 Hz, 2H), 5.85 (d, *J* = 4.6 Hz, 1H), 4.61 (d, *J* = 5.5 Hz, 1H), 3.82 (s, 3H), 2.43 (s, 3H). **^{13}C NMR (101 MHz, $CDCl_3$)** δ 196.9, 164.1, 139.1, 136.2, 131.5, 128.1, 126.8, 126.1, 113.9, 75.2, 55.5, 15.5. **IR (neat):** 3442, 1665 cm^{-1}. **HRMS (ESI)** calcd for $C_{16}H_{17}O_3S$ $[M+H]^+$: 289.0893, found: 289.0894.

2-hydroxy-1-(4-methoxyphenyl)-2-(p-tolyl)ethan-1-one (4v)

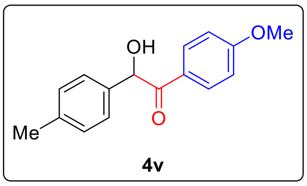

The general procedure B was followed by using 4-methylbenzaldehyde (24.0 mg, 0.20 mmol, 1 equiv), cyclohexyl isocyanide (37 µl, 0.3 mmol, 1.5 equiv) and (4-methoxyphenyl)boronic acid (55.0 mg, 0.36 mmol, 1.8 equiv) to afford 24.0 mg (47%) of the product **4v** as a colorless oil. TLC (petroleum ether: ethyl acetate, 84:16 v/v): R_f = 0.25, the ratio of eluents (petroleum ether: ethyl acetate, v/v): 90:10. **^{1}H NMR (400 MHz, $CDCl_3$)** δ 7.90 (d, *J* = 8.5 Hz, 2H), 7.22 (d, *J* = 7.5 Hz, 2H), 7.12 (d, *J* = 7.6 Hz, 2H), 6.86 (d, *J* = 8.4 Hz, 2H), 5.86 (s, 1H), 4.59 (s, 1H), 3.81 (s, 3H), 2.29 (s, 3H). **^{13}C NMR (101 MHz, $CDCl_3$)** δ 197.3, 164.0, 138.3, 136.6, 131.5, 129.7, 127.6, 126.3, 113.9, 75.5, 55.4, 21.1. **IR (neat):** 3431, 1664 cm^{-1}. **HRMS (ESI)** calcd for $C_{16}H_{17}O_3$ $[M+H]^+$: 257.1172, found: 257.1170.

2-hydroxy-1-(4-methoxyphenyl)-2-(m-tolyl)ethan-1-one (4w)

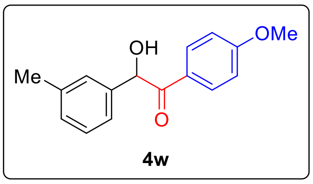

The general procedure B was followed by using 3-methylbenzaldehyde (24.0 mg, 0.20 mmol, 1 equiv), cyclohexyl isocyanide (37 µl, 0.3 mmol, 1.5 equiv) and (4-methoxyphenyl)boronic acid (55.0 mg, 0.36 mmol, 1.8 equiv) to afford 25.0 mg (48%) of the product **4w** as a colorless oil. TLC (petroleum ether: ethyl acetate, 84:16 v/v): R_f = 0.25, the ratio of eluents (petroleum ether: ethyl acetate, v/v): 90:10. **^{1}H NMR (400 MHz, $CDCl_3$)** δ 7.92 (d, *J* = 8.7 Hz, 2H), 7.21 (t, *J* = 7.7 Hz, 1H), 7.13 (s, 2H), 7.08 (d, *J* = 7.4 Hz, 1H), 6.86 (d, *J* = 8.7 Hz, 2H), 5.85 (d, *J* = 4.8 Hz, 1H), 4.63 (d, *J* = 5.6 Hz, 1H), 3.81 (s, 3H), 2.30 (s, 3H). **^{13}C NMR (101 MHz, $CDCl_3$)** δ 197.2, 164.0, 139.4, 138.8, 131.5, 129.2, 128.9, 128.2, 126.2, 124.8, 113.9, 75.8, 55.4, 21.3. **IR (neat):** 3468, 1670 cm^{-1}. **HRMS (ESI)** calcd for $C_{16}H_{17}O_3$ $[M+H]^+$: 257.1172, found: 257.1171.

2-(3,4-dimethylphenyl)-2-hydroxy-1-(4-methoxyphenyl)ethan-1-one (4x)

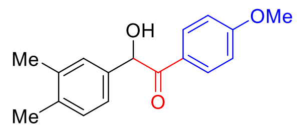

4x

The general procedure B was followed by using 3,4-dimethylbenzaldehyde (27.0 mg, 0.20 mmol, 1 equiv), cyclohexyl isocyanide (37 μl, 0.3 mmol, 1.5 equiv) and (4-methoxyphenyl)boronic acid (55.0 mg, 0.36 mmol, 1.8 equiv) to afford 29.0 mg (53%) of the product **4x** as a colorless oil. TLC (petroleum ether: ethyl acetate, 84:16 v/v): R_f = 0.25, the ratio of eluents (petroleum ether: ethyl acetate, v/v): 90:10. **^{1}H NMR (400 MHz, $CDCl_3$)** δ 7.92 (d, *J* = 8.7 Hz, 2H), 7.07 (s, 3H), 6.86 (d, *J* = 8.7 Hz, 2H), 5.83 (d, *J* = 5.7 Hz, 1H), 4.57 (d, *J* = 6.0 Hz, 1H), 3.81 (s, 3H), 2.21 (s, 3H), 2.19 (s, 3H). **^{13}C NMR (101 MHz, $CDCl_3$)** δ 197.3, 163.9, 137.4, 137.0, 137.0, 131.5, 130.2, 128.7, 126.3, 125.2, 113.9, 75.6, 55.4, 19.8, 19.5. **IR (neat):** 3454, 1670 cm^{-1}. **HRMS (ESI)** calcd for $C_{17}H_{19}O_3$ $[M+H]^+$: 271.1329, found: 271.1322.

2-(3,5-dimethylphenyl)-2-hydroxy-1-(4-methoxyphenyl)ethan-1-one (4y)

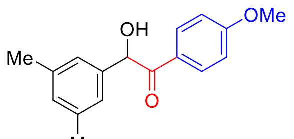

4y

The general procedure B was followed by using 3,5-dimethylbenzaldehyde (27.0 mg, 0.20 mmol, 1 equiv), cyclohexyl isocyanide (37 μl, 0.3 mmol, 1.5 equiv) and (4-methoxyphenyl)boronic acid (55.0 mg, 0.36 mmol, 1.8 equiv) to afford 28.0 mg (51%) of the product **4y** as a colorless oil. TLC (petroleum ether: ethyl acetate, 84:16 v/v): R_f = 0.30, the ratio of eluents (petroleum ether: ethyl acetate, v/v): 90:10. **^{1}H NMR (400 MHz, $CDCl_3$)** δ 7.92 (d, *J* = 8.6 Hz, 2H), 6.93 (s, 2H), 6.90 (s, 1H), 6.87 (d, *J* = 8.6 Hz, 2H), 5.80 (d, *J* = 5.8 Hz, 1H), 4.57 (d, *J* = 6.0 Hz, 1H), 3.82 (s, 3H), 2.26 (s, 6H).**^{13}C NMR (101 MHz, $CDCl_3$)** δ 197.2, 164.0, 139.4, 138.7, 131.6, 130.2, 126.3, 125.4, 113.9, 75.8, 55.4, 21.2. **IR (neat):** 3456, 1655 cm^{-1}. **HRMS (ESI)** calcd for $C_{17}H_{19}O_3$ $[M+H]^+$: 271.1329, found: 271.1326.

methyl 4-(1-hydroxy-2-(4-methoxyphenyl)-2-oxoethyl)benzoate (4z)[2]

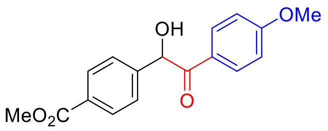

4z

The general procedure B was followed by using methyl 4-formylbenzoate (33.0 mg, 0.20 mmol, 1 equiv), cyclohexyl isocyanide (37 μl, 0.3 mmol, 1.5 equiv) and (4-methoxyphenyl)boronic acid (55.0 mg, 0.36 mmol, 1.8 equiv) to afford 36.0 mg (60%) of the product **4z** as a light yellow oil. TLC (petroleum ether: ethyl acetate, 84:16 v/v): R_f = 0.15, the ratio of eluents (petroleum ether: ethyl acetate, v/v): 80:20. **^{1}H NMR (400 MHz, $CDCl_3$)** δ 7.98 (d, *J* = 7.9 Hz, 2H), 7.88 (d, *J* = 8.2 Hz, 2H), 7.41 (d, *J* = 7.9 Hz, 2H), 6.86 (d, *J* = 8.2 Hz, 2H), 5.93 (s, 1H), 4.72 (s, 1H), 3.87 (s, 3H), 3.81 (s, 3H). **^{13}C NMR (101 MHz, $CDCl_3$)** δ 196.5, 166.5, 164.2, 144.3, 131.5, 130.3, 130.2, 127.7, 125.9, 114.0, 75.2, 55.5, 52.1. **IR (neat):** 3425, 1720, 1664 cm^{-1}.

Methyl 3-(1-hydroxy-2-(4-methoxyphenyl)-2-oxoethyl)benzoate (4aa)

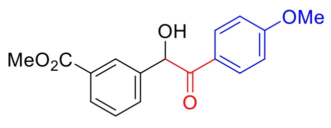

4aa

The general procedure B was followed by using methyl 3-formylbenzoate (33.0 mg, 0.20 mmol, 1 equiv), cyclohexyl isocyanide (37 µl, 0.3 mmol, 1.5 equiv) and (4-methoxyphenyl)boronic acid (55.0 mg, 0.36 mmol, 1.8 equiv) to afford 25.0 mg (42%) of the product **4aa** as a colorless oil. TLC (petroleum ether: ethyl acetate, 84:16 v/v): R_f = 0.15, the ratio of eluents (petroleum ether: ethyl acetate, v/v): 80:20. **^{1}H NMR (400 MHz, $CDCl_3$)** δ 8.05 (s, 1H), 7.94 (d, *J* = 7.7 Hz, 1H), 7.90 (d, *J* = 8.1 Hz, 2H), 7.50 (d, *J* = 7.7 Hz, 1H), 7.39 (t, *J* = 7.7 Hz, 1H), 6.86 (d, *J* = 8.1 Hz, 2H), 5.94 (d, *J* = 5.2 Hz, 1H), 4.71 (d, *J* = 5.7 Hz, 1H), 3.89 (s, 3H), 3.81 (s, 3H). **^{13}C NMR (101 MHz, $CDCl_3$)** δ 196.6, 166.5, 164.2, 140.1, 132.0, 131.6, 130.9, 129.6, 129.2, 128.9, 125.9, 114.0, 75.3, 55.5, 52.2. **IR (neat):** 3382, 1720, 1663 cm^{-1}. **HRMS (ESI)** calcd for $C_{17}H_{17}O_5$ $[M+H]^+$: 301.1071, found:301.1073.

2-(4-fluoro-3-methylphenyl)-2-hydroxy-1-(4-methoxyphenyl)ethan-1-one (4ab)

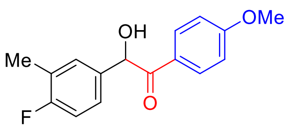

4ab

The general procedure B was followed by using 4-fluoro-3-methylbenzaldehyde (27.0 mg, 0.20 mmol, 1 equiv), cyclohexyl isocyanide (37 µl, 0.3 mmol, 1.5 equiv) and (4-methoxyphenyl)boronic acid (55.0 mg, 0.36 mmol, 1.8 equiv) to afford 27.0 mg (49%) of the product **4ab** as a colorless oil. TLC (petroleum ether: ethyl acetate, 84:16 v/v): R_f = 0.20, the ratio of eluents (petroleum ether: ethyl acetate, v/v): 90:10. **^{1}H NMR (400 MHz, $CDCl_3$)** δ 7.89 (d, *J* = 8.7 Hz, 2H), 7.19-7.08 (m, 2H), 6.94 (t, *J* = 8.8 Hz, 1H), 6.87 (d, *J* = 8.7 Hz, 2H), 5.83 (d, *J* = 5.8 Hz, 1H), 4.61 (d, *J* = 5.9 Hz, 1H), 3.82 (s, 3H), 2.21 (s, 3H). **^{13}C NMR (101 MHz, $CDCl_3$)** δ 197.0, 164.1, 161.2 (d, *J* = 245.0 Hz), 135.2 (d, *J* = 3.7 Hz), 131.5, 130.7 (d, *J* = 5.5 Hz), 126.8 (d, *J* = 8.4 Hz), 126.1, 125.8 (d, *J* = 17.7 Hz), 115.5 (d, *J* = 22.0 Hz), 113.9, 75.0, 55.5, 14.54 (d, *J* = 3.5 Hz). **IR (neat):** 3412, 1665 cm^{-1}. **HRMS (ESI)** calcd for $C_{16}H_{16}FO_3$ $[M+H]^+$: 275.1078, found: 275.1076.

2-(3-chlorophenyl)-2-hydroxy-1-(4-methoxyphenyl)ethan-1-one (4ac)

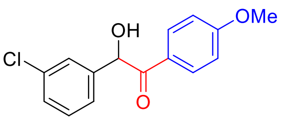

4ac

The general procedure B was followed by using 3-chlorobenzaldehyde (28.0 mg, 0.20 mmol, 1 equiv), cyclohexyl isocyanide (37 µl, 0.3 mmol, 1.5 equiv) and (4-methoxyphenyl)boronic acid (55.0 mg, 0.36 mmol, 1.8 equiv) to afford 27.0 mg (49%) of the product **4ac** as a white solid. mp: 76-78 °C. TLC (petroleum ether: ethyl acetate, 84:16 v/v): R_f = 0.25, the ratio of eluents (petroleum ether: ethyl acetate, v/v): 90:10. **^{1}H NMR (400 MHz, $CDCl_3$)** δ 7.89 (d, *J* = 7.7 Hz, 2H), 7.34 (s, 1H), 7.28-7.18 (m, 3H), 6.88 (d, *J* = 7.8 Hz, 2H), 5.86 (d, *J* = 4.1 Hz, 1H), 4.66 (d, *J* = 5.3 Hz, 1H), 3.83 (s, 3H). **^{13}C NMR (101 MHz, $CDCl_3$)** δ 196.5, 164.3, 141.5, 134.8, 131.5, 130.3, 128.6, 127.8, 125.9. 125.8, 114.1, 75.0, 55.5. **IR (neat):** 3308, 1670 cm^{-1}. **HRMS (ESI)** calcd for $C_{15}H_{13}ClO_3$ $[M+H]^+$: 299.0445, found: 299.0448.

2-(4-bromophenyl)-2-hydroxy-1-(4-methoxyphenyl)ethan-1-one (4ad)

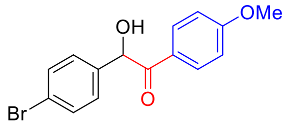

4ad

The general procedure B was followed by using 4-bromobenzaldehyde (37.0 mg, 0.20 mmol, 1 equiv), cyclohexyl isocyanide (37 μl, 0.3 mmol, 1.5 equiv) and (4-methoxyphenyl)boronic acid (55.0 mg, 0.36 mmol, 1.8 equiv) to afford 32.0 mg (50%) of the product **4ad** as a colorless oil. TLC (petroleum ether: ethyl acetate, 84:16 v/v): R_f = 0.25, the ratio of eluents (petroleum ether: ethyl acetate, v/v): 90:10. ^{1}H NMR (400 MHz, $CDCl_3$) δ 7.87 (d, *J* = 8.1 Hz, 2H), 7.44 (d, *J* = 7.6 Hz, 2H), 7.21 (d, *J* = 7.9 Hz, 2H), 6.87 (d, *J* = 8.0 Hz, 2H), 5.85 (s, 1H), 4.65 (d, *J* = 3.9 Hz, 1H), 3.83 (s, 3H). ^{13}C NMR (101 MHz, $CDCl_3$) δ 196.7, 164.2, 138.6, 132.2, 131.5, 129.3, 125.9, 122.5, 114.0, 74.9, 55.5. **IR (neat):** 3427, 1670 cm^{-1}. **HRMS (ESI)** calcd for $C_{15}H_{14}BrO_2$ $[M+H]^+$: 321.0121, found: 321.0120.

2-hydroxy-2-(3-iodophenyl)-1-(4-methoxyphenyl)ethan-1-one (4ae)

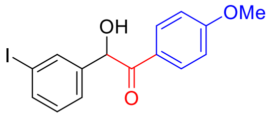

4ae

The general procedure B was followed by using 3-iodobenzaldehyde (46.0 mg, 0.20 mmol, 1 equiv), cyclohexyl isocyanide (37 μl, 0.3 mmol, 1.5 equiv) and (4-methoxyphenyl)boronic acid (55.0 mg, 0.36 mmol, 1.8 equiv) to afford 51.0 mg (69%) of the product **4ae** as a colorless oil. TLC (petroleum ether: ethyl acetate, 84:16 v/v): R_f = 0.25, the ratio of eluents (petroleum ether: ethyl acetate, v/v): 90:10. ^{1}H NMR (400 MHz, $CDCl_3$) δ 7.89 (d, *J* = 8.6 Hz, 2H), 7.70 (s, 1H), 7.60 (d, *J* = 7.8 Hz, 1H), 7.28 (d, *J* = 7.8 Hz, 1H), 7.04 (t, *J* = 7.8 Hz, 1H), 6.89 (d, *J* = 8.6 Hz, 2H), 5.81 (d, *J* = 4.7 Hz, 1H), 4.64 (d, *J* = 5.5 Hz, 1H), 3.83 (s, 3H). ^{13}C NMR (101 MHz, $CDCl_3$) δ 196.4, 164.3, 141.7, 137.5, 136.6, 131.6, 130.7, 126.9, 125.9, 114.1, 94.8, 74.9, 55.5. **IR (neat):** 3322, 1667 cm^{-1}. **HRMS (ESI)** calcd for $C_{15}H_{14}IO_2$ $[M+H]^+$: 368.9982, found: 368.9981.

2-hydroxy-1-(4-methoxyphenyl)-2-(naphthalen-2-yl)ethan-1-one (4af)

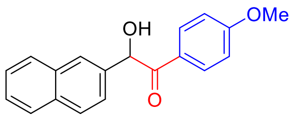

4af

The general procedure B was followed by using 2-naphthaldehyde (35.0 mg, 0.20 mmol, 1 equiv), cyclohexyl isocyanide (37 μl, 0.3 mmol, 1.5 equiv) and (4-methoxyphenyl)boronic acid (55.0 mg, 0.36 mmol, 1.8 equiv) to afford 36.0 mg (62%) of the product **4af** as a yellow oil. TLC (petroleum ether: ethyl acetate, 84:16 v/v): R_f = 0.20, the ratio of eluents (petroleum ether: ethyl acetate, v/v): 85:15. **^{1}H NMR (400 MHz, $CDCl_3$)** δ 7.95 (d, *J* = 8.2 Hz, 2H), 7.86 (s, 1H), 7.85-7.73 (m, 3H), 7.54-7.43 (m, 2H), 7.39 (d, *J* = 8.4 Hz, 1H), 6.83 (d, *J* = 8.2 Hz, 2H), 6.06 (d, *J* = 5.6 Hz, 1H), 4.76 (d, *J* = 5.9 Hz, 1H), 3.78 (s, 3H). **^{13}C NMR (101 MHz, $CDCl_3$)** δ 197.1, 164.0, 136.9, 133.4, 133.1, 131.6, 129.0, 128.0, 127.7, 127.4, 126.4, 126.3, 126.2, 124.8, 113.9, 75.9, 55.4. **IR (neat):** 3453, 1664 cm^{-1}. **HRMS (ESI)** calcd for $C_{19}H_{17}O_3$ $[M+H]^+$: 293.1172, found: 293.1163.

2-hydroxy-1-(4-methoxyphenyl)-2-(naphthalen-1-yl)ethan-1-one (4ag)

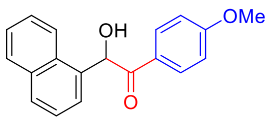

4ag

The general procedure B was followed by using 1-naphthaldehyde (35.0 mg, 0.20 mmol, 1 equiv), cyclohexyl isocyanide (37 μl, 0.3 mmol, 1.5 equiv) and (4-methoxyphenyl)boronic acid (55.0 mg, 0.36 mmol, 1.8 equiv) to afford 32.0 mg (55%) of the product **4ag** as a colorless oil. TLC (petroleum ether: ethyl acetate, 84:16 v/v): R_f = 0.20, the ratio of eluents (petroleum ether: ethyl acetate, v/v): 85:15. **^{1}H NMR (400 MHz, $CDCl_3$)** δ 8.36 (d, J = 8.5 Hz, 1H), 7.92-7.76 (m, 4H), 7.63 (t, J = 7.6 Hz, 1H), 7.54 (t, J = 7.4 Hz, 1H), 7.35 (t, J = 7.6 Hz, 1H), 7.25 (d, J = 8.9 Hz, 1H), 6.77 (d, J = 8.2 Hz, 2H), 6.54 (d, J = 2.2 Hz, 1H), 4.64 (d, J = 4.1 Hz, 1H), 3.76 (s, 3H). **^{13}C NMR (101 MHz, $CDCl_3$)** δ 198.3, 164.0, 135.7, 134.3, 131.4, 129.4, 128.9, 127.0, 126.7, 126.5, 126.1, 125.4, 123.3, 113.8, 73.3, 55.4. **IR (neat):** 3453, 1658 cm^{-1}. **HRMS (ESI)** calcd for $C_{19}H_{17}O_3$ $[M+H]^+$: 293.1172, found: 293.1168.

1-(3,4-dimethylphenyl)-2-hydroxy-4-phenylbutan-1-one (5a)

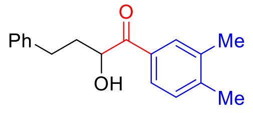

5a

The general procedure A was followed by using 3-phenylpropanal (27.0 mg, 0.20 mmol, 1 equiv), tertbutyl isocyanide (34 μl, 0.3 mmol, 1.5 equiv) and (3,4-dimethylphenyl)boronic acid (54.0 mg, 0.36 mmol, 1.8 equiv) to afford 38.1 mg (71%) of the product **5a** as a light yellow oil. TLC (petroleum ether: ethyl acetate, 84:16 v/v): R_f = 0.50, the ratio of eluents (petroleum ether: ethyl acetate, v/v): 95:5. **^{1}H NMR (400 MHz, $CDCl_3$)** δ 7.50 (d, J = 10.0 Hz, 2H), 7.33-7.26 (m, 2H), 7.23-7.16 (m, 4H), 4.99 (d, J = 8.6 Hz, 1H), 2.88-2.74 (m, 2H), 2.31 (s, 3H), 2.27 (s, 3H), 2.19-2.10 (m, 1H), 1.85-1.76 (m, 1H). **^{13}C NMR (101 MHz, $CDCl_3$)** δ 201.7, 143.7, 141.2, 137.3, 131.2, 130.0, 129.6, 128.7, 128.4, 126.2, 126.1, 71.9, 37.9, 31.3, 20.1, 19.7. **IR (neat):** 3471, 1674 cm^{-1}. **HRMS (ESI)** calcd for $C_{18}H_{20}O_2$ $[M+H]^+$: 269.1536, found: 269.1541.

2-hydroxy-4-phenyl-1-(p-tolyl)butan-1-one (5b)

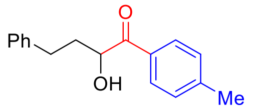

5b

The general procedure A was followed by using 3-phenylpropanal (27.0 mg, 0.20 mmol, 1 equiv), tertbutyl isocyanide (34 μl, 0.3 mmol, 1.5 equiv) and p-tolylboronic acid (49.0 mg, 0.36 mmol, 1.8 equiv) to afford 25.4 mg (50%) of the product **5b** as a colorless oil. TLC (petroleum ether: ethyl acetate, 84:16 v/v): R_f = 0.50, the ratio of eluents (petroleum ether: ethyl acetate, v/v): 95:5. **^{1}H NMR (400 MHz, $CDCl_3$)** δ 7.68 (d, J = 6.8 Hz, 2H), 7.31-7.23 (m, 4H), 7.22-7.15 (m, 3H), 5.01 (s, 1H), 3.79 (d, J = 4.8 Hz, 1H), 2.90-2.73 (m, 2H), 2.42 (s, 3H), 2.22-2.09 (m, 1H), 1.89-1.76 (m, 1H). **^{13}C NMR (151 MHz, $CDCl_3$)** δ 210.5, 145.0, 141.1, 130.8, 129.5, 128.6, 128.6, 128.4, 126.1, 72.0, 37.9, 31.3, 21.7. **IR (neat):** 3463, 1671 cm^{-1}. **HRMS (ESI)** calcd for $C_{17}H_{18}O_2$ $[M+H]^+$: 255.1380, found: 255.1380.

1-(4-(tert-butyl)phenyl)-2-hydroxy-4-phenylbutan-1-one (5c)

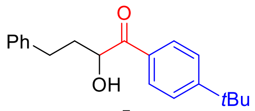

5c

The general procedure A was followed by using 3-phenylpropanal (27.0 mg, 0.20 mmol, 1 equiv), tertbutyl isocyanide (34 μl, 0.3 mmol, 1.5 equiv) and (4-(*tert*-butyl)phenyl)boronic acid (64.0 mg, 0.36 mmol, 1.8 equiv) to afford 43.7 mg (81%) of the product **5c** as a colorless oil. TLC (petroleum ether: ethyl acetate, 84:16 v/v): R_f = 0.60, the ratio of eluents (petroleum ether: ethyl acetate, v/v): 95:5. **^{1}H NMR (400 MHz, $CDCl_3$)** δ 8.19 (d, *J* = 8.1 Hz, 2H), 7.92 (d, *J* = 7.9 Hz, 2H), 7.78-7.72 (m, 2H), 7.65 (d, *J* = 7.3 Hz, 3H), 5.48 (s, 1H), 4.27 (d, *J* = 6.1 Hz, 1H), 3.43-3.19 (m, 2H), 2.71-2.56 (m, 1H), 2.37-2.21 (m, 1H), 1.80 (s, 9H). **^{13}C NMR (101 MHz, $CDCl_3$)** δ 201.4, 157.9, 141.1, 130.7, 128.6, 128.5, 128.4, 126.1, 125.8, 72.1, 37.8, 35.2, 31.3, 31.0. **IR (neat):** 3426, 1676 cm^{-1}. **HRMS (ESI)** calcd for $C_{20}H_{24}O_2$ $[M+H]^+$: 297.1849, found: 297.1851.

2-hydroxy-4-phenyl-1-(4-((tetrahydro-2H-pyran-2-yl)oxy)phenyl)butan-1-one (5d)

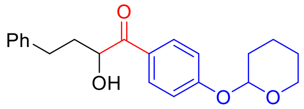

5d

The general procedure A was followed by using 3-phenylpropanal (27.0 mg, 0.20 mmol, 1 equiv), tertbutyl isocyanide (34 μl, 0.3 mmol, 1.5 equiv) and (4-((tetrahydro-2H-pyran-2-yl)oxy)phenyl)boronic acid (80.0 mg, 0.36 mmol, 1.8 equiv) to afford 43.7 mg (81%) of the product **5d** as a light yellow oil. TLC (petroleum ether: ethyl acetate, 84:16 v/v): R_f = 0.25, the ratio of eluents (petroleum ether: ethyl acetate, v/v): 90:10. **^{1}H NMR (400 MHz, $CDCl_3$)** δ 7.75 (d, *J* = 8.6 Hz, 2H), 7.33-7.26 (m, 2H), 7.18 (d, *J* = 7.0 Hz, 3H), 7.07 (d, *J* = 7.9 Hz, 2H), 5.52 (s, 1H), 4.97 (s, 1H), 3.93-3.75 (m, 2H), 3.68-3.55 (m, 1H), 2.93-2.72 (m, 2H), 2.25-2.08 (m, 1H), 2.06-1.94 (m, 1H), 1.93-1.76 (m, 3H), 1.75-1.62 (m, 2H). **^{13}C NMR (101 MHz, $CDCl_3$)** δ 200.2, 161.7, 141.2, 130.7, 128.7, 128.4, 126.7, 126.1, 116.2, 96.1, 71.8, 62.0, 38.0, 31.3, 30.0, 25.0, 18.4. **IR (neat):** 3468, 1670 cm^{-1}. **HRMS (ESI)** calcd for $C_{17}H_{18}O_3$ $[M+H]^+$: 341.1747, found: 341.1748.

2-hydroxy-1-(4-methoxy-3-methylphenyl)-4-phenylbutan-1-one (5e)

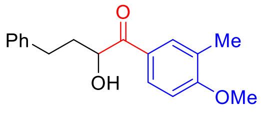

5e

The general procedure A was followed by using 3-phenylpropanal (27.0 mg, 0.20 mmol, 1 equiv), tertbutyl isocyanide (34 μl, 0.3 mmol, 1.5 equiv) and (4-methoxy-3-methylphenyl)boronic acid (60.0 mg, 0.36 mmol, 1.8 equiv) to afford 43.7 mg (81%) of the product **5e** as a light yellow oil. TLC (petroleum ether: ethyl acetate, 84:16 v/v): R_f = 0.30, the ratio of eluents (petroleum ether: ethyl acetate, v/v): 90:10. **^{1}H NMR (400 MHz, $CDCl_3$)** δ 7.65 (d, *J* = 8.5 Hz, 1H), 7.56 (s, 1H), 7.34-7.27 (m, 2H), 7.23-7.18 (m, 3H), 6.82 (d, *J* = 8.5 Hz, 1H), 4.97 (d, *J* = 8.4 Hz, 1H), 3.89 (s, 3H), 2.90-2.75 (m, 2H), 2.20 (s, 3H), 2.18-2.12 (m, 1H), 1.87-1.77 (m, 1H). **^{13}C NMR (101 MHz, $CDCl_3$)** δ 200.4, 162.4, 141.2, 131.1, 128.7, 128.7, 128.4, 127.2, 126.0, 125.7, 109.4, 71.6, 55.6, 38.1, 31.3, 16.2. **IR (neat):** 3478, 1667 cm^{-1}. **HRMS (ESI)** calcd for $C_{18}H_{20}O_3$ $[M+H]^+$: 285.1485, found: 285.1487.

1-(2,3-dihydrobenzofuran-5-yl)-2-hydroxy-4-phenylbutan-1-one (5f)

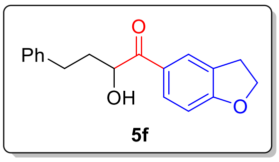

The general procedure A was followed by using 3-phenylpropanal (27.0 mg, 0.20 mmol, 1 equiv), tertbutyl isocyanide (34 μl, 0.3 mmol, 1.5 equiv) and (2,3-dihydrobenzofuran-5-yl)boronic acid (59.0 mg, 0.36 mmol, 1.8 equiv) to afford 43.7 mg (81%) of the product **5f** as a colorless oil. TLC (petroleum ether: ethyl acetate, 84:16 v/v): R_f = 0.30, the ratio of eluents (petroleum ether: ethyl acetate, v/v): 90:10. **^{1}H NMR (400 MHz, $CDCl_3$)** δ 7.64 (s, 1H), 7.59 (d, *J* = 8.4 Hz, 1H), 7.33-7.25 (m, 2H), 7.19 (d, *J* = 7.2 Hz, 3H), 6.77 (d, *J* = 8.3 Hz, 1H), 4.95 (t, *J* = 5.7 Hz, 1H), 4.66 (t, *J* = 8.7 Hz, 2H), 3.83 (d, *J* = 6.4 Hz, 1H), 3.21 (t, *J* = 8.6 Hz, 2H), 2.88-2.74 (m, 2H), 2.21-2.07 (m, 1H), 1.91-1.75 (m, 1H). **^{13}C NMR (151 MHz, $CDCl_3$)** δ 200.0, 165.1, 141.2, 130.6, 128.7, 128.4, 128.1, 126.4, 126.0, 125.9, 109.4, 72.3, 71.6, 38.2, 31.3, 28.9. **IR (neat):** 3463, 1667 cm^{-1}. **HRMS (ESI)** calcd for $C_{18}H_{18}O_3$ $[M+H]^+$: 283.1329, found: 283.1336.

1-(3,4-dimethoxyphenyl)-2-hydroxy-4-phenylbutan-1-one (5g)

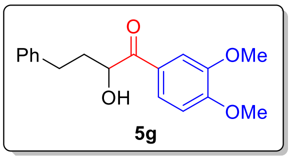

The general procedure A was followed by using 3-phenylpropanal (27.0 mg, 0.20 mmol, 1 equiv), tertbutyl isocyanide (34 μl, 0.3 mmol, 1.5 equiv) and (3,4-dimethoxyphenyl)boronic acid (66.0 mg, 0.36 mmol, 1.8 equiv) to afford 43.7 mg (81%) of the product **5g** as a colorless oil. TLC (petroleum ether: ethyl acetate, 84:16 v/v): R_f = 0.20, the ratio of eluents (petroleum ether: ethyl acetate, v/v): 85:15. **^{1}H NMR (400 MHz, $CDCl_3$)** δ 7.34 (d, *J* = 9.0 Hz, 2H), 7.31-7.25 (m, 2H), 7.23-7.16 (m, 3H), 6.84 (d, *J* = 8.2 Hz, 1H), 4.97 (t, *J* = 7.3 Hz, 1H), 3.93 (s, 3H), 3.85 (s, 3H), 3.79 (d, *J* = 6.6 Hz, 1H), 2.92-2.74 (m, 2H), 2.21-2.09 (m, 1H), 1.88-1.75 (m, 1H). **^{13}C NMR (151 MHz, $CDCl_3$)** δ 200.3, 153.9, 149.1, 141.1, 128.7, 128.4, 126.2, 126.0, 123.1, 110.4, 110.1, 71.4, 56.1, 55.9, 38.2, 31.2. **IR (neat):** 3472, 1668 cm^{-1}. **HRMS (ESI)** calcd for $C_{18}H_{20}O_4$ $[M+H]^+$: 301.1434, found: 301.1439.

1-(2,3-dihydrobenzo[b][1,4]dioxin-6-yl)-2-hydroxy-4-phenylbutan-1-one (5h)

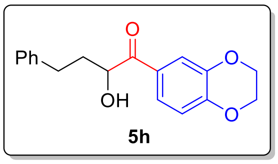

The general procedure A was followed by using 3-phenylpropanal (27.0 mg, 0.20 mmol, 1 equiv), tertbutyl isocyanide (34 μl, 0.3 mmol, 1.5 equiv) and (2,3-dihydrobenzo[b][1,4]dioxin-6-yl)boronic acid (65.0 mg, 0.36 mmol, 1.8 equiv) to afford 43.7 mg (81%) of the product **5h** as a colorless oil. TLC (petroleum ether: ethyl acetate, 84:16 v/v): R_f = 0.20, the ratio of eluents (petroleum ether: ethyl acetate, v/v): 90:10. **^{1}H NMR (400 MHz, $CDCl_3$)** δ 7.38 (s, 1H), 7.34-7.26 (m, 3H), 7.22-7.15 (m, 3H), 6.89 (d, *J* = 8.5 Hz, 1H), 4.95 (s, 1H), 4.37-4.20 (m, 4H), 3.79 (d, *J* = 5.3 Hz, 1H), 2.91-2.71 (m, 2H), 2.23-2.08 (m, 1H), 1.91-1.74 (m, 1H). **^{13}C NMR (101 MHz, $CDCl_3$)** δ 200.1, 148.7, 143.5, 141.1, 128.5, 128.4, 127.0, 126.0, 122.6, 117.9, 117.5, 71.9, 64.7, 64.0, 37.9, 31.2. **IR (neat):** 3456, 1672 cm^{-1}. **HRMS (ESI)** calcd for $C_{18}H_{18}O_4$ $[M+H]^+$: 299.1278, found: 299.1278.

1-(benzo[d][1,3]dioxol-5-yl)-2-hydroxy-4-phenylbutan-1-one (5i)

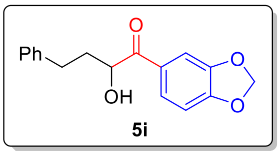

The general procedure A was followed by using 3-phenylpropanal (27.0 mg, 0.20 mmol, 1 equiv), tertbutyl isocyanide (34 μl, 0.3 mmol, 1.5 equiv) and benzo[d][1,3]dioxol-5-ylboronic acid (60.0 mg, 0.36 mmol, 1.8 equiv) to afford 43.7 mg (81%) of the product **5i** as a light yellow oil. TLC (petroleum ether: ethyl acetate, 84:16 v/v): R_f = 0.25, the ratio of eluents (petroleum ether: ethyl acetate, v/v): 90:10. **^{1}H NMR (400 MHz, $CDCl_3$)** δ 7.35-7.27 (m, 4H), 7.20 (t, *J* = 7.3 Hz, 3H), 6.82 (d, *J* = 8.0 Hz, 1H), 6.05 (s, 2H), 4.93 (d, *J* = 7.8 Hz, 1H), 3.77 (s, 1H), 2.91-2.73 (m, 2H), 2.21-2.08 (m, 1H), 1.87-1.78 (m, 1H).**^{13}C NMR (101 MHz, $CDCl_3$)** δ 199.8, 152.5, 148.3, 141.1, 128.6, 128.4, 127.9, 126.1, 124.9, 108.2, 108.2, 102.0, 71.84, 38.1, 31.3. **IR (neat):** 3476, 1672 cm^{-1}. **HRMS (ESI)** calcd for $C_{17}H_{16}O_4$[M+H]$^+$:285.1121, found:285.1122.

1-(4-(allyloxy)phenyl)-2-hydroxy-4-phenylbutan-1-one (5j)

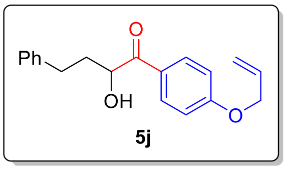

The general procedure A was followed by using 3-phenylpropanal (27.0 mg, 0.20 mmol, 1 equiv), tertbutyl isocyanide (34 μl, 0.3 mmol, 1.5 equiv) and (4-(allyloxy)phenyl)boronic acid (64.0 mg, 0.36 mmol, 1.8 equiv) to afford 43.7 mg (81%) of the product **5j** as a colorless oil. TLC (petroleum ether: ethyl acetate, 84:16 v/v): R_f = 0.40, the ratio of eluents (petroleum ether: ethyl acetate, v/v): 93:7. **^{1}H NMR (400 MHz, $CDCl_3$)** δ 7.76 (d, *J* = 8.2 Hz, 2H), 7.34-7.25 (m, 2H), 7.24-7.15 (m, 3H), 6.93 (d, *J* = 8.2 Hz, 2H), 6.11-5.99 (m, 1H), 5.43 (d, *J* = 17.2 Hz, 1H), 5.33 (d, *J* = 10.5 Hz, 1H), 4.98 (t, *J* = 7.2 Hz, 1H), 4.61 (d, *J* = 5.0 Hz, 2H), 3.84 (d, *J* = 6.3 Hz, 1H), 2.92-2.72 (m, 2H), 2.25-2.09 (m, 1H), 1.92-1.76 (m, 1H). **^{13}C NMR (101 MHz, $CDCl_3$)** δ 200.1, 163.1, 141.2, 132.2, 130.8, 128.6, 128.4, 126.3, 126.0, 118.3, 114.7, 71.7, 68.9, 38.0, 31.3. **IR (neat):** 3471, 1672 cm^{-1}. **HRMS (ESI)** calcd for $C_{19}H_{21}O_3$ [M+H]$^+$: 297.1485, found: 297.1484.

1-(4-(but-2-yn-1-yloxy)phenyl)-2-hydroxy-4-phenylbutan-1-one (5k)

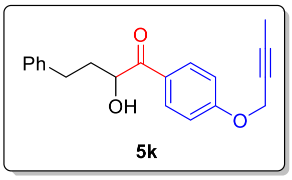

The general procedure A was followed by using 3-phenylpropanal (27.0 mg, 0.20 mmol, 1 equiv), tertbutyl isocyanide (34 μl, 0.3 mmol, 1.5 equiv) and (4-(but-2-yn-1-yloxy)phenyl)boronic acid (69.4 mg, 0.36 mmol, 1.8 equiv) to afford 43.7 mg (81%) of the product **5k** as a colorless oil. TLC (petroleum ether: ethyl acetate, 84:16 v/v): R_f = 0.30, the ratio of eluents (petroleum ether: ethyl acetate, v/v): 90:10. **^{1}H NMR (400 MHz, $CDCl_3$)** δ 7.77 (d, *J* = 7.4 Hz, 2H), 7.31-7.24 (m, 2H), 7.23-7.15 (m, 3H), 6.98 (d, *J* = 7.5 Hz, 2H), 4.98 (t, *J* = 7.0 Hz, 1H), 4.71 (s, 2H), 3.81 (d, *J* = 6.0 Hz, 1H), 2.91-2.72 (m, 2H), 2.24-2.06 (m, 1H), 1.91-1.73 (m, 4H). **^{13}C NMR (101 MHz, $CDCl_3$)** δ 200.2, 162.3, 141.2, 130.7, 128.6, 128.4, 126.6, 126.0, 114.9, 84.7, 73.1, 71.8, 56.6, 38.0, 31.3. **IR (neat):** 3464, 1669 cm^{-1}. **HRMS (ESI)** calcd for $C_{20}H_{21}O_3$ [M+H]$^+$: 309.1485, found: 309.1484.

1-(4-(diphenylamino)phenyl)-2-hydroxy-4-phenylbutan-1-one (5l)

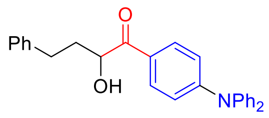

5l

The general procedure A was followed by using 3-phenylpropanal (27.0 mg, 0.20 mmol, 1 equiv), tertbutyl isocyanide (34 μl, 0.3 mmol, 1.5 equiv) and (4-(diphenylamino)phenyl)boronic acid (104.0 mg, 0.36 mmol, 1.8 equiv) to afford 43.7 mg (81%) of the product **5l** as a yellow oil. TLC (petroleum ether: ethyl acetate, 84:16 v/v): R_f = 0.35, the ratio of eluents (petroleum ether: ethyl acetate, v/v): 90:10. **^{1}H NMR (400 MHz, $CDCl_3$)** δ 7.66 (d, *J* = 8.1 Hz, 2H), 7.42-7.32 (m, 4H), 7.31-7.27 (m, 2H), 7.24-7.16 (m, 9H), 6.96 (d, *J* = 8.1 Hz, 2H), 4.97 (s, 1H), 3.93 (d, *J* = 4.3 Hz, 1H), 2.94-2.72 (m, 2H), 2.28-2.10 (m, 1H), 1.92-1.80 (m, 1H). **^{13}C NMR (151 MHz, $CDCl_3$)** δ 199.5, 152.8, 146.0, 141.2, 130.2, 129.7, 128.6, 128.4, 126.2, 126.0, 125.1, 124.9, 118.9, 71.6, 38.1, 31.3. **IR (neat):** 3461, 1662 cm^{-1}. **HRMS (ESI)** calcd for $C_{28}H_{26}NO_2$ $[M+H]^+$: 408.1958, found: 408.1968.

1-(furan-2-yl)-2-hydroxy-4-phenylbutan-1-one (5m)

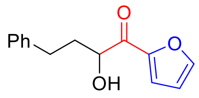

5m

The general procedure A was followed by using 3-phenylpropanal (27.0 mg, 0.20 mmol, 1 equiv), tertbutyl isocyanide (34 μl, 0.3 mmol, 1.5 equiv) and (4-methoxyphenyl)boronic acid (55.0 mg, 0.36 mmol, 1.8 equiv) to afford 43.7 mg (81%) of the product **5m** as a red oil. TLC (petroleum ether: ethyl acetate, 84:16 v/v): R_f = 0.33, the ratio of eluents (petroleum ether: ethyl acetate, v/v): 90:10. **^{1}H NMR (400 MHz, $CDCl_3$)** δ 7.62 (s, 1H), 7.33-7.26 (m, 2H), 7.24-7.19 (m, 4H), 6.56 (s, 1H), 4.83 (d, *J* = 7.2 Hz, 1H), 3.62 (s, 1H), 2.91-2.80 (m, 2H), 2.31-2.20 (m, 1H), 2.00-1.89 (m, 1H). ^{13}C NMR (101 MHz, $CDCl_3$) δ 190.2, 150.1, 147.1, 141.1, 128.5, 128.3, 126.0, 119.0, 112.5, 72.7, 37.3, 31.2. **IR (neat):** 3459, 1658 cm^{-1}. **HRMS (ESI)** calcd for $C_{14}H_{14}O_3$ $[M+H]^+$: 231.1016, found: 231.1018.

2-hydroxy-4-phenyl-1-(thiophen-2-yl)butan-1-one (5n)

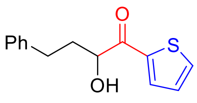

5n

The general procedure A was followed by using 3-phenylpropanal (27.0 mg, 0.20 mmol, 1 equiv), tertbutyl isocyanide (34 μl, 0.3 mmol, 1.5 equiv) and thiophen-2-ylboronic acid (46.0 mg, 0.36 mmol, 1.8 equiv) to afford 43.7 mg (81%) of the product **5n** as a yellow oil. TLC (petroleum ether: ethyl acetate, 84:16 v/v): R_f = 0.30, the ratio of eluents (petroleum ether: ethyl acetate, v/v): 90:10. **^{1}H NMR (400 MHz, $CDCl_3$)** δ 7.71 (d, *J* = 4.5 Hz, 1H), 7.55 (s, 1H), 7.35-7.27 (m, 2H), 7.25-7.18 (m, 3H), 7.13 (s, 1H), 4.85 (s, 1H), 3.60 (s, 1H), 2.93-2.78 (m, 2H), 2.31-2.18 (m, 1H), 2.02-1.90 (m, 1H). **^{13}C NMR (101 MHz, $CDCl_3$)** δ 194.4, 141.0, 139.7, 134.7, 132.9, 128.6, 128.5, 128.3, 126.1, 73.0, 38.6, 31.2. **IR (neat):** 3478, 1648 cm^{-1}. **HRMS (ESI)** calcd for $C_{14}H_{14}O_2S[M+H]^+$: 247.0787, found: 247.0788.

2-hydroxy-4-phenyl-1-(thiophen-3-yl)butan-1-one (5o)

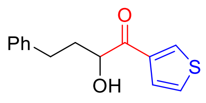

The general procedure A was followed by using 3-phenylpropanal (27.0 mg, 0.20 mmol, 1 equiv), tertbutyl isocyanide (34 μl, 0.3 mmol, 1.5 equiv) and thiophen-3-ylboronic acid (46.0 mg, 0.36 mmol, 1.8 equiv) to afford 43.7 mg (81%) of the product **5o** as a yellow oil. TLC (petroleum ether: ethyl acetate, 84:16 v/v): R_f = 0.40, the ratio of eluents (petroleum ether: ethyl acetate, v/v): 93:7. **^{1}H NMR (400 MHz, $CDCl_3$)** δ 7.91 (s, 1H), 7.43 (d, *J* = 4.5 Hz, 1H), 7.35-7.27 (m, 3H), 7.23-7.17 (m, 3H), 4.81 (d, *J* = 7.1 Hz, 1H), 3.65 (s, 1H), 2.92-2.77 (m, 2H), 2.25-2.14 (m, 1H), 1.95-1.78 (m, 1H).**^{13}C NMR (101 MHz, $CDCl_3$)** δ 195.9, 141.0, 137.9, 133.1, 128.7, 128.5, 126.9, 126.8, 126.2, 731, 38.0, 31.3. **IR (neat):** 3468, 1671 cm^{-1}. **HRMS (ESI)** calcd for $C_{14}H_{14}O_2S$[M+H]$^+$:247.0787, found:247.0789.

1-(5-bromothiophen-2-yl)-2-hydroxy-4-phenylbutan-1-one (5p)

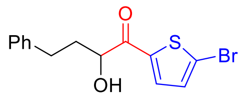

The general procedure A was followed by using 3-phenylpropanal (27.0 mg, 0.20 mmol, 1 equiv), tertbutyl isocyanide (34 μl, 0.3 mmol, 1.5 equiv) and (5-bromothiophen-2-yl)boronic acid (74.2 mg, 0.36 mmol, 1.8 equiv) to afford 43.7 mg (81%) of the product **5p** as a yellow oil. TLC (petroleum ether: ethyl acetate, 84:16 v/v): R_f = 0.40, the ratio of eluents (petroleum ether: ethyl acetate, v/v): 93:7. **^{1}H NMR (400 MHz, $CDCl_3$)** δ 7.34-7.28 (m, 2H), 7.27-7.17 (m, 4H), 7.11-7.07 (m, 1H), 4.76 (s, 1H), 3.46 (d, *J* = 5.5 Hz, 1H), 2.84 (t, *J* = 7.4 Hz, 2H), 2.28-2.12 (m, 1H), 1.99-1.90 (m, 1H).**^{13}C NMR (151 MHz, $CDCl_3$)** δ 193.3, 141.1, 140.8, 133.1, 131.4, 128.6, 128.5, 126.2, 124.1, 72.7, 38.6, 31.2. **IR (neat):** 3483, 1655 cm^{-1}. **HRMS (ESI)** calcd for $C_{14}H_{13}BrO_2S$ [M+H]$^+$:324.9892, found: 324.9901.

1-(benzofuran-2-yl)-2-hydroxy-4-phenylbutan-1-one (5q)

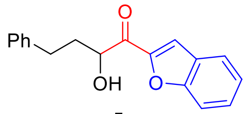

The general procedure A was followed by using 3-phenylpropanal (27.0 mg, 0.20 mmol, 1 equiv), tertbutyl isocyanide (34 μl, 0.3 mmol, 1.5 equiv) and benzofuran-2-ylboronic acid (58.4 mg, 0.36 mmol, 1.8 equiv) to afford 43.7 mg (81%) of the product **5q** as a yellow oil. TLC (petroleum ether: ethyl acetate, 84:16 v/v): R_f = 0.35, the ratio of eluents (petroleum ether: ethyl acetate, v/v): 90:10. **^{1}H NMR (400 MHz, $CDCl_3$)** δ 7.71 (d, *J* = 7.8 Hz, 1H), 7.60-7.44 (m, 3H), 7.40-7.27 (m, 3H), 7.25-7.17 (m, 3H), 4.96 (d, *J* = 8.0 Hz, 1H), 3.56 (s, 1H), 2.99-2.80 (m, 2H), 2.41-2.27 (m, 1H), 2.09-1.93 (m, 1H).**^{13}C NMR (151 MHz, $CDCl_3$)** δ 192.5, 155.7, 149.9, 141.0, 128.9, 128.6, 128.4, 126.6, 126.1, 124.2, 123.5, 114.7, 112.5, 73.2, 37.3, 31.2. **IR (neat):** 3496, 1675 cm^{-1}. **HRMS (ESI)** calcd for $C_{18}H_{16}O_3$ [M+H]$^+$:281.1172, found: 281.1178.

1-(benzo[b]thiophen-3-yl)-2-hydroxy-4-phenylbutan-1-one (5r)

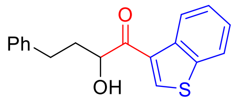

5r

The general procedure A was followed by using 3-phenylpropanal (27.0 mg, 0.20 mmol, 1 equiv), tertbutyl isocyanide (34 µl, 0.3 mmol, 1.5 equiv) and benzo[b]thiophen-3-ylboronic acid (64.0 mg, 0.36 mmol, 1.8 equiv) to afford 43.7 mg (81%) of the product **5r** as a red oil. TLC (petroleum ether: ethyl acetate, 84:16 v/v): R_f = 0.30, the ratio of eluents (petroleum ether: ethyl acetate, v/v): 90:10. **^{1}H NMR (400 MHz, CDCl$_3$)** δ 8.68 (d, *J* = 8.1 Hz, 1H), 7.96 (s, 1H), 7.88 (d, *J* = 8.0 Hz, 1H), 7.52 (t, *J* = 7.6 Hz, 1H), 7.45 (t, *J* = 7.5 Hz, 1H), 7.34-7.27 (m, 2H), 7.24-7.18 (m, 3H), 4.96 (d, *J* = 8.8 Hz, 1H), 2.94-2.80 (m, 2H), 2.25-2.16 (m, 1H), 2.00-1.91 (m, 1H). **^{13}C NMR (101 MHz, CDCl$_3$)** δ 196.5, 140.0, 139.6, 137.4, 136.5, 131.2, 128.7, 128.5, 126.1, 126.1, 125.8, 125.3, 122.3, 72.8, 38.5, 31.3. **IR (neat):** 3439, 1664 cm^{-1}. **HRMS (ESI)** calcd for $C_{18}H_{16}O_2S$ [M+H]$^+$: 297.0944, found: 297.0947.

tert-butyl 3-(2-hydroxy-4-phenylbutanoyl)-1H-indole-1-carboxylate (5s)

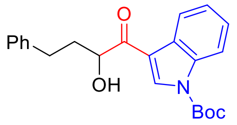

5s

The general procedure A was followed by using 3-phenylpropanal (27.0 mg, 0.20 mmol, 1 equiv), tertbutyl isocyanide (34 µl, 0.3 mmol, 1.5 equiv) and (1-(tert-butoxycarbonyl)-1H-indol-3-yl)boronic acid (94.0 mg, 0.36 mmol, 1.8 equiv) to afford 43.7 mg (81%) of the product **5s** as a yellow oil. TLC (petroleum ether: ethyl acetate, 84:16 v/v): R_f = 0.40, the ratio of eluents (petroleum ether: ethyl acetate, v/v): 90:10. **^{1}H NMR (400 MHz, CDCl$_3$)** δ 8.25 (d, *J* = 7.7 Hz, 1H), 8.15 (d, *J* = 8.0 Hz, 1H), 8.11 (s, 1H), 7.45-7.34 (m, 2H), 7.33-7.27 (m, 2H), 7.26-7.18 (m, 3H), 4.84 (dd, *J* = 8.2, 6.7 Hz, 1H), 3.83 (d, *J* = 6.2 Hz, 1H), 2.98-2.78 (m, 2H), 2.30-2.20 (m, 1H), 2.05-1.92 (m, 1H), 1.72 (s, 9H). **^{13}C NMR (101 MHz, CDCl$_3$)** δ 197.4, 148.7, 141.1, 135.3, 132.2, 128.6, 128.4, 127.2, 126.0, 125.8, 124.6, 122.3, 116.3, 115.1, 85.8, 72.9, 38.6, 31.1, 28.0. **IR (neat):** 3453, 1744 cm^{-1}. **HRMS (ESI)** calcd for $C_{23}H_{25}NO_4$ [M+H]$^+$:529.3312, found: 529.3318.

2-hydroxy-4-phenyl-1-(1-tosyl-1H-pyrrolo[2,3-b]pyridin-3-yl)butan-1-one (5t)

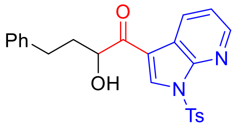

5t

The general procedure A was followed by using 3-phenylpropanal (27.0 mg, 0.20 mmol, 1 equiv), tertbutyl isocyanide (34 µl, 0.3 mmol, 1.5 equiv) and (1-tosyl-1H-pyrrolo[2,3-b]pyridin-3-yl)boronic acid (114.0 mg, 0.36 mmol, 1.8 equiv) to afford 43.7 mg (81%) of the product **5t** as a yellow oil. TLC (petroleum ether: ethyl acetate, 80:20 v/v): R_f = 0.10, the ratio of eluents (petroleum ether: ethyl acetate, v/v): 80:20. **^{1}H NMR (400 MHz, CDCl$_3$)** δ 8.51-8.45 (m, 1H), 8.41 (d, *J* = 7.9 Hz, 1H), 8.21 (s, 1H), 8.12 (d, *J* = 7.3 Hz, 2H), 7.37-7.19 (m, 8H), 4.80 (s, 1H), 3.63 (d, *J* = 4.3 Hz, 1H), 2.96-2.79 (m, 2H), 2.39 (s, 3H), 2.27-2.17 (m, 1H), 20.3-1.92 (m, 1H). **^{13}C NMR (151 MHz, CDCl$_3$)** δ 197.0, 146.8, 146.3, 146.3, 140.8, 134.1, 131.8, 131.5, 129.9, 128.6, 128.6,

126.2, 120.5, 120.1, 114.3, 72.9, 38.6, 31.1, 21.7. **IR (neat):** 3474, 1656 cm^{-1}. **HRMS (ESI)** calcd for $C_{24}H_{22}N_2O_4S$ $[M+H]^+$:435.1373, found: 435.1382.

2-hydroxy-1-(1H-indol-5-yl)-4-phenylbutan-1-one (5u)

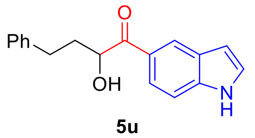

5u

The general procedure A was followed by using 3-phenylpropanal (27.0 mg, 0.20 mmol, 1 equiv), tertbutyl isocyanide (34 μl, 0.3 mmol, 1.5 equiv) and (1H-indol-5-yl)boronic acid (58.0 mg, 0.36 mmol, 1.8 equiv) to afford 43.7 mg (81%) of the product **5u** as a white solid. mp: 130-132 °C. TLC (petroleum ether: ethyl acetate, 80:20 v/v): R_f = 0.20, the ratio of eluents (petroleum ether: ethyl acetate, v/v): 85:15. **^{1}H NMR (400 MHz, $CDCl_3$)** δ 8.87 (s, 1H), 8.11 (s, 1H), 7.74 (d, J = 7.2 Hz, 1H), 7.42 (d, J = 7.3 Hz, 1H), 7.37-7.05 (m, 6H), 6.64 (s, 1H), 5.20 (s, 1H), 4.10 (d, J = 3.7 Hz, 1H), 3.08-2.70 (m, 2H), 2.26 (s, 1H), 1.96 (d, J = 13.4 Hz, 1H).**^{13}C NMR (101 MHz, $CDCl_3$)** δ 201.6, 141.3, 138.9, 128.7, 128.4, 1275, 126.0, 126.0, 125.7, 123.2, 122.4, 111.4, 104.4, 71.9, 38.4, 31.4. **IR (neat):** 3441, 1654 cm^{-1}. **HRMS (ESI)** calcd for $C_{18}H_{17}NO_2$$[M+H]^+$:280.1332, found:280.1333.

1-(benzofuran-5-yl)-2-hydroxy-4-phenylbutan-1-one (5v)

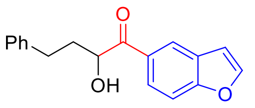

5v

The general procedure A was followed by using 3-phenylpropanal (27.0 mg, 0.20 mmol, 1 equiv), tertbutyl isocyanide (34 μl, 0.3 mmol, 1.5 equiv) and benzofuran-5-ylboronic acid (58.4 mg, 0.36 mmol, 1.8 equiv) to afford 43.7 mg (81%) of the product **5v** as a light yellow oil. TLC (petroleum ether: ethyl acetate, 84:16 v/v): R_f = 0.40, the ratio of eluents (petroleum ether: ethyl acetate, v/v): 95:5. **^{1}H NMR (400 MHz, $CDCl_3$)** δ 8.01 (s, 1H), 7.79 (d, J = 8.6 Hz, 1H), 7.71 (s, 1H), 7.54 (d, J = 8.6 Hz, 1H), 7.34-7.15 (m, 5H), 6.82 (s, 1H), 5.11 (d, J = 7.5 Hz, 1H), 3.84 (s, 1H), 2.93-2.76 (m, 2H), 2.26-2.13 (m, 1H), 1.93-1.82 (m, 1H). **^{13}C NMR (101 MHz, $CDCl_3$)** δ 201.3, 157.8, 146.7, 141.1, 128.7, 128.4, 127.7, 126.1, 125.1, 122.8, 111.9, 107.2, 72.0, 38.0, 31.3. **IR (neat):** 3478, 1675 cm^{-1}. **HRMS (ESI)** calcd for $C_{18}H_{16}O_3$$[M+H]^+$:281.1172, found:281.1174.

1-(dibenzo[b,d]thiophen-4-yl)-2-hydroxy-4-phenylbutan-1-one (5w)

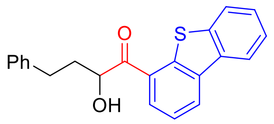

5w

The general procedure A was followed by using 3-phenylpropanal (27.0 mg, 0.20 mmol, 1 equiv), tertbutyl isocyanide (34 μl, 0.3 mmol, 1.5 equiv) and dibenzo[b,d]thiophen-4-ylboronic acid (82.0 mg, 0.36 mmol, 1.8 equiv) to afford 43.7 mg (81%) of the product **5w** as a yellow oil. TLC (petroleum ether: ethyl acetate, 84:16 v/v): R_f = 0.40, the ratio of eluents (petroleum ether: ethyl acetate, v/v): 95:5. **^{1}H NMR (400 MHz, $CDCl_3$)** δ 8.40 (d, J = 7.7 Hz, 1H), 8.19 (d, J = 7.4 Hz, 1H), 7.95 (d, J = 7.0 Hz, 1H), 7.75 (d, J = 7.6 Hz, 1H), 7.57-7.47 (m, 3H), 7.34-7.27 (m, 2H), 7.24-7.17 (m, 3H), 5.23 (t, J = 7.0 Hz, 1H), 3.82 (d, J = 6.8 Hz, 1H), 3.00-2.77 (m, 2H), 2.33-2.19 (m, 1H), 2.03-1.88 (m, 1H). **^{13}C NMR (101 MHz, $CDCl_3$)**

δ 200.8, 141.8, 140.9, 140.4, 137.7, 133.6, 128.7, 128.5, 128.3, 127.5, 126.9, 126.7, 126.2, 124.7, 124.0, 122.8, 121.4, 72.0, 38.6, 31.4. **IR (neat):** 3398, 1674 cm^{-1}. **HRMS (ESI)** calcd for $C_{22}H_{18}O_2S$ $[M+H]^+$:347.1100, found:347.1105.

1-(6,9-diphenyl-9H-carbazol-2-yl)-2-hydroxy-4-phenylbutan-1-one (5x)

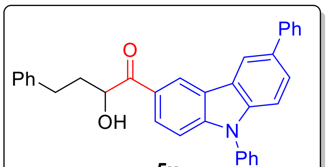

The general procedure A was followed by using 3-phenylpropanal (27.0 mg, 0.20 mmol, 1 equiv), tertbutyl isocyanide (34 μl, 0.3 mmol, 1.5 equiv) and (6,9-diphenyl-9H-carbazol-3-yl)boronic acid (131.0 mg, 0.36 mmol, 1.8 equiv) to afford 43.7 mg (81%) of the product **5x** as a white solid. mp: 151-153 °C. TLC (petroleum ether: ethyl acetate, 84:16 v/v): R_f = 0.40, the ratio of eluents (petroleum ether: ethyl acetate, v/v): 93:7. **^{1}H NMR (400 MHz, $CDCl_3$)** δ 8.55 (s, 1H), 8.32 (s, 1H), 7.96 (d, *J* = 6.5 Hz, 1H), 7.85-7.16 (m, 18H), 5.22 (s, 1H), 3.99 (s, 1H), 3.12-2.77 (m, 2H), 2.30 (s, 1H), 1.97 (s, 1H). **^{13}C NMR (101 MHz, $CDCl_3$)** δ 200.9, 144.3, 141.4, 141.2, 141.1, 136.6, 134.7, 130.1, 128.8, 128.5, 128.3, 127.3, 127.0, 126.8, 126.46, 126.1, 125.5, 123.7, 123.3, 122.1, 119.0, 110.6, 110.0, 71.5, 38.5, 31.4. **IR (neat):** 3422, 1659 cm^{-1}. **HRMS (ESI)** calcd for $C_{34}H_{28}NO_2$ $[M+H]^+$: 482.2115, found: 482.2118.

(*E*)-1-cyclohexyl-1-hydroxy-4-phenylbut-3-en-2-one (5y)

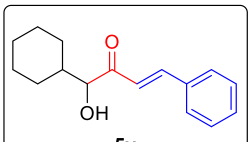

The general procedure B was followed by using cyclohexanecarbaldehyde (23.0 mg, 0.20 mmol, 1 equiv), tertbutyl isocyanide (34 μl, 0.3 mmol, 1.5 equiv) and (*E*)-styrylboronic acid (54.0 mg, 0.36 mmol, 1.8 equiv) to afford 40.0 mg (82%) of the product **5y** as a colorless oil. TLC (petroleum ether: ethyl acetate, 84:16 v/v): R_f = 0.50, the ratio of eluents (petroleum ether: ethyl acetate, v/v): 95:5. **^{1}H NMR (500 MHz, $CDCl_3$)** δ 7.76 (d, *J* = 15.9 Hz, 1H), 7.62-7.57 (m, 2H), 7.44-7.39 (m, 3H), 6.88 (d, *J* = 15.9 Hz, 1H), 4.32 (dd, *J* = 5.4, 2.8 Hz, 1H), 3.56 (d, *J* = 5.6 Hz, 1H), 1.86-1.46 (m, 5H), 1.54-1.46 (m, 1H), 1.33-1.09 (m, 5H). **^{13}C NMR (126 MHz, $CDCl_3$)** δ 200.6, 144.3, 134.1, 131.0, 129.0, 128.6, 120.7, 80.1, 41.6, 30.1, 26.5, 26.0, 25.9, 25.2. **IR (neat):** 3454, 1682 cm^{-1}. **HRMS (ESI)** calcd for $C_{16}H_{21}O_2$ $[M+H]^+$: 245.1536, found: 245.1530.

tert-butyl (*E*)-4-(7-chloro-1-hydroxy-2-oxohept-3-en-1-yl)piperidine-1-carboxylate (5z)

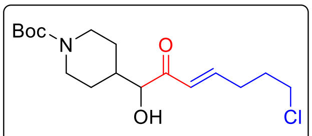

The general procedure B was followed by using tert-butyl 4-formylpiperidine-1-carboxylate (43.0 mg, 0.20 mmol, 1 equiv), tertbutyl isocyanide (34 μl, 0.3 mmol, 1.5 equiv) and (*E*)-(5-chloropent-1-en-1-yl)boronic acid (54.0 mg, 0.36 mmol, 1.8 equiv) to afford 43.0 mg (63%) of the product **5z** as a light yellow oil. TLC (petroleum ether: ethyl acetate, 84:16 v/v): R_f = 0.50, the ratio of eluents (petroleum ether: ethyl acetate, v/v): 95:5. **^{1}H NMR (500 MHz, $CDCl_3$)** δ 7.03 (dt, *J* = 15.6, 7.0 Hz, 1H), 6.32 (dt, *J* = 15.6, 1.5 Hz, 1H), 4.23 (dd, *J* = 5.6, 3.0 Hz, 1H), 4.21-3.96 (m, 2H), 3.55 (t, *J* = 6.3 Hz, 2H), 3.47 (d, *J* = 5.3 Hz, 1H),

2.74-2.52 (m, 2H), 2.45 (ddd, J = 14.7, 7.3, 1.5 Hz, 2H), 2.00-1.93 (m, 2H), 1.91-1.81 (m, 1H), 1.69-1.60 (m, 2H), 1.43 (s, 9H), 1.38-1.28 (m, 1H), 1.13 (dd, J = 13.2, 2.4 Hz, 1H). **^{13}C NMR (126 MHz, CDCl$_3$)** δ 199.6, 154.6, 148.1, 125.7, 79.4, 78.4, 43.9, 39.8, 30.5, 29.7, 28.8, 28.4. **IR (neat):** 3415, 1664 cm^{-1}. **HRMS (ESI)** calcd for $C_{17}H_{29}ClNO_4$ [M+H]$^+$: 346.1780, found: 346.1785.

4-hydroxy-1,6-diphenylhex-1-yn-3-one (7a)

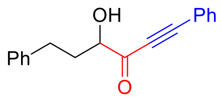

7a

The general procedure C was followed by using 3-phenylpropanal (27.0 mg, 0.20 mmol, 1 equiv), tertbutyl isocyanide (57 µl, 0.5 mmol, 2.5 equiv) and phenylacetylene trifluoroborate salt (125.0 mg, 0.6 mmol, 3 equiv) to afford 25.0 mg (47%) of the product **7a** as a light yellow oil. TLC (petroleum ether: ethyl acetate, 84:16 v/v): R_f = 0.45, the ratio of eluents (petroleum ether: ethyl acetate, v/v): 93:7. **^{1}H NMR (500 MHz, CDCl$_3$)** δ 7.59-7.56 (m, 2H), 7.52-7.49 (m, 1H), 7.43-7.39 (m, 2H), 7.32-7.29 (m, 2H), 7.26-7.20 (m, 3H), 4.40 (dd, J = 8.0, 3.7 Hz, 1H), 2.89-2.81 (m, 2H), 2.46-2.35 (m, 1H), 2.12-2.02 (m, 1H). **^{13}C NMR (126 MHz, CDCl$_3$)** δ 189.3, 141.1, 133.2, 131.3, 128.7, 128.6, 128.5, 126.1, 119.2, 96.9, 84.9, 77.5, 35.6, 31.0. **IR (neat):** 3434, 1667 cm^{-1}. **HRMS (ESI)** calcd for $C_{18}H_{17}O_2$ [M+H]$^+$: 265.1223, found: 265.1233.

4-hydroxy-6-(5-methylfuran-2-yl)-1-phenylhex-1-yn-3-one (7b)

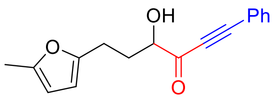

7b

The general procedure C was followed by using 3-(5-methylfuran-2-yl)propanal (27.6 mg, 0.20 mmol, 1 equiv), tertbutyl isocyanide (57 µl, 0.5 mmol, 2.5 equiv) and phenylacetylene trifluoroborate salt (125.0 mg, 0.6 mmol, 3 equiv) to afford 24.0 mg (45%) of the product **7b** as a light yellow oil. TLC (petroleum ether: ethyl acetate, 84:16 v/v): R_f = 0.50, the ratio of eluents (petroleum ether: ethyl acetate, v/v): 95:5. **^{1}H NMR (500 MHz, CDCl$_3$)** δ 7.63-7.55 (m, 2H), 7.52-7.46 (m, 1H), 7.43-7.36 (m, 2H), 5.91 (d, J = 3.0 Hz, 1H), 5.85-5.82 (m, 1H), 4.39 (dd, J = 7.9, 3.8 Hz, 1H), 2.80 (t, J = 7.5 Hz, 1H), 2.49-2.36 (m, 1H), 2.20 (d, J = 0.7 Hz, 3H), 2.11-1.97 (m, 1H). **^{13}C NMR (126 MHz, CDCl$_3$)** δ 189.1, 152.6, 150.7, 133.2, 131.3, 128.7, 119.3, 106.6, 105.9, 96.9, 84.9, 77.4, 32.4, 23.4, 13.5. **IR (neat):** 3409, 1670 cm^{-1}. **HRMS (ESI)** calcd for $C_{17}H_{17}O_3$ [M+H]$^+$: 269.1172, found: 269.1181.

1-cyclohexyl-1-hydroxy-4-phenylbut-3-yn-2-one (7c)

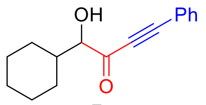

7c

The general procedure C was followed by using cyclohexanecarbaldehyde (23.0 mg, 0.20 mmol, 1 equiv), tertbutyl isocyanide (57 µl, 0.5 mmol, 2.5 equiv) and phenylacetylene trifluoroborate salt (125.0 mg, 0.6 mmol, 3 equiv) to afford 25.0 mg (52%) of the product **7c** as a light yellow oil. TLC (petroleum ether: ethyl acetate, 84:16 v/v): R_f = 0.50, the ratio of eluents (petroleum ether: ethyl acetate, v/v): 95:5. **^{1}H NMR (500 MHz, CDCl$_3$)** δ 7.63-7.59 (m, 2H), 7.52-7.48 (m, 1H), 7.45-7.40 (m, 2H), 4.26 (dd, J = 5.1, 2.8 Hz, 1H), 3.31 (d, J = 5.1 Hz, 1H),

2.17-2.08 (m, 1H), 1.89-1.73 (m, 3H), 1.71-1.63 (m, 1H), 1.53-1.44 (m, 1H), 1.42-1.30 (m, 2H), 1.28-1.23 (m,2H), 1.22-1.10 (m, 1H). **^{13}C NMR (126 MHz, $CDCl_3$)** δ 189.7, 133.2, 133.2, 131.2, 128.7, 119.4, 96.2, 85.4, 82.5, 41.8, 29.8, 26.5, 26.1, 25.9, 25.4. **IR (neat):** 3454, 1664 cm^{-1}. **HRMS (ESI)** calcd for $C_{16}H_{17}O_2$ $[M-H]^-$: 241.1234, found: 241.1236.

1-cyclopropyl-1-hydroxy-4-phenylbut-3-yn-2-one (7d)

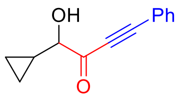

7d

The general procedure C was followed by using cyclopropanecarbaldehyde (14.0 mg, 0.20 mmol, 1 equiv), tertbutyl isocyanide (57 μl, 0.5 mmol, 2.5 equiv) and phenylacetylene trifluoroborate salt (125.0 mg, 0.6 mmol, 3 equiv) to afford 21.0 mg (53%) of the product **7c** as a light yellow oil. TLC (petroleum ether: ethyl acetate, 84:16 v/v): R_f = 0.45, the ratio of eluents (petroleum ether: ethyl acetate, v/v): 93:7. **^{1}H NMR (500 MHz, $CDCl_3$)** δ 7.62-7.59 (m, 2H), 7.52-7.48 (m, 1H), 7.44-7.39 (m, 2H), 3.92 (d, *J* = 7.3 Hz, 1H), 1.22-1.11 (m, 1H), 0.79-0.70 (m, 2H), 0.67-0.58 (m, 1H), 0.56-0.48 (m, 1H). **^{13}C NMR (126 MHz, $CDCl_3$)** δ 188.6, 133.1, 131.3, 131.3, 128.7, 119.4, 96.8, 85.7, 80.1, 14.3, 2.8, 1.9. **IR (neat):** 3458, 1663 cm^{-1}. **HRMS (ESI)** calcd for $C_{13}H_{13}O_2$ $[M+H]^+$: 201.0910, found: 201.0918.

(2S,3R)-2-hydroxy-3-(4-isobutylphenyl)-1-(4-methoxyphenyl)butan-1-one (8a) dr= 1.70:1

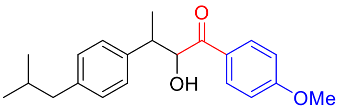

8a

The general procedure A was followed by using 2-(4-isobutylphenyl)propanal (38.0 mg, 0.20 mmol, 1 equiv), tertbutyl isocyanide (34 μl, 0.3 mmol, 1.5 equiv) and (4-methoxyphenyl)boronic acid (55.0 mg, 0.36 mmol, 1.8 equiv) to afford 44.3 mg (68%) of the product **8a** as a colorless oil. TLC (petroleum ether: ethyl acetate, 84:16 v/v): R_f = 0.33, 0.30, the ratio of eluents (petroleum ether: ethyl acetate, v/v): 90:10. **^{1}H NMR (400 MHz, $CDCl_3$)** δ 7.93 (d, *J* = 7.4 Hz, 0.63*2H), 7.80 (d, *J* = 7.4 Hz, 0.37*2H), 7.29 (d, *J* = 6.9 Hz, 0.63*2H), 7.10 (d, *J* = 7.0 Hz, 0.63*2H), 6.96 (m, 3H), 6.83 (d, *J* = 6.9 Hz, 0.37*2H), 5.20 (d, *J* = 6.6 Hz, 0.63*1H), 5.16 (d, *J* = 7.1 Hz, 0.37*1H), 3.89 (s, 3H), 3.83 (d, *J* = 5.9 Hz, 0.63*1H), 3.57 (d, *J* = 6.9 Hz, 0.37*1H), 3.35-3.26 (m, 0.37*1H), 3.20 (dd, *J* = 13.6, 6.7 Hz, 0.63*1H), 2.45 (d, *J* = 7.0 Hz, 0.63*2H), 2.39 (d, *J* = 6.9 Hz, 0.37*2H), 1.92-1.75 (m, 1H), 1.52 (d, *J* = 6.8 Hz, 0.37*3H), 1.10 (d, *J* = 6.7 Hz, 0.63*3H), 0.89 (m, 6H). **^{13}C NMR (151 MHz, $CDCl_3$)** δ 199.8, 199.1, 164.1, 164.0, 141.2, 140.1, 140.0, 137.0, 130.1, 130.8, 129.2, 128.6, 127.9, 127.4, 126.7, 114.1, 114.0, 55.5, 45.0, 44.2, 43.5, 30.2, 30.1, 22.3, 18.0, 13.5. **IR (neat):** 3460, 1668 cm^{-1}. **HRMS (ESI)** calcd for $C_{21}H_{26}O_3$ $[M+H]^+$: 327.1955, found: 327.1960.

(2R,3S)-2-hydroxy-3-(6-methoxynaphthalen-2-yl)-1-(4-methoxyphenyl)butan-1-one (8b)

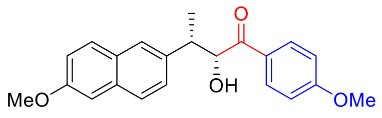

8b

The general procedure A was followed by using (S)-2-(6-methoxynaphthalen-2-yl)propanal (42.8 mg, 0.20 mmol, 1 equiv), tertbutyl isocyanide (34 μl, 0.3 mmol, 1.5 equiv) and (4-methoxyphenyl)boronic acid (55.0 mg, 0.36 mmol, 1.8 equiv) to afford 38.0 mg (54%) of the product **8b** as a white solid. mp: 109-111 °C. TLC (petroleum ether: ethyl acetate, 84:16 v/v): R_f = 0.30, the ratio of eluents (petroleum ether: ethyl acetate, v/v): 93:7. $[\alpha]^{25}_{D}$ -248.0 (*c* 0.3, $CHCl_3$). **^{1}H NMR (600 MHz, $CDCl_3$)** δ 7.98 (d, *J* = 8.8 Hz, 2H), 7.73 (d, *J* = 8.0 Hz, 3H), 7.54 (dd, *J* = 8.5, 1.1 Hz, 1H), 7.18-7.10 (m, 2H), 7.00 (d, *J* = 8.8 Hz, 2H), 5.30 (dd, *J* = 6.6, 2.2 Hz, 1H), 3.92 (s, 3H), 3.90 (s, 3H), 3.45-3.27 (m, 1H), 1.17 (d, *J* = 7.1 Hz, 3H). **^{13}C NMR (151 MHz, $CDCl_3$)** δ 199.6, 164.2, 157.4, 139.3, 133.5, 131.0, 129.2, 129.0, 127.0, 126.8, 126.5, 125.8, 118.8, 114.2, 105.5, 76.7, 55.6, 55.3, 43.7, 13.5. **IR (neat):** 3490, 1662 cm^{-1}. **HRMS (ESI)** calcd for $C_{22}H_{22}O_4S$ $[M+H]^+$: 351.1591, found: 351.1596. Note: Another diastereoisome **8b'** was obtained 15.0 mg (21%) as colorless oil without clean NMR.

3-(3-benzoylphenyl)-2-hydroxy-1-(4-methoxyphenyl)butan-1-one (8c) dr = 2.10:1

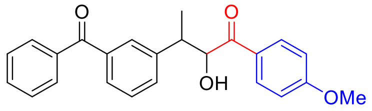

8c

The general procedure A was followed by using 2-(3-benzoylphenyl)propanal (47.6 mg, 0.20 mmol, 1 equiv), tertbutyl isocyanide (34 μl, 0.3 mmol, 1.5 equiv) and (4-methoxyphenyl)boronic acid (55.0 mg, 0.36 mmol, 1.8 equiv) to afford 56.1 mg (75%) of the product **8c** as a colorless oil (dr = 2.1:1). TLC (petroleum ether: ethyl acetate, 84:16 v/v): R_f = 0.20, 0.18, the ratio of eluents (petroleum ether: ethyl acetate, v/v): 85:15. **^{1}H NMR (400 MHz, $CDCl_3$)** δ 7.88 (d, *J* = 8.0 Hz, 0.67*2H), 7.80-7.71 (m, 2.66H), 7.66-7.61 (m, 1H), 7.59-7.48 (m, 2H), 7.45-7.35 (m, 3H), 7.23-7.14 (m, 1H), 6.96-6.85(m, 2H), 5.15 (t, *J* = 7.4 Hz, 1H), 3.85 (d, *J* = 6.4 Hz, 0.67*1H), 3.82 (s, 3H), 3.60 (d, *J* = 6.4 Hz, 0.33*1H), 3.36-3.30 (m, 0.33*1H), 3.26 (dd, *J* = 14.3, 7.6 Hz, 0.67*1H), 1.49 (d, *J* = 6.8 Hz, 0.33*1H), 1.06 (d, *J* = 6.8 Hz, 0.67*2H).**^{13}C NMR (101 MHz, $CDCl_3$)** δ 199.2, 198.8, 196.7, 196.5, 164.2, 164.1, 144.3, 140.2, 137.6, 137.5, 137.1, 132.4, 132.3, 132.2, 131.9, 130.9, 130.7, 130.0, 129.9, 129.8, 129.3, 128.7, 128.6, 128.2, 128.2, 128.1, 127.8, 126.9, 126.4, 114.2, 114.1, 76.6, 76.2, 55.5, 55.5, 44.4, 43.7, 18.2, 13.3. **IR (neat):** 3455, 1655 cm^{-1}. **HRMS (ESI)** calcd for $C_{24}H_{22}O_4$ $[M+H]^+$: 375.1591, found: 375.1595.

6-(2,4-dimethylphenoxy)-2-hydroxy-1-(4-methoxyphenyl)-3,3-dimethylhexan-1-one (8d)

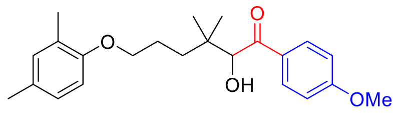

8d

The general procedure A was followed by using 5-(2,4-dimethylphenoxy)-2,2-dimethylpentanal (46.8 mg, 0.20 mmol, 1 equiv), tertbutyl isocyanide (34 μl, 0.3 mmol, 1.5 equiv) and (4-methoxyphenyl)boronic acid (55.0 mg, 0.36 mmol, 1.8 equiv) to

afford 28.1 mg (38%) of the product **8d** as a colorless oil. TLC (petroleum ether: ethyl acetate, 84:16 v/v): R_f = 0.40, the ratio of eluents (petroleum ether: ethyl acetate, v/v): 95:5. **^{1}H NMR (400 MHz, $CDCl_3$)** δ 7.87 (d, *J* = 7.5 Hz, 2H), 7.00 (d, *J* = 7.4 Hz, 1H), 6.95 (d, *J* = 7.5 Hz, 2H), 6.65 (d, *J* = 7.4 Hz, 1H), 6.60 (s, 1H), 4.89 (d, *J* = 8.1 Hz, 1H), 3.98-3.79 (m, 5H), 3.52 (d, *J* = 8.0 Hz, 1H), 2.31 (s, 3H), 2.15 (s, 3H), 1.92-1.70 (m, 2H), 1.67-1.60 (m, 1H), 1.42-1.33 (m, 1H), 0.86 (s, 6H). **^{13}C NMR (101 MHz, $CDCl_3$)** δ 202.2, 164.0, 157.0, 136.4, 130.9, 130.2, 130.2, 123.6, 120.6, 113.9, 112.1, 77.5, 68.4, 55.5, 38.6, 35.6, 24.4, 24.2, 23.1, 21.4, 15.8. **IR (neat):** 3476, 1663 cm^{-1}. **HRMS (ESI)** calcd for $C_{23}H_{30}O_4$ $[M+H]^+$: 371.2217, found: 371.2219.

3-(1-(4-chlorobenzoyl)-5-methoxy-2-methyl-1H-indol-3-yl)-2-hydroxy-1-(4-methoxyphenyl)propan-1-one (8e)

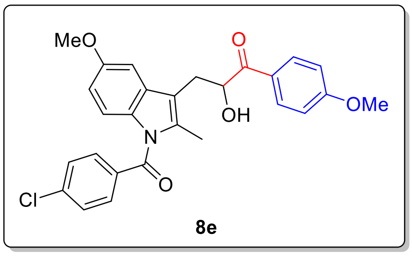

The general procedure A was followed by using 2-(1-(4-chlorobenzoyl)-5-methoxy-2-methyl-1H-indol-3-yl)acetaldehyde (27.0 mg, 0.20 mmol, 1 equiv), tertbutyl isocyanide (34 µl, 0.3 mmol, 1.5 equiv) and (4-methoxyphenyl)boronic acid (55.0 mg, 0.36 mmol, 1.8 equiv) to afford 50.5 mg (53%) of the product **8e** as a red oil. TLC (petroleum ether: ethyl acetate, 80:20 v/v): R_f = 0.15, the ratio of eluents (petroleum ether: ethyl acetate, v/v): 75:25. **^{1}H NMR (400 MHz, $CDCl_3$)** δ 7.83 (d, *J* = 8.0 Hz, 2H), 7.52 (d, *J* = 7.9 Hz, 2H), 7.43 (d, *J* = 7.7 Hz, 2H), 6.99-6.76 (m, 4H), 6.65 (d, *J* = 9.0 Hz, 1H), 5.35 (d, *J* = 5.8 Hz, 1H), 3.86 (s, 3H), 3.78 (s, 3H), 3.25-3.01 (m, 2H), 2.04 (s, 3H). **^{13}C NMR (101 MHz, $CDCl_3$)** δ 199.7, 168.1, 164.3, 155.9, 139.0, 135.7, 134.0, 131.0, 131.0, 130.9, 130.9, 129.0, 126.8, 114.8, 114.6, 113.9, 111.5, 72.2, 55.6, 55.6, 31.5, 13.5. **IR (neat):** 3474, 1672 cm^{-1}. **HRMS (ESI)** calcd for $C_{27}H_{24}ClNO_5$ $[M+H]^+$: 478.1416, found: 478.1422.

(1R,2S,5R)-2-isopropyl-5-methylcyclohexyl 4-(1-hydroxy-2-(4-methoxyphenyl)-2-oxoethyl)benzoate (8f) dr = 1:1

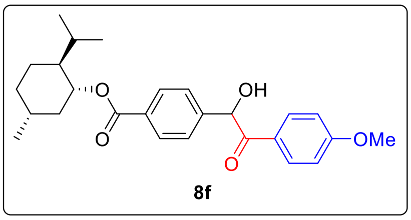

The general procedure B was followed by using (1R,2S,5R)-2-isopropyl-5-methylcyclohexyl 4-formylbenzoate (58.0 mg, 0.20 mmol, 1 equiv), cyclohexyl isocyanide (37 µl, 0.3 mmol, 1.5 equiv) and (4-methoxyphenyl)boronic acid (55.0 mg, 0.36 mmol, 1.8 equiv) to afford 70.0 mg (82%) of the product **8f** as a colorless oil. TLC (petroleum ether: ethyl acetate, 84:16 v/v): R_f = 0.25, the ratio of eluents (petroleum ether: ethyl acetate, v/v): 90:10. **HPLC analysis:** HPLC DAICEL CHIRALCEL IA-3, hexane/isopropanol = 85/15, 1mL/min, λ = 254 nm, tR = 17.9 min, tR = 22.2 min, dr = 1:1. **^{1}H NMR (400 MHz, $CDCl_3$)** δ 7.99 (d, *J* = 7.5 Hz, 2H), 7.89 (d, *J* = 7.9 Hz, 2H), 7.41 (d, *J* = 7.6 Hz, 2H), 6.87 (d, *J* = 8.0 Hz, 2H), 5.94 (s, 1H), 4.89 (td, *J* = 10.7, 3.9 Hz, 1H), 4.69

(s, 1H), 3.82 (s, 3H), 2.07 (d, *J* = 10.3 Hz, 1H), 1.95-1.83 (m, 1H), 1.71 (d, *J* = 11.1 Hz, 2H), 1.59-1.45 (m, 2H), 1.19-0.99 (m, 2H), 0.98-0.82 (m, 7H), 0.75 (d, *J* = 6.8 Hz, 3H). **^{13}C NMR (101 MHz, $CDCl_3$)** δ 196.6, 165.5, 164.2, 144.1, 131.5, 130.8, 130.3, 127.6, 125.9, 114.0, 75.3, 74.9, 55.5, 47.2, 40.9, 34.2, 31.4, 26.4, 26.4, 23.5, 21.9, 20.7, 16.4, 16.4. **IR (neat):** 3456, 1708, 1670 cm^{-1}. **HRMS (ESI)** calcd for $C_{26}H_{33}O_5$ $[M+H]^+$: 425.2323, found: 425.2327.

(1S,2R,4S)-1,7,7-trimethylbicyclo[2.2.1]heptan-2-yl
4-(1-hydroxy-2-(4-methoxyphenyl)-2-oxoethyl)benzoate (8g) dr = 1:1

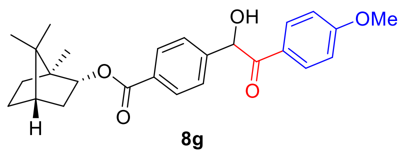

8g

The general procedure B was followed by using (1S,2R,4S)-1,7,7-trimethylbicyclo[2.2.1]heptan-2-yl 4-formylbenzoate (57.0 mg, 0.20 mmol, 1 equiv), cyclohexyl isocyanide (37 µl, 0.3 mmol, 1.5 equiv) and (4-methoxyphenyl)boronic acid (55.0 mg, 0.36 mmol, 1.8 equiv) to afford 47.0 mg (56%) of the product **8g** as a colorless oil. TLC (petroleum ether: ethyl acetate, 84:16 v/v): R_f = 0.25, the ratio of eluents (petroleum ether: ethyl acetate, v/v): 90:10. **HPLC analysis:** HPLC DAICEL CHIRALCEL IA-3, hexane/isopropanol = 85/15, 1mL/min, λ = 254 nm, tR = 18.5 min, tR = 22.1 min, dr = 1:1. **^{1}H NMR (400 MHz, $CDCl_3$)** δ 8.00 (d, *J* = 7.8 Hz, 2H), 7.89 (d, *J* = 8.4 Hz, 2H), 7.42 (d, *J* = 7.8 Hz, 2H), 6.87 (d, *J* = 8.4 Hz, 2H), 5.95 (d, *J* = 5.5 Hz, 1H), 5.07 (d, *J* = 9.6 Hz, 1H), 4.71 (d, *J* = 5.7 Hz, 1H), 3.82 (s, 3H), 2.55-2.36 (m, 1H), 2.15-2.03 (m, 1H), 1.85-1.71 (m, 2H), 1.45-1.33 (m, 1H), 1.30-1.23 (m, 1H), 1.11-1.02 (m, 1H), 0.96-0.85 (m, 9H). **^{13}C NMR (101 MHz, $CDCl_3$)** δ 196.5, 166.2, 164.2, 144.1, 131.5, 130.8, 130.2, 127.6, 125.9, 114.0, 80.6, 75.3, 55.5, 49.0, 47.8, 44.9, 36.8, 28.0, 27.3, 19.7, 18.8, 13.5. **IR (neat):** 3464, 1709, 1670 cm^{-1}. **HRMS (ESI)** calcd for $C_{26}H_{31}O_5$ $[M+H]^+$: 423.2166, found: 423.2163.

(3R,8R,9R,10R,13S,14R,17S)-10,13-dimethyl-17-((S)-5-methylhexan-2-yl)-2,3,4,5,8,9,10,11,12,13,14,15,16,17-tetradecahydro-1H-cyclopenta[a]phenanthren-3-yl
4-(1-hydroxy-2-(4-methoxyphenyl)-2-oxoethyl)benzoate (8h) dr = 1:1

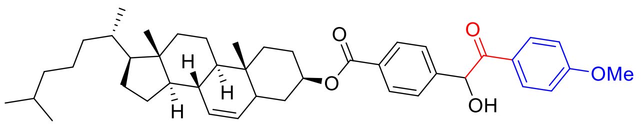

8h

The general procedure B was followed by using (3R,8R,9R,10R,13S,14R,17S)-10,13-dimethyl-17-((S)-6-methylheptan-2-yl)-2,3,4,5,8,9,10,11,12,13,14,15,16,17-tetradecahydro-1H-cyclopenta[a]phenanthren-3-yl 4-formylbenzoate (104.0 mg, 0.20 mmol, 1 equiv), cyclohexyl isocyanide (37 µl, 0.3 mmol, 1.5 equiv) and (4-methoxyphenyl)boronic acid (55.0 mg, 0.36 mmol, 1.8 equiv) to afford 52.0 mg (40%) of the product **8h** as a white solid. mp: 130-132 °C. TLC (petroleum ether: ethyl acetate, 84:16 v/v): R_f = 0.25, the ratio of eluents (petroleum ether: ethyl acetate, v/v): 90:10. **HPLC analysis:** HPLC DAICEL CHIRALCEL IA-3, hexane/isopropanol = 85/15, 1mL/min, λ = 254 nm, tR = 15.7 min, tR = 16.3 min, dr = 1:1. **^{1}H NMR (400 MHz, $CDCl_3$)** δ 7.99 (d, *J* = 6.6 Hz, 2H), 7.88 (d, *J* = 7.6 Hz, 2H), 7.40 (d, *J* = 6.8 Hz, 2H), 6.86 (d, *J* = 7.6 Hz,

2H), 5.93 (s, 1H), 5.40 (s, 1H), 4.81 (s, 1H), 4.70 (s, 1H), 3.82 (s, 3H), 2.42 (s, 2H), 2.04-1.83 (m, 5H), 1.66-1.45 (m, 7H), 1.31-1.01 (m, 15H), 0.97-0.81 (m, 11H), 0.68 (s, 3H). **^{13} NMR (151 MHz, $CDCl_3$)** δ 196.6, 165.4, 164.2, 144.1, 139.5, 131.5, 130.8, 130.3, 127.6, 125.9, 122.8, 114.0, 75.3, 74.7, 56.7, 56.1, 55.5, 50.0, 42.3, 39.7, 39.5, 38.1, 36.9, 36.6, 36.2, 35.8, 31.9, 31.8, 28.2, 28.0, 27.8, 24.3, 23.8, 22.8, 22.5, 21.0, 19.3, 18.7, 11.8. **IR (neat):** 3410, 1712, 1669 cm^{-1}. **HRMS (ESI)** calcd for $C_{43}H_{59}O_5$ $[M+H]^+$: 655.4357, found: 655.4366.

(5S,8R,9S,10S,13S,14S)-3-(4-(2-hydroxy-4-phenylbutanoyl)phenoxy)-10,13-dimethylhexadecahydro-17H-cyclopenta[a]phenanthren-17-one (8i) dr = 1:1

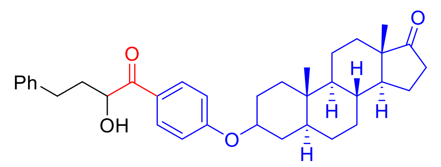

8i

The general procedure A was followed by using 3-phenylpropanal (27.0 mg, 0.20 mmol, 1 equiv), tertbutyl isocyanide (34 μl, 0.3 mmol, 1.5 equiv) and (4-(((5S,8R,9S,10S,13S,14S)-10,13-dimethyl-17-oxohexadecahydro-1H-cyclopenta[a]phenanthren-3-yl)oxy)phenyl)boronic acid (148.0 mg, 0.36 mmol, 1.8 equiv) to afford 57 mg (54%) of the product **8i** as a colorless oil. TLC (petroleum ether: ethyl acetate, 84:16 v/v): R_f = 0.20, the ratio of eluents (petroleum ether: ethyl acetate, v/v): 85:15. **HPLC analysis:** HPLC DAICEL CHIRALCEL IA-3, hexane/isopropanol = 85/15, 1mL/min, λ = 254 nm, tR = 24.8 min, tR = 41.7 min, dr = 1:1. **^{1}H NMR (400 MHz, $CDCl_3$)** δ 7.74 (d, J = 8.1 Hz, 2H), 7.29-7.27 (m, 1H), 7.22-7.13 (m, 4H), 6.89 (d, J = 7.8 Hz, 2H), 4.97 (t, J = 7.3 Hz, 1H), 4.64 (s, 1H), 3.83 (d, J = 6.3 Hz, 1H), 2.88-2.75 (m, 2H), 2.44 (dd, J = 19.2, 8.8 Hz, 1H), 2.21-1.99 (m, 2H), 1.97-1.87 (m, 2H), 1.84-1.78 (m, 2H), 1.75-1.45 (m, 9H), 1.41-1.21 (m, 7H), 1.11-0.98 (m, 1H), 0.86 (s, 6H). **^{13}C NMR (151 MHz, $CDCl_3$)** δ 221.4, 200.0, 162.5, 141.2, 130.9, 128.6, 128.4, 126.0, 125.5, 115.4, 72.2, 71.6, 54.2, 51.4, 47.8, 39.6, 38.1, 35.9, 35.8, 35.0, 32.5, 32.4, 31.5, 31.3, 30.7, 28.0, 25.5,21.7, 20.0, 13.8, 11.4. **IR (neat):** 3469, 1735, 1668 cm^{-1}. **HRMS (ESI)** calcd for $C_{23}H_{25}NO_4[M+H]^+$:380.1856, found:380.1857.

Ethyl 2-(4-(2-hydroxy-4-phenylbutanoyl)phenoxy)-2-methylpropanoate (8j)

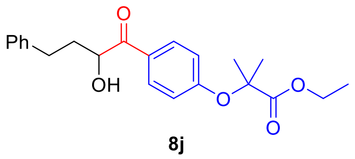

8j

The general procedure A was followed by using 3-phenylpropanal (27.0 mg, 0.20 mmol, 1 equiv), tertbutyl isocyanide (34 μl, 0.3 mmol, 1.5 equiv) and (4-((1-ethoxy-2-methyl-1-oxopropan-2-yl)oxy)phenyl)boronic acid (91.0 mg, 0.36 mmol, 1.8 equiv) to afford 43.7 mg (81%) of the product **8j** as a colorless oil. TLC (petroleum ether: ethyl acetate, 84:16 v/v): R_f = 0.30, the ratio of eluents (petroleum ether: ethyl acetate, v/v): 90:10. **^{1}H NMR (400 MHz, $CDCl_3$)** δ 7.70 (d, J = 8.6 Hz, 2H), 7.30-7.25 (m, 3H), 7.22-7.14 (m, 3H), 6.80 (d, J = 8.6 Hz, 2H), 4.96 (d, J = 7.0 Hz, 1H), 4.23 (q, J = 7.0 Hz, 2H), 3.80 (s, 1H), 2.94-2.71 (m, 2H), 2.22-2.06 (m, 1H), 1.81 (td, J = 13.8, 8.5 Hz, 1H), 1.66 (s, 6H), 1.21 (t, J = 7.1 Hz, 3H). **^{13}C NMR (101 MHz, $CDCl_3$)** δ 200.2, 173.4, 160.4, 141.1, 130.4, 128.6, 128.4, 126.6, 126.0, 117.4, 79.4, 71.8, 61.7, 38.0, 31.3,

25.4, 25.3, 14.0. **IR (neat):** 3478, 1733, 1672 cm^{-1}. **HRMS (ESI)** calcd for $C_{22}H_{26}O_5$ $[M+H]^+$: 371.1853, found:371.1854.

4 Gram-scale synthesis of 5e and 5f

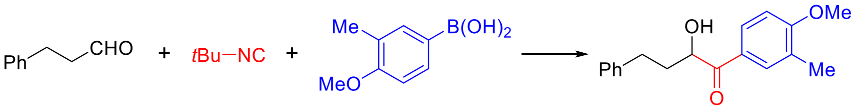

1a 10 mmol; **2a** 15 mmol; **3e** 18 mmol; **5e** 89% yield, **2.53 g**

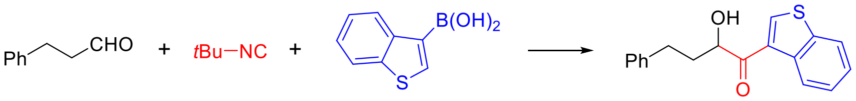

1a 10 mmol; **2a** 15 mmol; **3r** 18 mmol; **5r** 63% yield, **1.86 g**

In air, a 100 mL schlenk tube was charged with arylboronic acids (18 mmol, 1.8 equiv). The tube was evacuated and filled with argon for three cycles. Then, chloroform (35 mL), pH = 8 buffer (15 mL), 3-phenylpropanal (1.35 g, 10 mmol, 1 equiv), tertbutyl isocyanide (1.7 ml, 15 mmol, 1.5 equiv) were added under argon. The reaction was allowed to stir at corresponding temperature for 24 hours. Upon completion of the reaction, diluted with dichloromethane (20 mL), and washed with brine (30 mL). The organic layer was separated and dried over Na_2SO_4. After removal of the solvent, the crude reaction mixture was purified on silica gel (petroleum ether: ethyl acetate 90:10, v/v) to afford the desired products **5e** (2.53 g, 89%) and **5r** (1.86g, 63%).

5 Transformations of α-hydroxyketone

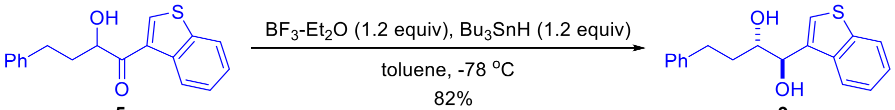

5r **9**

To a solution of α-hydroxyketone **5r** (178 mg, 0.60 mmol, 1 equiv) in toluene (6 mL) was added $BF_3 \cdot Et_2O$ (92ul, 0.72 mmol, 1.2 equiv) dropwise at -78 ℃ under argon and the reaction was stirred for 10 min at the same temperature. Then, Bu_3SnH (210 mg, 0.72 mmol, 1.2 equiv) was added dropwise and the reaction mixture was stirred for 2 h at -78 ℃, and poured into the cooled saturated $NaHCO_3$ (5 mL, 0 °C). The mixture was stirred for several hours and extracted with ethyl acetate (20 mL). The combined organic extracts were dried over Na_2SO_4 and K_2CO_3. After removal of the solvent, the crude reaction mixture was purified on silica gel to afford the desired product ±**9** (148mg, 82%) as a white solid.[3]

1-(benzo[b]thiophen-3-yl)-4-phenylbutane-1,2-diol (9)

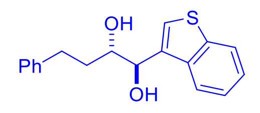

9

mp: 103-105 °C. TLC (petroleum ether: ethyl acetate, 80:20 v/v): R_f = 0.10, the ratio of eluents (petroleum ether: ethyl acetate, v/v): 70:30. **^{1}H NMR (400 MHz, $CDCl_3$)** δ 7.95-7.76 (m, 2H), 7.52 (d, J = 6.8 Hz, 1H), 7.44-7.32 (m, 2H), 7.30-7.20

(m, 3H), 7.19-7.08 (m, 3H), 5.13 (s, 1H), 4.07 (s, 1H), 2.93-2.74 (m, 1H), 2.69-2.55 (m, 1H), 2.37 (s, 1H), 2.02 (s, 1H), 1.93-1.69 (m, 2H). **^{13}C NMR (101 MHz, $CDCl_3$)** δ 141.7, 140.7 137.4, 135.7, 128.4, 128.3, 125.8, 124.5, 124.2, 124.0, 122.9, 122.0, 73.5, 72.8, 33.1, 32.0. **IR (neat):** 3442 cm^{-1}. **HRMS (ESI)** calcd for $C_{18}H_{18}O_2S$ $[M+H]^+$: 299.1100, found: 299.1094.

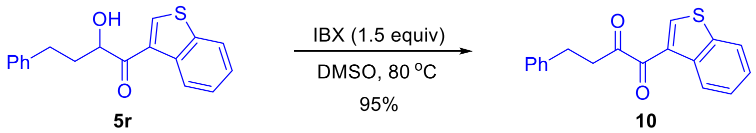

To a solution of α-hydroxy ketone **5r** (296 mg, 1.0 mmol, 1 equiv) in DMSO (2 mL) was added IBX (420 mg, 1.5 mmol, 1.5 equiv). Then the reaction was stirred for 4 hours at 80 ℃. The solution was then diluted with water and extracted with Et_2O (20 mL) , the organic layers were then pooled and washed with saturated aqueous $NaHCO_3$, brine, dried over Na_2SO_4. After removal of the solvent, the crude reaction mixture was purified on silica gel to afford the desired product **10** (280 mg, 95%) as a yellow oil.[4]

1-(benzo[b]thiophen-3-yl)-4-phenylbutane-1,2-dione (10)

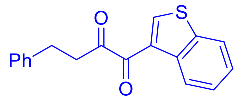

10

TLC (petroleum ether: ethyl acetate, 84:16 v/v): R_f = 0.70, the ratio of eluents (petroleum ether: ethyl acetate, v/v): 95:5. **^{1}H NMR (400 MHz, $CDCl_3$)** δ 8.78 (d, *J* = 7.5 Hz, 1H), 8.70 (s, 1H), 7.89 (d, *J* = 7.4 Hz, 1H), 7.61-7.40 (m, 2H), 7.40-7.11 (m, 5H), 3.33 (t, *J* = 6.6 Hz, 2H), 3.08 (t, *J* = 6.4 Hz, 2H). **^{13}C NMR (101 MHz, $CDCl_3$)** δ 201.2, 184.4, 143.6, 140.3, 139.2, 136.8, 129.1, 128.5, 128.3, 126.3, 126.2, 125.8, 125.2, 122.3, 39.4, 29.1. **IR (neat):** 1715, 1651 cm^{-1}. **HRMS (ESI)** calcd for $C_{18}H_{14}O_2S[M+H]^+$: 295.0787, found: 295.0789.

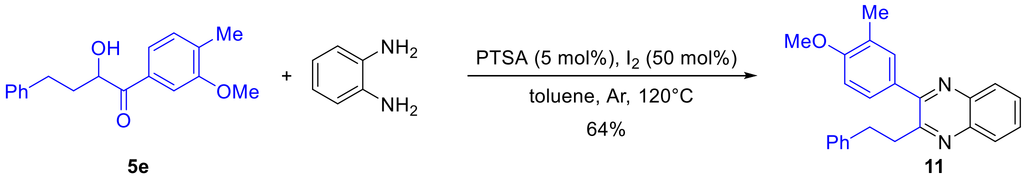

To a 25 mL sealed tube was charged with α-hydroxyketone **5e** (142 mg, 0.50 mmol, 1 equiv), 1,2-diaminobenzene (82 mg, 0.75 mmol, 1.5 equiv), PTSA (5 mg, 0.025 mmol, 0.05 equiv), iodine (64 mg, 0.25 mmol, 0.5 equiv), 4 Å molecular sieve (125 mg) and toluene (5 mL). The reaction was stirred at 120 °C for 14 hours. Then, 20 mL saturated sodium sulfite solution was added and the mixture was extracted with ethyl acetate (20 mL). The combined organic extracts were dried over Na_2SO_4. After removal of the solvent, the crude reaction mixture was purified on silica gel to afford the desired product **9** (112 mg, 63%) as a yellow solid.[5]

2-(4-methoxy-3-methylphenyl)-3-phenethylquinoxaline (11)

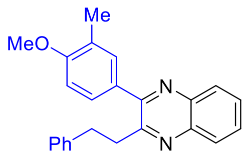

11

mp: 104-106 °C. TLC (petroleum ether: ethyl acetate, 84:16 v/v): R_f = 0.60, the ratio of eluents (petroleum ether: ethyl acetate, v/v): 95:5. **^{1}H NMR (400 MHz,**

CDCl$_3$) δ 8.12 (d, *J* = 6.9 Hz, 2H), 7.80-7.67 (m, 2H), 7.43-7.30 (m, 2H), 7.28-7.15 (m, 3H), 7.11 (d, *J* = 7.1 Hz, 2H), 6.93 (d, *J* = 8.3 Hz, 1H), 3.91 (s, 3H), 3.47-3.34 (m, 2H), 3.19-3.06 (m, 2H), 2.30 (s, 3H). **^{13}C NMR (101 MHz, CDCl$_3$)** δ 158.4, 155.3, 155.0, 141.4, 141.2, 140.8, 131.2, 130.8, 129.3, 129.2, 129.1, 128.5, 128.3, 127.6, 127.0, 125.9, 109.7, 55.5, 38.0, 34.8, 16.3. **HRMS (ESI)** calcd for $C_{24}H_{23}N_2O$ [M+H]$^+$: 355.1805, found: 355.1804.

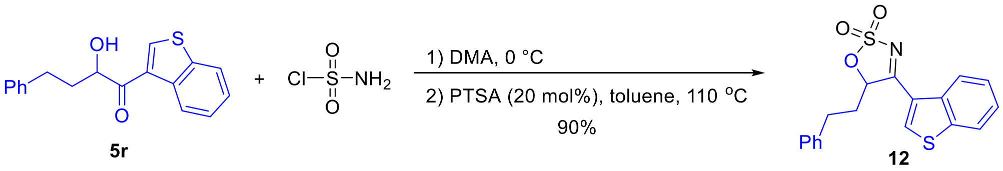

To a solution of α-hydroxyketone **5r** (148 mg, 0.50 mmol, 1 equiv) in DMA (2 mL) was added sulfamoyl chloride (116 mg, 1.0 mmol, 2 equiv) at 0 ℃. The reaction mixture was stirred at room temperature for 2 hours, diluted with ethyl acetate (20 mL), and washed with brine (10 mL). The organic layer was dried over Na_2SO_4. After removal of the solvent, the residue was dissolved in toluene (2 mL), and PTSA (20 mg, 0.1 mmol, 0.2 equiv) was added. The mixture was stirred at 110 ℃ for 1 hour, cooled to room temperature, diluted with ethyl acetate (20 mL), and washed with brine (10 mL). The organic layer was separated and dried over Na_2SO_4. After removal of the solvent, the crude reaction mixture was purified on silica gel to afford the desired product **12** (161 mg, 90%) as a white solid.[6]

4-(benzo[b]thiophen-3-yl)-5-phenethyl-5H-1,2,3-oxathiazole 2,2-dioxide (12)

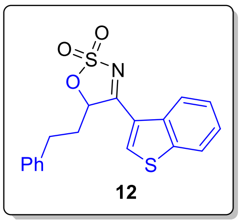

mp: 166-168 ^{o}C. TLC (petroleum ether: ethyl acetate, 84:16 v/v): R_f = 0.25, the ratio of eluents (petroleum ether: ethyl acetate, v/v): 90:10. **^{1}H NMR (400 MHz, CDCl$_3$)** δ 8.72 (d, *J* = 7.9 Hz, 1H), 7.91 (d, *J* = 7.6 Hz, 1H), 7.86 (s, 1H), 7.60-7.46 (m, 2H), 7.35-7.11 (m, 5H), 5.77 (s, 1H), 3.08-2.82 (m, 2H), 2.46-2.26 (m, 2H). **^{13}C NMR (101 MHz, CDCl$_3$)** δ 172.2, 139.6, 139.1, 139.0, 135.5, 128.9, 128.7, 126.9, 126.7, 126.6, 125.7, 124.5, 122.6, 86.7, 36.8, 31.3. **HRMS (ESI)** calcd for $C_{18}H_{16}NO_3S_2$ [M+H]$^+$: 358.0566, found: 358.0568.

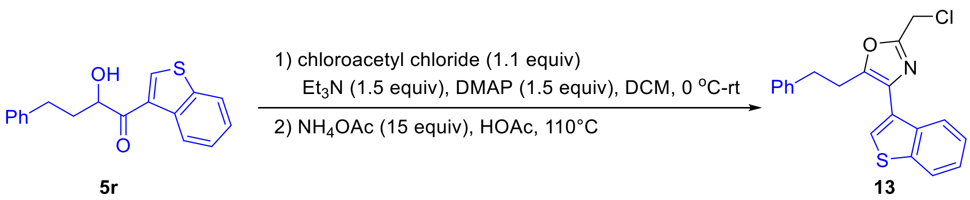

Step 1: In argon, to a solution of α-hydroxyketone **5r** (148 mg, 0.50 mmol, 1 equiv) in dichloromethane (3 mL) was added DMAP (62 mg, 0.50 mmol, 1 equiv) and triethylamine (70 ul, 0.50 mmol, 1.0 equiv). The mixture was cooled to 0 ^{o}C and chloroacetyl chloride (44 ul, 1.1 equiv) was added dropwise by syringe. The reaction mixture was stirred at room temperature for 16 hours. The reaction mixture was diluted with dichloromethane (20 mL), washed with water (10 mL), 5% aqueous HCl (10 mL), and 5% aqueous $NaHCO_3$ (10 mL). The

organic layers were separated, combined and dried over Na_2SO_4. After removal of the solvent, the crude reaction mixture was purified on silica gel (petroleum ether: ethyl acetate 90:10, v/v) to afford the chloroacetyl ester (130 mg, 70%) as a red oil.

Step 2: In argon, to a solution of chloroacetyl ester (130 mg, 0.35 mmol, 1.0 equiv) in glacial acetic acid (8 mL) was added ammonium acetate (404 mg, 5.25 mmol, 15 equiv). The reaction was stirred at 110 ℃ for 3 hours. Upon completion of the reaction, the reaction was poured into cold water (10 mL), neutralized with saturated aqueous $NaHCO_3$(10 mL) and extracted with dichloromethane (20 mL). The organic layers were separated, combined and dried over Na_2SO_4. After removal of the solvent, the crude reaction mixture was purified on silica gel to afford the product **13** (56 mg, 45%) as a red oil.[7]

4-(benzo[b]thiophen-3-yl)-2-(chloromethyl)-5-phenethyloxazole (13)

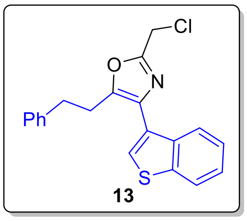

TLC (petroleum ether: ethyl acetate, 84:16 v/v): R_f = 0.80, the ratio of eluents (petroleum ether: ethyl acetate, v/v): 97:3. **^{1}H NMR (400 MHz, $CDCl_3$)** δ 8.02-7.93 (m, 1H), 7.89-7.82 (m, 1H), 7.42-7.33 (m, 2H), 7.31-7.23 (m, 2H), 7.22-7.16 (m, 1H), 7.11 (d, *J* = 7.2 Hz, 2H), 6.97 (s, 1H), 4.70 (s, 2H), 3.16-2.96 (m, 4H). **^{13}C NMR (101 MHz, $CDCl_3$)** δ 157.3, 149.8, 140.1, 140.0, 138.0, 131.9, 128.6, 128.4, 126.8, 126.5, 124.7, 124.6, 124.4, 123.6, 122.5, 36.1, 34.2, 27.7. **HRMS (ESI)** calcd for $C_{18}H_{20}O_4$ $[M+H]^+$: 354.0714, found: 354.0721.

6 Synthetic applications

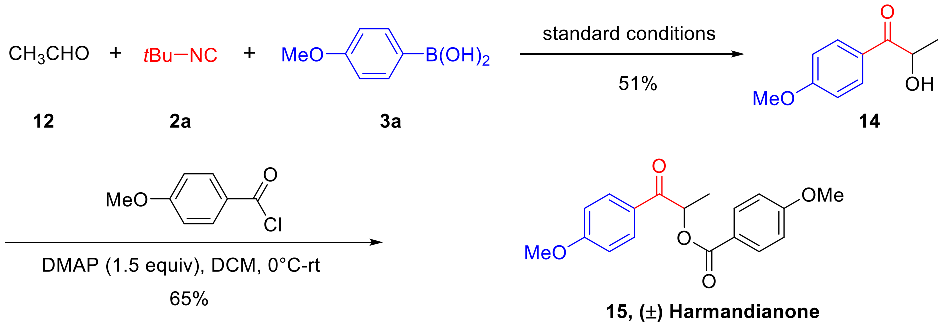

Step 1: The general procedure A was followed by using acetaldehyde (40% in water, 110 mg, 1.0 mmol, 1 equiv), tertbutyl isocyanide (170 μl,1.5 mmol, 1.5 equiv) and (4-methoxyphenyl)boronic acid (275 mg, 1.8 mmol, 1.8 equiv) to afford 93 mg (51%) of the α-hydroxyketone **14** as a colorless oil.

Step 2: To a solution of α-hydroxyketone **14** (58 mg, 0.32 mmol, 1.0 equiv) in dichloromethane (5 mL) was added DMAP (59 mg, 0.18 mmol, 1.5 equiv). The miture was cooled to 0 °C and 4-methoxybenzoyl chloride (57 ul, 0.42 mmol, 1.1 equiv) was added. The reaction mixture was stirred at room temperature for overnight. Upon completion of the reaction, diluted with dichloromethane (20 mL), and washed with brine (10 mL). The organic layer was separated and dried over Na_2SO_4. After removal of the solvent, the crude reaction mixture was purified on silica gel to afford the desired product **15** (65 mg, 65%) as a white solid.

2-hydroxy-1-(4-methoxyphenyl)propan-1-one (14)[8]

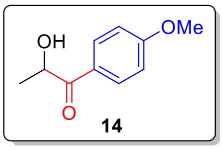

TLC (petroleum ether: ethyl acetate, 84:16 v/v): R_f = 0.25, the ratio of eluents (petroleum ether: ethyl acetate, v/v): 90:10. **^{1}H NMR (400 MHz, $CDCl_3$)** δ 7.92 (d, *J* = 7.8 Hz, 2H), 6.97 (d, *J* = 7.8 Hz, 2H), 5.17-5.02 (m, 1H), 3.89 (s, 3H), 3.84 (d, *J* = 6.3 Hz, 1H), 1.44 (d, *J* = 6.9 Hz, 3H). **^{13}C NMR (101 MHz, $CDCl_3$)** δ 200.7, 164.2, 131.0, 126.1, 114.1, 68.9, 55.5, 22.6. **IR (neat):** 3419, 1670 cm^{-1}.

1-(4-methoxyphenyl)-1-oxopropan-2-yl 4-methoxybenzoate (15)[9]

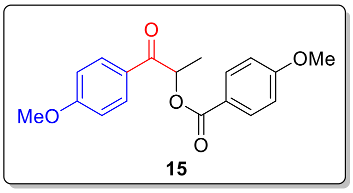

mp: 133-135 °C. TLC (petroleum ether: ethyl acetate, 84:16 v/v): R_f = 0.40, the ratio of eluents (petroleum ether: ethyl acetate, v/v): 93:7. **^{1}H NMR (400 MHz, $CDCl_3$)** δ 8.04 (d, *J* = 8.6 Hz, 2H), 7.99 (d, *J* = 8.7 Hz, 2H), 6.95 (d, *J* = 8.8 Hz, 2H), 6.92 (d, *J* = 8.7 Hz, 2H), 6.15 (q, *J* = 6.8 Hz, 1H), 3.87 (s, 3H), 3.86 (s, 3H), 1.64 (d, *J* = 6.9 Hz, 3H). **^{13}C NMR (101 MHz, $CDCl_3$)** δ 195.3, 165.6, 163.8, 163.6, 131.9, 130.1, 127.3, 122.0, 113.9, 113.6, 71.3, 55.4, 55.3, 17.3. **IR (neat):** 1719, 1688 cm^{-1}.

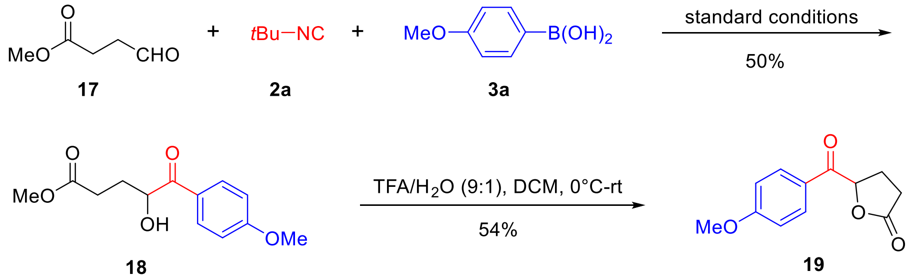

Step 1: The general procedure A was followed by using methyl 4-oxobutanoate (58 mg, 0.50 mmol, 1 equiv), tertbutyl isocyanide (85 μl, 0.75 mmol, 1.5 equiv) and (4-methoxyphenyl)boronic acid (137 mg, 0.90 mmol, 1.8 equiv) to afford 61 mg (50%) of the *α*-hydroxyketone **18** as a colorless oil.

Step 2: In argon, to a solution of *α*-hydroxyketone **18** (63 mg, 0.25 mmol, 1.0 equiv) in dichloromethane (2 mL) at 0 °C were added H_2O (1.2 mL) and TFA (110 ul). The miture was cooled to 0 °C and 4-methoxybenzoyl chloride (57 ul, 0.42 mmol, 1.1 equiv) was added. The reaction mixture was stirred at room temperature overnight. Upon completion, the reaction was diluted with dichloromethane (10 mL), and washed with saturated aqueous $NaHCO_3$ (5 mL). The organic layer was separated and dried over Na_2SO_4. After removal of the solvent, the crude reaction mixture was purified on silica gel to afford the desired product **19** (30 mg, 54%) as a white solid.

methyl 4-hydroxy-5-(4-methoxyphenyl)-5-oxopentanoate (18)

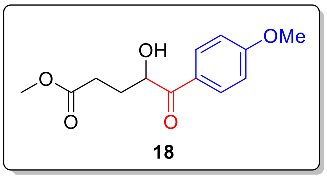

TLC (petroleum ether: ethyl acetate, 84:16 v/v): R_f = 0.10, the ratio of eluents (petroleum ether: ethyl acetate, v/v): 80:20. **^{1}H NMR (400 MHz, $CDCl_3$)** δ

8.02 (d, J = 7.9 Hz, 2H), 6.98 (d, J = 8.0 Hz, 2H), 5.08 (t, J = 7.8 Hz, 1H), 3.88 (s, 3H), 3.74 (d, J = 6.5 Hz, 1H), 3.69 (s, 3H), 2.77-2.61 (m, 1H), 2.45 (dt, J = 11.9, 5.7 Hz, 1H), 2.36-2.20 (m, 1H), 1.69-1.55 (m, 1H). **^{13}C NMR (101 MHz, CDCl$_3$)** δ 199.7, 173.8, 164.3, 131.2, 126.0, 114.1, 71.4, 55.5, 51.6, 31.3, 29.1. **IR (neat):** 3469, 1731, 1670 cm^{-1}. **HRMS (ESI)** calcd for $C_{13}H_{17}O_5$ [M+H]$^+$: 253.1071, found: 253.1069.

5-(4-methoxybenzoyl)dihydrofuran-2(3H)-one (19)[10]

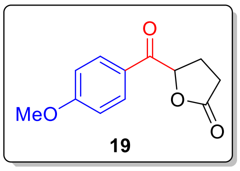

mp: 123-125 °C (lit.[10] 125-126 °C). TLC (petroleum ether: ethyl acetate, 84:16 v/v): R_f = 0.50, the ratio of eluents (petroleum ether: ethyl acetate, v/v): 95:5. **^{1}H NMR (400 MHz, CDCl$_3$)** δ 7.95 (d, J = 8.6 Hz, 2H), 6.97 (d, J = 8.6 Hz, 2H), 5.75 (t, J = 6.4 Hz, 1H), 3.88 (s, 3H), 2.68-2.35 (m, 4H). **^{13}C NMR (101 MHz, CDCl$_3$)** δ 192.7, 176.4, 164.4, 131.2, 126.6, 114.2, 78.1, 55.6, 26.9, 25.0. **IR (neat):**1770, 1682 cm^{-1}.

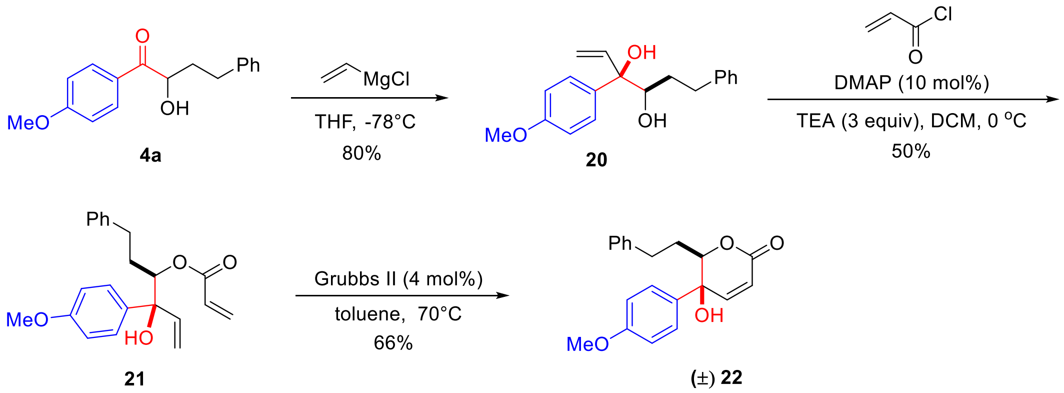

Step1: In argon, to a solution of the *α*-hydroxyketone **4a** (1.16 g, 4.3 mmol, 1 equiv) in dry THF (30 mL) was added a solution of vinylmagnesium chloride (9.5 mmol, 2.2 equiv) at -78 ℃. The mixture was stirred at same temperature until the starting material was fully consumed as indicated by TLC. Then, this reaction was quenched by saturated aqueous NH_4Cl (10 mL), extracted with Et_2O (30 mL) and dried over Na_2SO_4. After removal of the solvent, the crude reaction mixture was purified on silica gel (petroleum ether: ethyl acetate 70:30, v/v) to afford the diol **20** (30 mg, 80%) as a yellow oil.

Step 2: To a solution of the diol **20** (1.02 g, 3.50 mmol, 1 equiv) in DCM (40 mL) was added triethylamine (1.5 mL, 10.5 mmol, 3 equiv). The mixture was cooled to 0 ℃, and acryloyl chloride (0.39 mL, 4.80 mmol, 1.5 equiv) was added, followed by DMAP (33 mg, 0.30 mmol). After 2 hours the starting material was completely consumed and the mixture was quenched by water (20 mL). The organic layer was separated and dried over Na_2SO_4. After removal of the solvent, the crude reaction mixture was purified on silica gel (petroleum ether: ethyl acetate 80:20, v/v) to afford the acrylate **21** (616 mg, 50%) as a white solid.

Step 3: In argon, to a solution of the acrylate **21** (212 mg, 0.60 mmol, 1 equiv) in toluene (30 mL) was added Grubbs II catalyst (19 mg, 4 mol %). The reaction was stirred at 70 ℃ for 1 hour, then, was cooled to room

temperature. After removal of the solvent, the crude reaction mixture was purified on silica gel to afford desired product **22** (128 mg, 66%) as a white solid.[11]

5-hydroxy-5-(4-methoxyphenyl)-6-phenethyl-5,6-dihydro-2H-pyran-2-one (22)

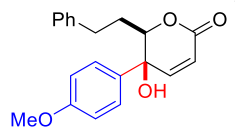

22

mp: 124-126 ^{o}C. TLC (petroleum ether: ethyl acetate, 80:20 v/v): R_f = 0.10, the ratio of eluents (petroleum ether: ethyl acetate, v/v): 75:25. **^{1}H NMR (400 MHz, CDCl$_3$)** δ 7.25 (d, *J* = 7.4 Hz, 2H), 7.22-7.09 (m, 3H), 7.00 (d, *J* = 7.2 Hz, 2H), 6.89 (d, *J* = 7.8 Hz, 2H), 6.83 (d, *J* = 9.7 Hz, 1H), 6.16 (d, *J* = 9.6 Hz, 1H), 4.38 (d, *J* = 10.1 Hz, 1H), 3.82 (s, 3H), 2.99-2.83 (m, 2H), 2.63-2.46 (m, 1H), 2.28-2.08 (m, 1H), 1.80-1.60 (m, 1H). **^{13}C NMR (101 MHz, CDCl$_3$)** δ 164.0, 159.4, 150.4, 140.8, 132.3, 128.4, 128.3, 126.7, 125.9, 121.3, 114.0, 84.8, 70.0, 55.3, 31.0, 28.8. **IR (neat):** 3368, 1701 cm^{-1}. **HRMS (ESI)** calcd for $C_{20}H_{21}O_4$ $[M+H]^+$: 325.1434, found: 325.1432.

7 NMR spectra

^{1}H NMR (400 MHz, $CDCl_3$) spectrum of 4a

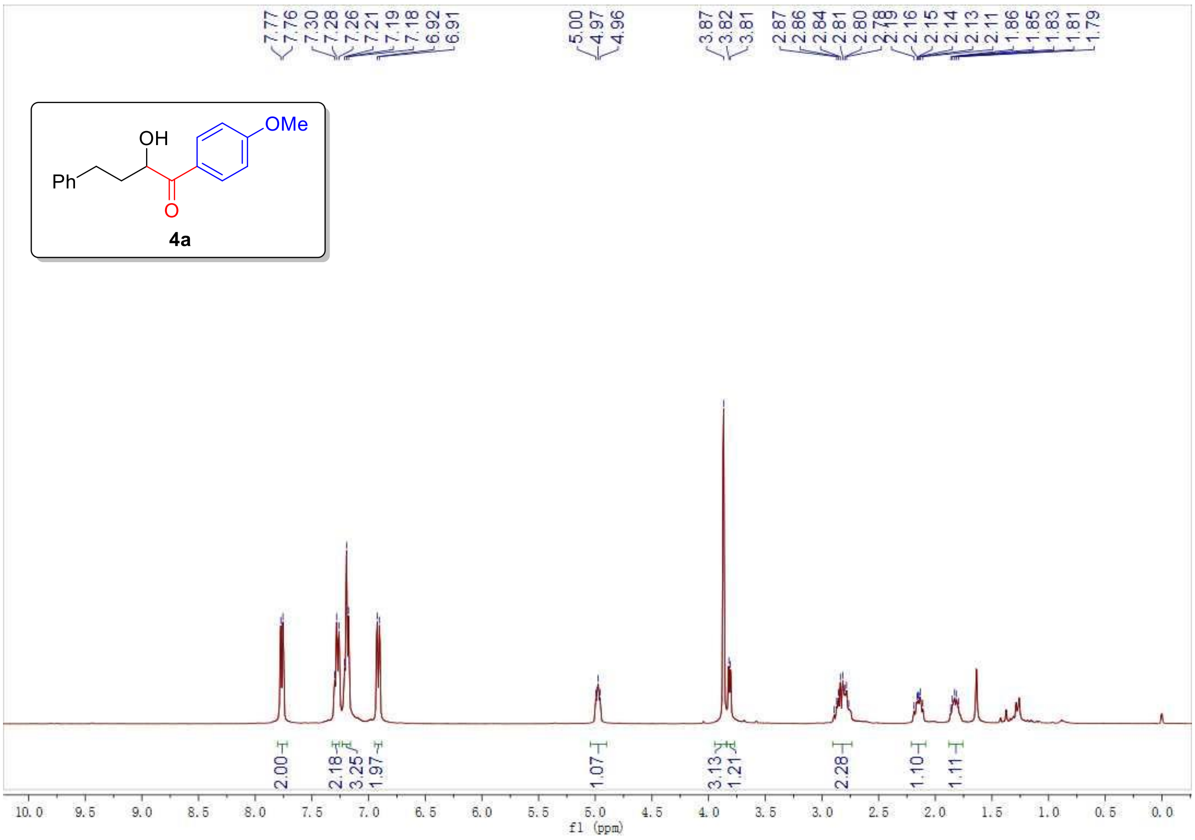

^{13}C NMR (101 MHz, $CDCl_3$) spectrum of 4a

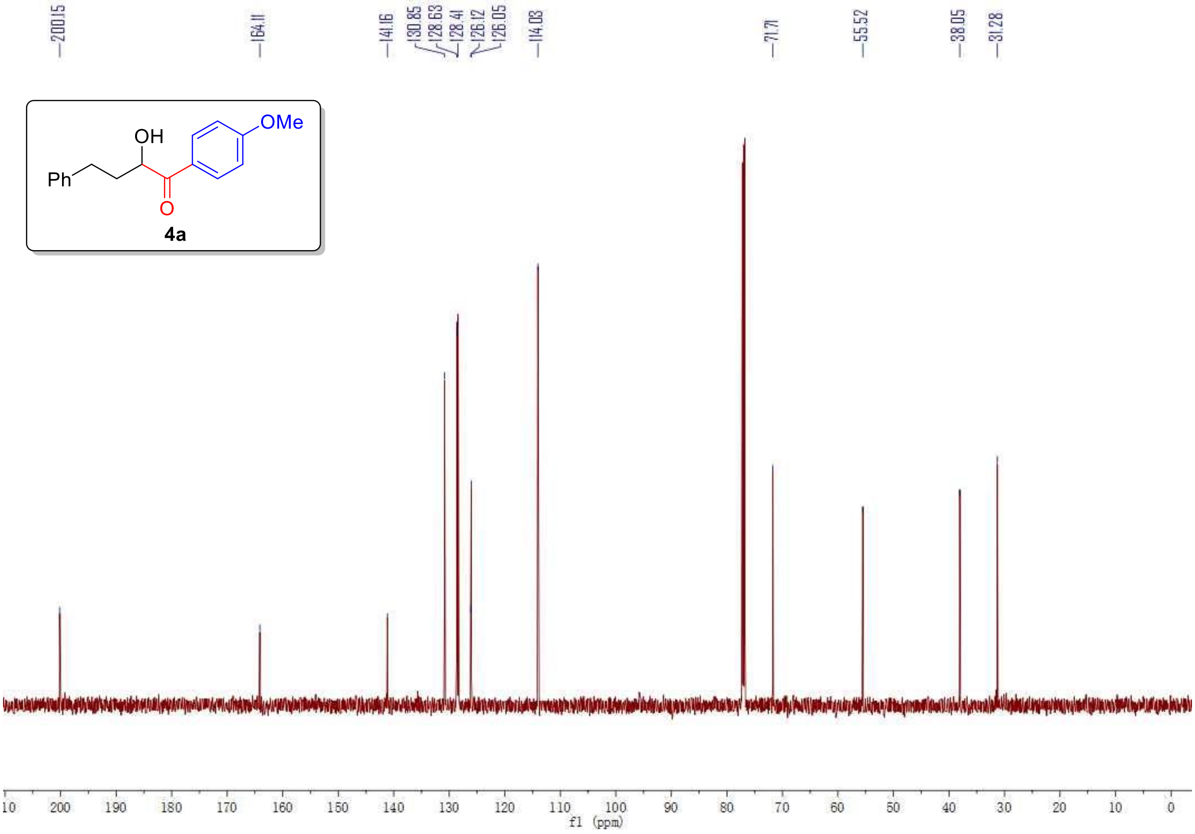

^{1}H NMR (400 MHz, $CDCl_3$) spectrum of 4b

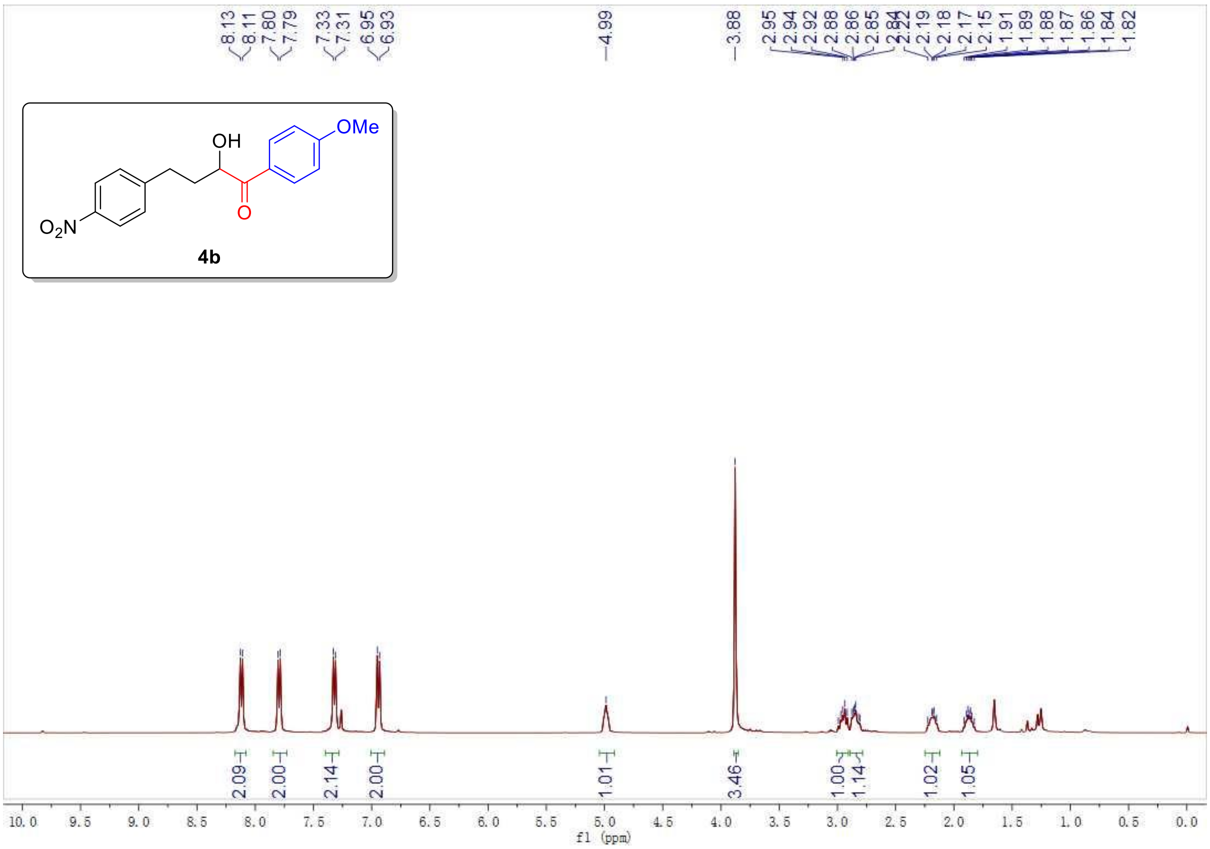

^{13}C NMR (101 MHz, $CDCl_3$) spectrum of 4b

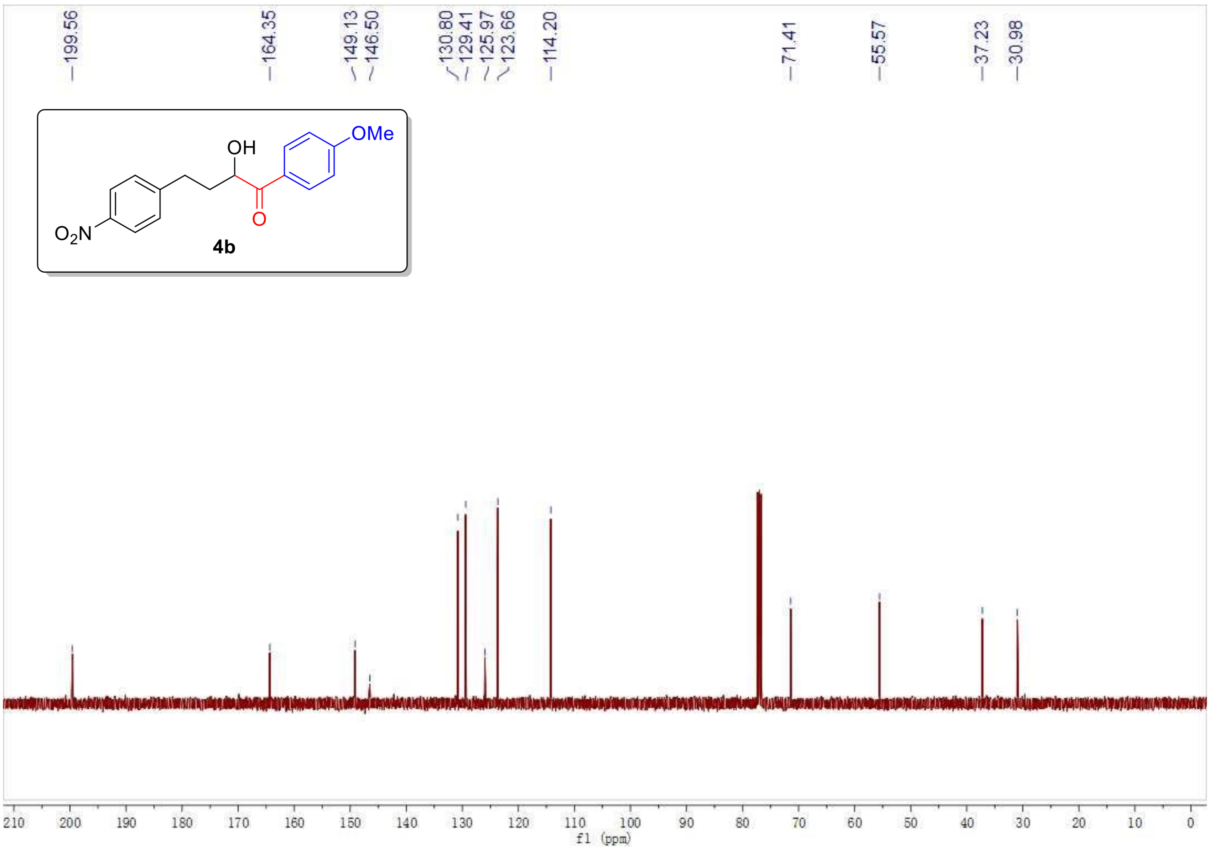

^1H NMR (400 MHz, $CDCl_3$) spectrum of 4c

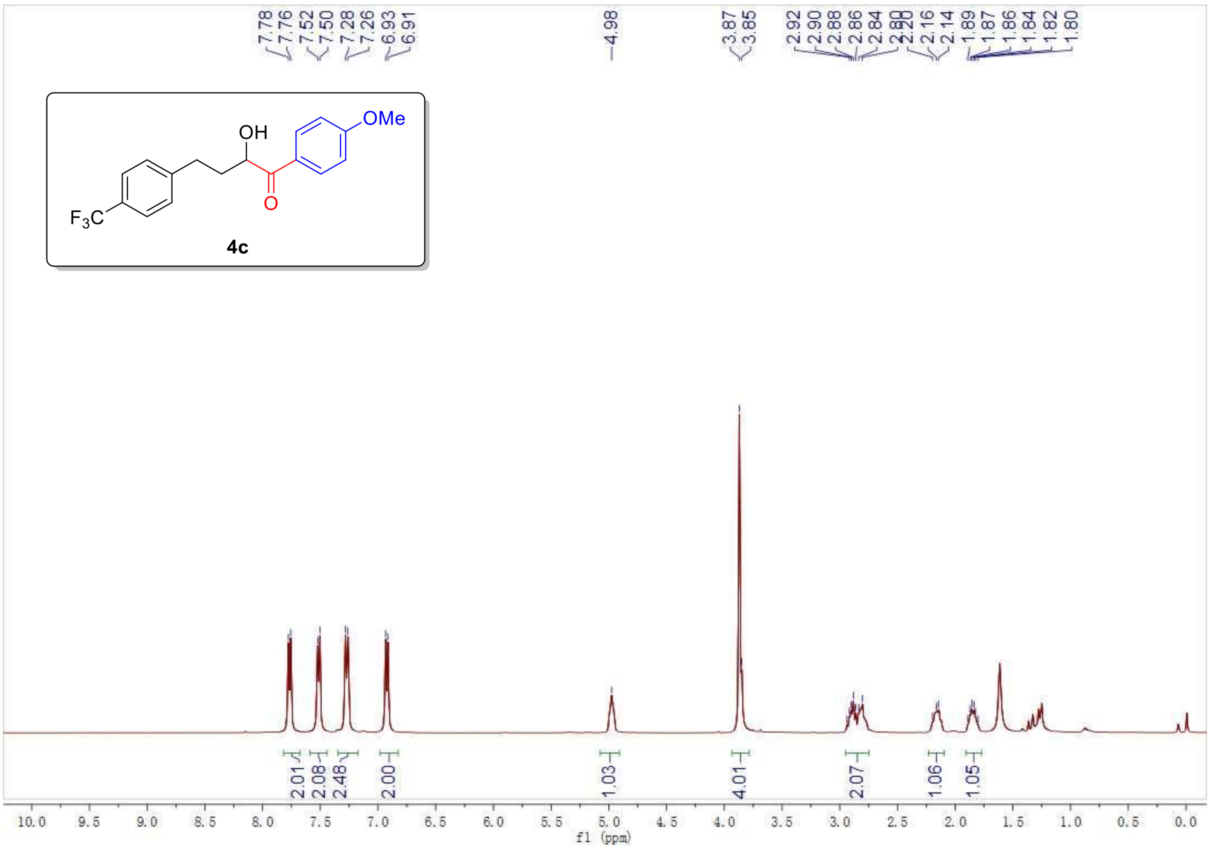

^{13}C NMR (101 MHz, $CDCl_3$) spectrum of 4c

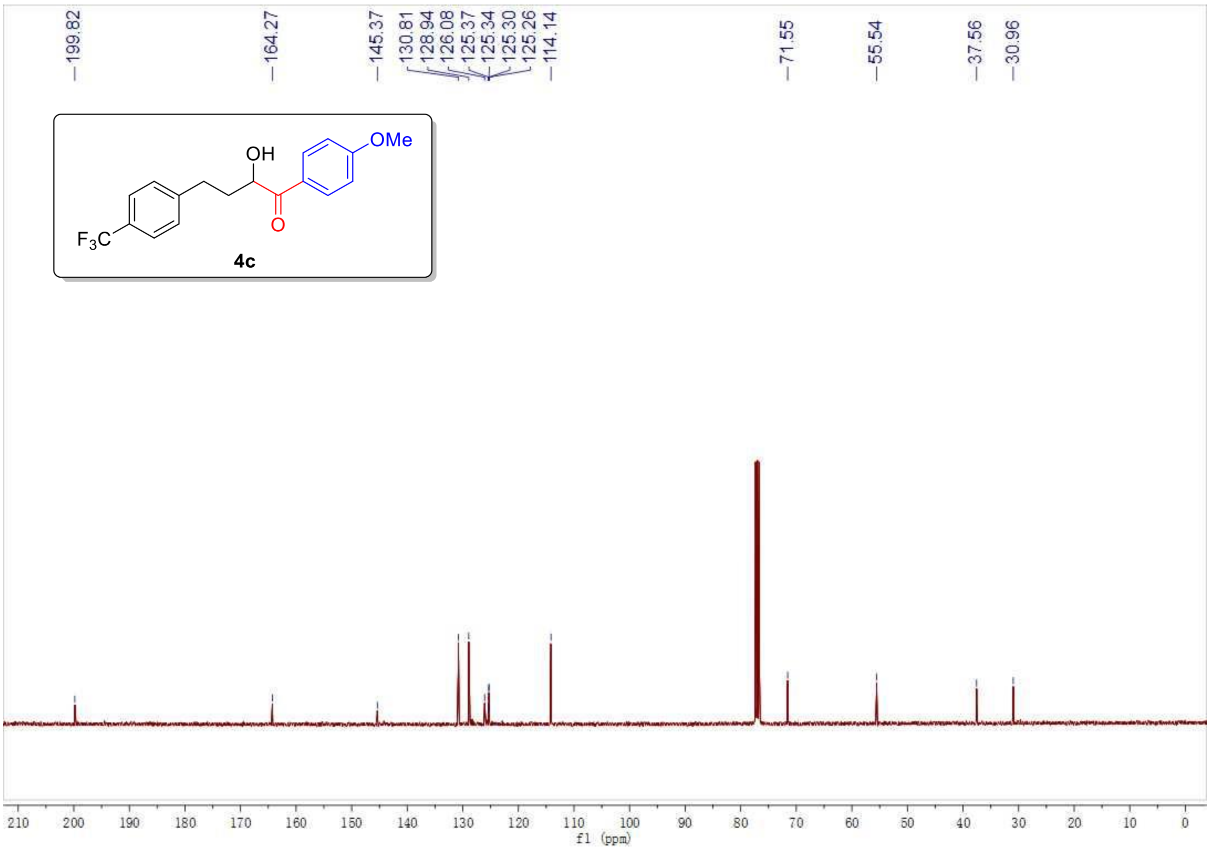

^{1}H NMR (400 MHz, $CDCl_3$) spectrum of 4d

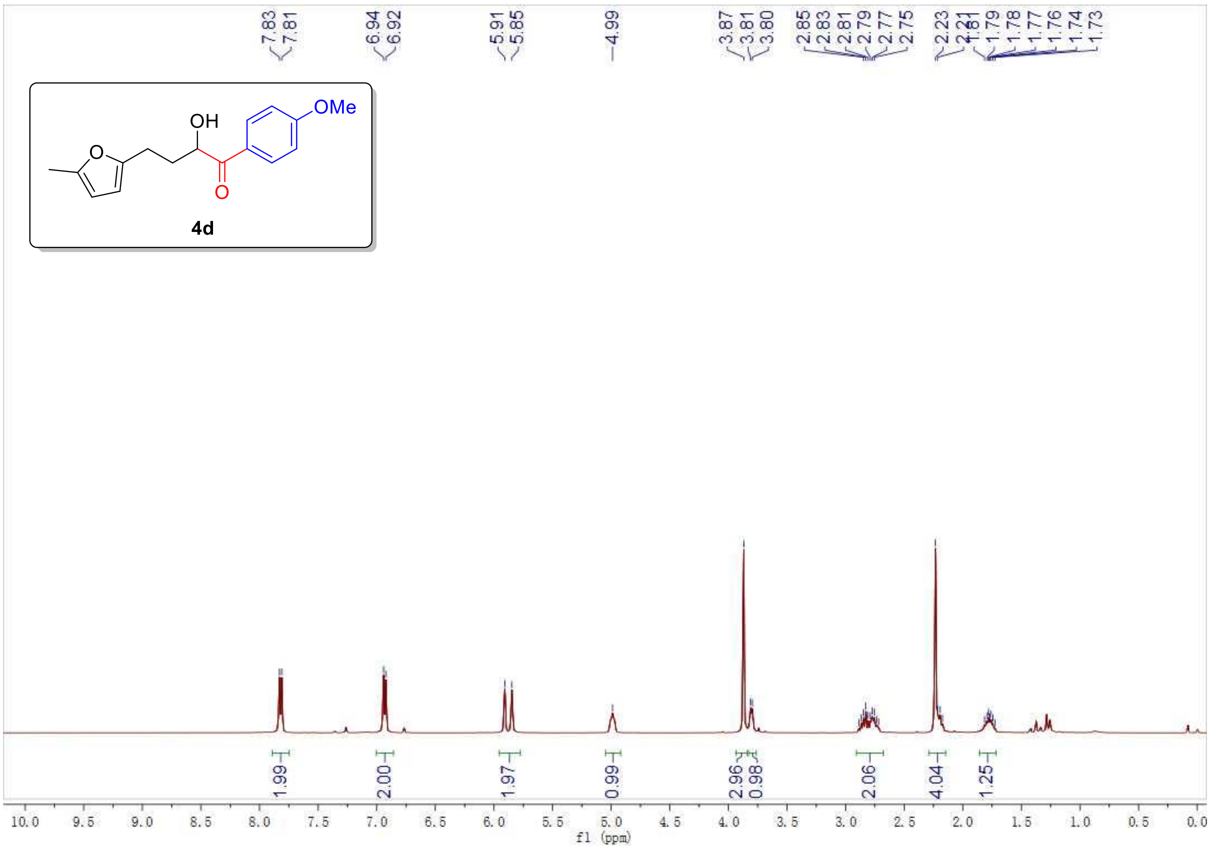

^{13}C NMR (101 MHz, $CDCl_3$) spectrum of 4d

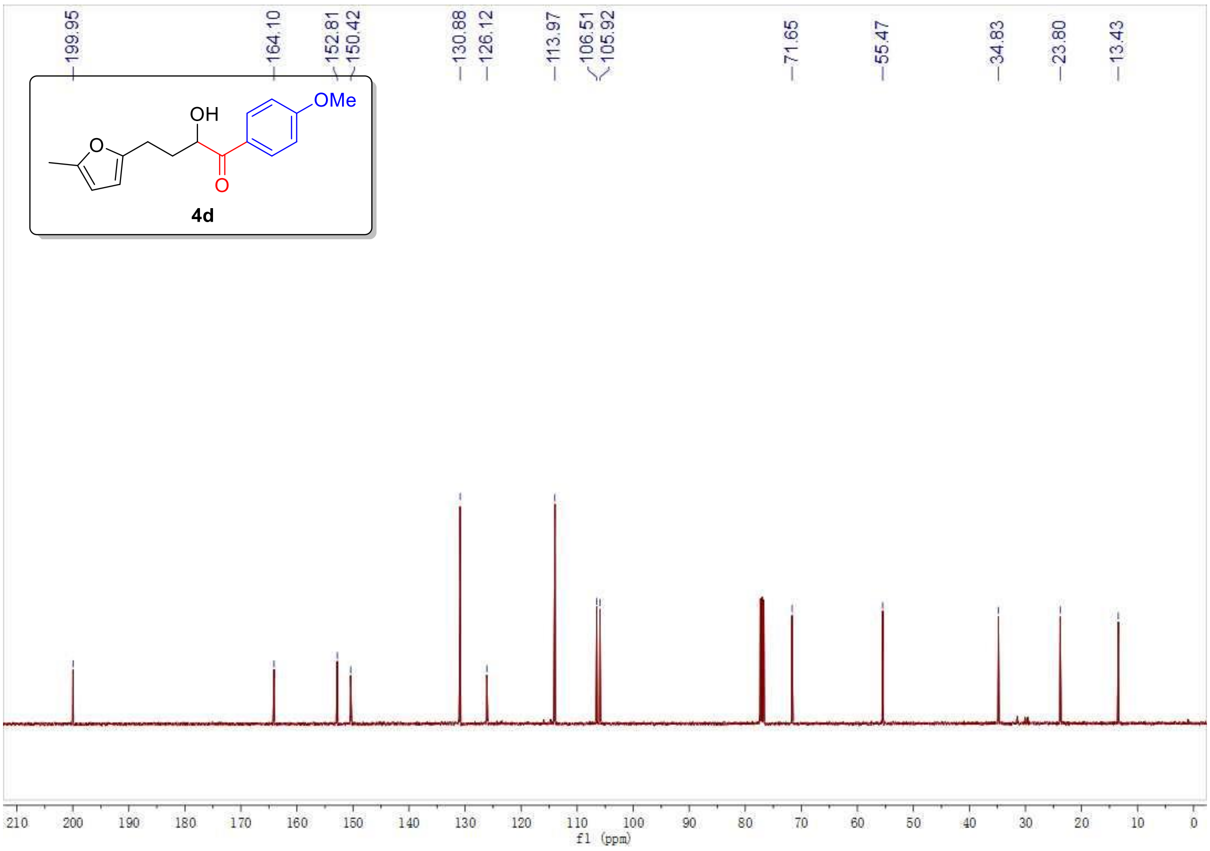

^{1}H NMR (400 MHz, $CDCl_3$) spectrum of 4e

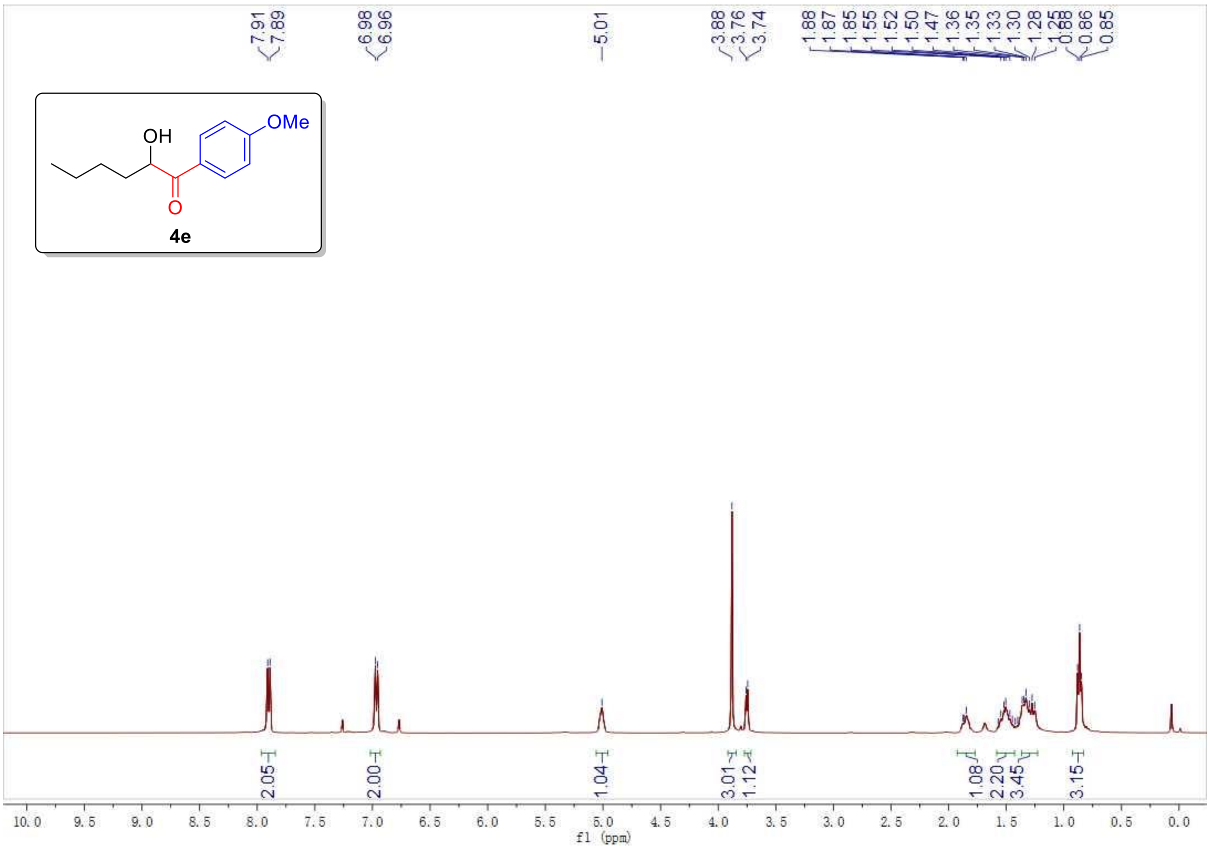

^{13}C NMR (101 MHz, $CDCl_3$) spectrum of 4e

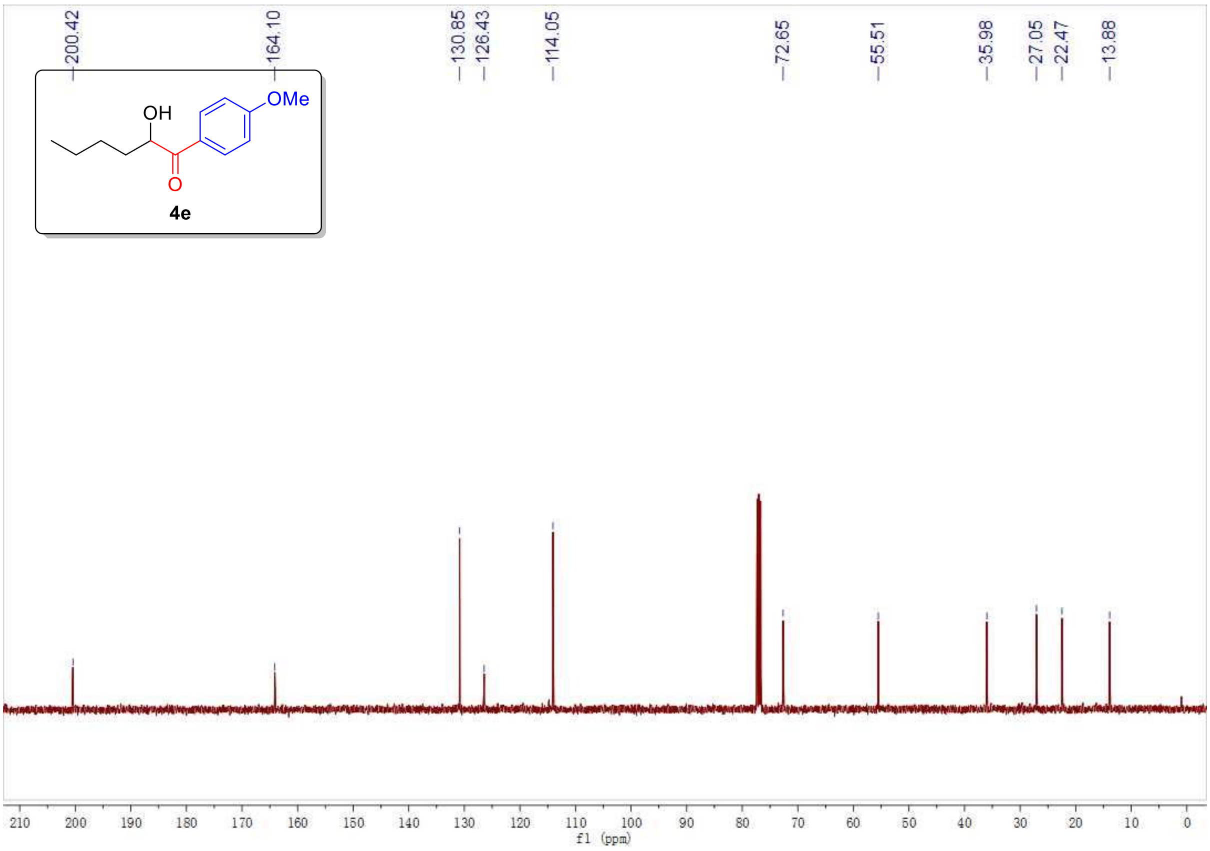

^{1}H NMR (400 MHz, $CDCl_3$) spectrum of 4f

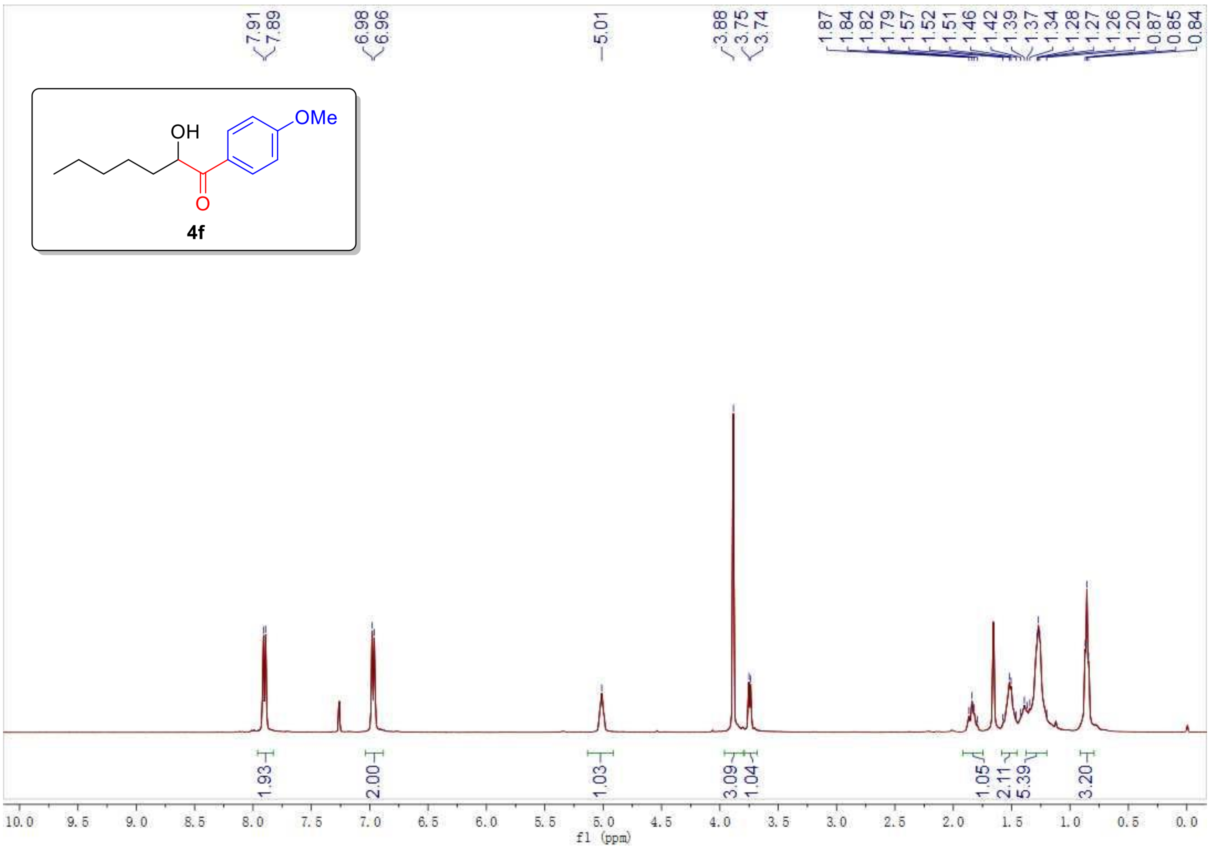

^{13}C NMR (101 MHz, $CDCl_3$) spectrum of 4f

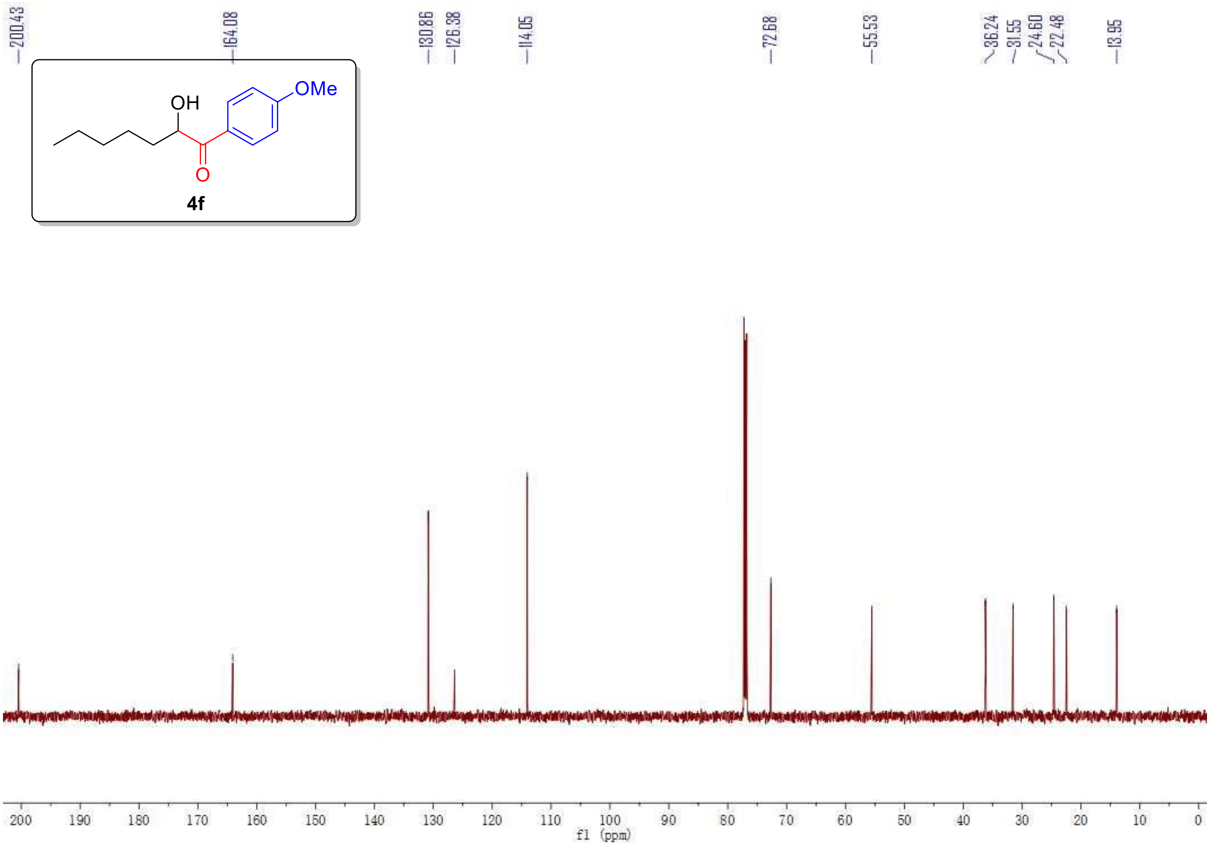

^{1}H NMR (400 MHz, $CDCl_3$) spectrum of 4g

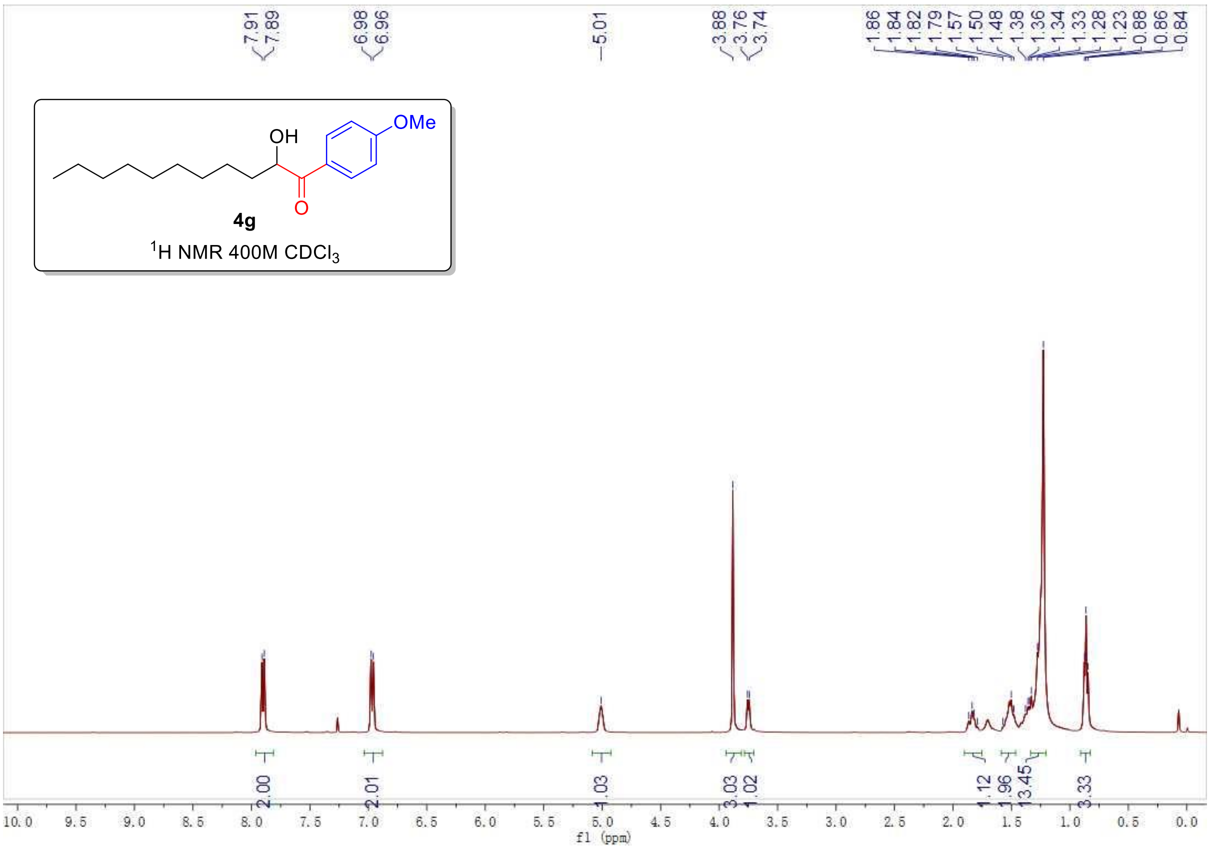

^{13}C NMR (101 MHz, $CDCl_3$) spectrum of 4g

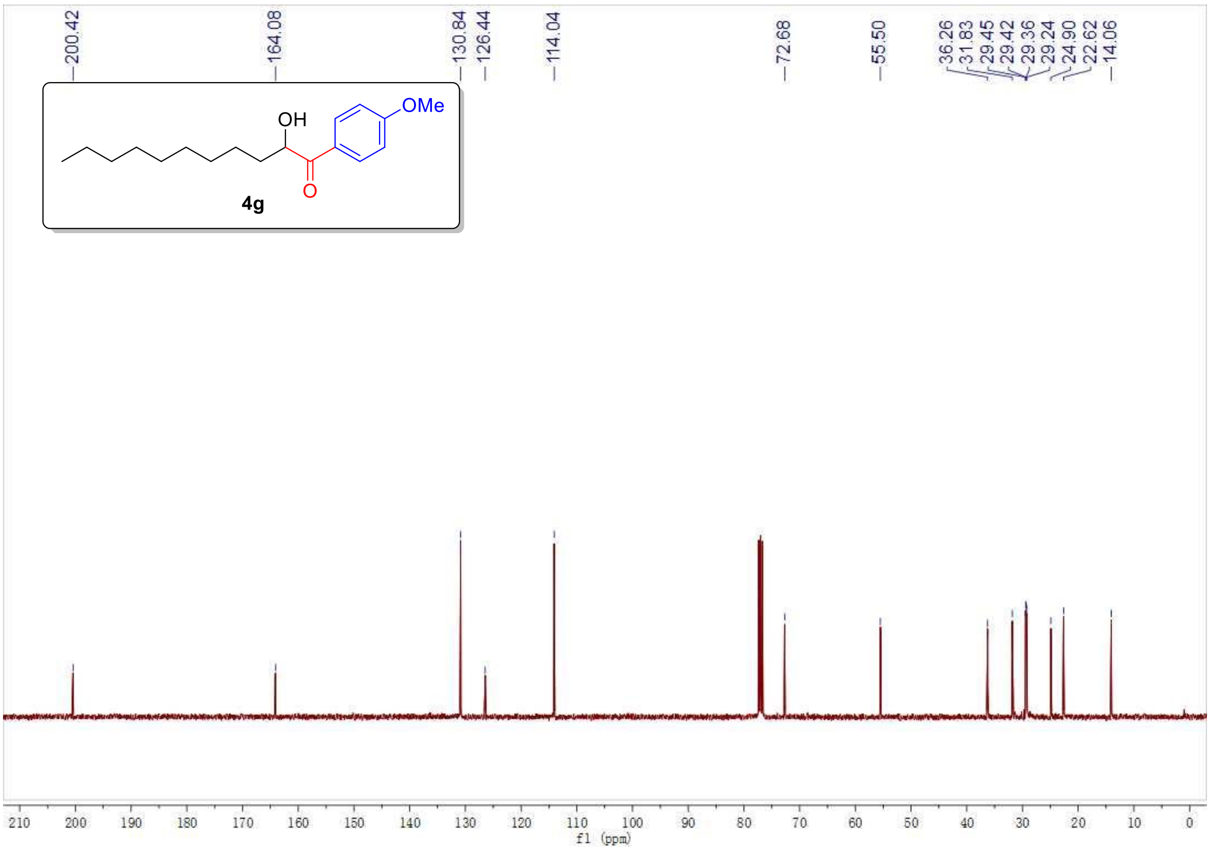

^{1}H NMR (400 MHz, $CDCl_3$) spectrum of 4h

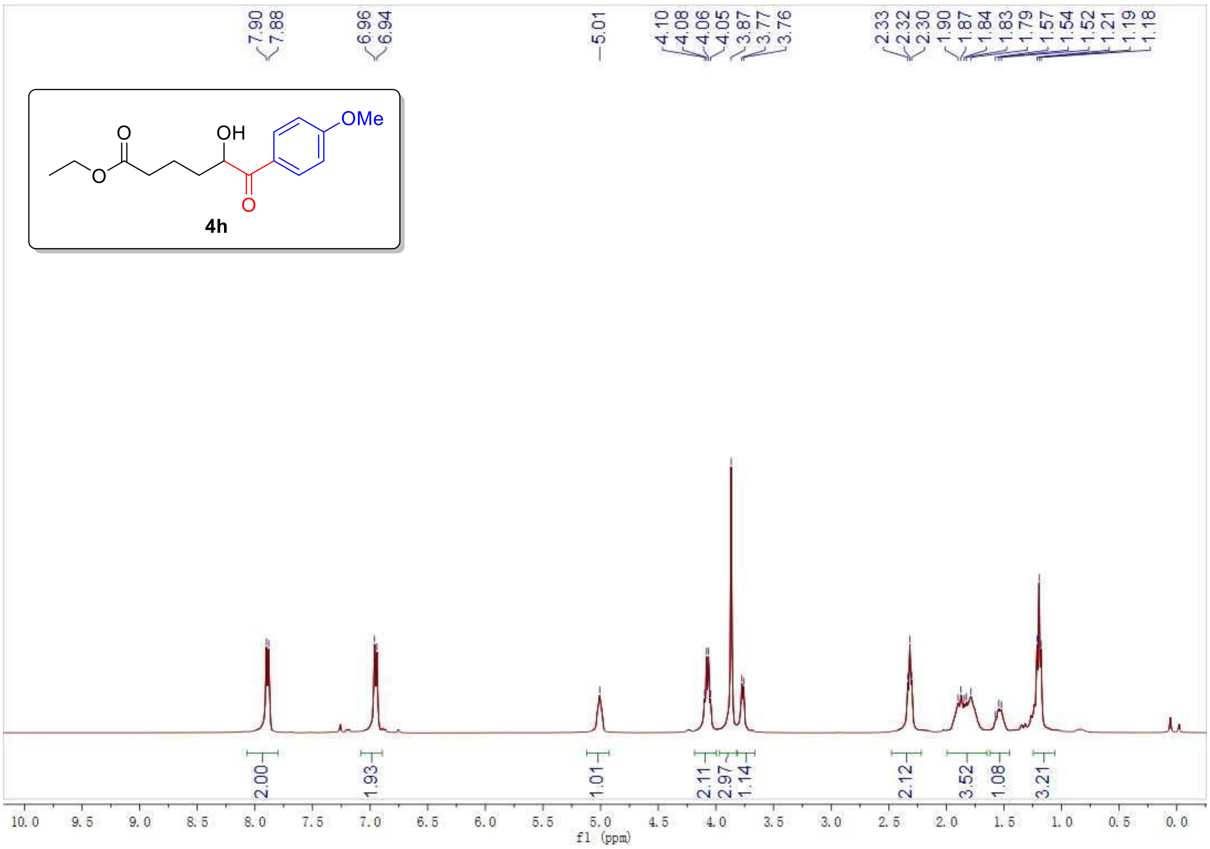

^{13}C NMR (101 MHz, $CDCl_3$) spectrum of 4h

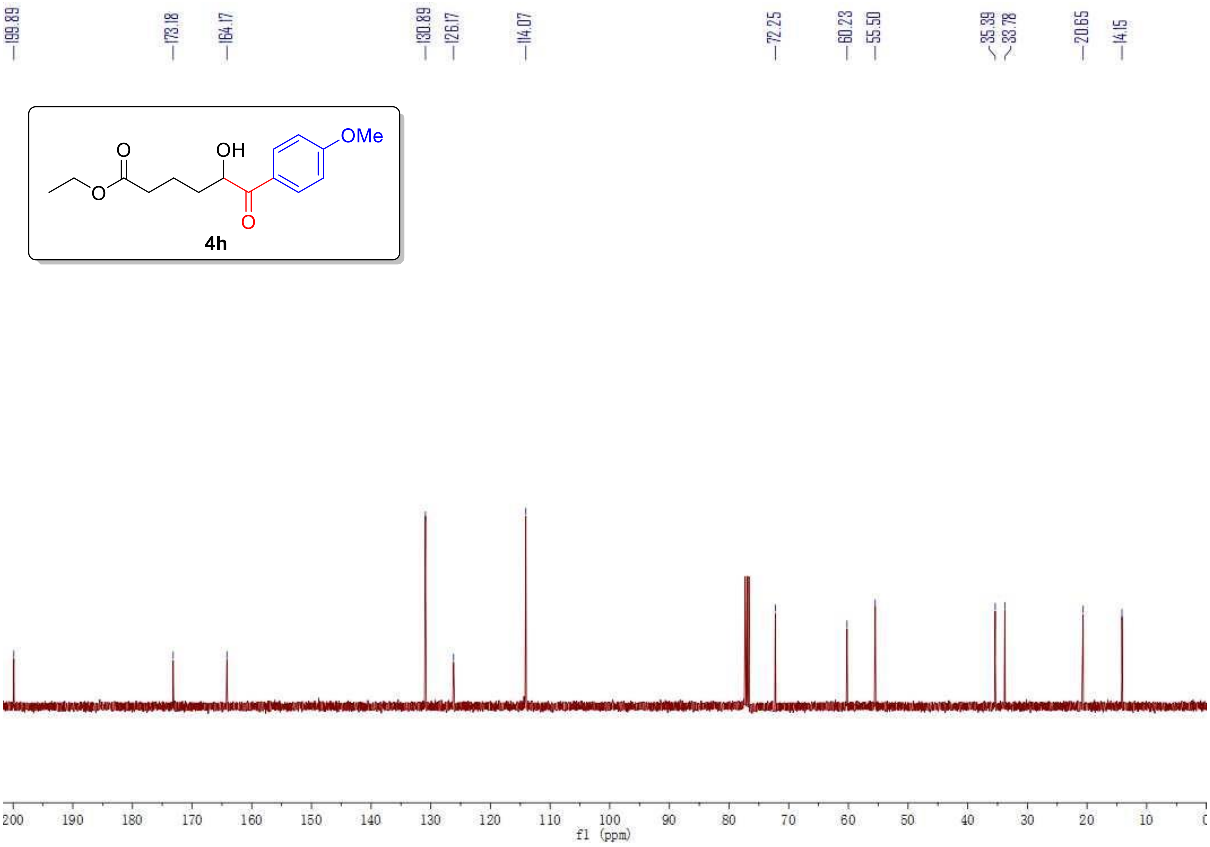

^{1}H NMR (400 MHz, $CDCl_3$) spectrum of 4i

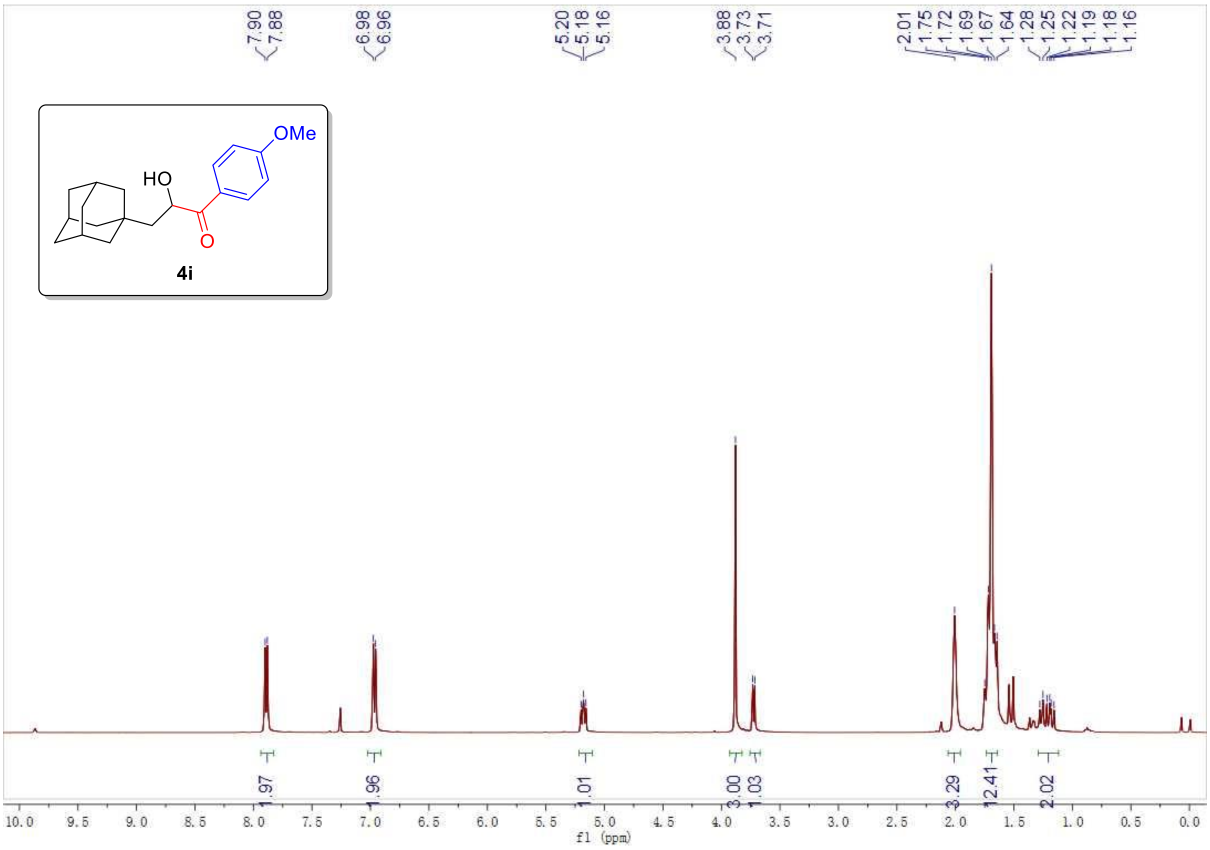

^{13}C NMR (101 MHz, $CDCl_3$) spectrum of 4i

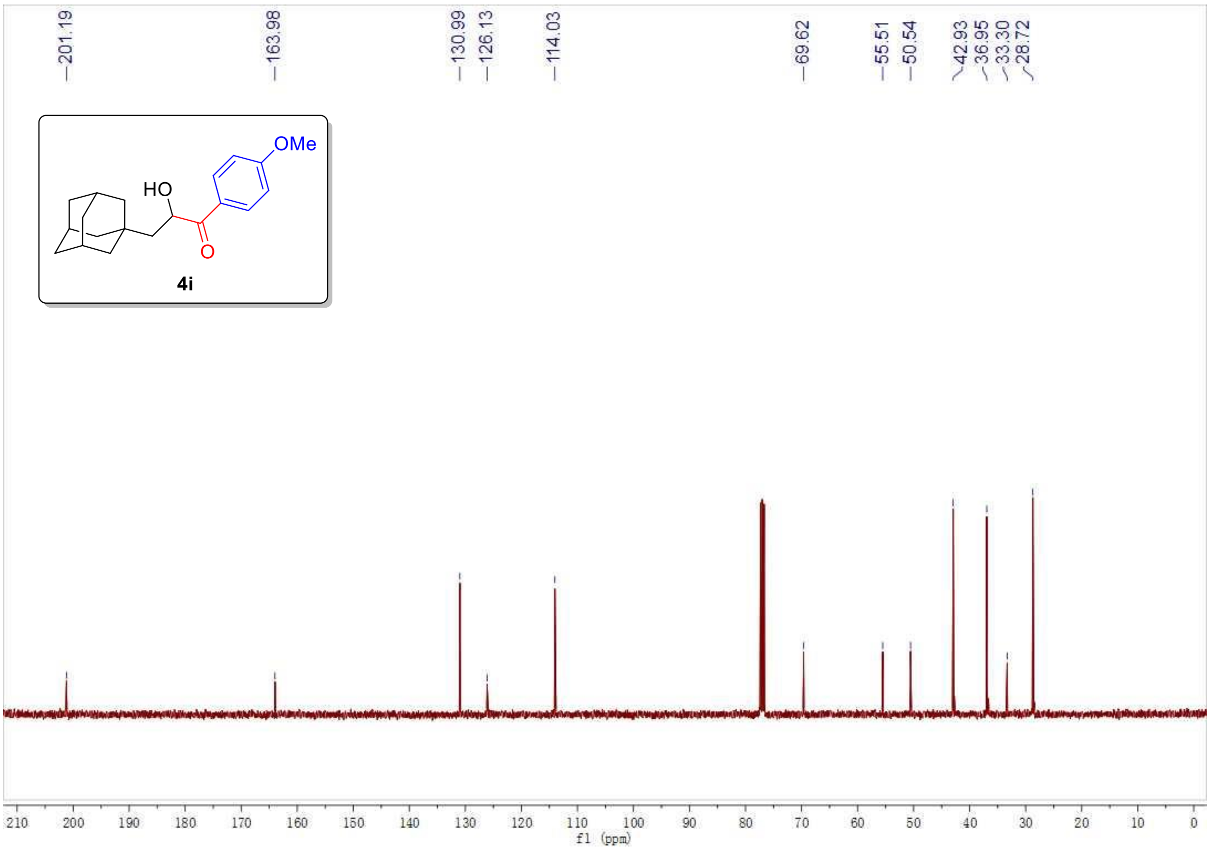

^{1}H NMR (400 MHz, $CDCl_3$) spectrum of 4j

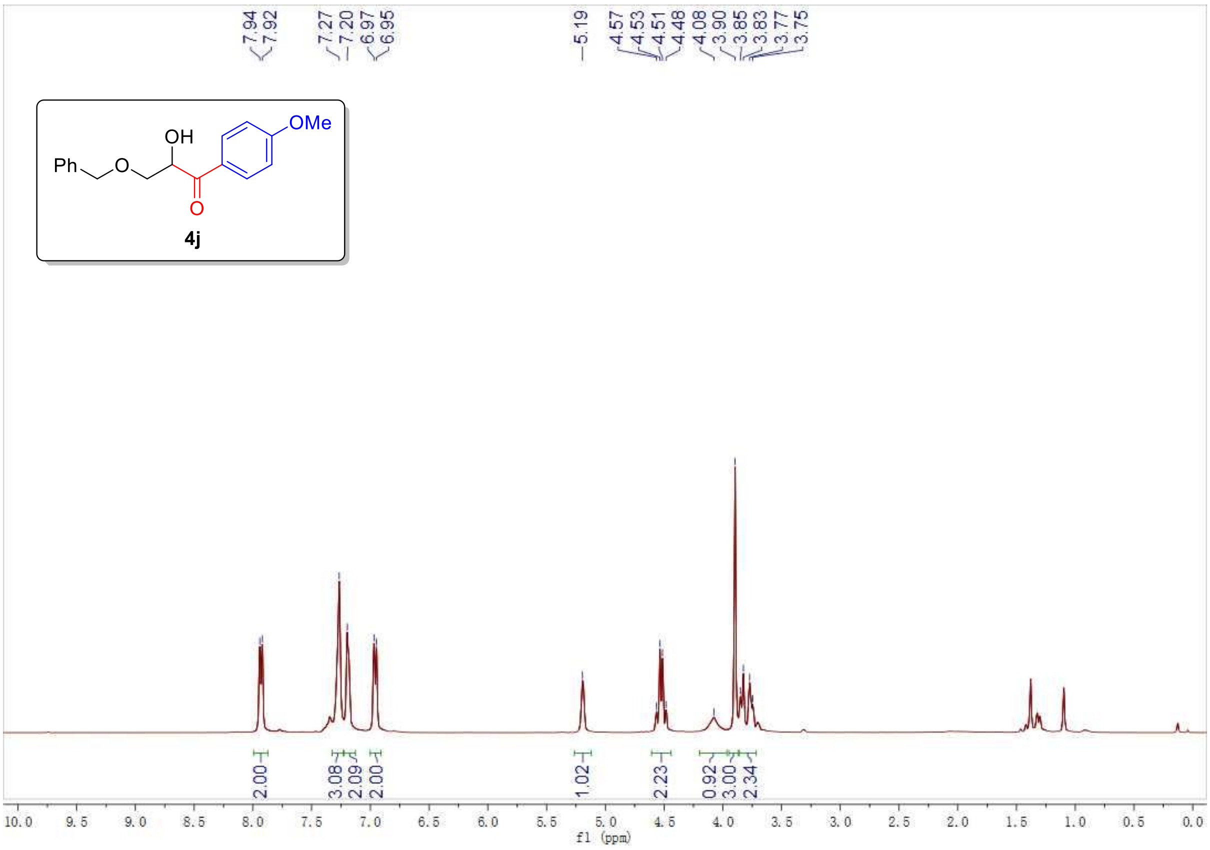

^{13}C NMR (101 MHz, $CDCl_3$) spectrum of 4j

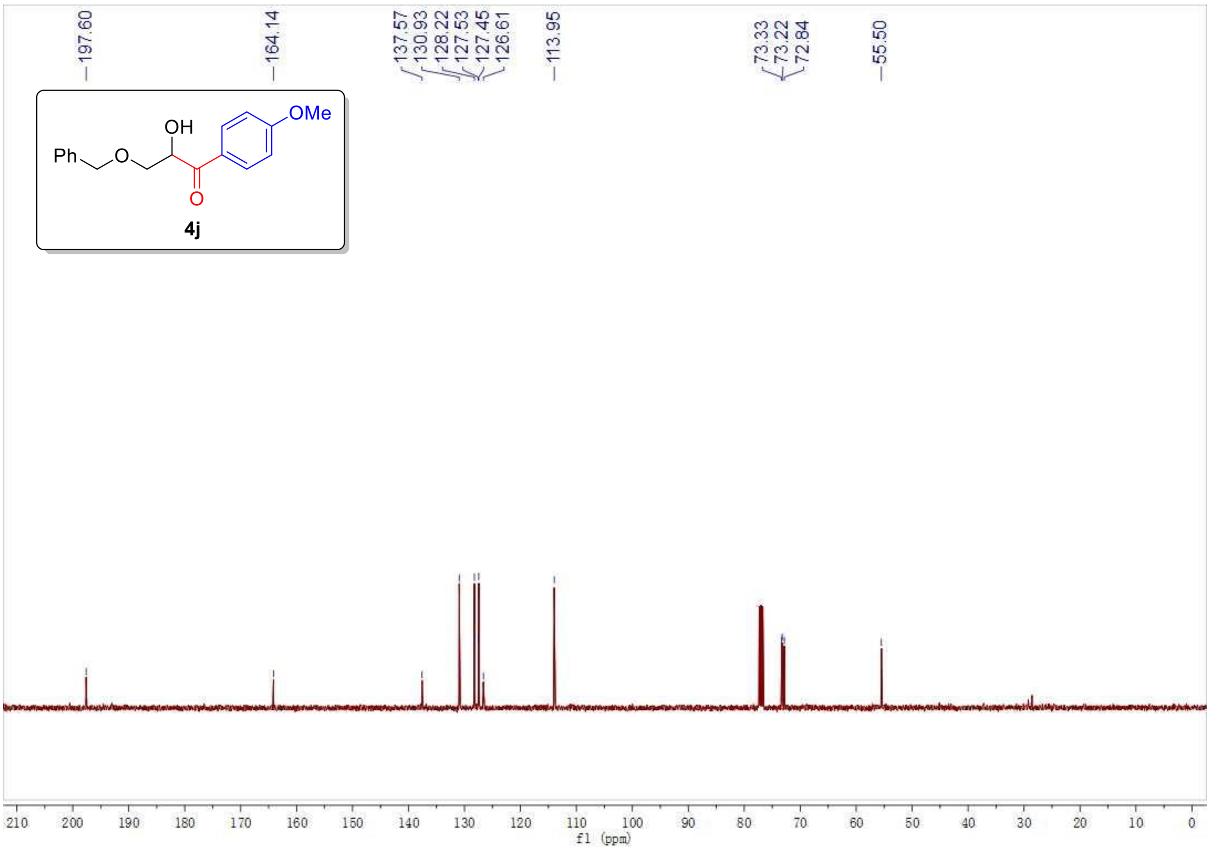

^{1}H NMR (400 MHz, $CDCl_3$) spectrum of 4k

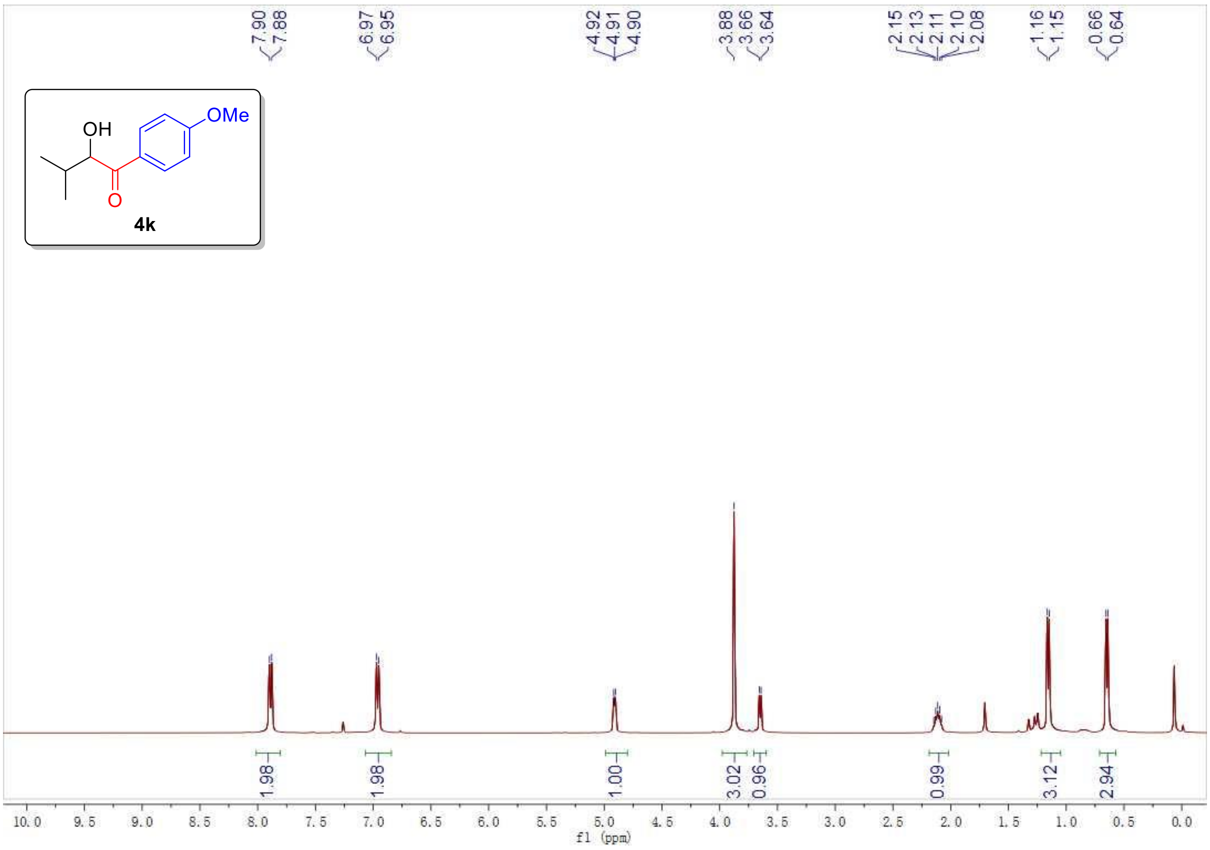

^{13}C NMR (101 MHz, $CDCl_3$) spectrum of 4k

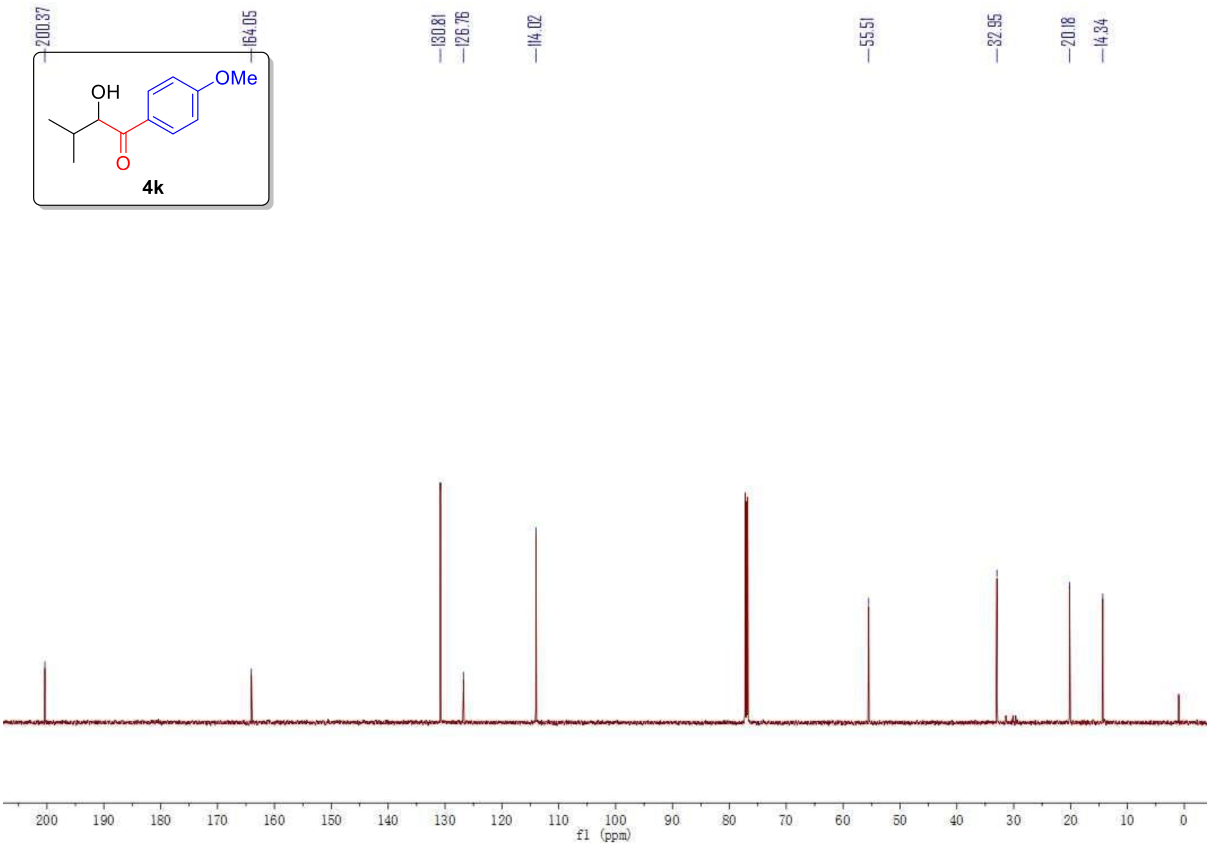

^{1}H NMR (400 MHz, $CDCl_3$) spectrum of 4l

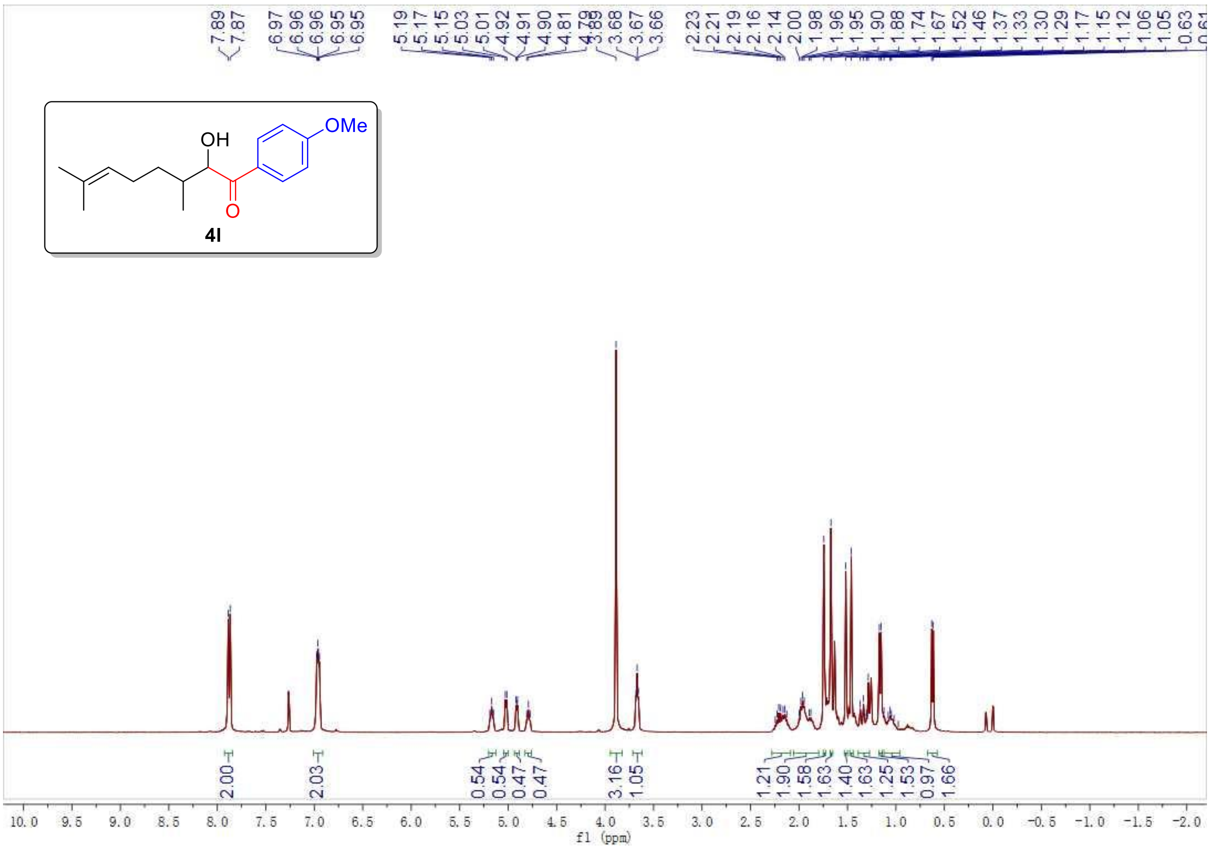

^{13}C NMR (101 MHz, $CDCl_3$) spectrum of 4l

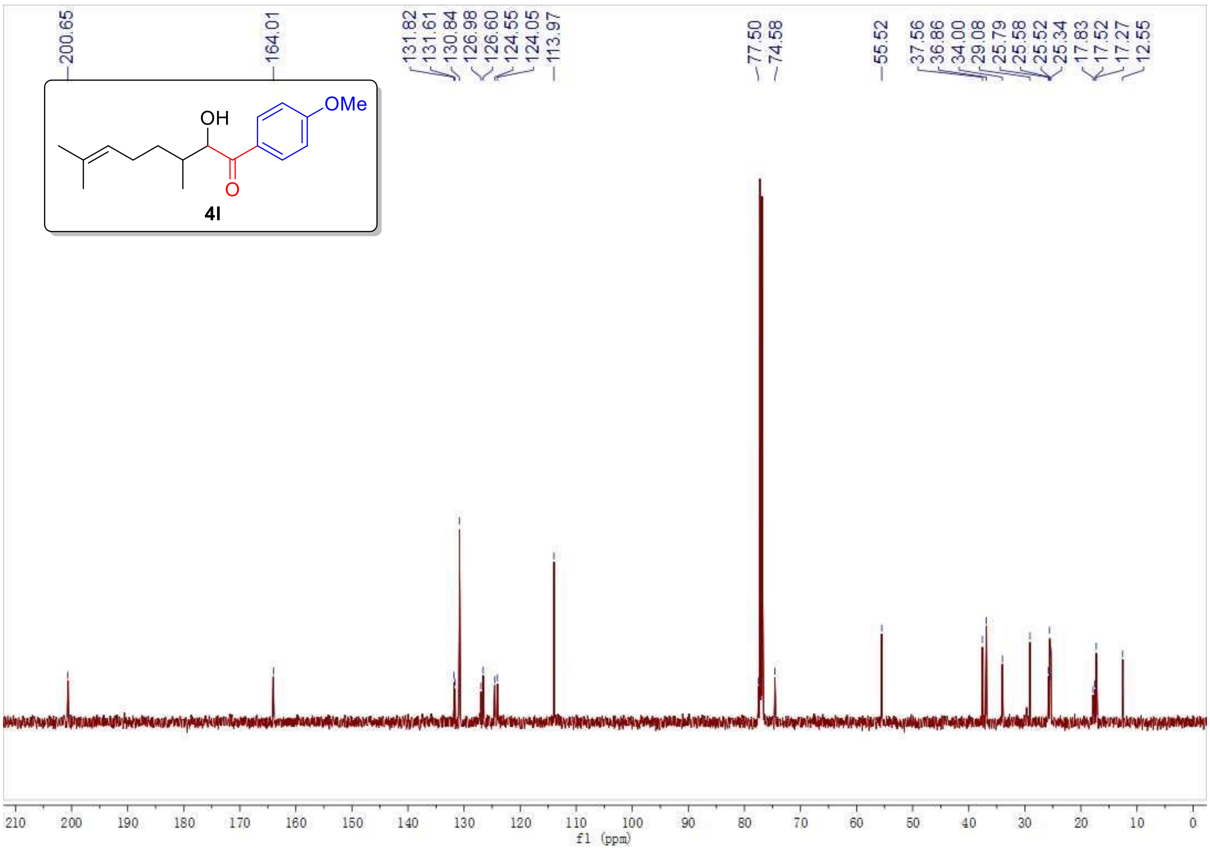

^{1}H NMR (400 MHz, $CDCl_3$) spectrum of 4m

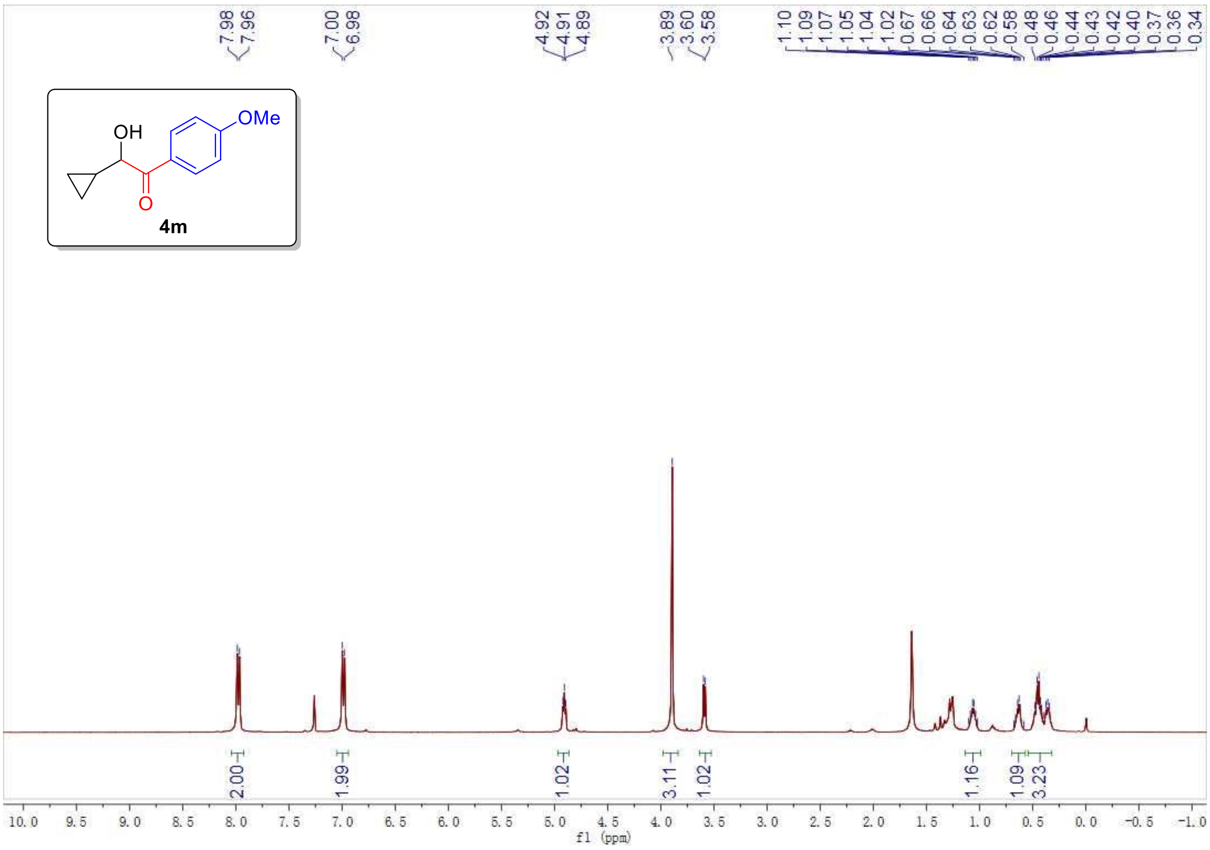

^{13}C NMR (400 MHz, $CDCl_3$) spectrum of 4m

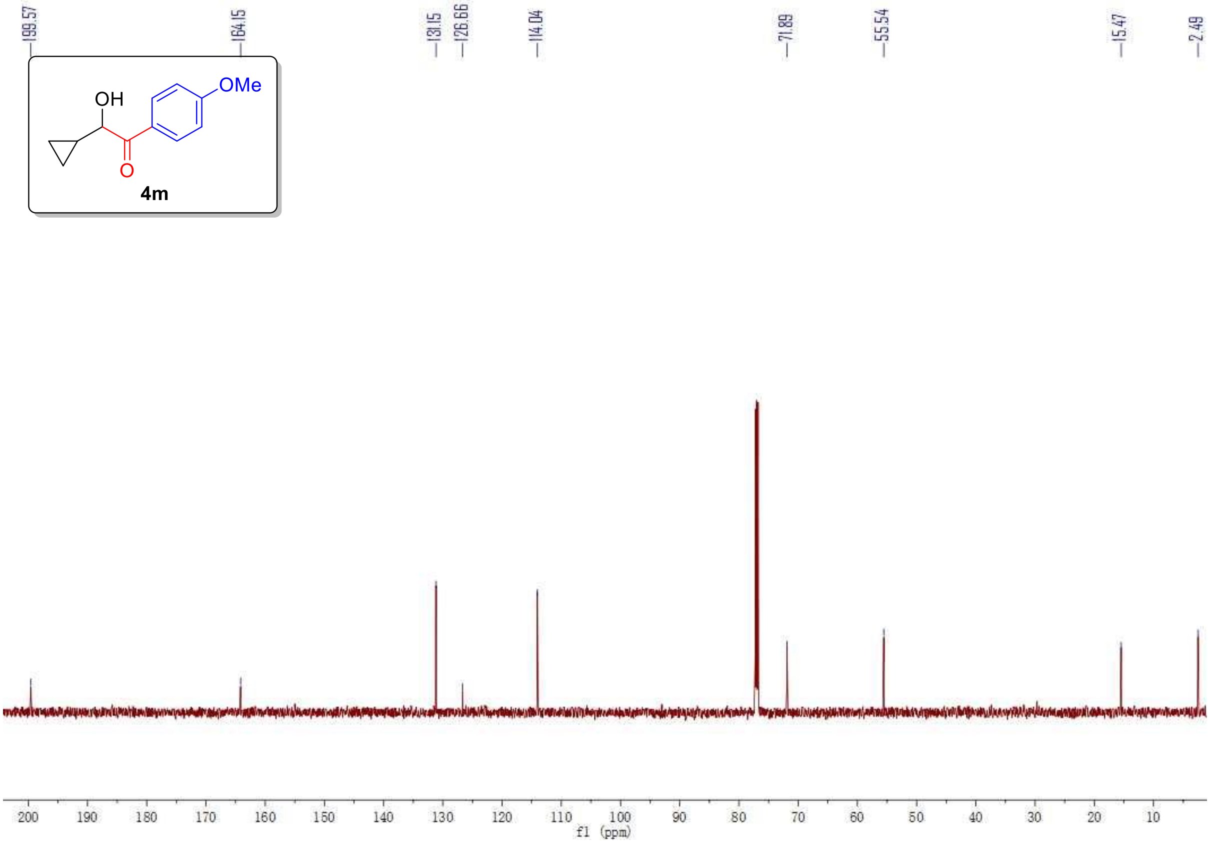

^{1}H NMR (400 MHz, $CDCl_3$) spectrum of 4n

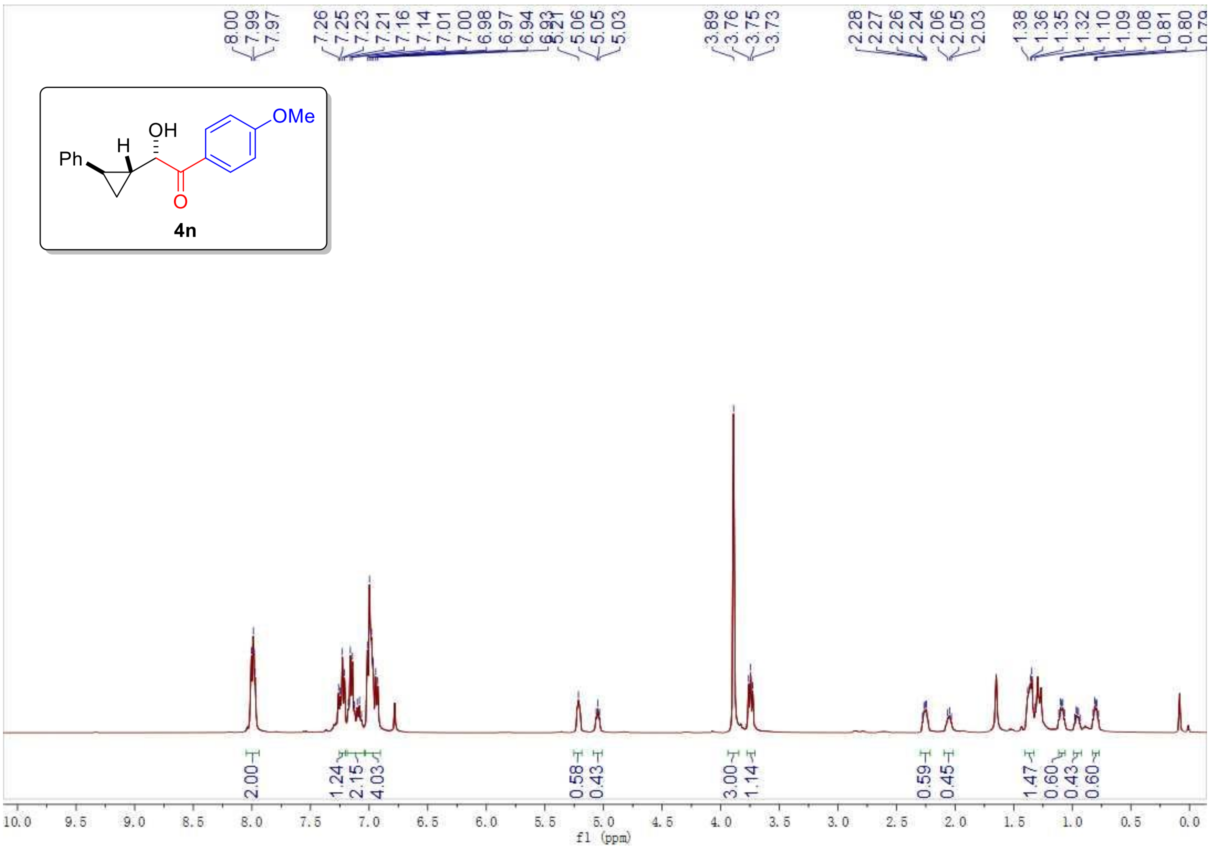

^{13}C NMR (101 MHz, $CDCl_3$) spectrum of 4n

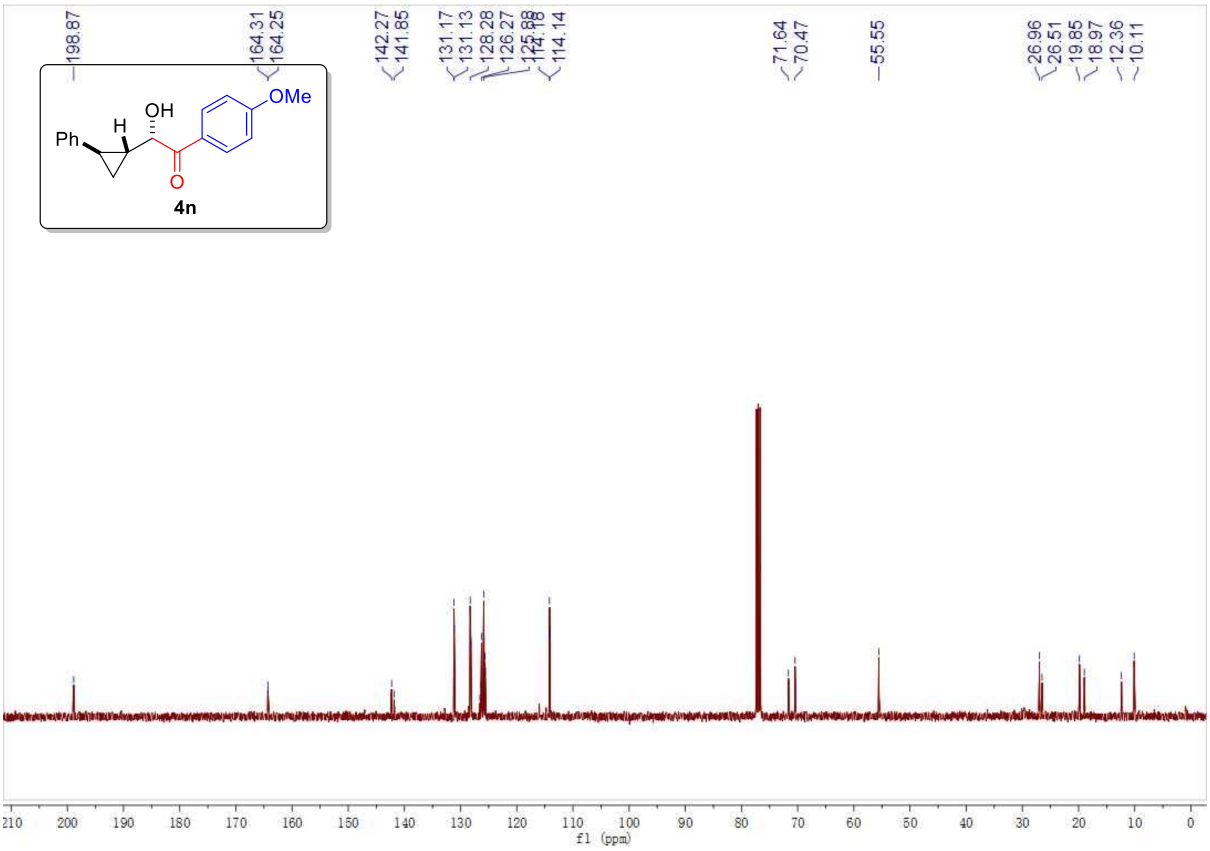

^{1}H NMR (400 MHz, $CDCl_3$) spectrum of 4o

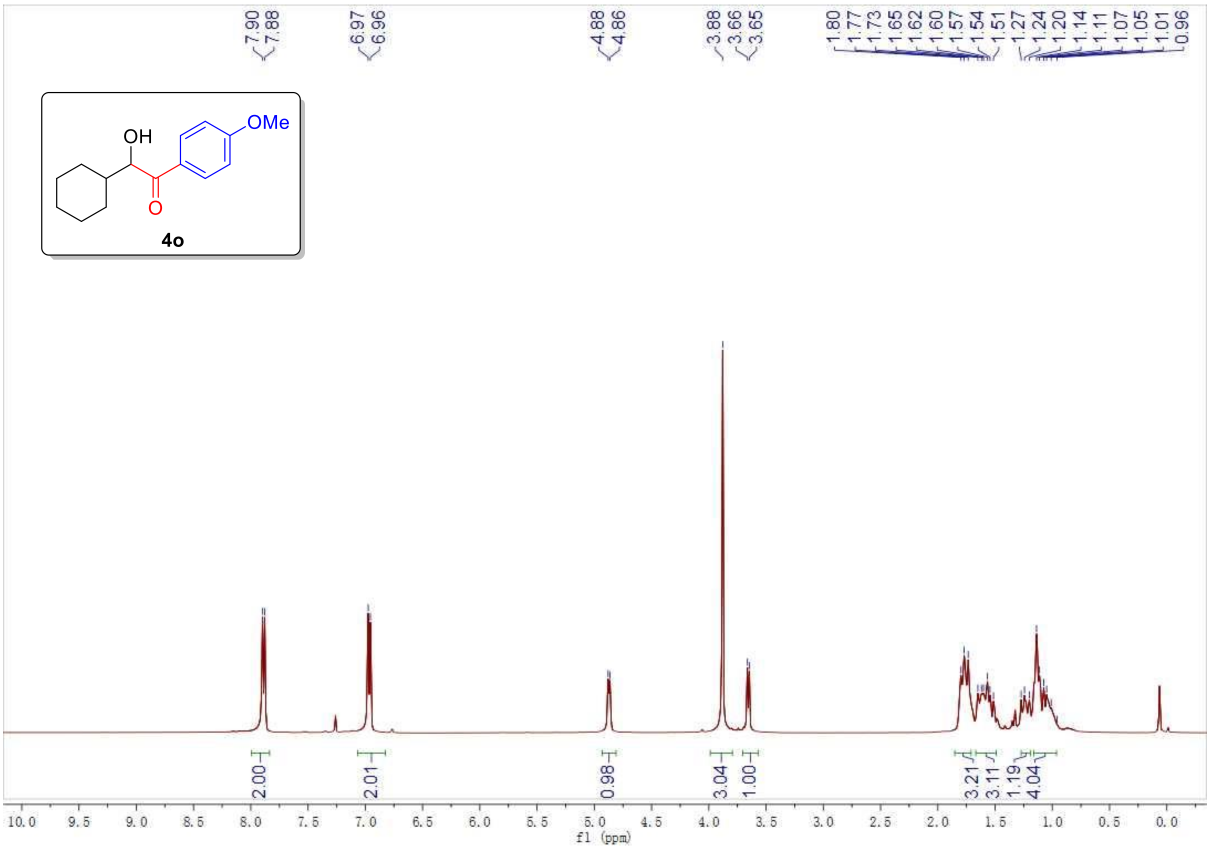

^{13}C NMR (101 MHz, $CDCl_3$) spectrum of 4o

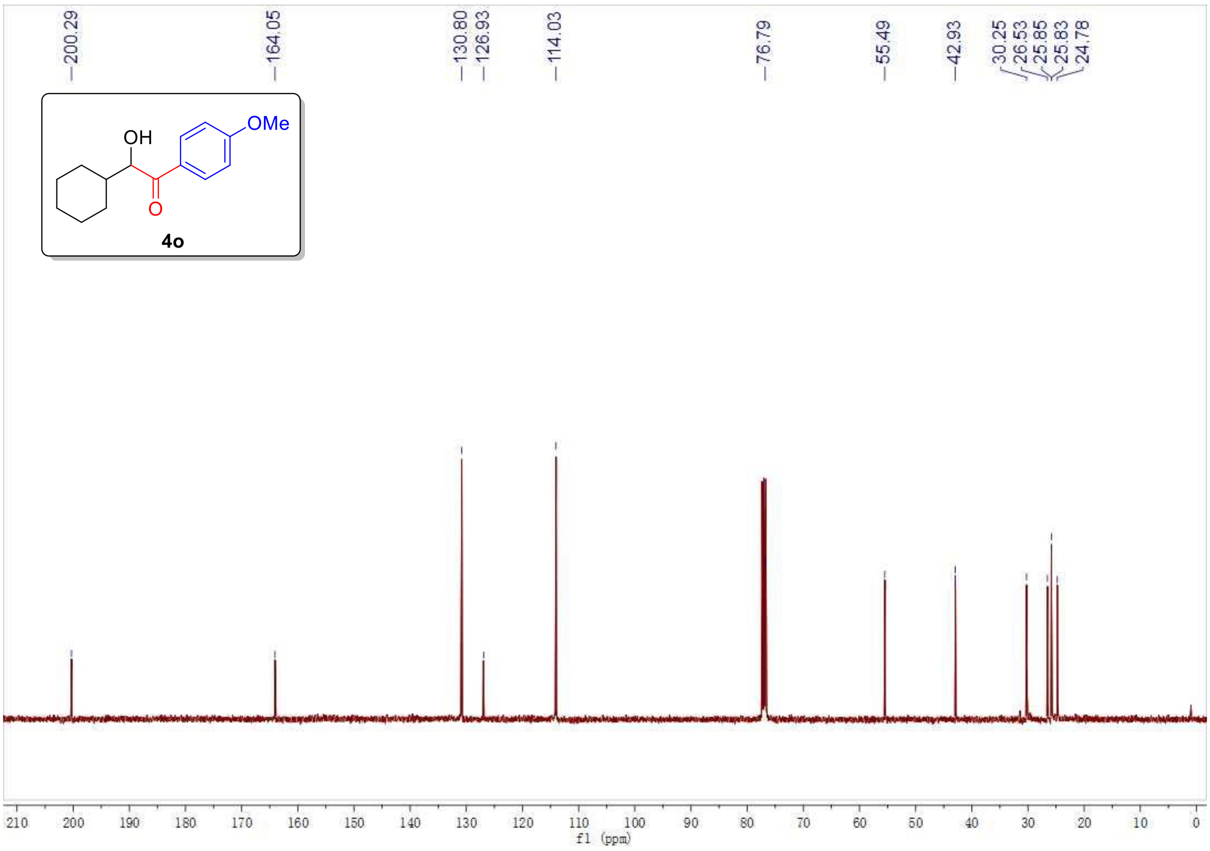

^{1}H NMR (400 MHz, $CDCl_3$) spectrum of 4p

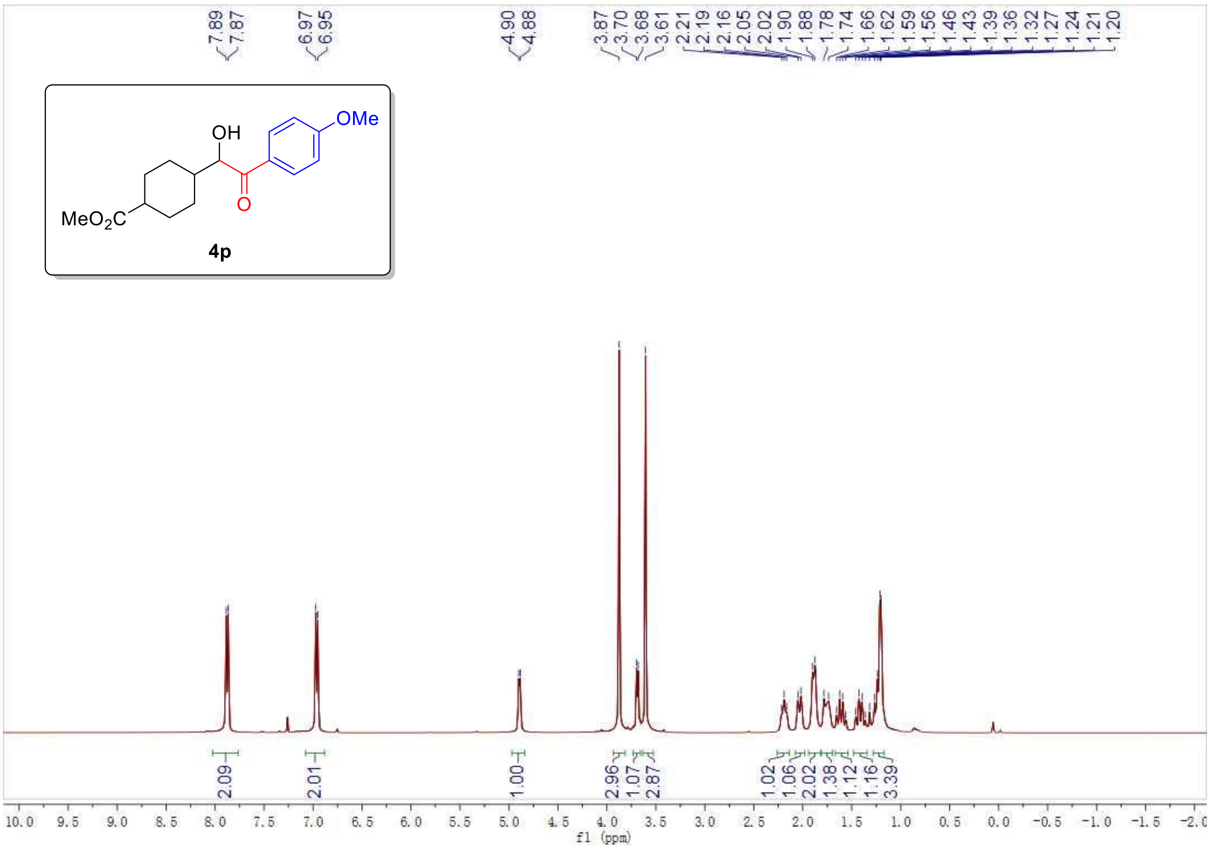

^{13}C NMR (101 MHz, $CDCl_3$) spectrum of 4p

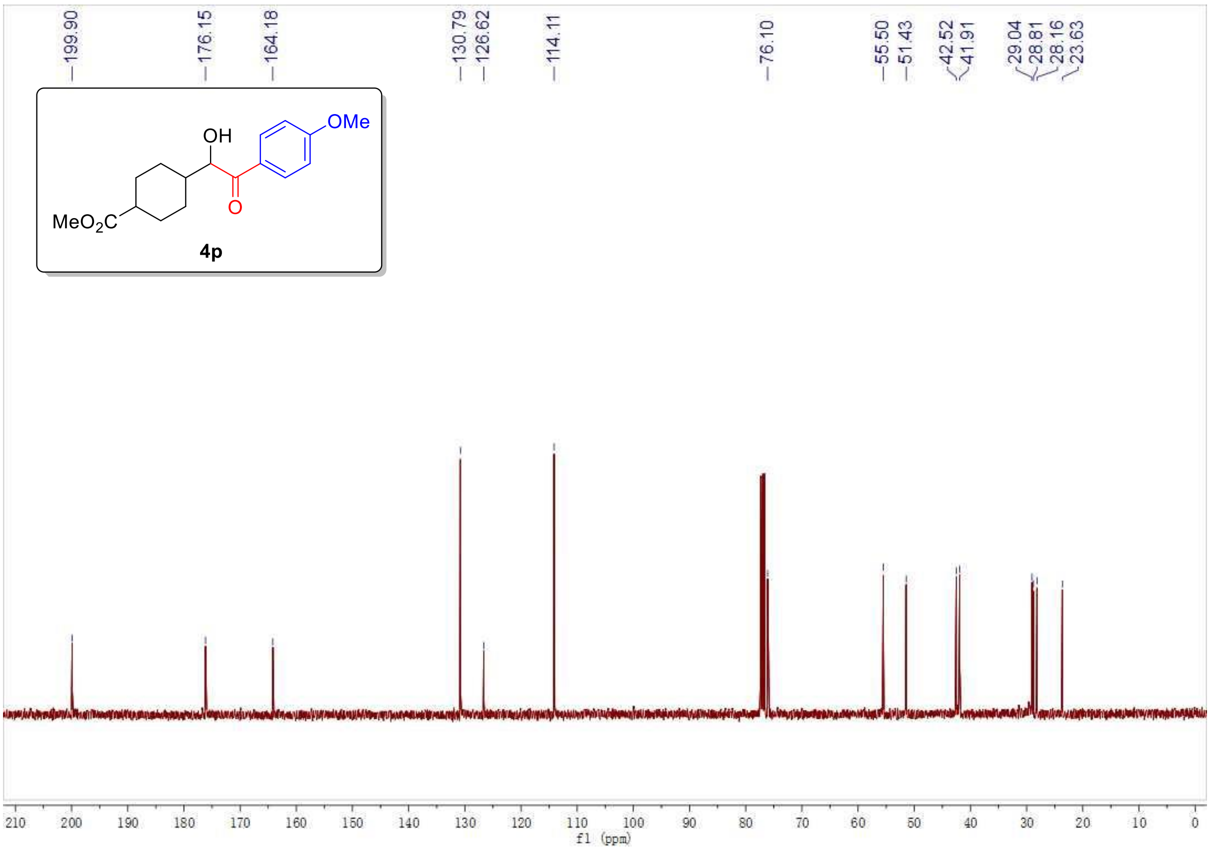

^{1}H NMR (400 MHz, $CDCl_3$) spectrum of 4q

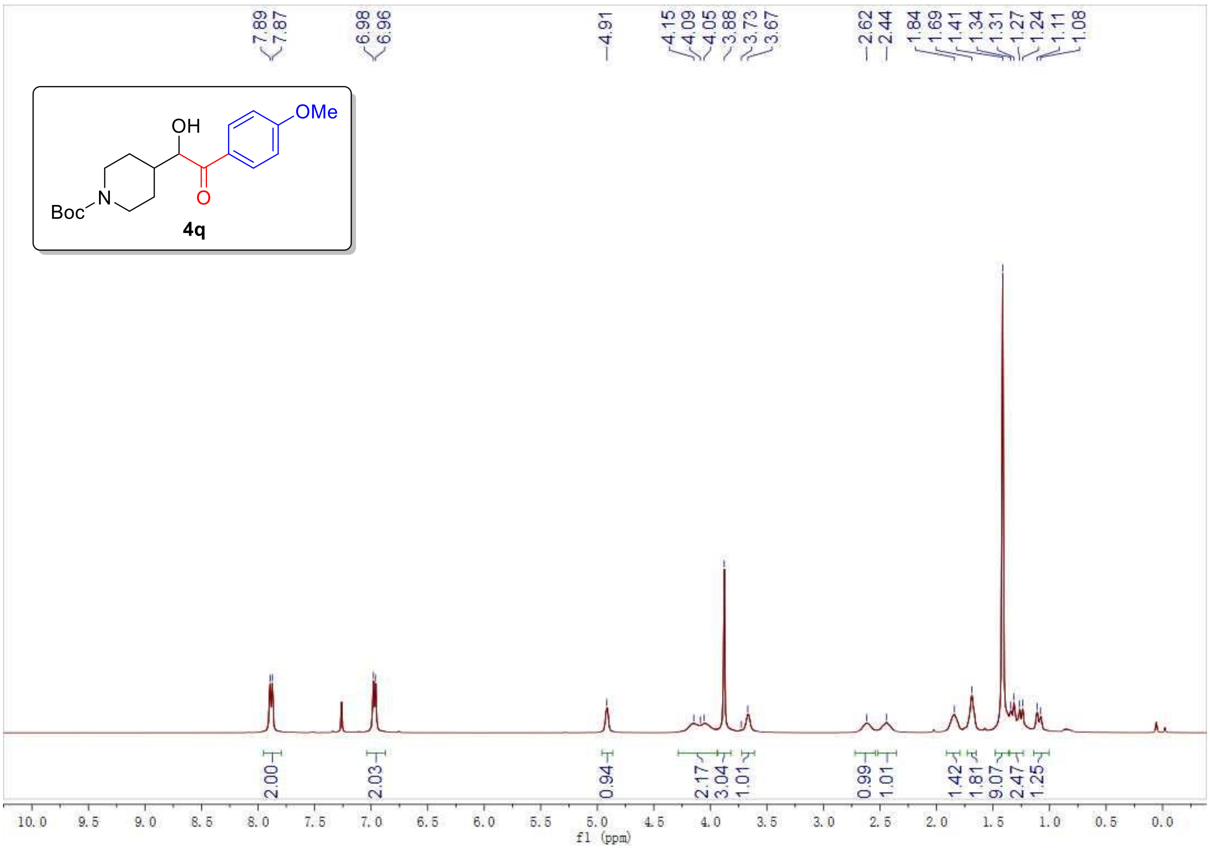

^{13}C NMR (101 MHz, $CDCl_3$) spectrum of 4q

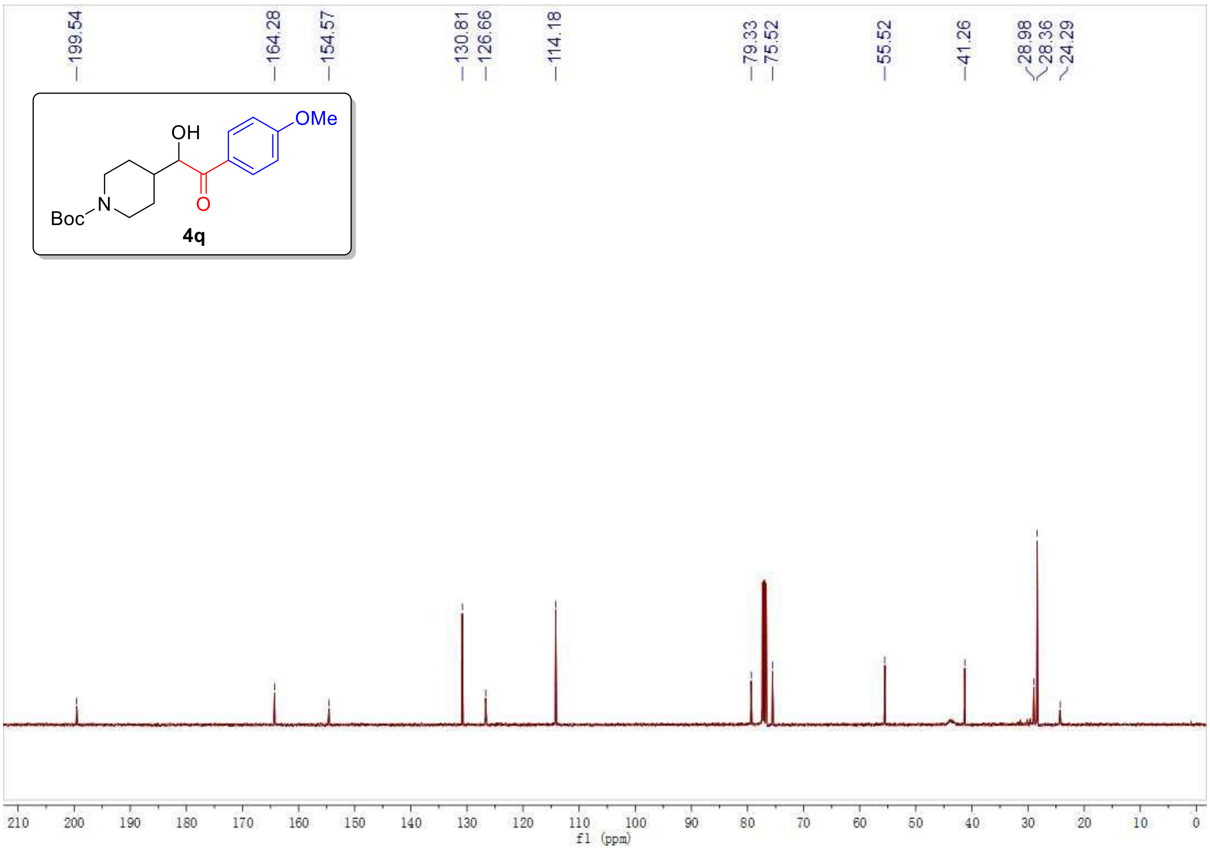

^{1}H NMR (400 MHz, $CDCl_3$) spectrum of 4r

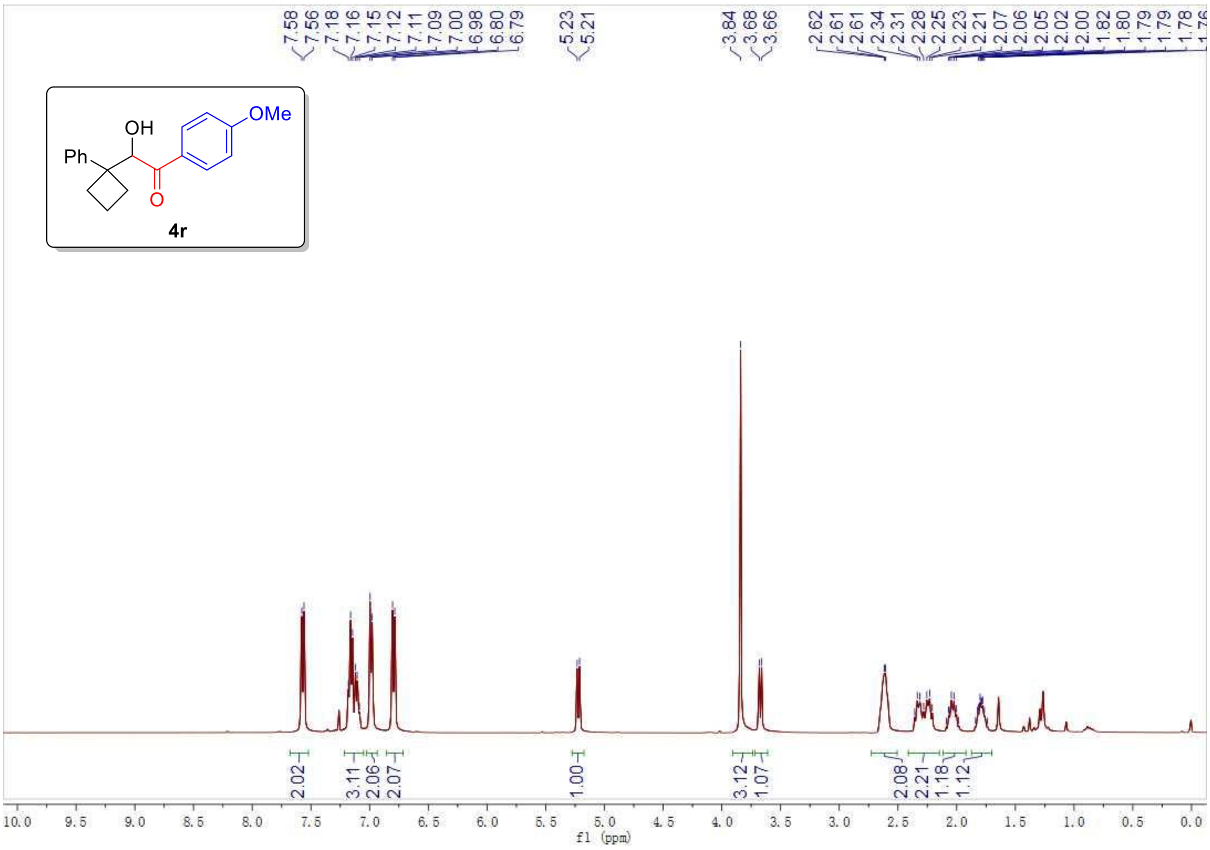

^{13}C NMR (101 MHz, $CDCl_3$) spectrum of 4r

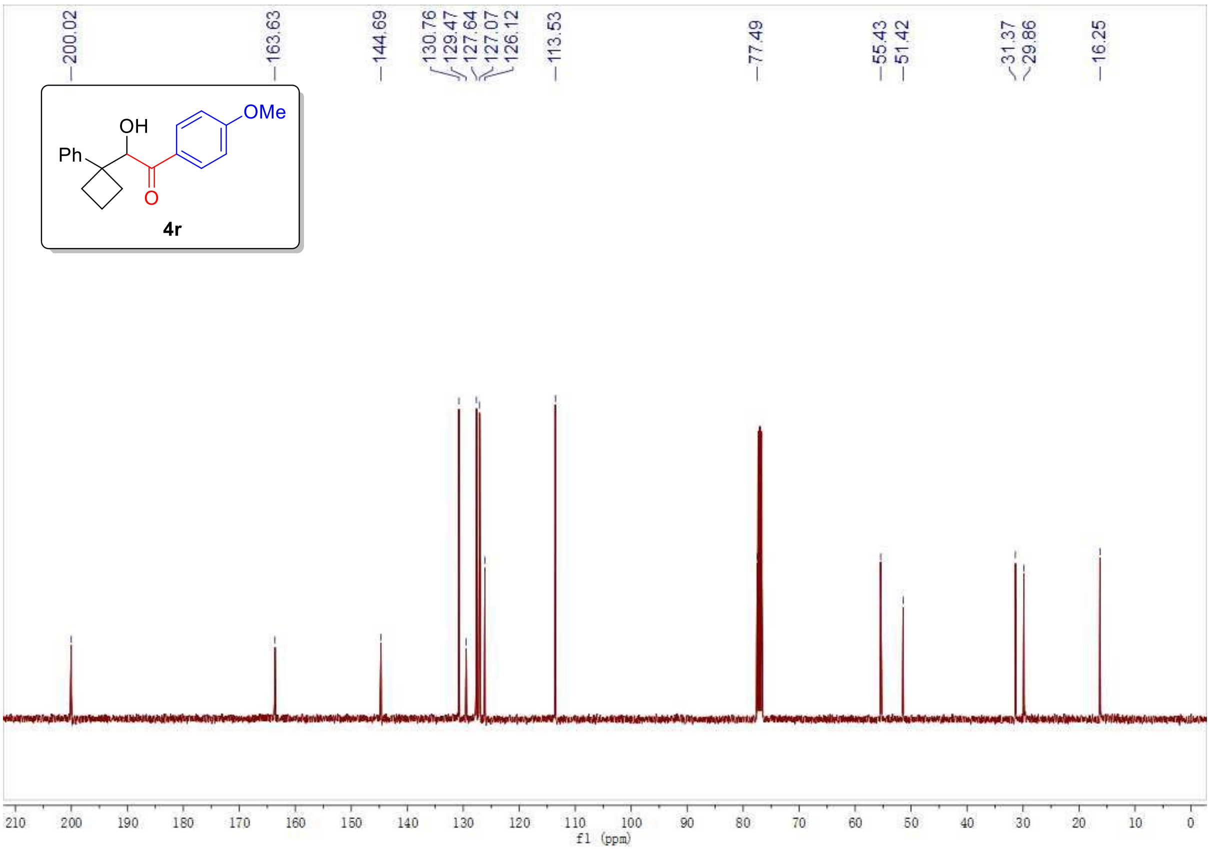

^{1}H NMR (400 MHz, $CDCl_3$) spectrum of 4s

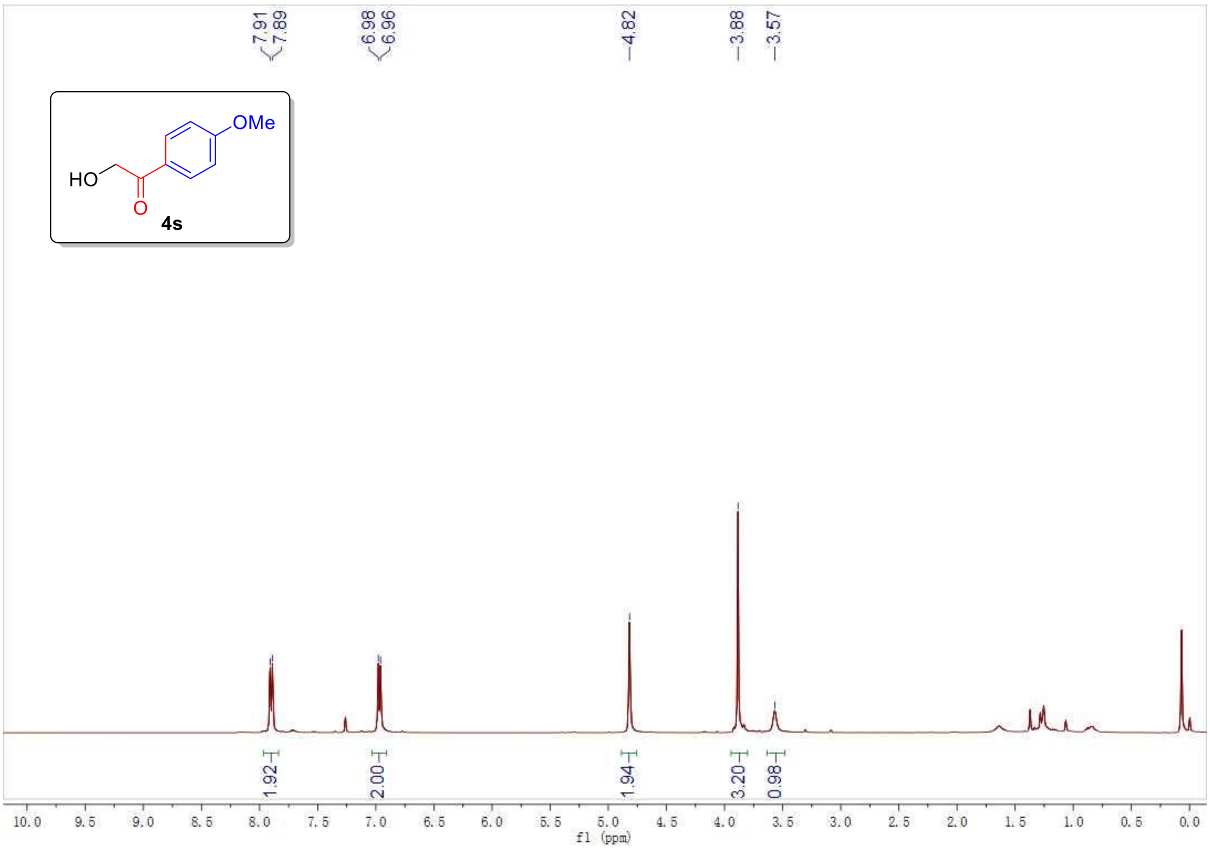

^{13}C NMR (101 MHz, $CDCl_3$) spectrum of 4s

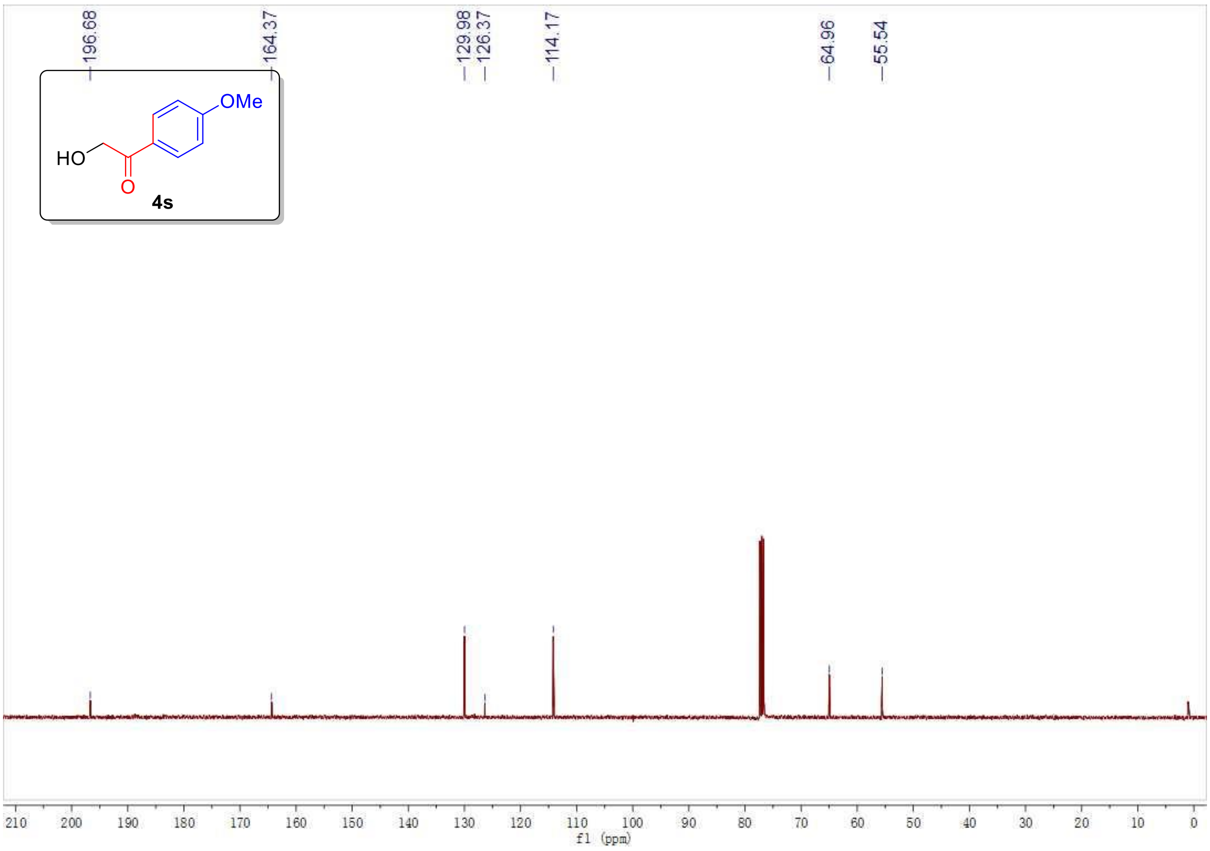

^{1}H NMR (400 MHz, $CDCl_3$) spectrum of 4t

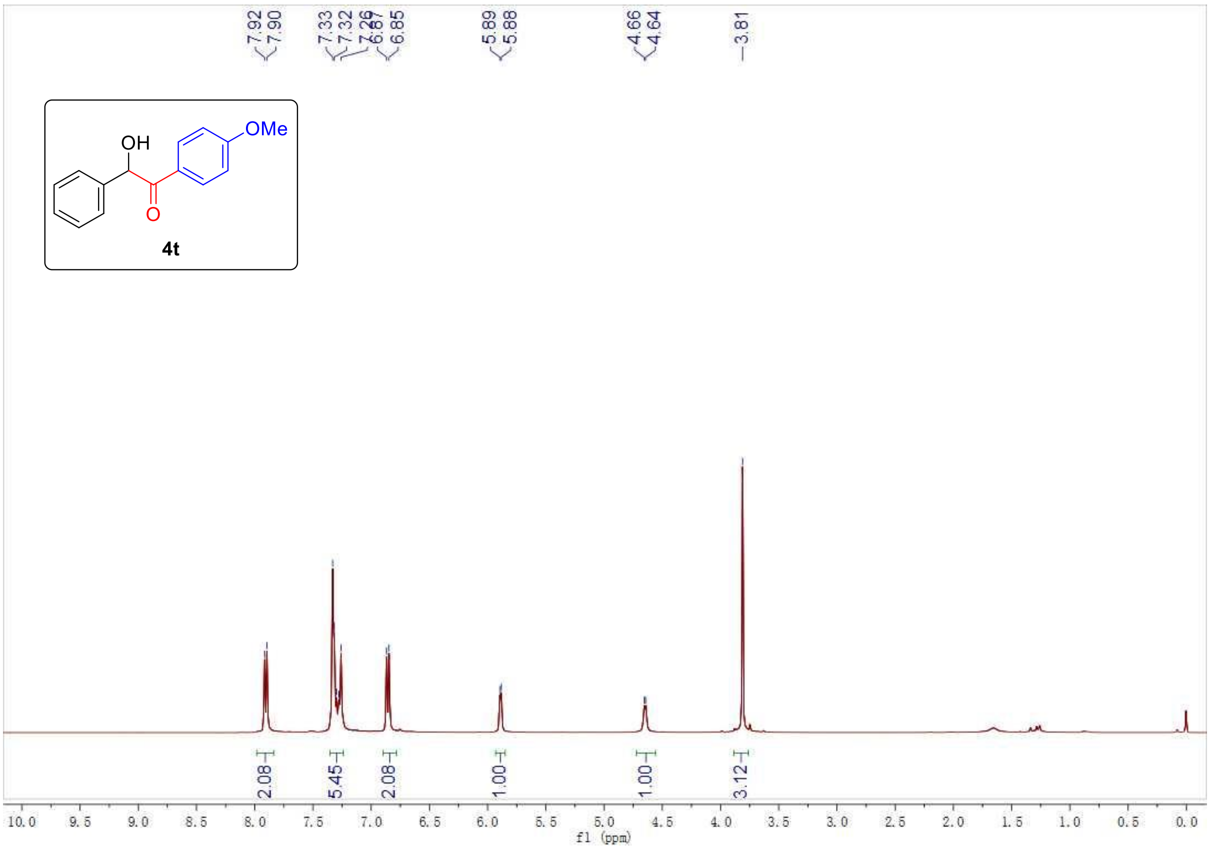

^{13}C NMR (101 MHz, $CDCl_3$) spectrum of 4t

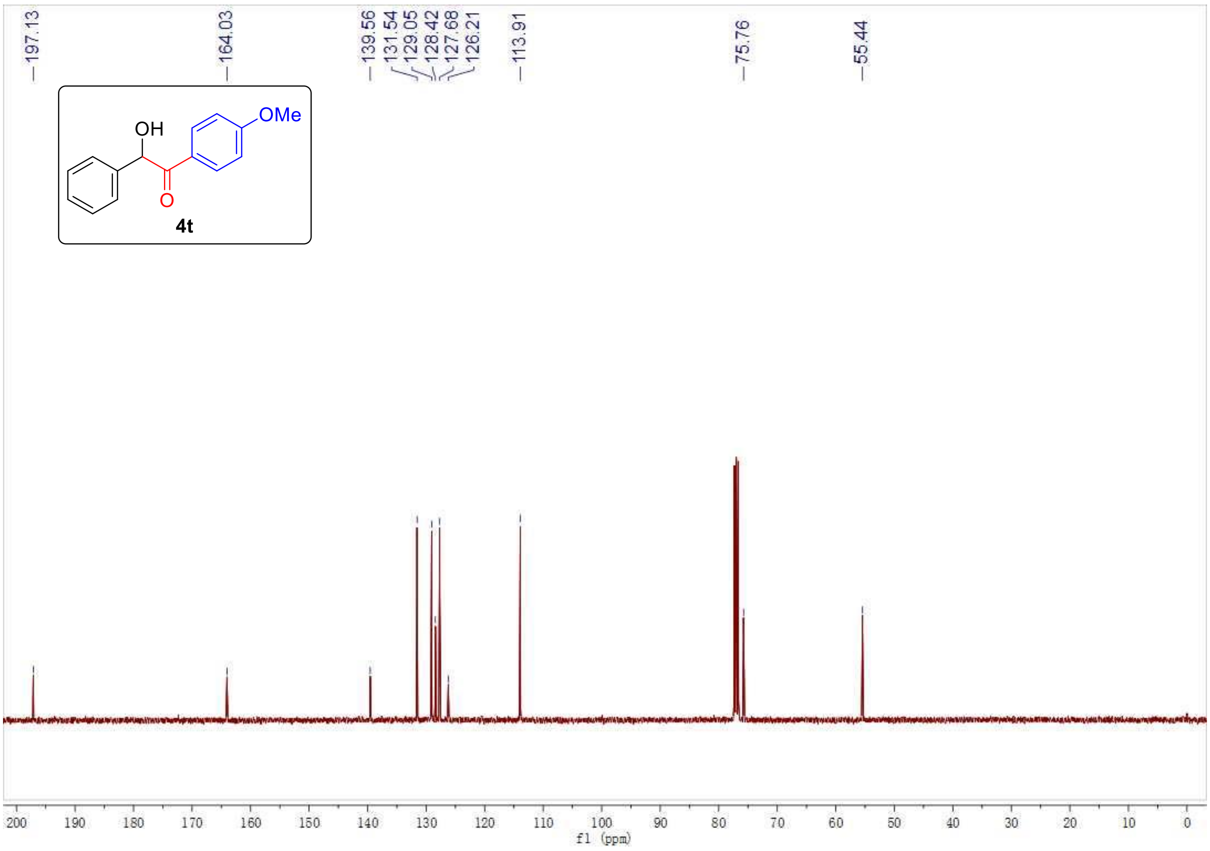

^{1}H NMR (400 MHz, $CDCl_3$) spectrum of 4u

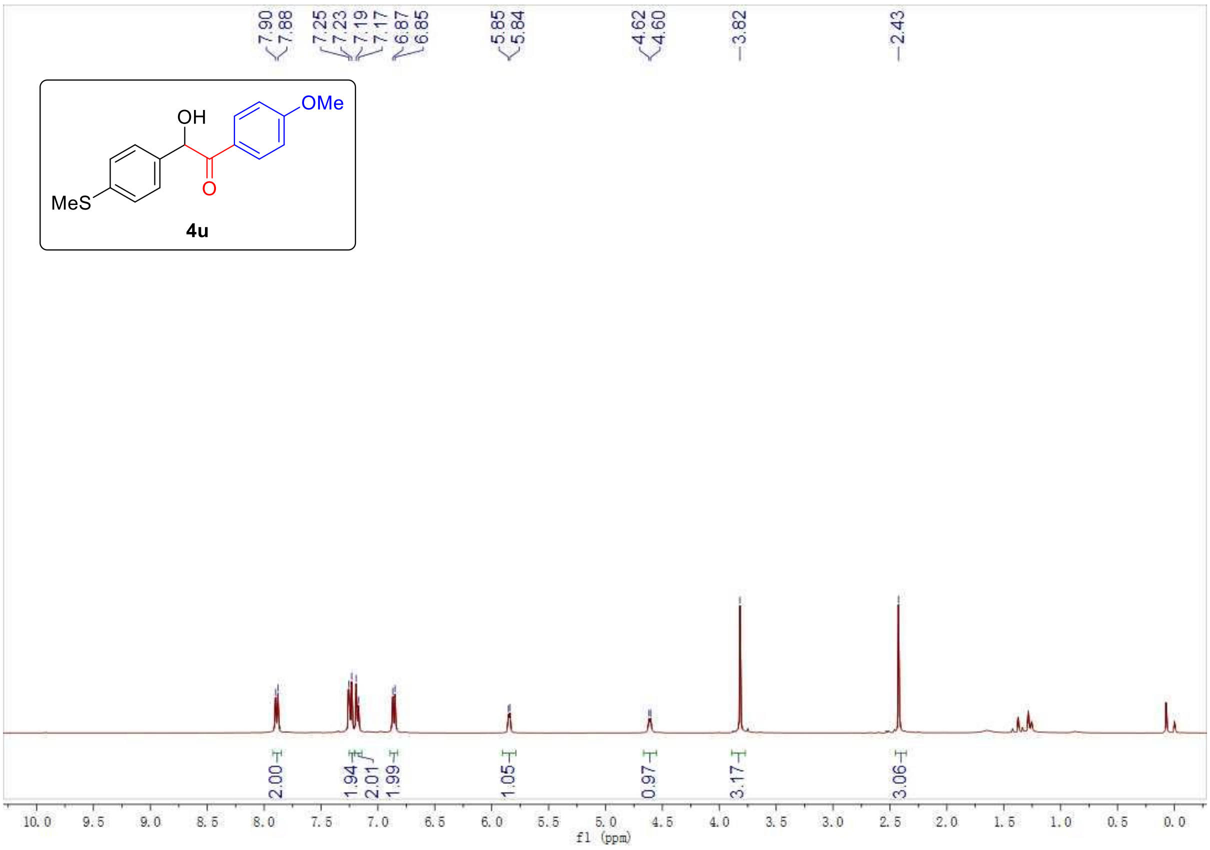

^{13}C NMR (101 MHz, $CDCl_3$) spectrum of 4u

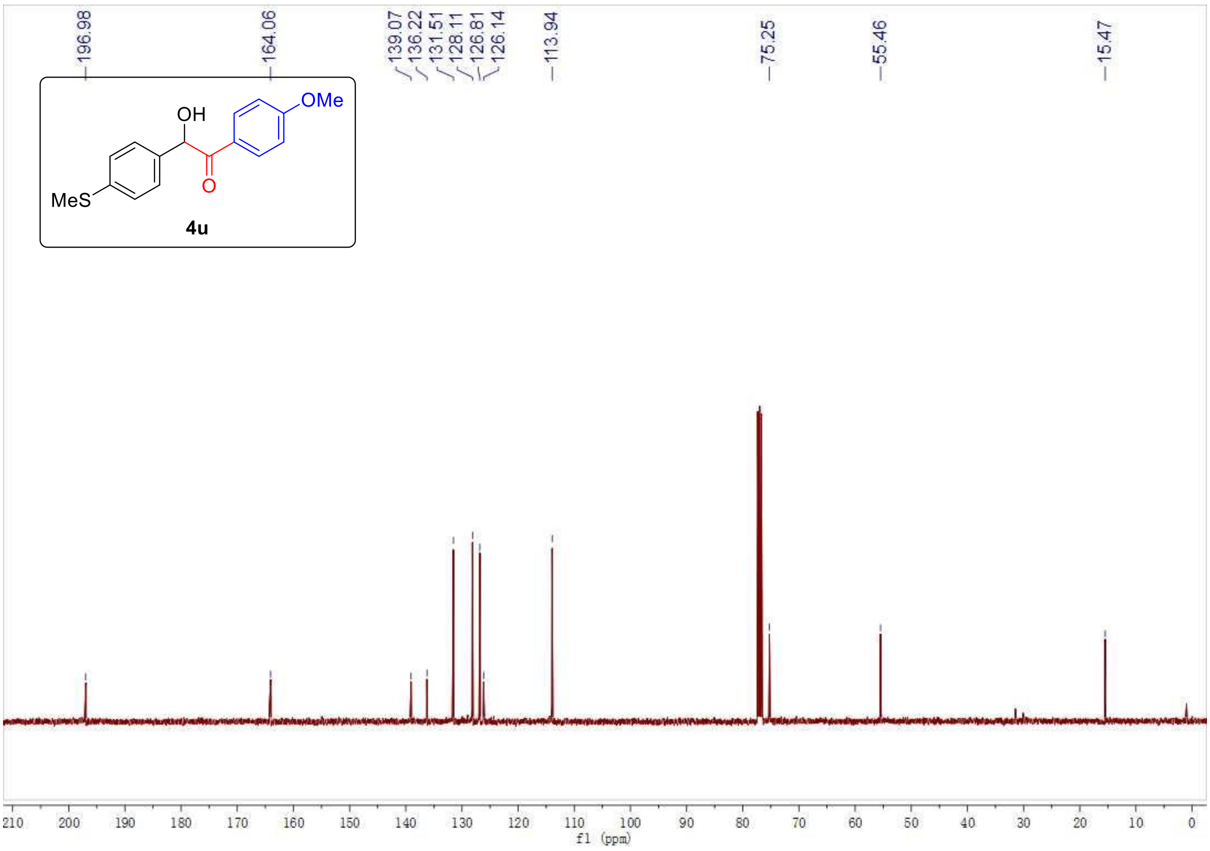

^{1}H NMR (400 MHz, $CDCl_3$) spectrum of 4v

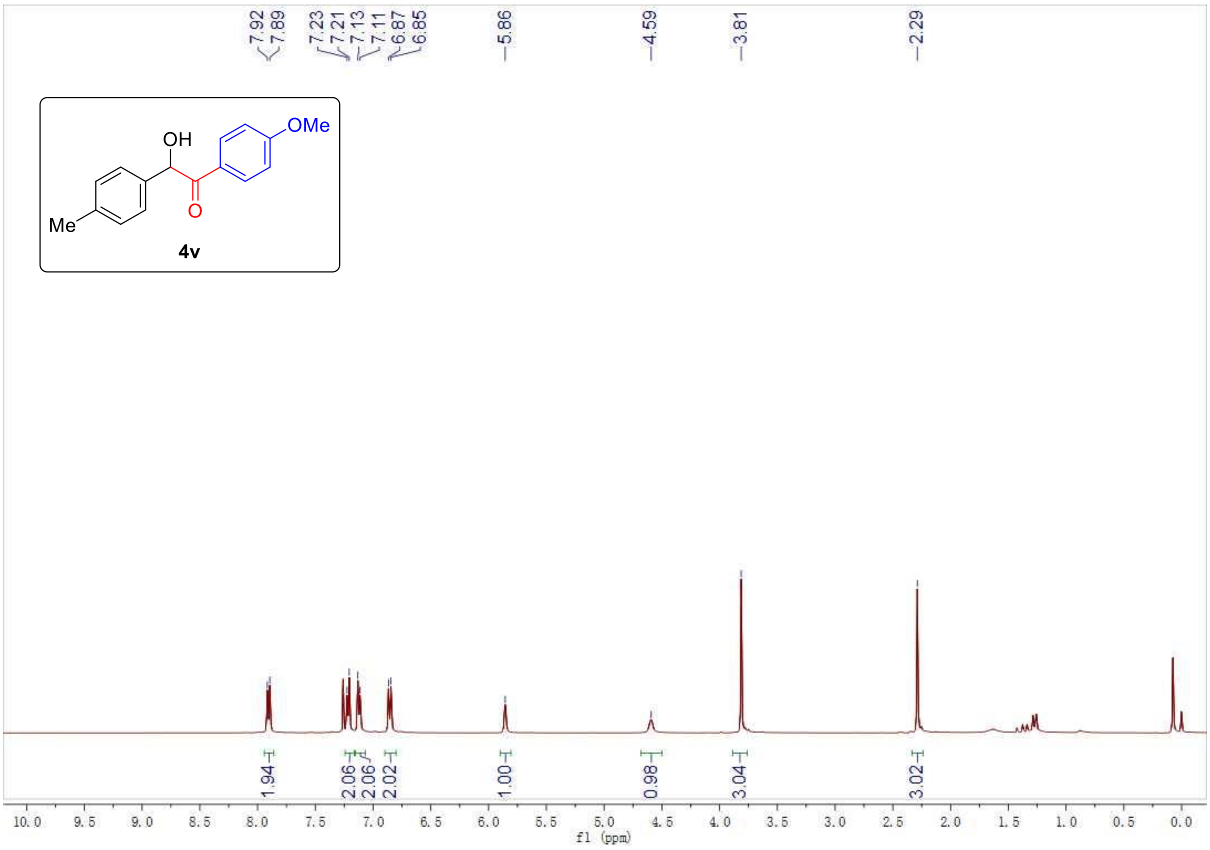

^{13}C NMR (101 MHz, $CDCl_3$) spectrum of 4v

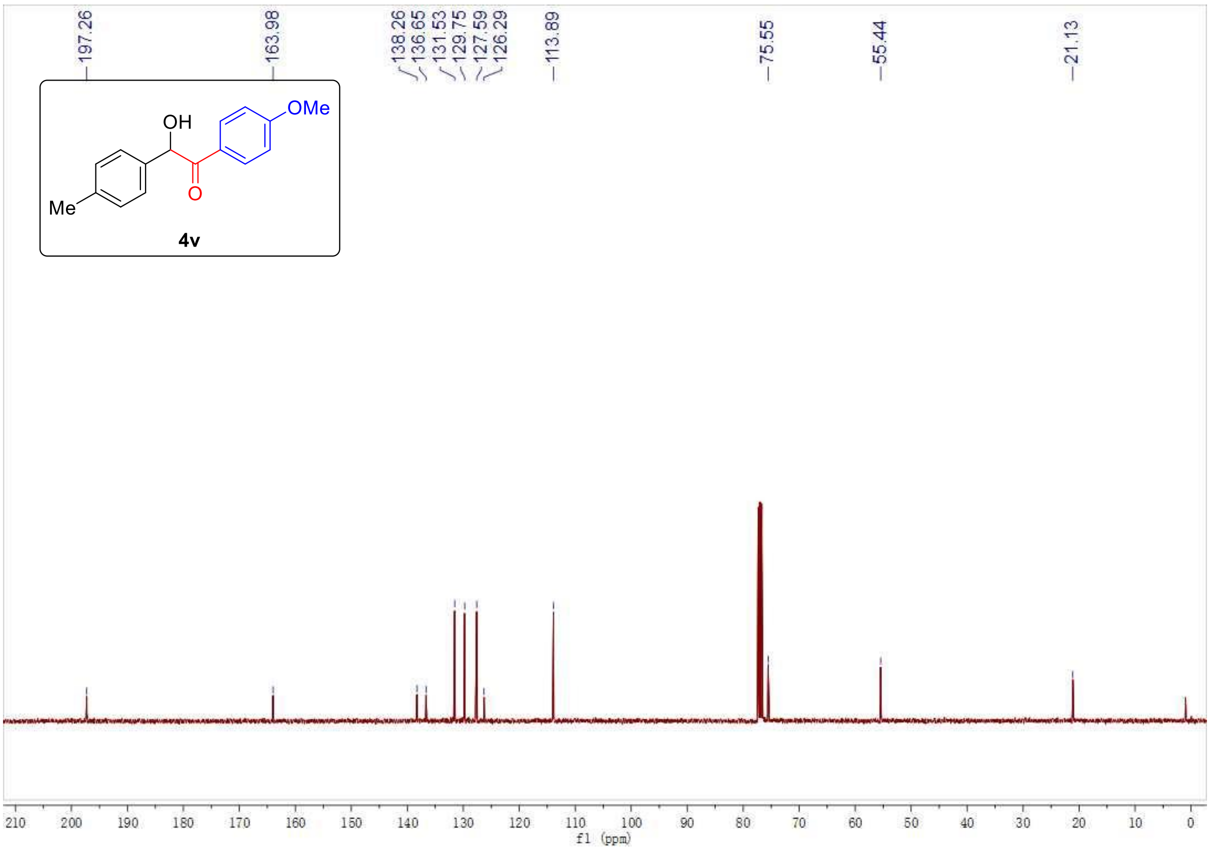

^{1}H NMR (400 MHz, $CDCl_3$) spectrum of 4w

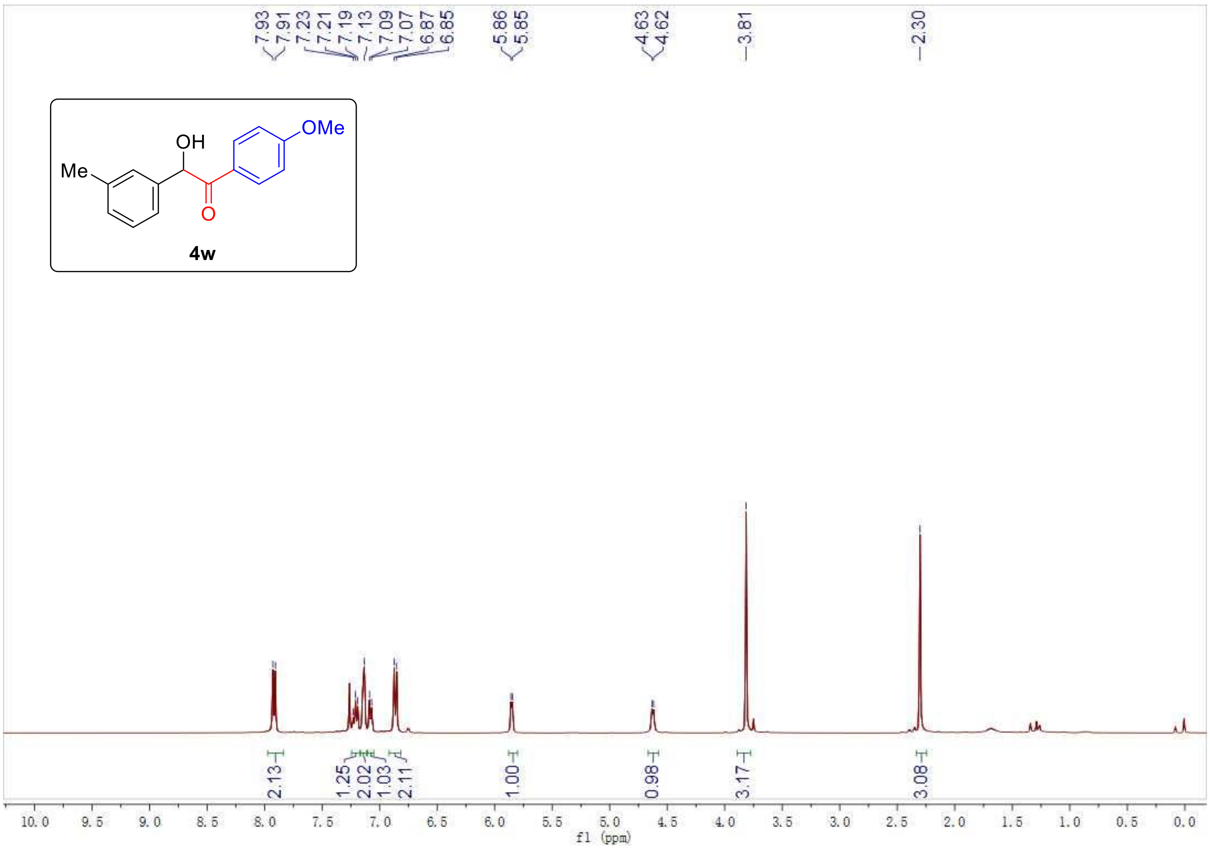

^{13}C NMR (101 MHz, $CDCl_3$) spectrum of 4w

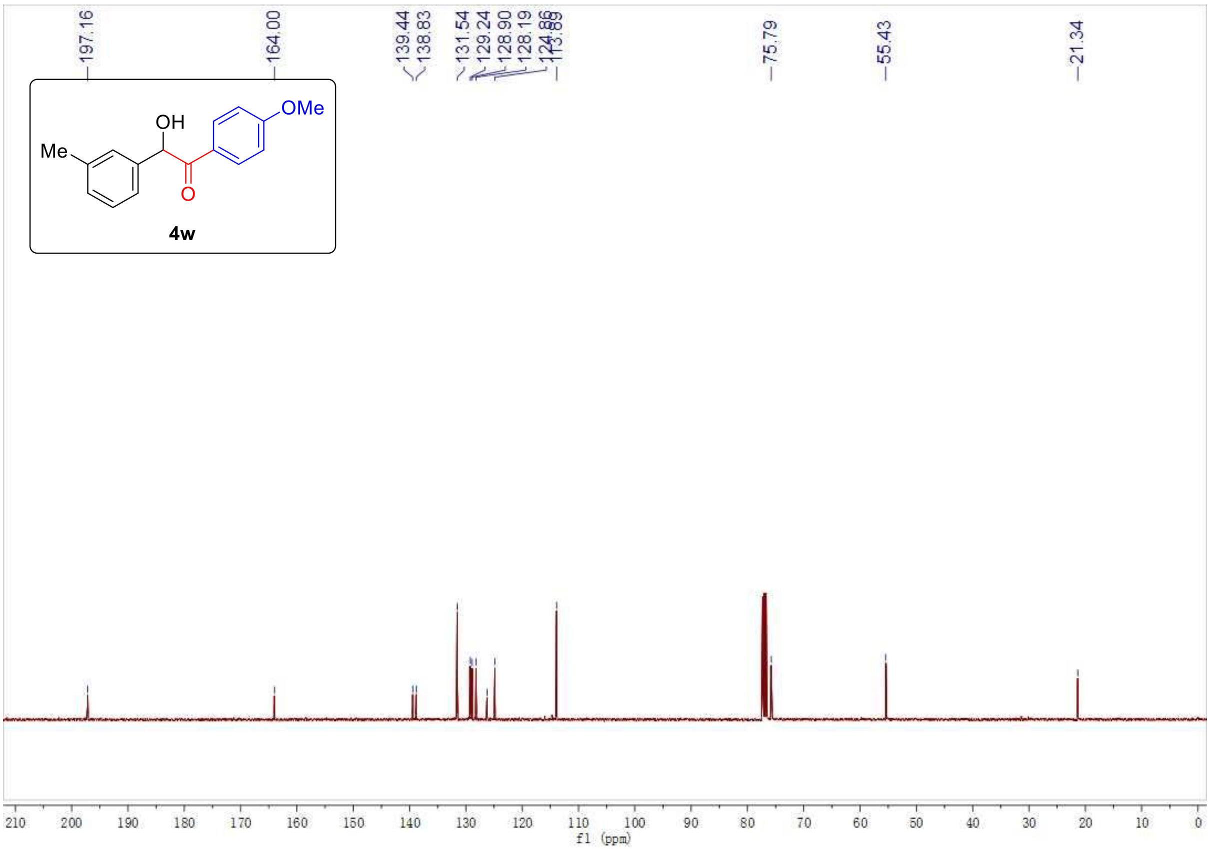

^{1}H NMR (400 MHz, $CDCl_3$) spectrum of 4x

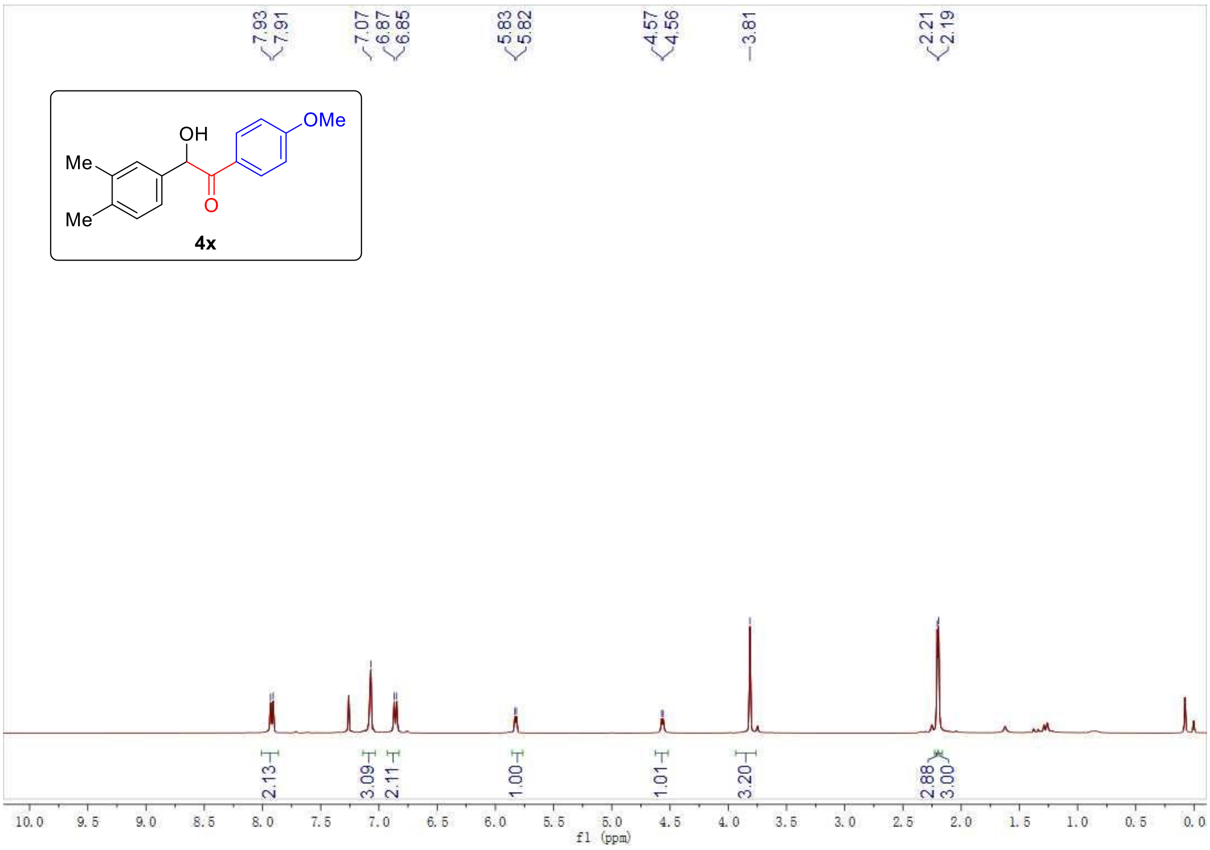

^{13}C NMR (101 MHz, $CDCl_3$) spectrum of 4x

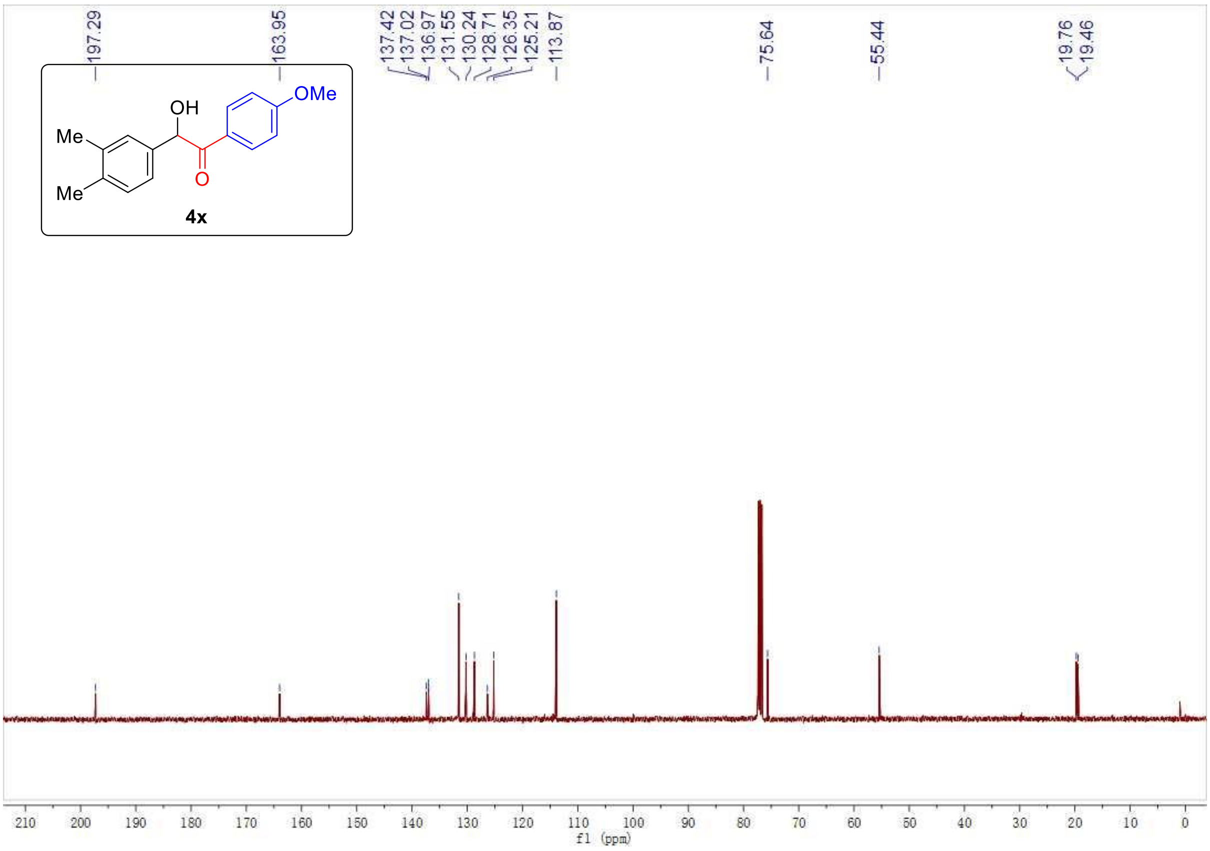

^{1}H NMR (400 MHz, $CDCl_3$) spectrum of 4y

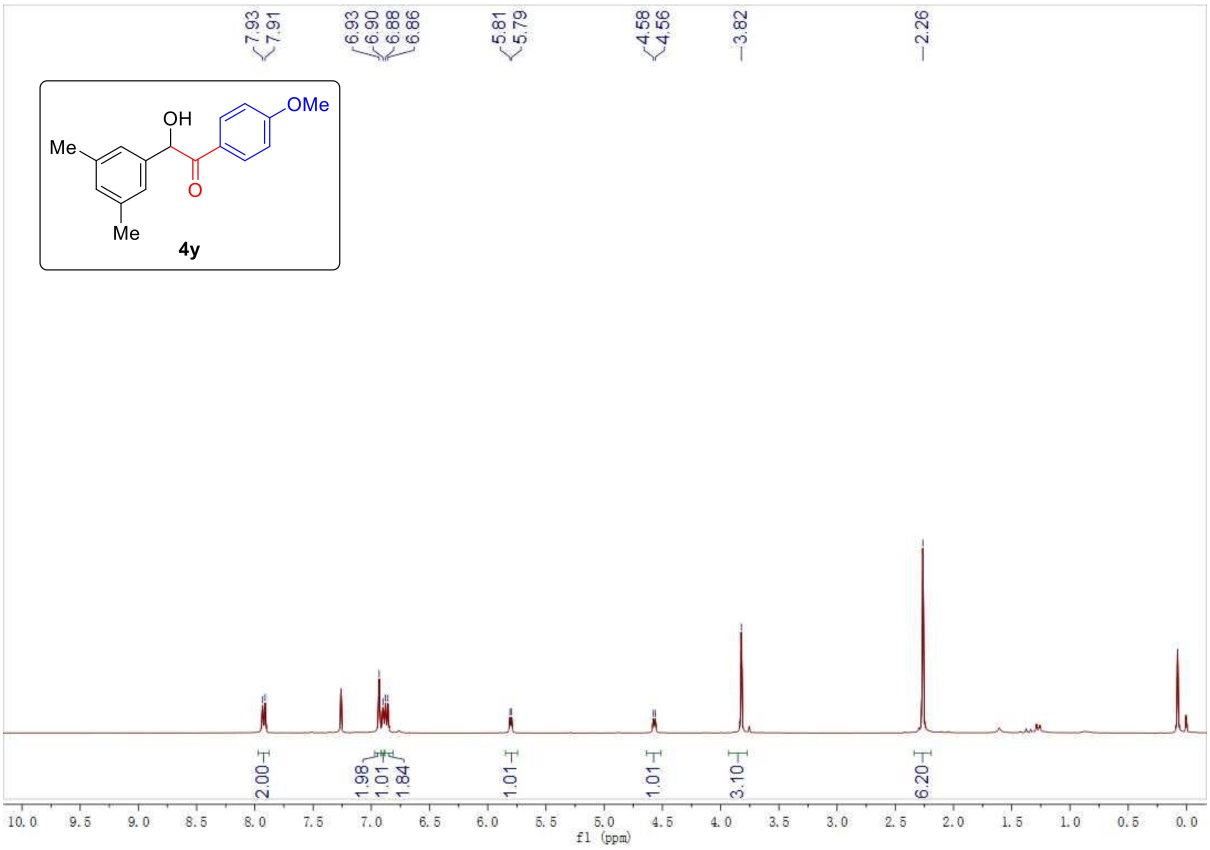

^{13}C NMR (101 MHz, $CDCl_3$) spectrum of 4y

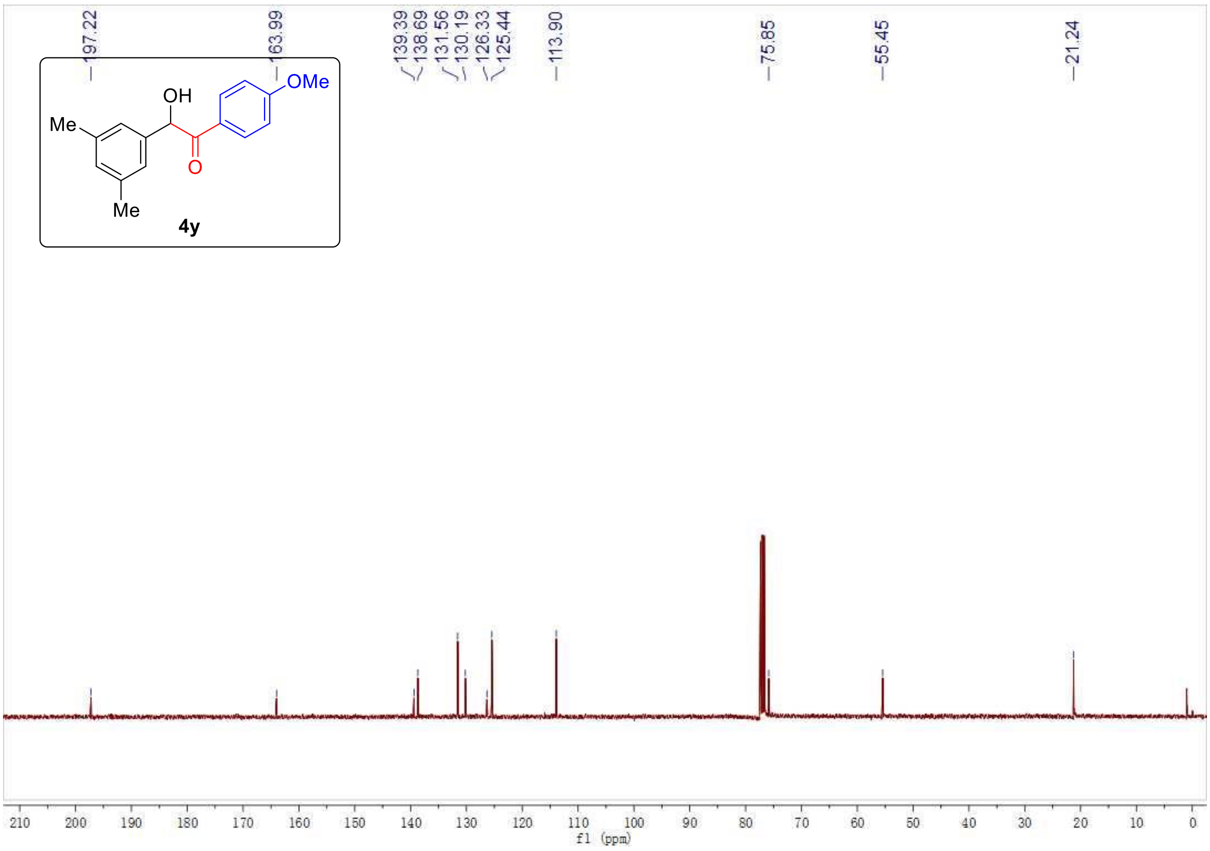

^{1}H NMR (400 MHz, $CDCl_3$) spectrum of 4z

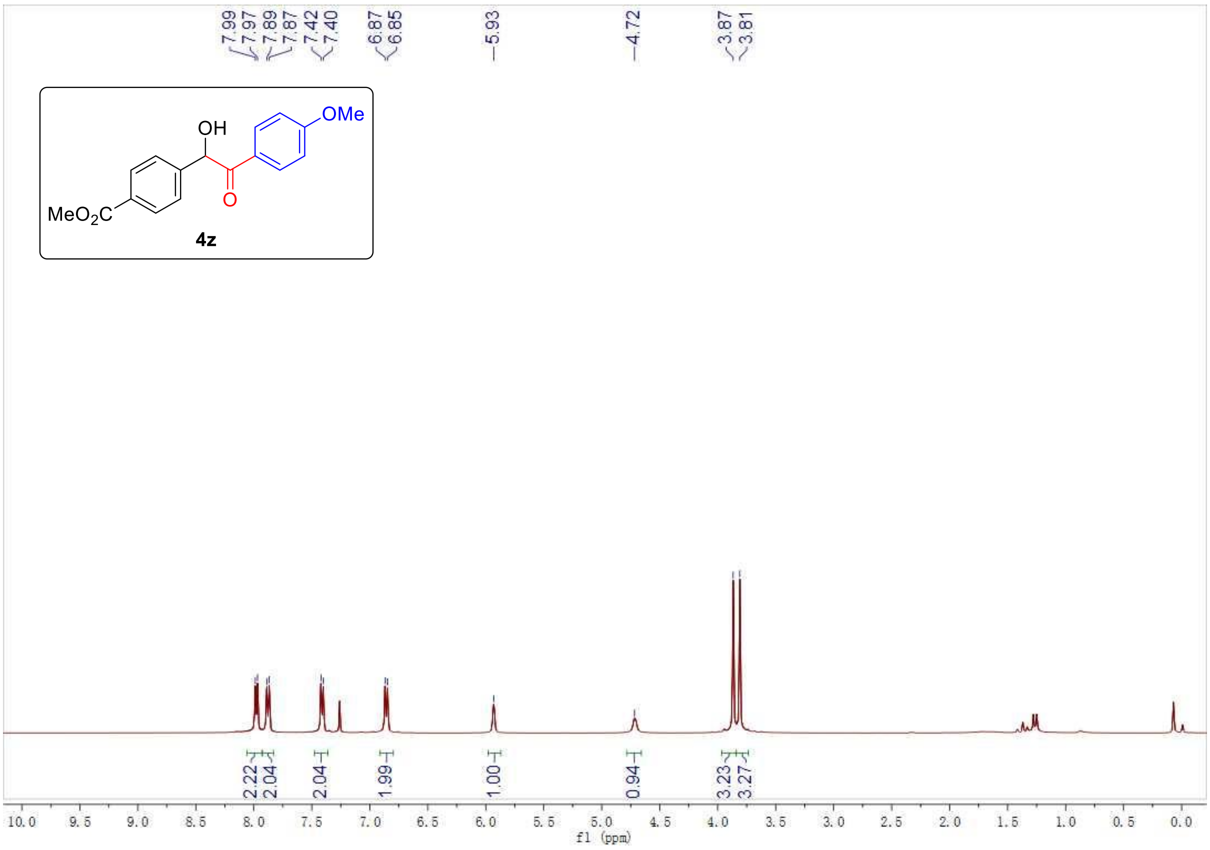

^{13}C NMR (101 MHz, $CDCl_3$) spectrum of 4z

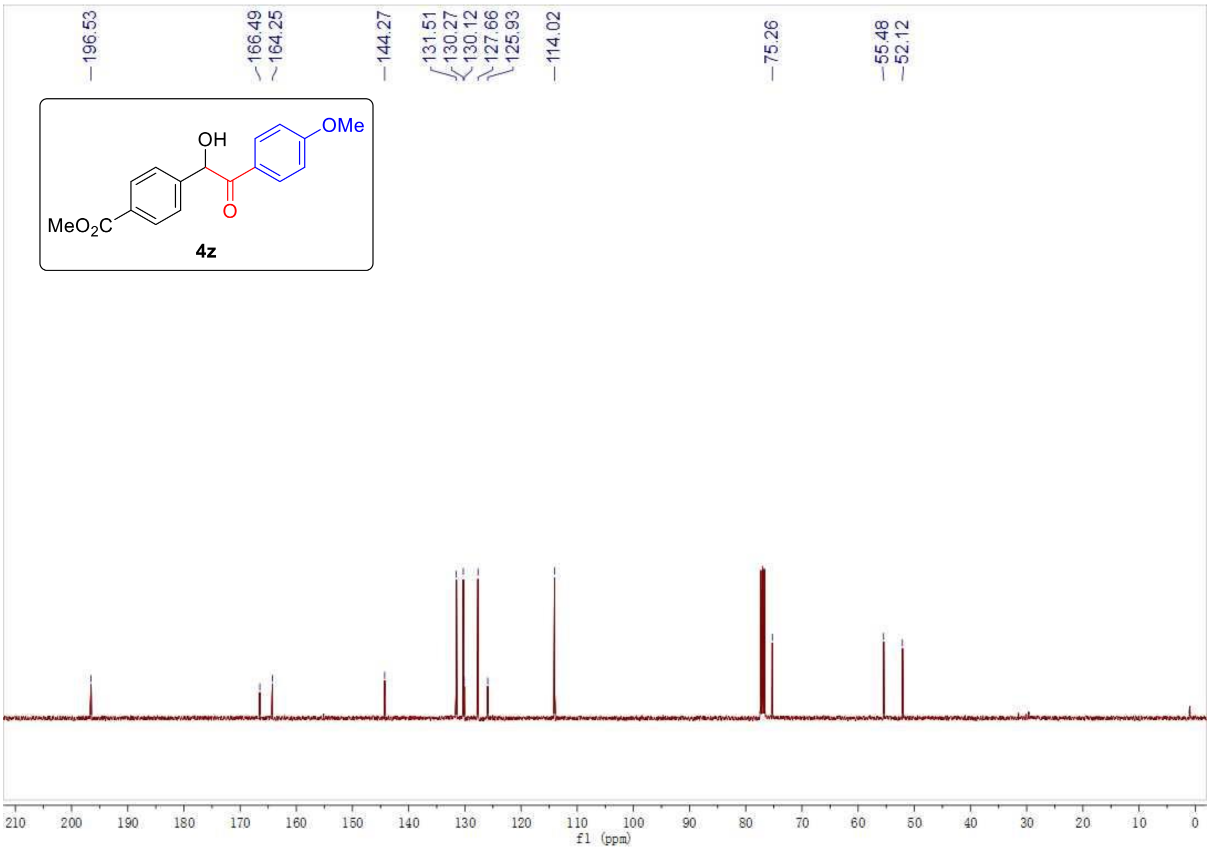

^{1}H NMR (400 MHz, $CDCl_3$) spectrum of 4aa

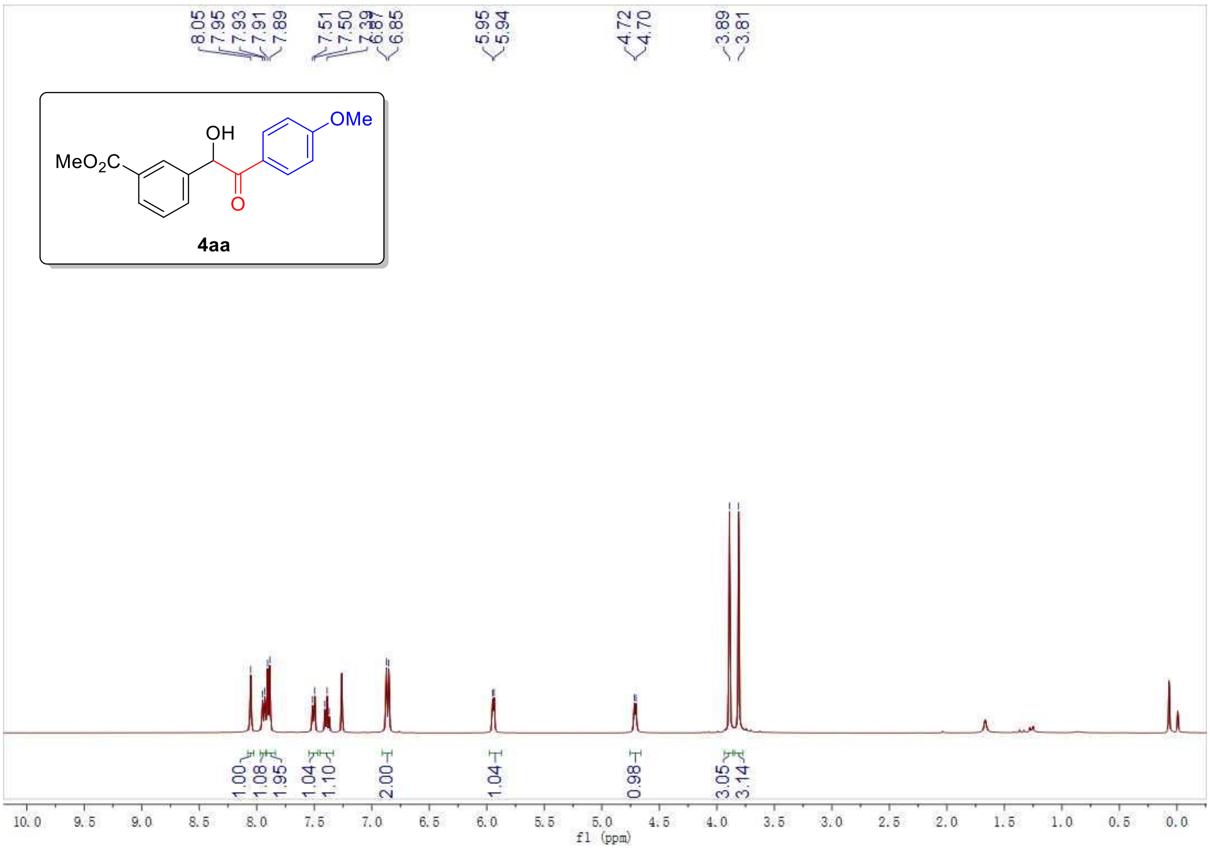

^{13}C NMR (101 MHz, $CDCl_3$) spectrum of 4aa

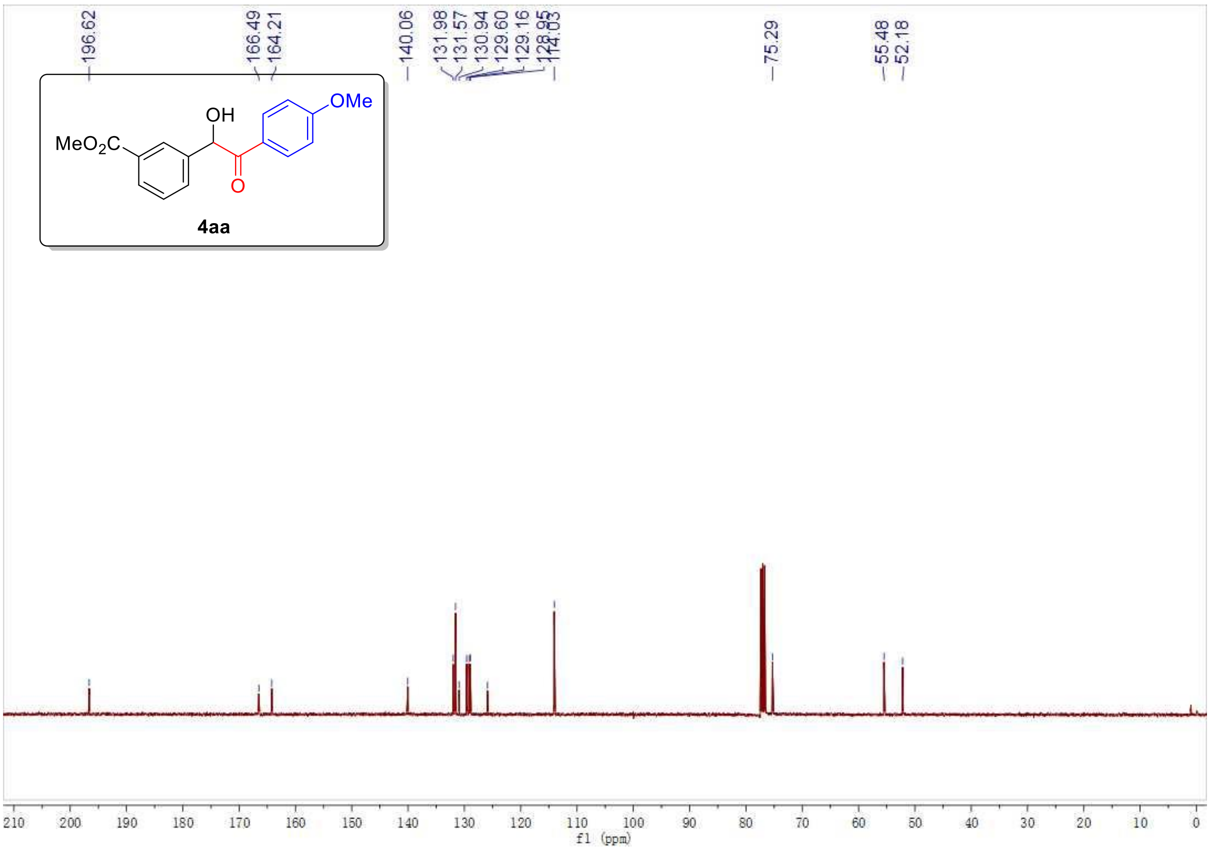

^{1}H NMR (400 MHz, CDCl$_3$) spectrum of 4ab

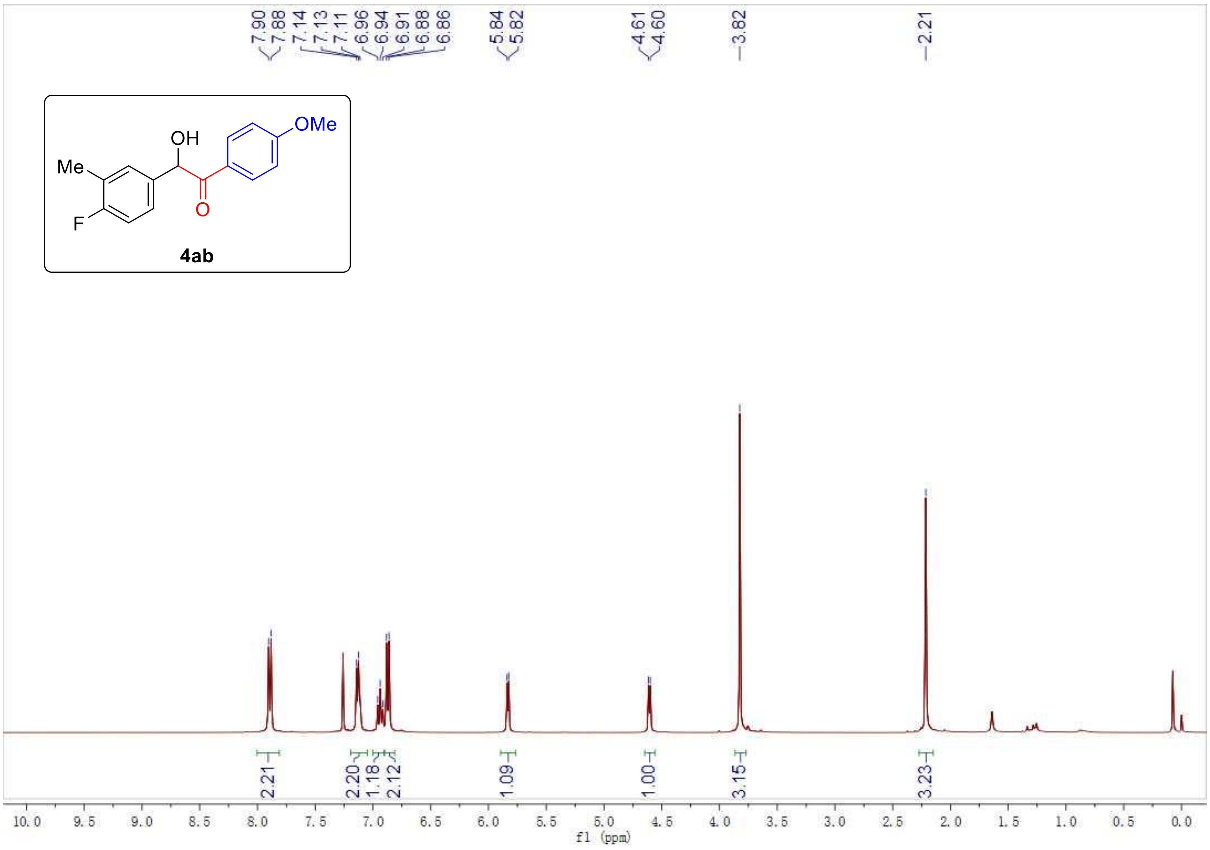

^{13}C NMR (101 MHz, CDCl$_3$) spectrum of 4ab

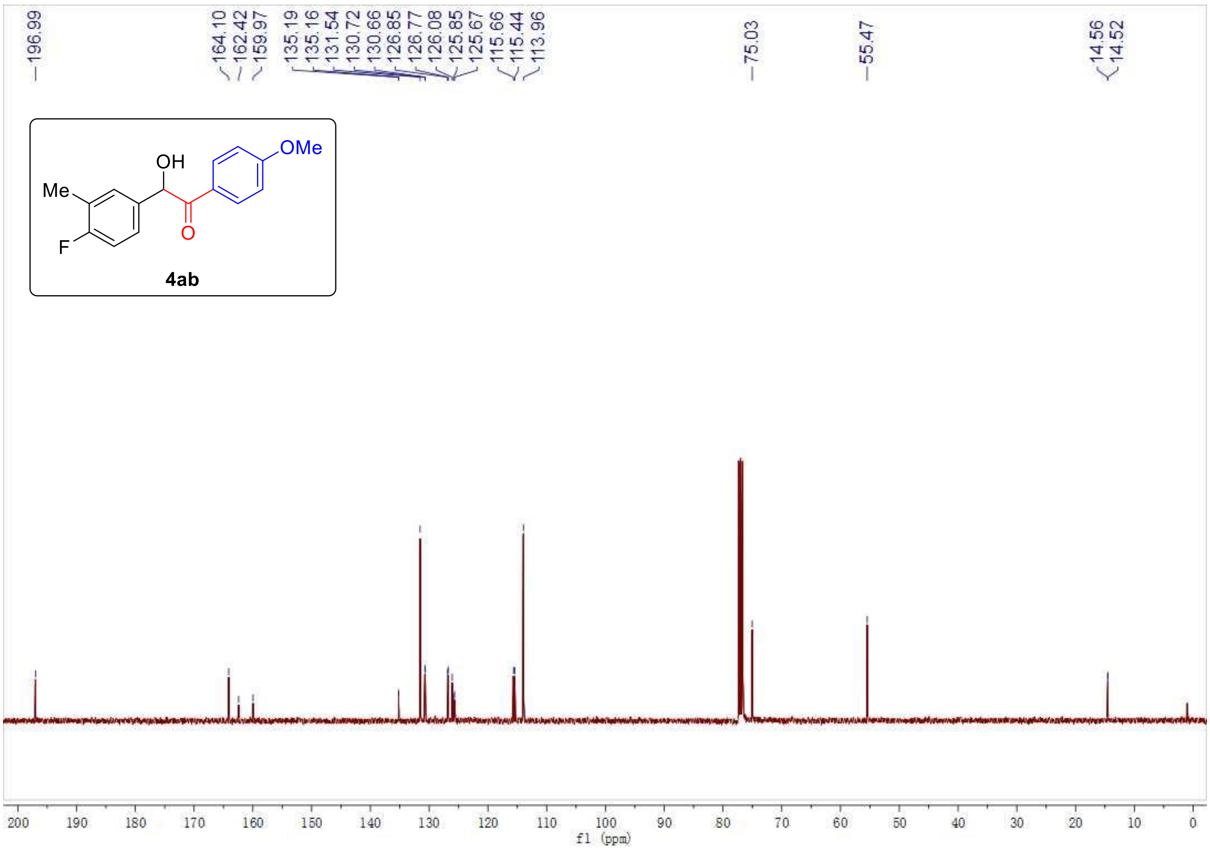

^{1}H NMR (400 MHz, $CDCl_3$) spectrum of 4ac

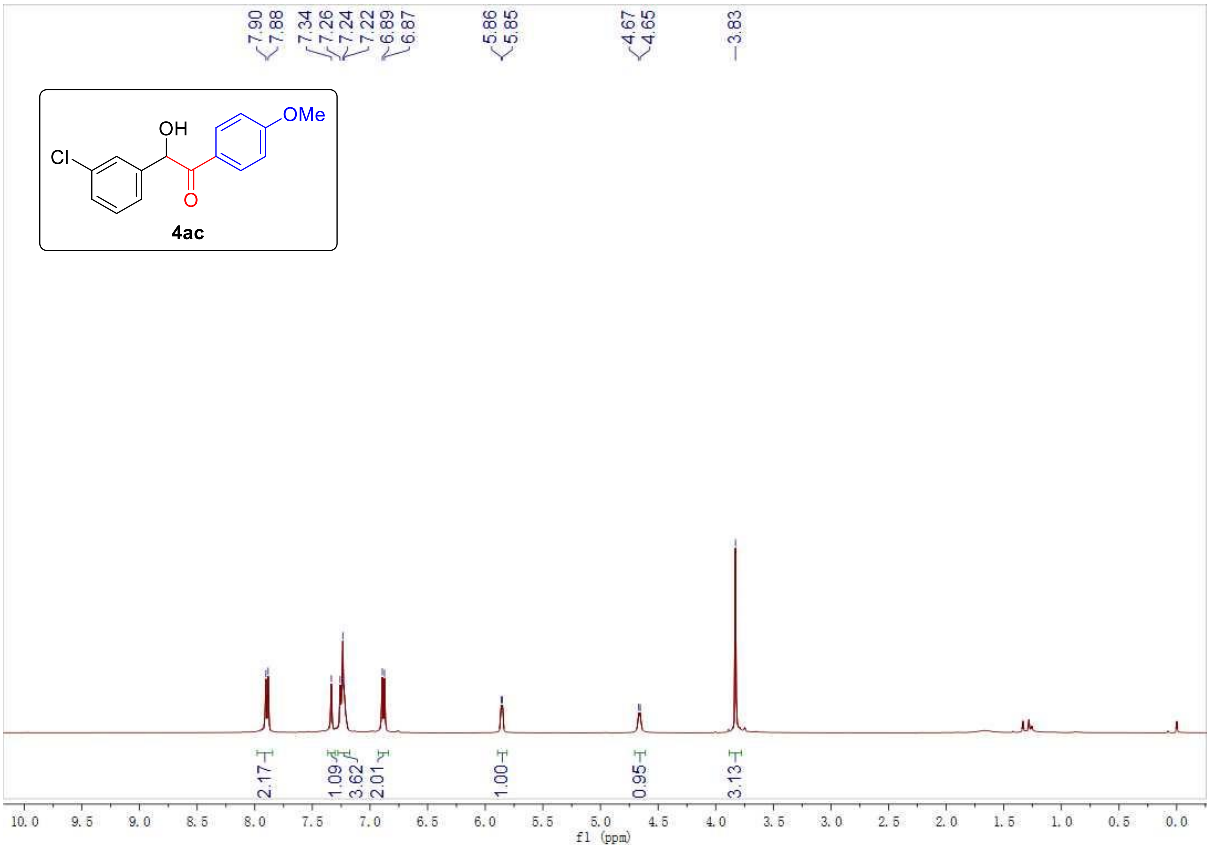

^{13}C NMR (101 MHz, $CDCl_3$) spectrum of 4ac

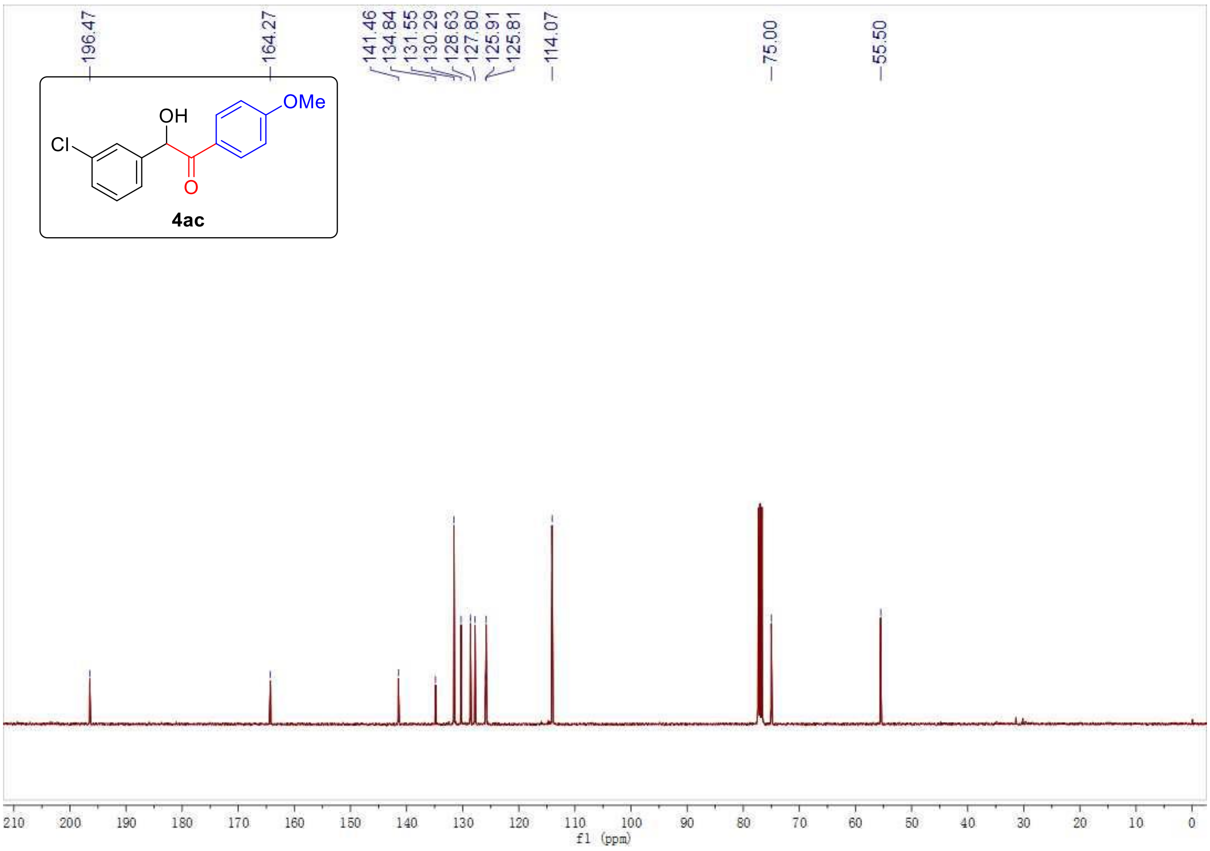

^{1}H NMR (400 MHz, $CDCl_3$) spectrum of 4ad

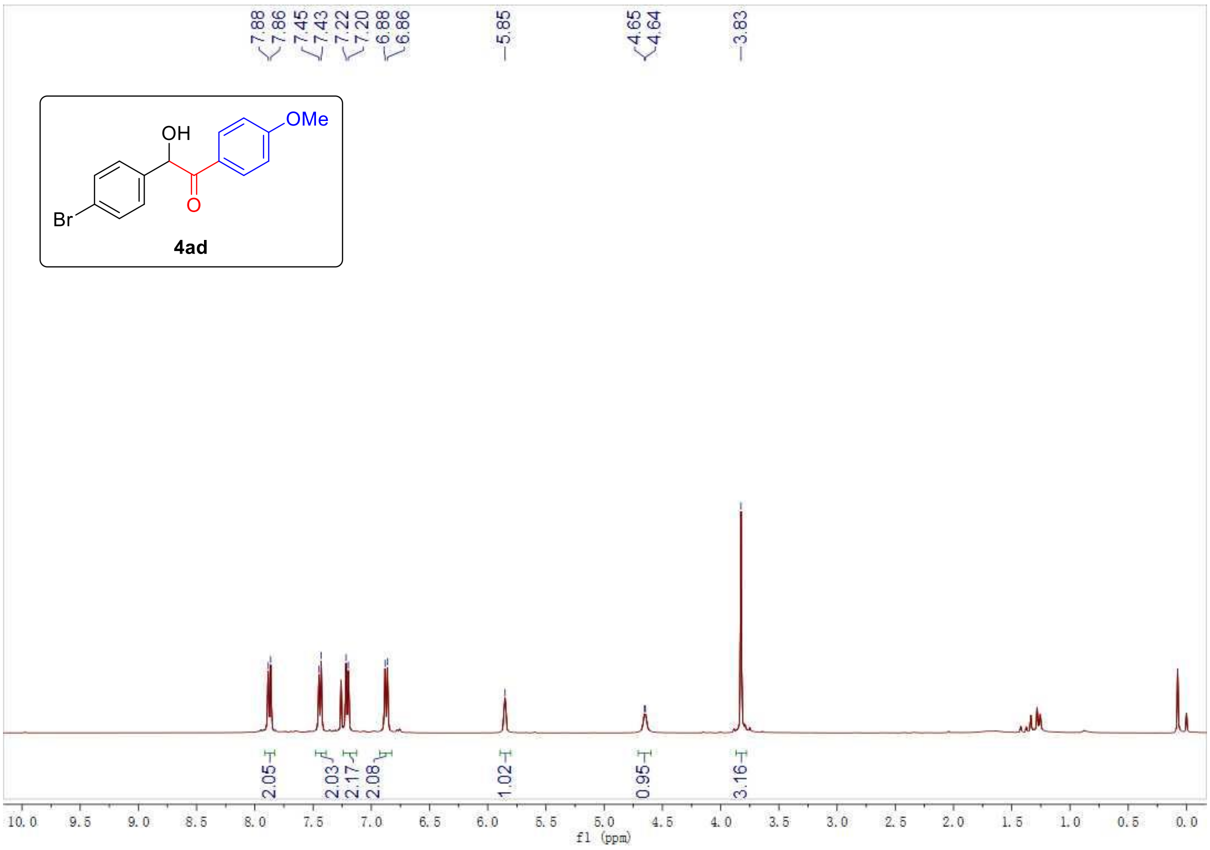

^{13}C NMR (101 MHz, $CDCl_3$) spectrum of 4ad

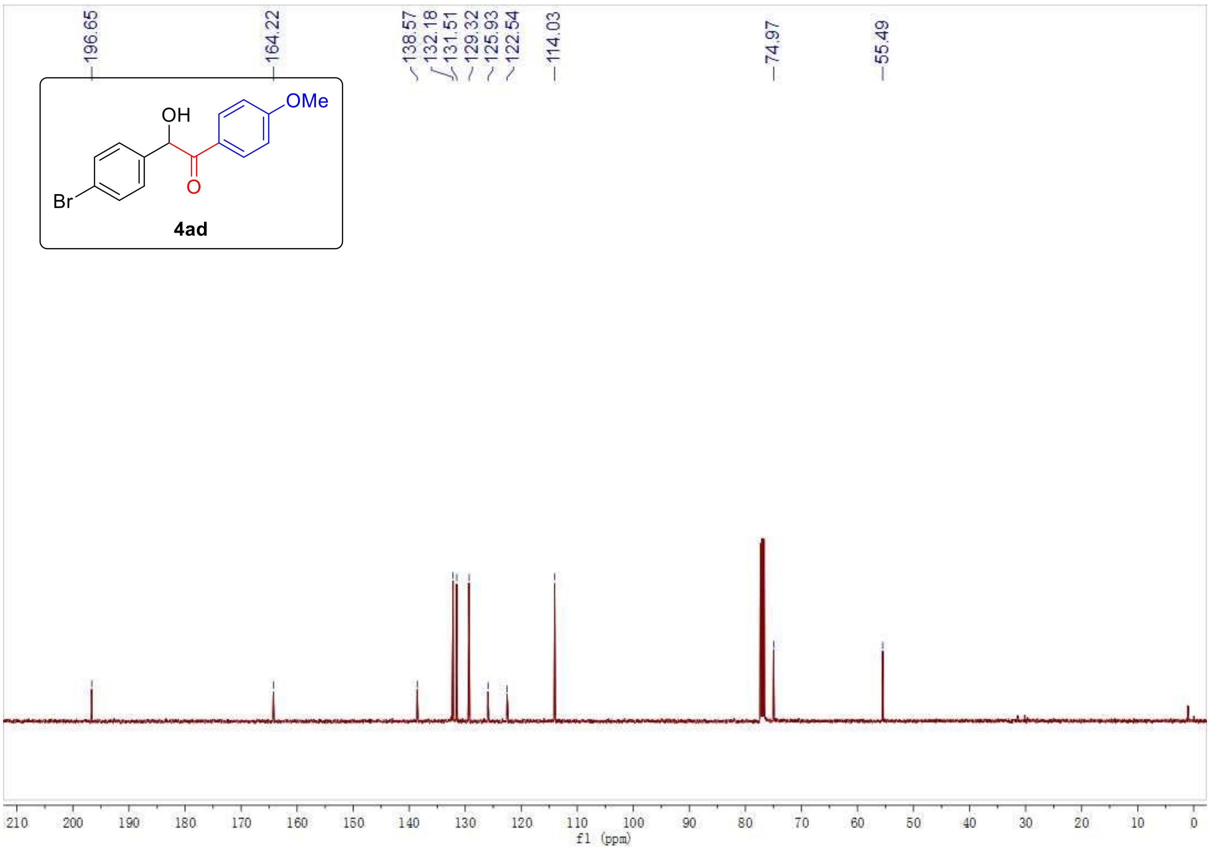

^{1}H NMR (400 MHz, $CDCl_3$) spectrum of 4ae

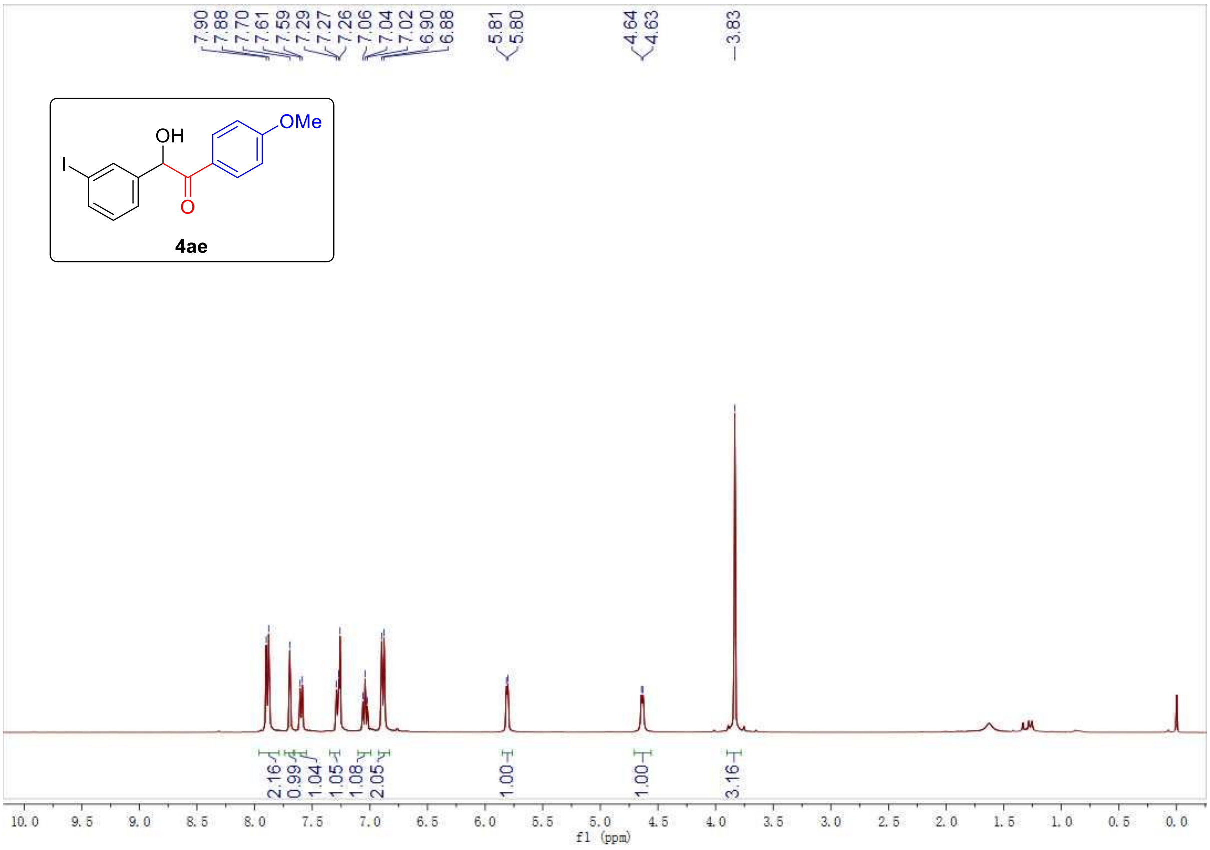

^{13}C NMR (101 MHz, $CDCl_3$) spectrum of 4ae

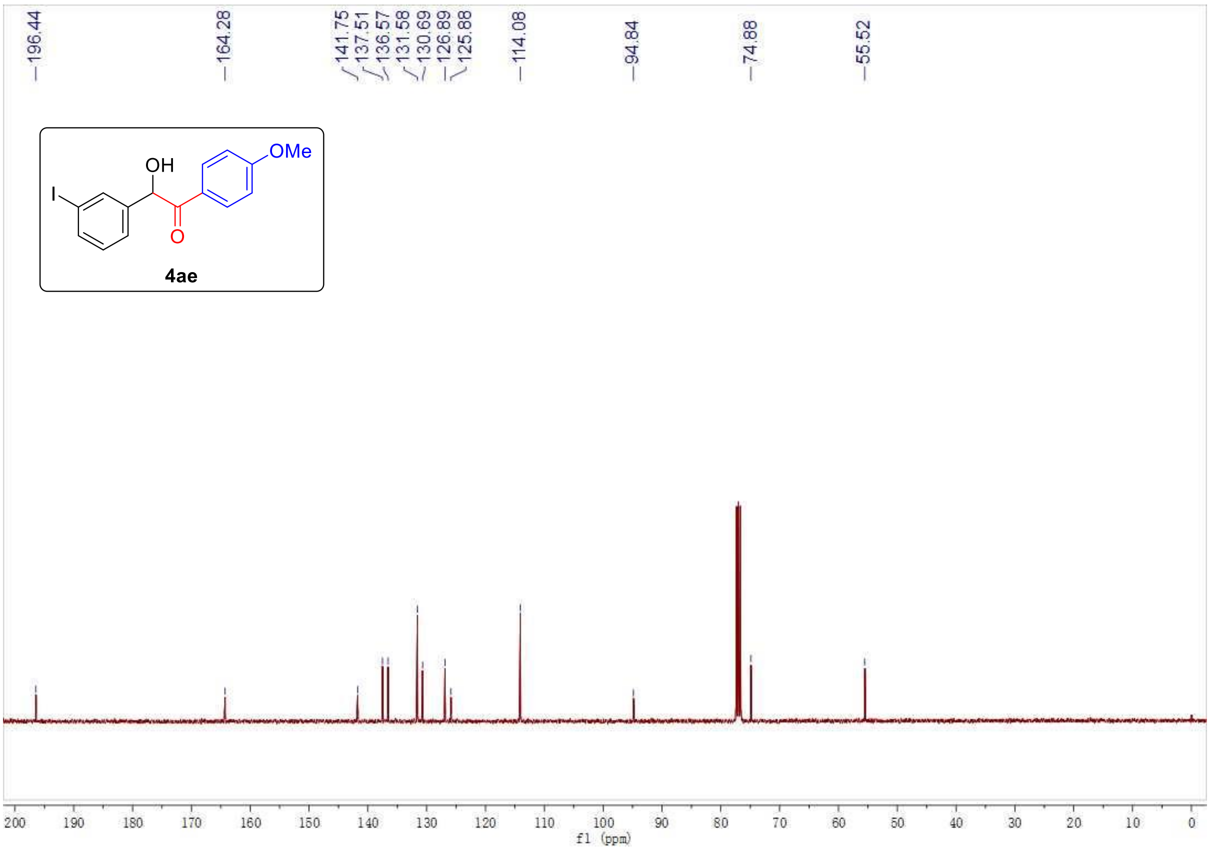

^{1}H NMR (400 MHz, $CDCl_3$) spectrum of 4af

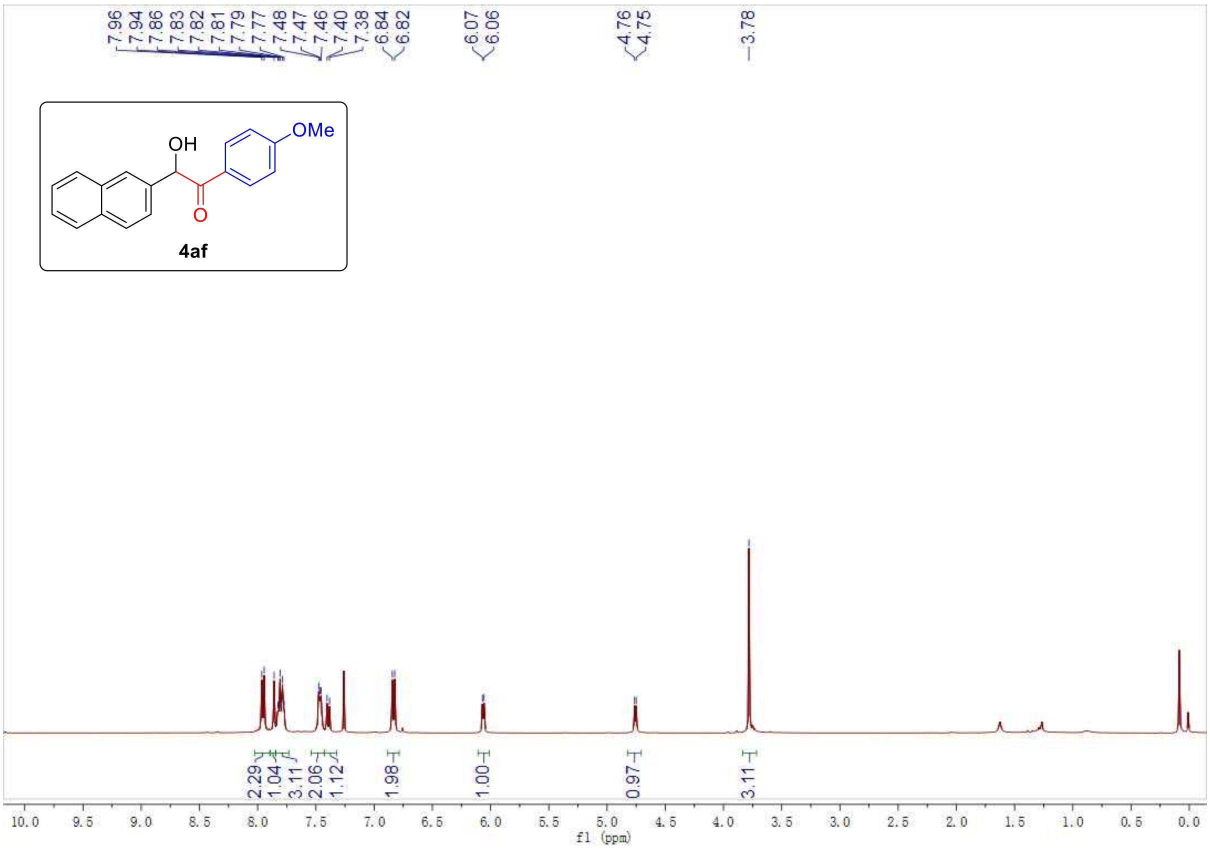

^{13}C NMR (101 MHz, $CDCl_3$) spectrum of 4af

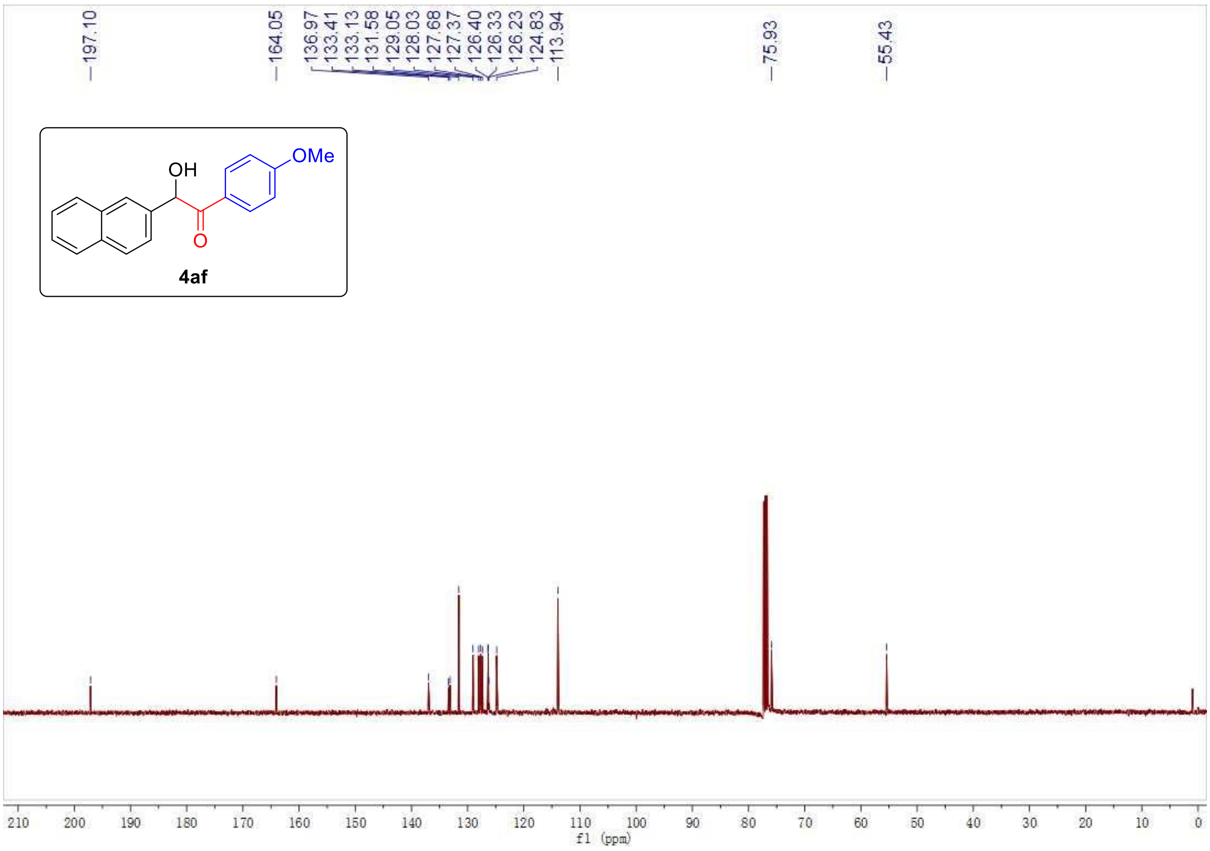

^{1}H NMR (400 MHz, CDCl$_3$) spectrum of 4ag

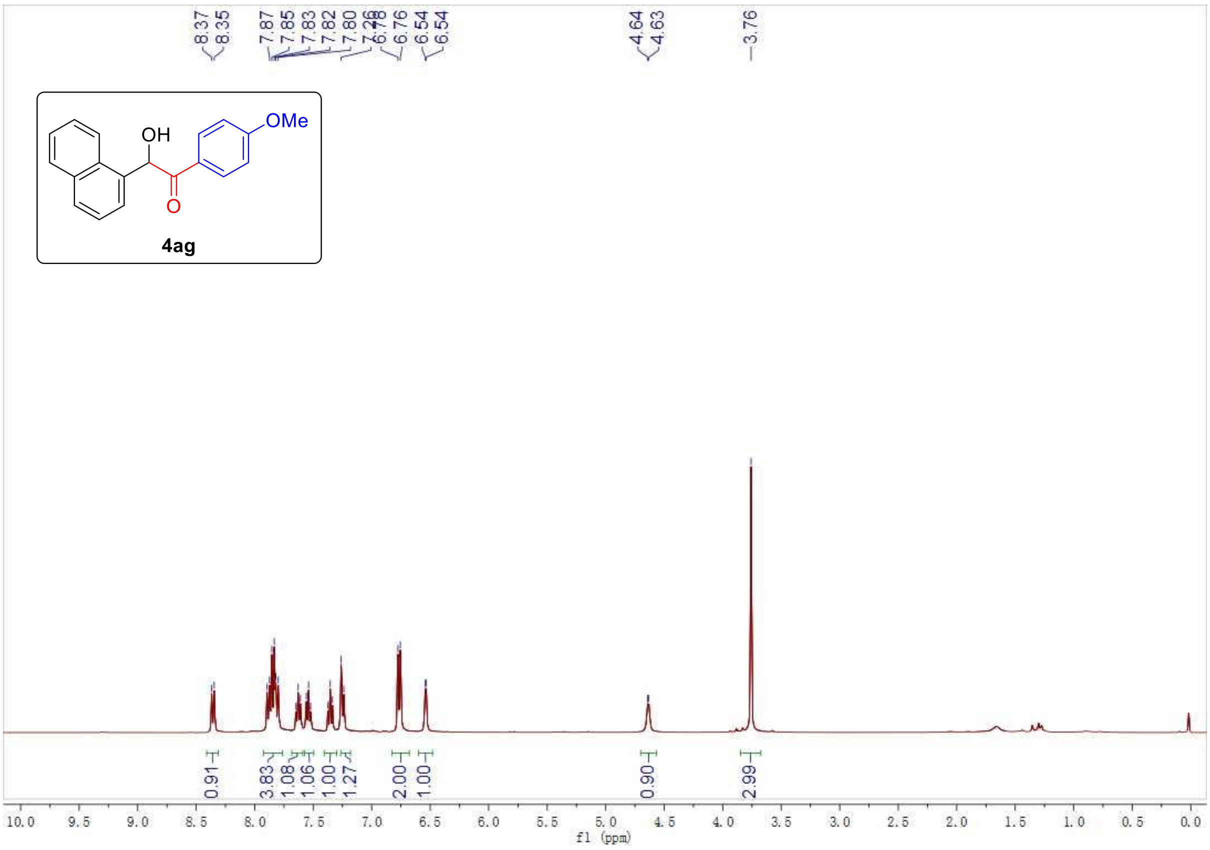

^{13}C NMR (101 MHz, CDCl$_3$) spectrum of 4ag

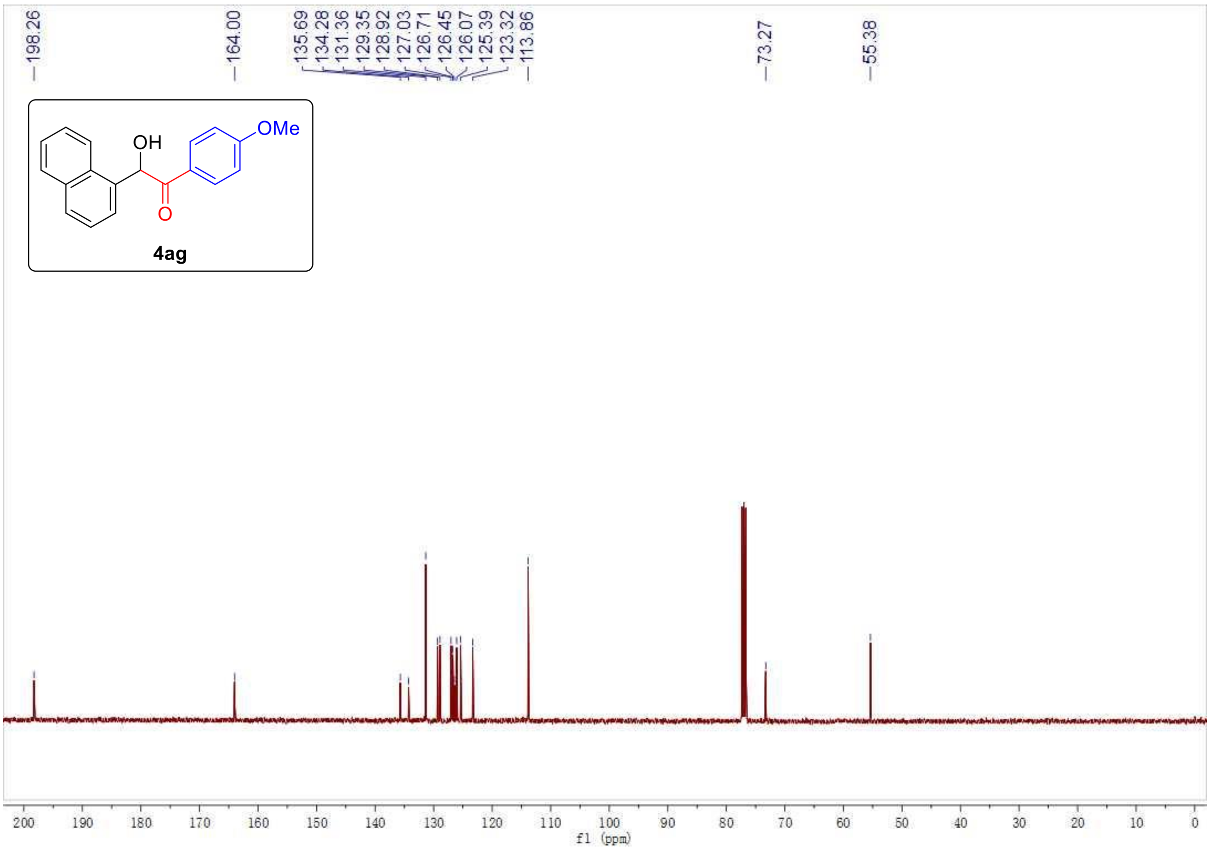

^{1}H NMR (400 MHz, $CDCl_3$) spectrum of 5a

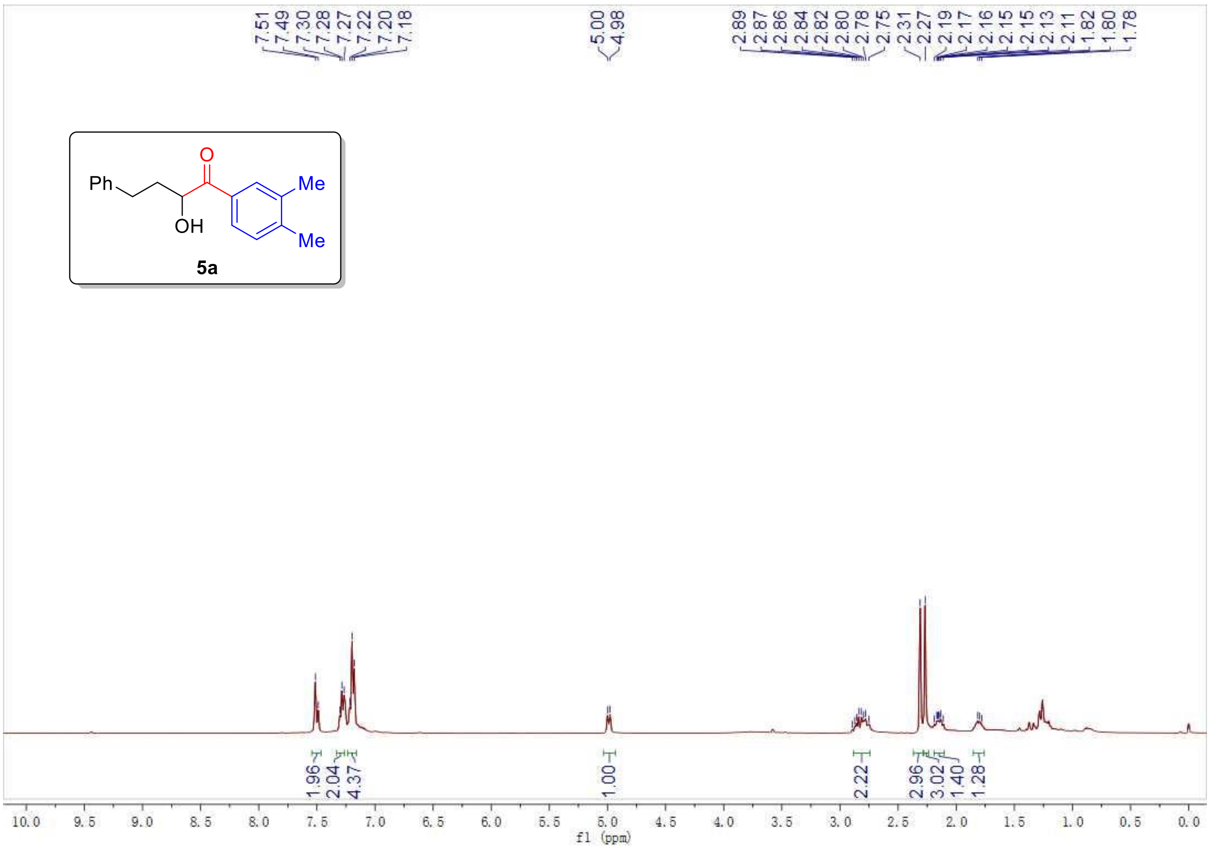

^{13}C NMR (101 MHz, $CDCl_3$) spectrum of 5a

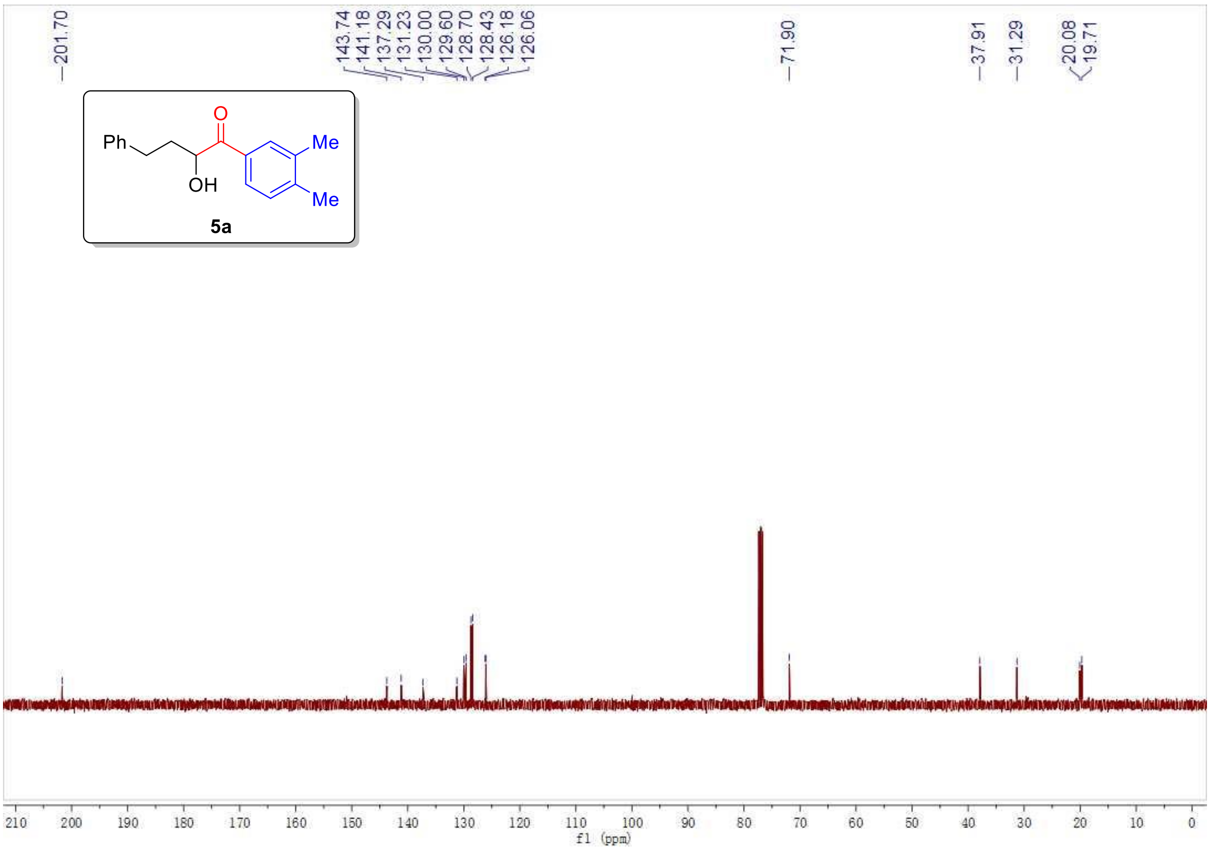

^{1}H NMR (400 MHz, $CDCl_3$) spectrum of 5b

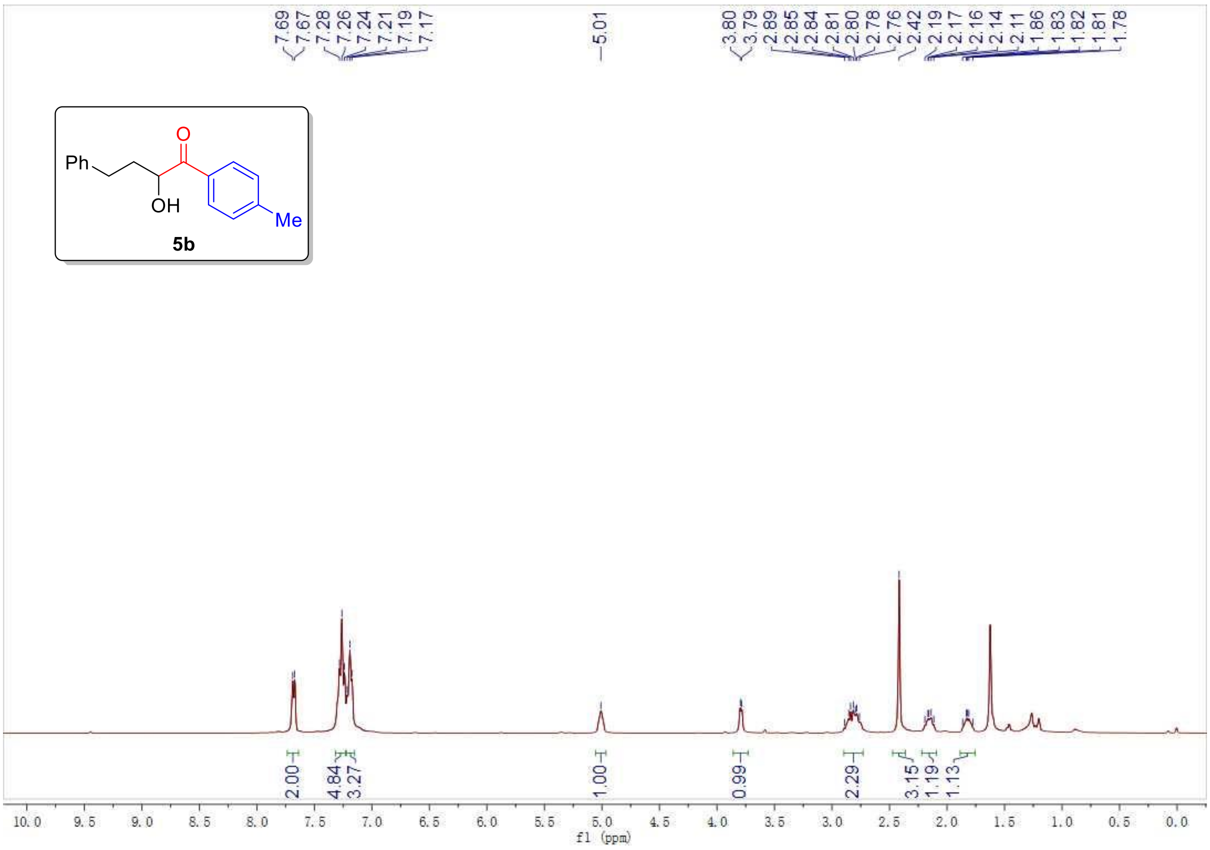

^{13}C NMR (101 MHz, $CDCl_3$) spectrum of 5b

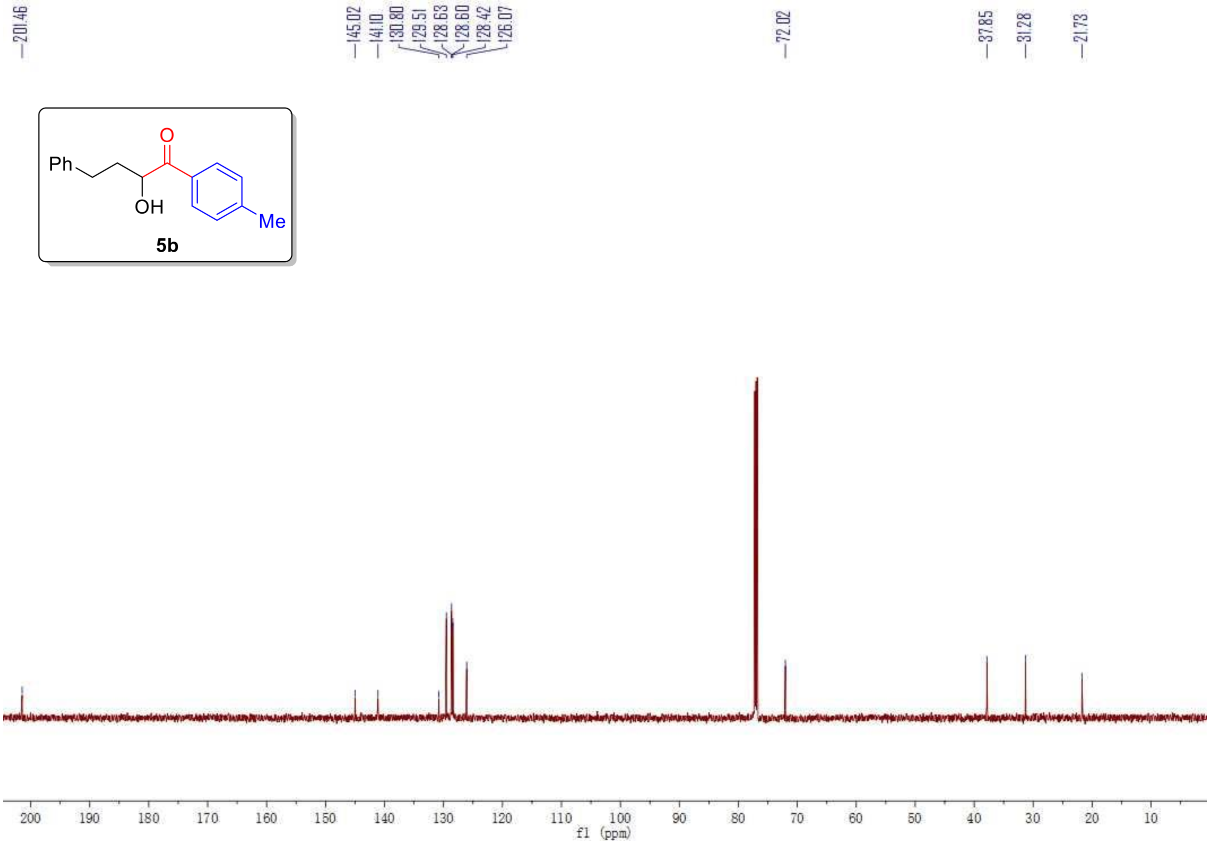

^{1}H NMR (400 MHz, $CDCl_3$) spectrum of 5c

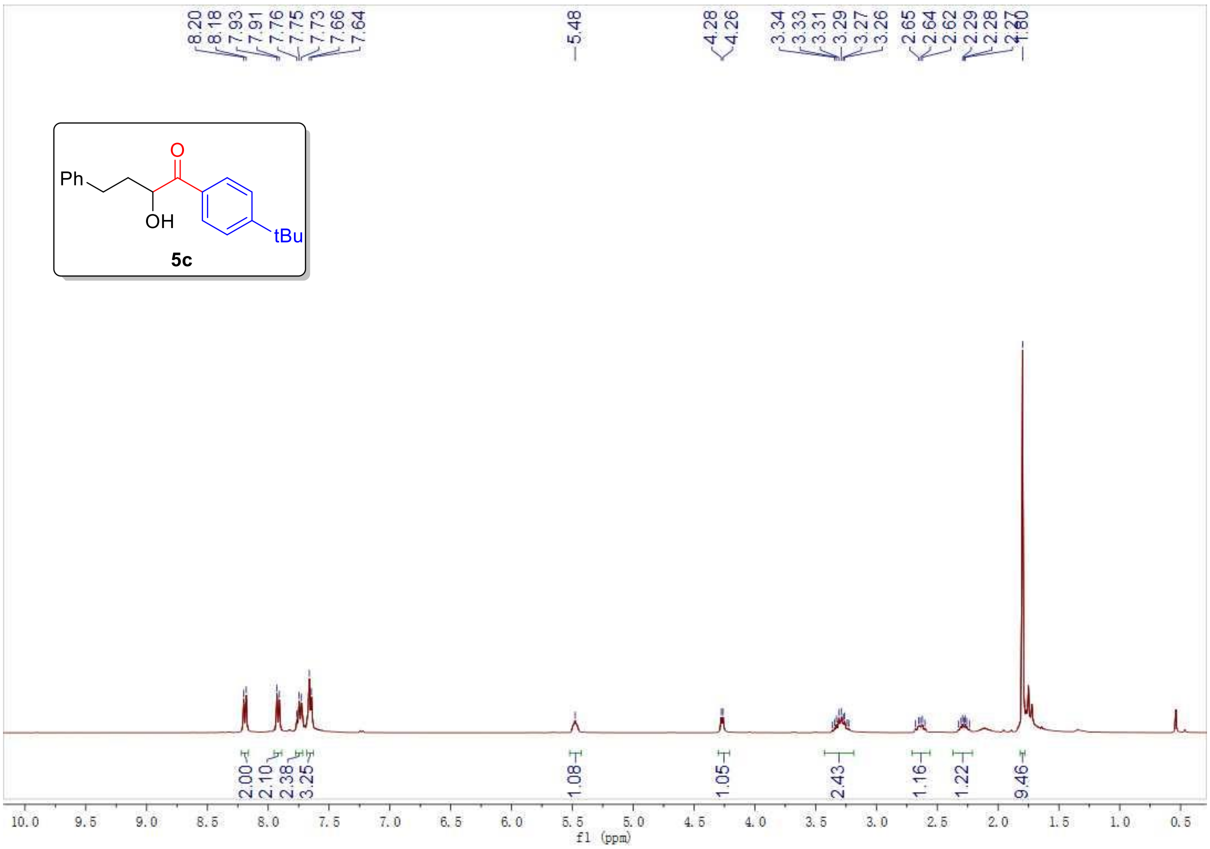

^{13}C NMR (101 MHz, $CDCl_3$) spectrum of 5c

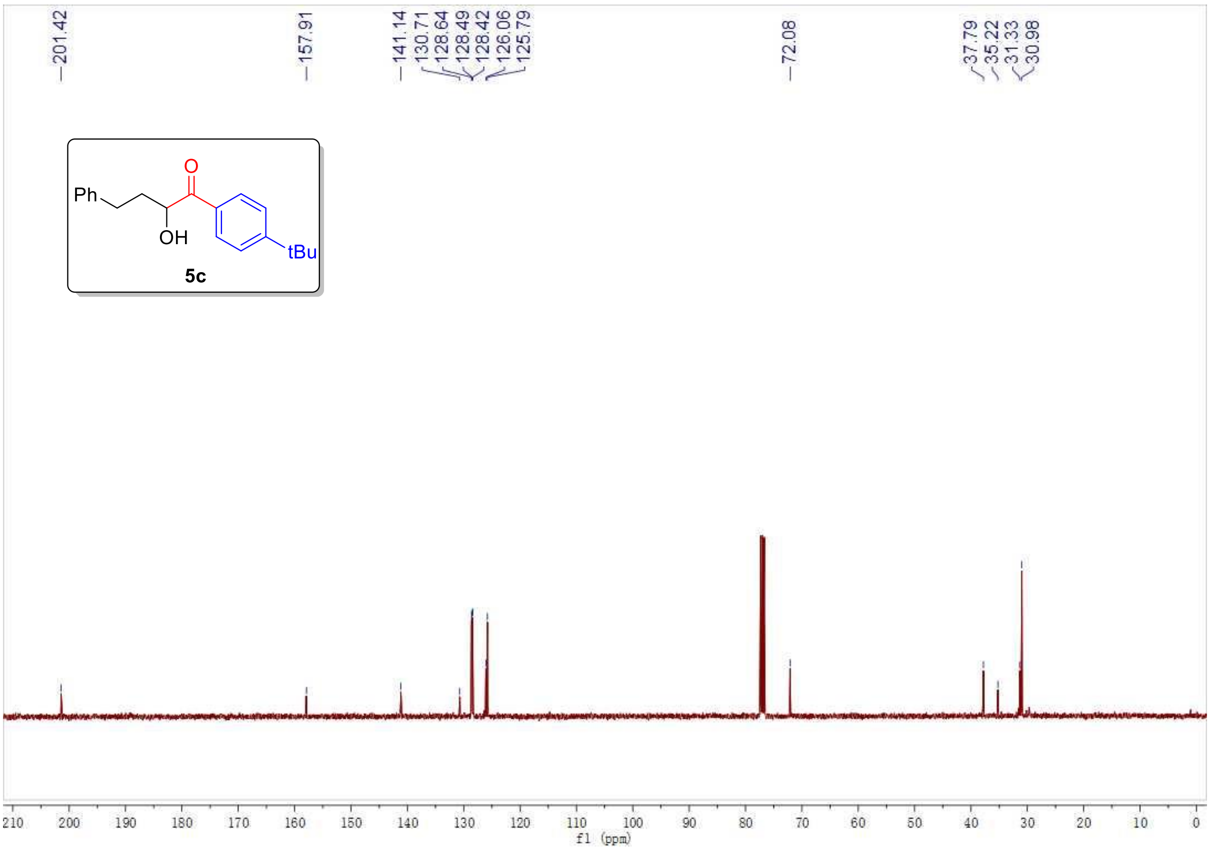

^{1}H NMR (400 MHz, $CDCl_3$) spectrum of 5d

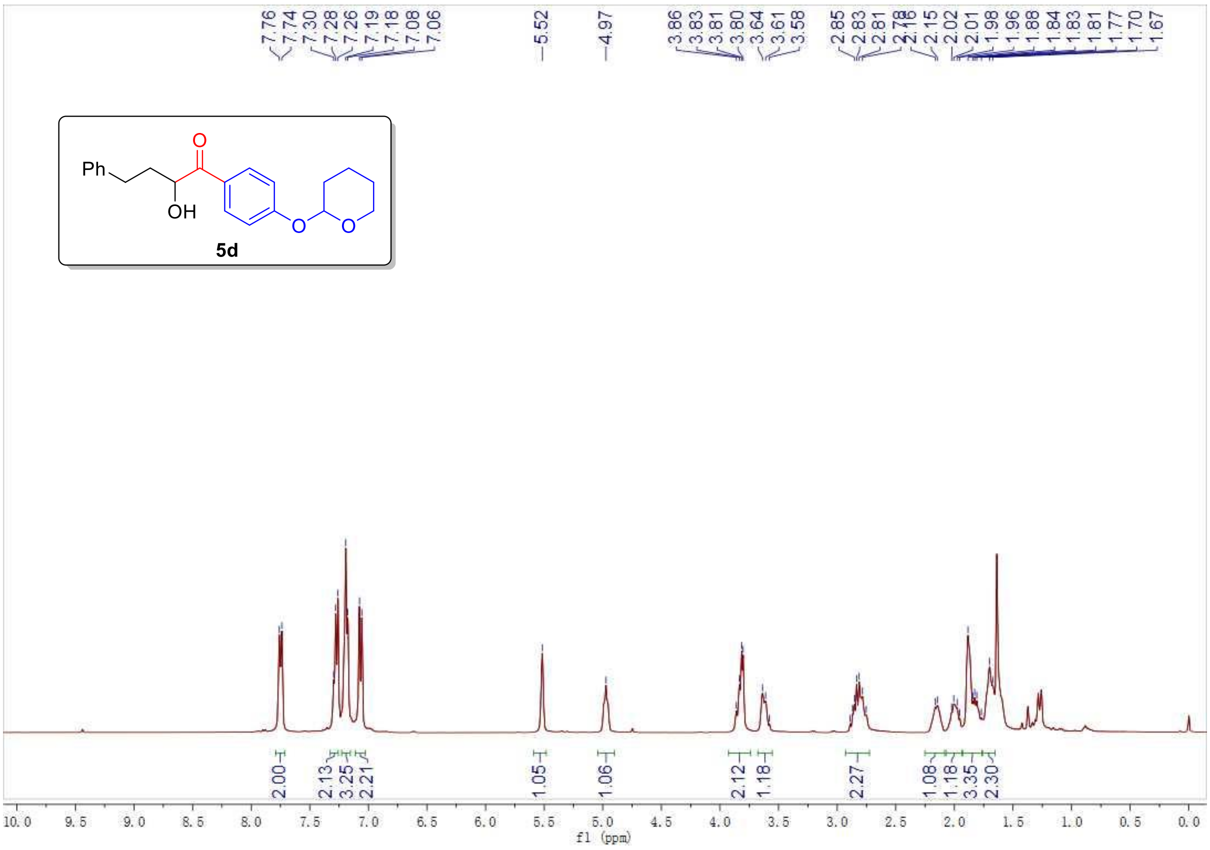

^{13}C NMR (101 MHz, $CDCl_3$) spectrum of 5d

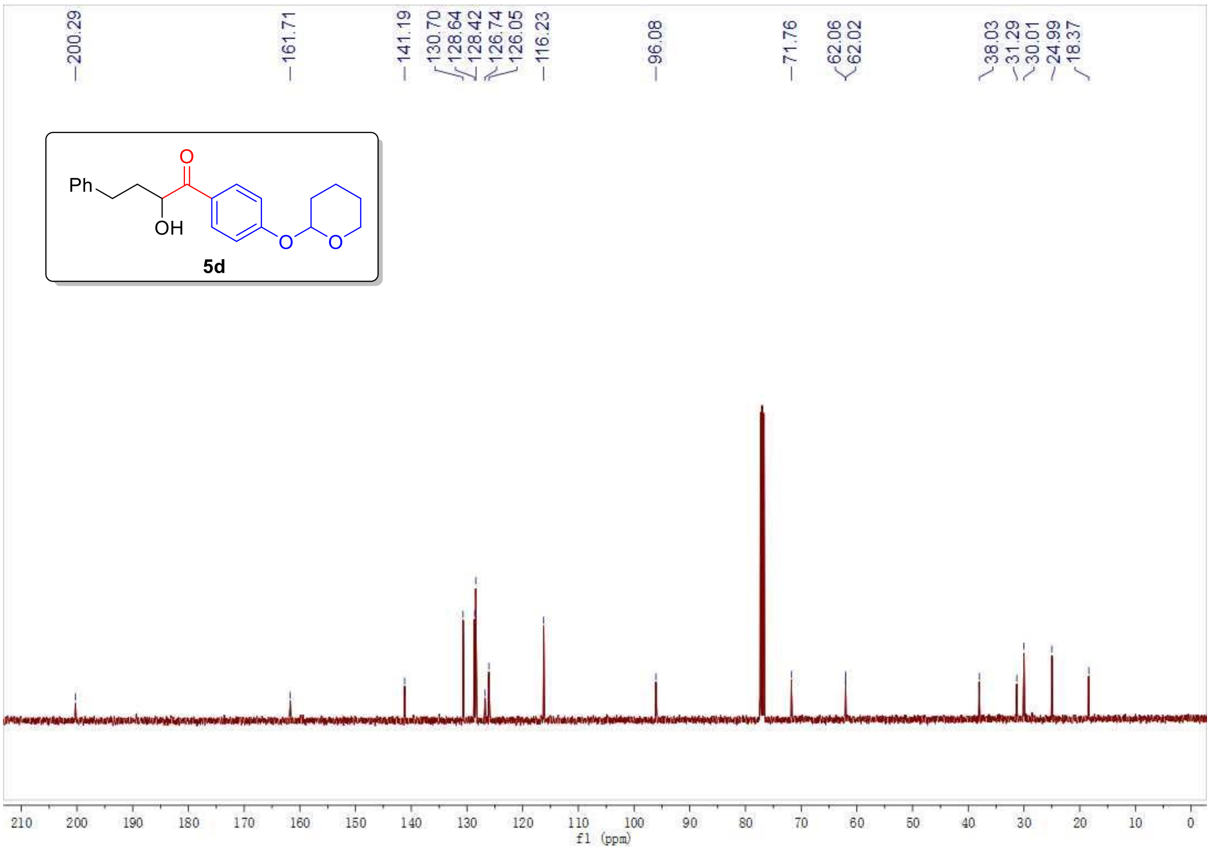

^{1}H NMR (400 MHz, $CDCl_3$) spectrum of 5e

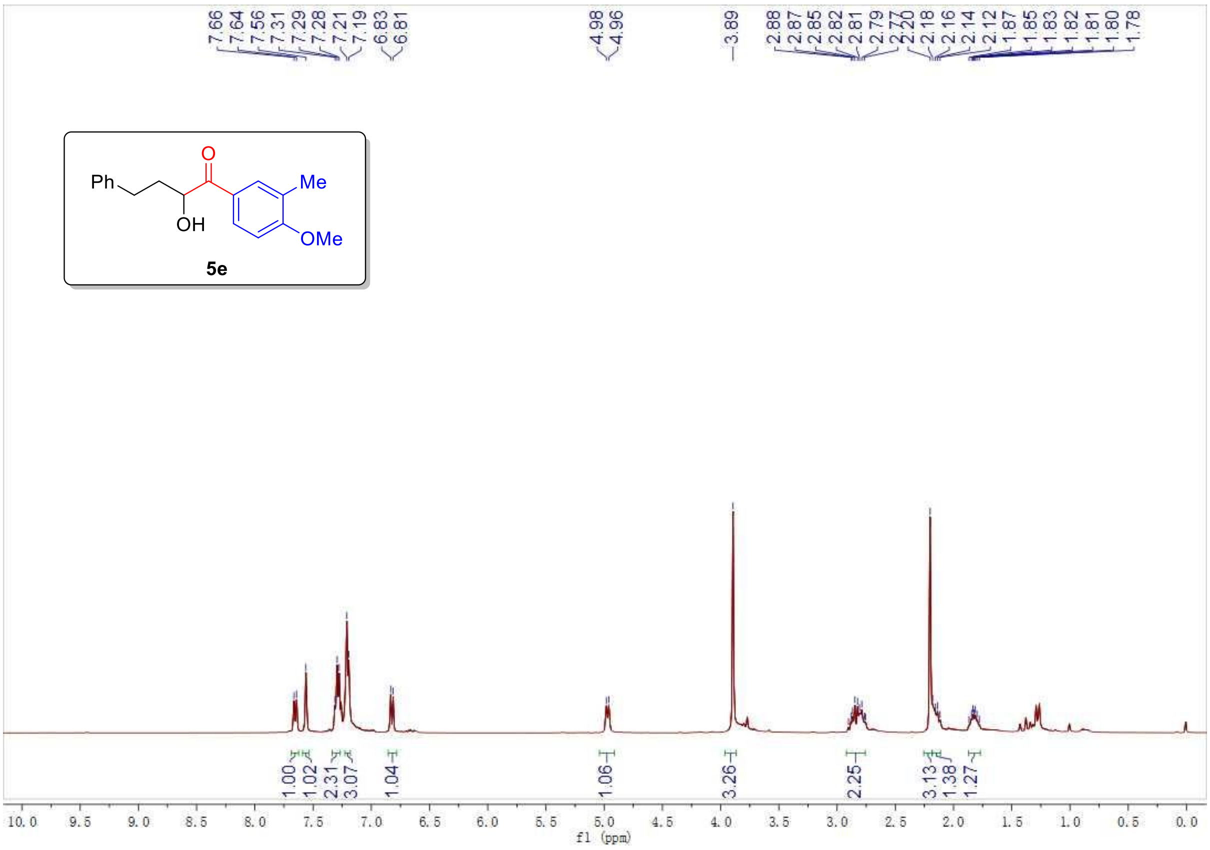

^{13}C NMR (101 MHz, $CDCl_3$) spectrum of 5e

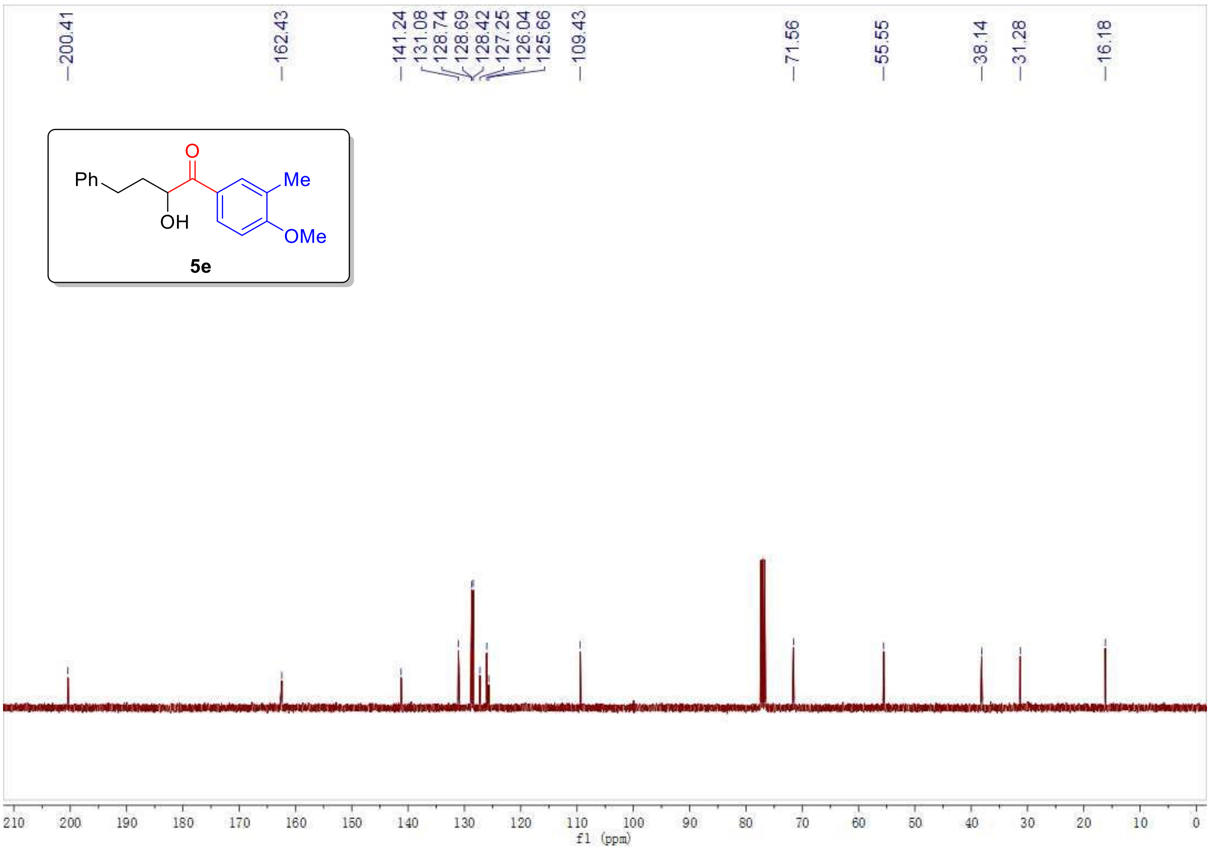

^{1}H NMR (400 MHz, $CDCl_3$) spectrum of 5f

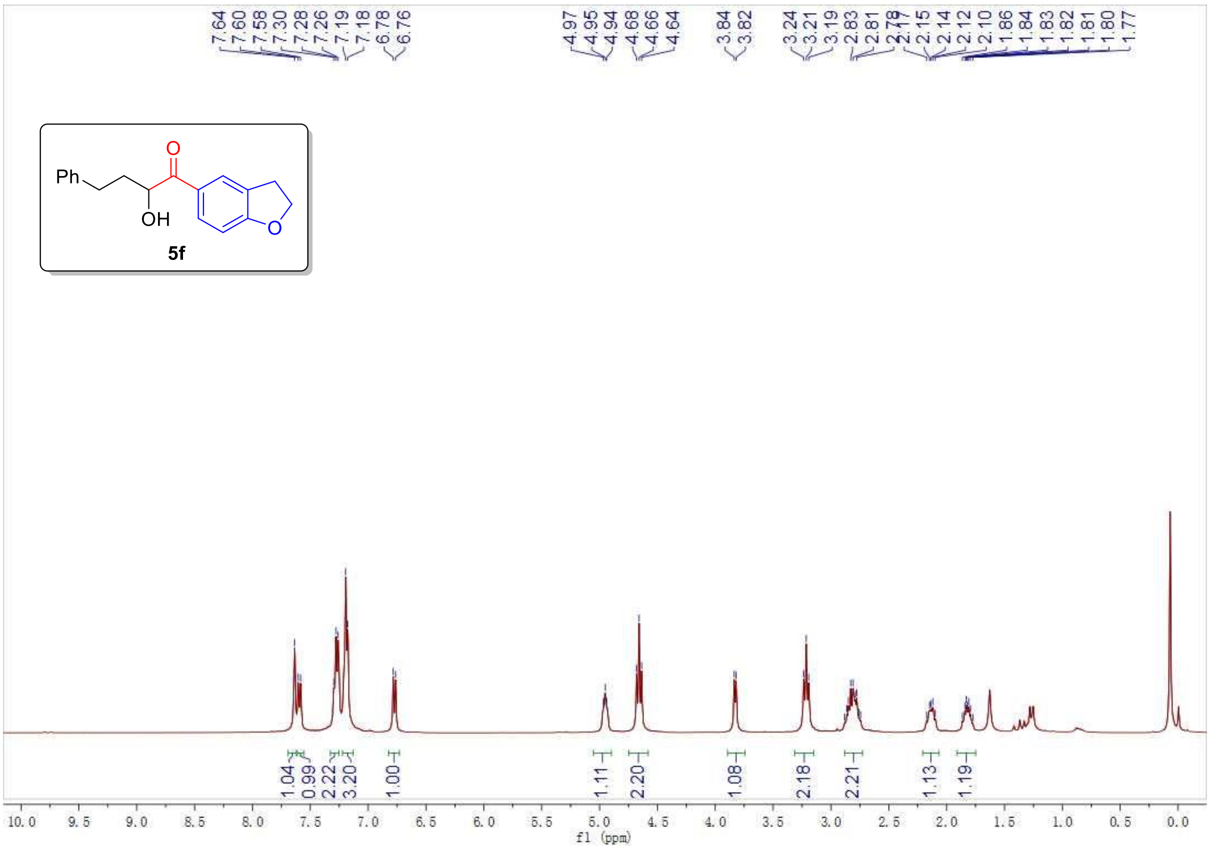

^{13}C NMR (101 MHz, $CDCl_3$) spectrum of 5f

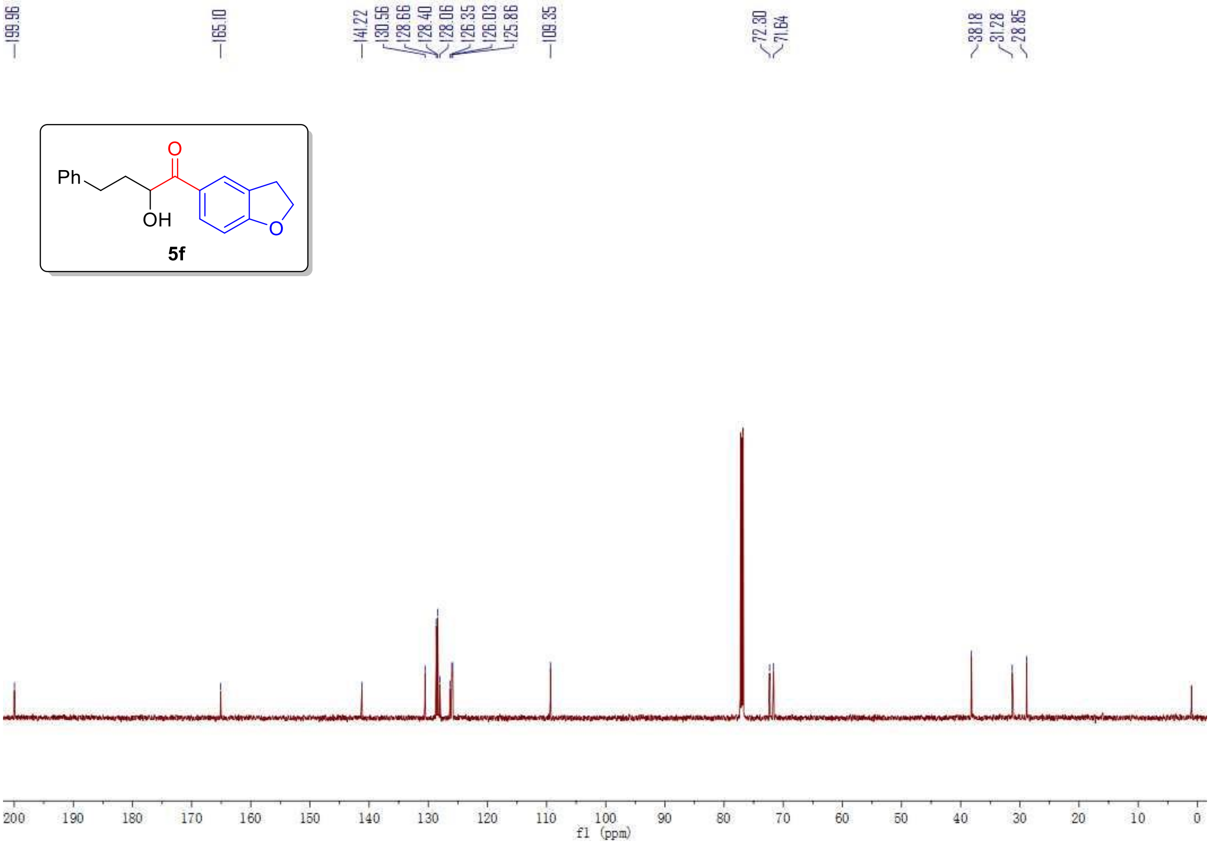

^{1}H NMR (400 MHz, $CDCl_3$) spectrum of 5g

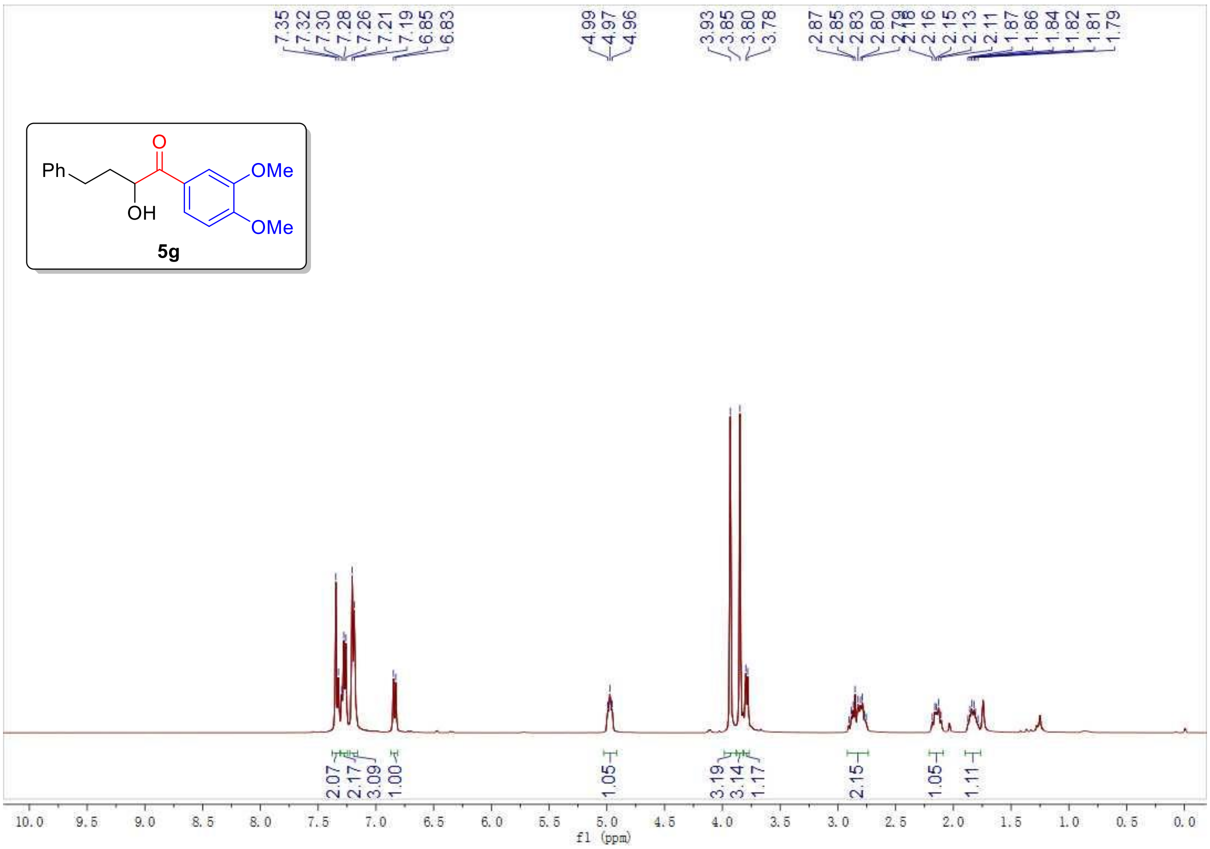

^{13}C NMR (101 MHz, $CDCl_3$) spectrum of 5g

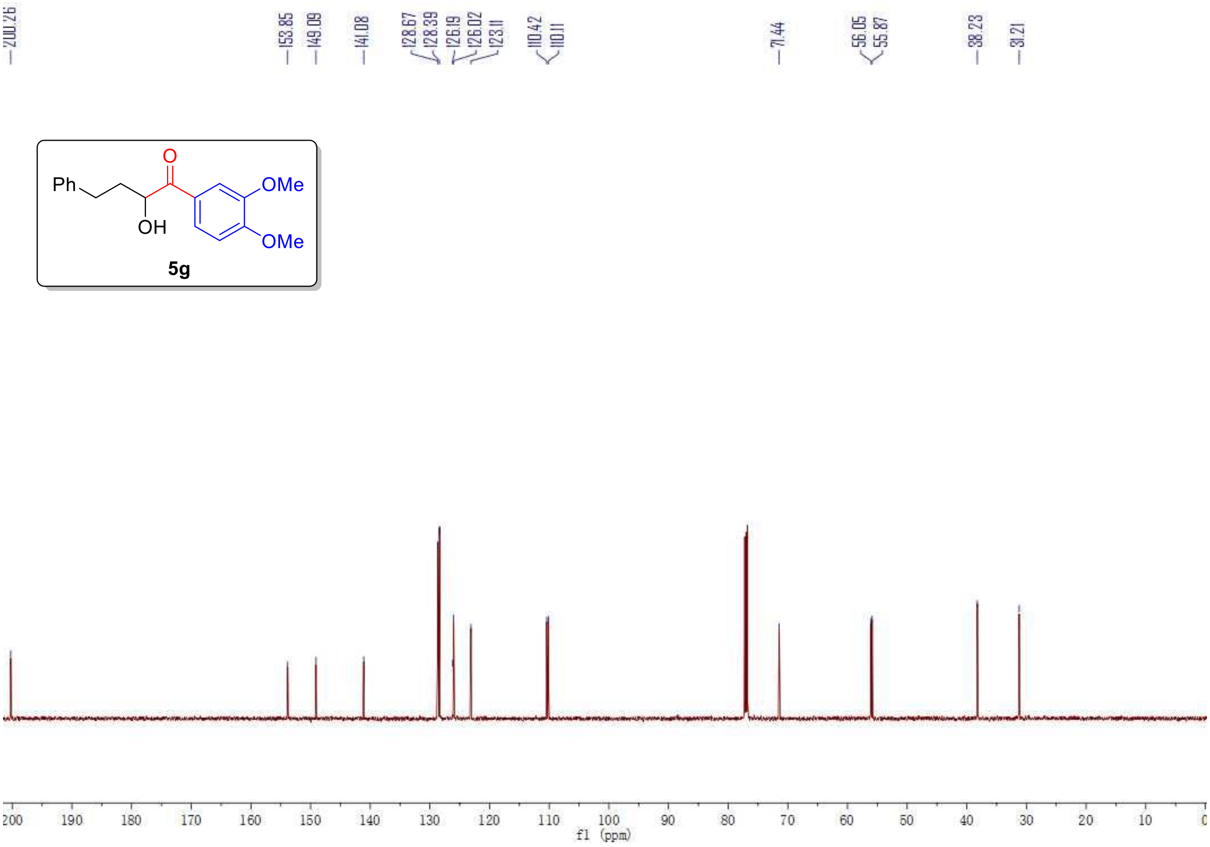

^{1}H NMR (400 MHz, $CDCl_3$) spectrum of 5h

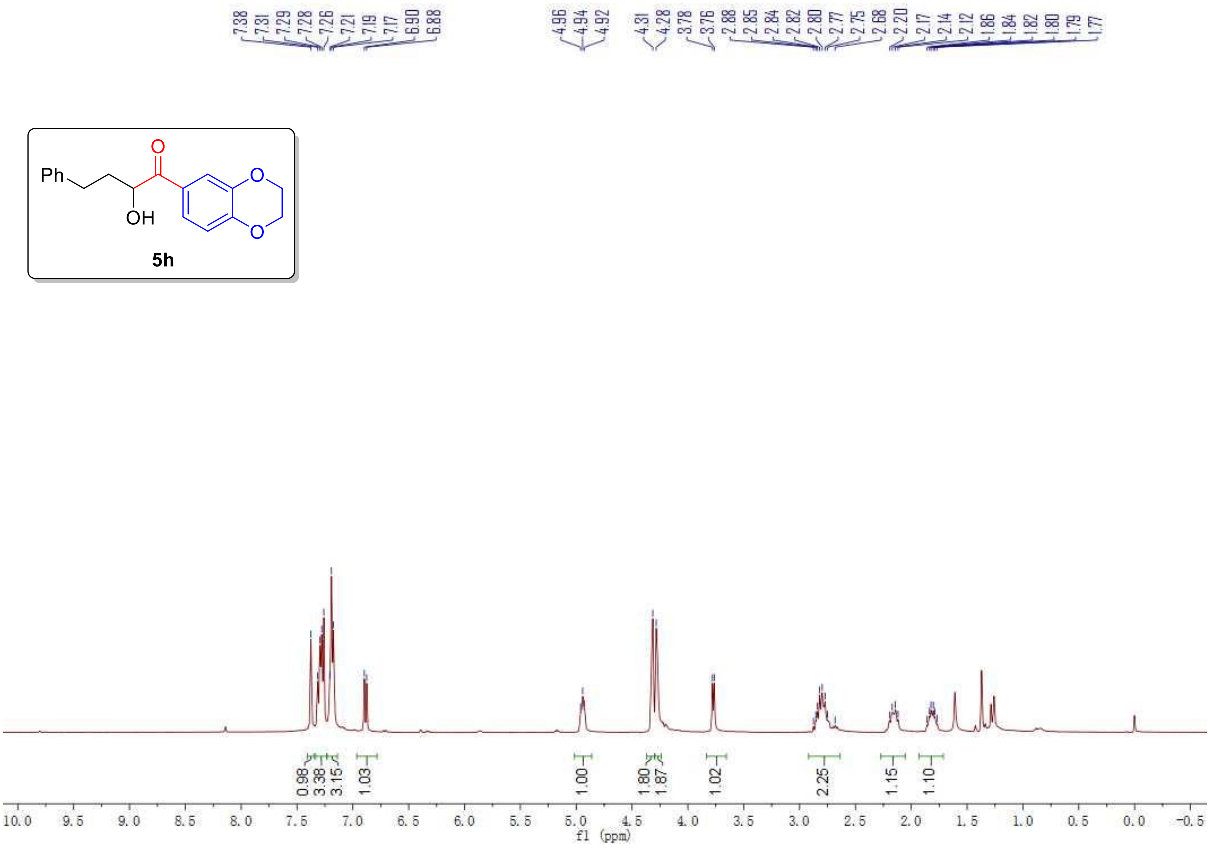

^{13}C NMR (101 MHz, $CDCl_3$) spectrum of 5h

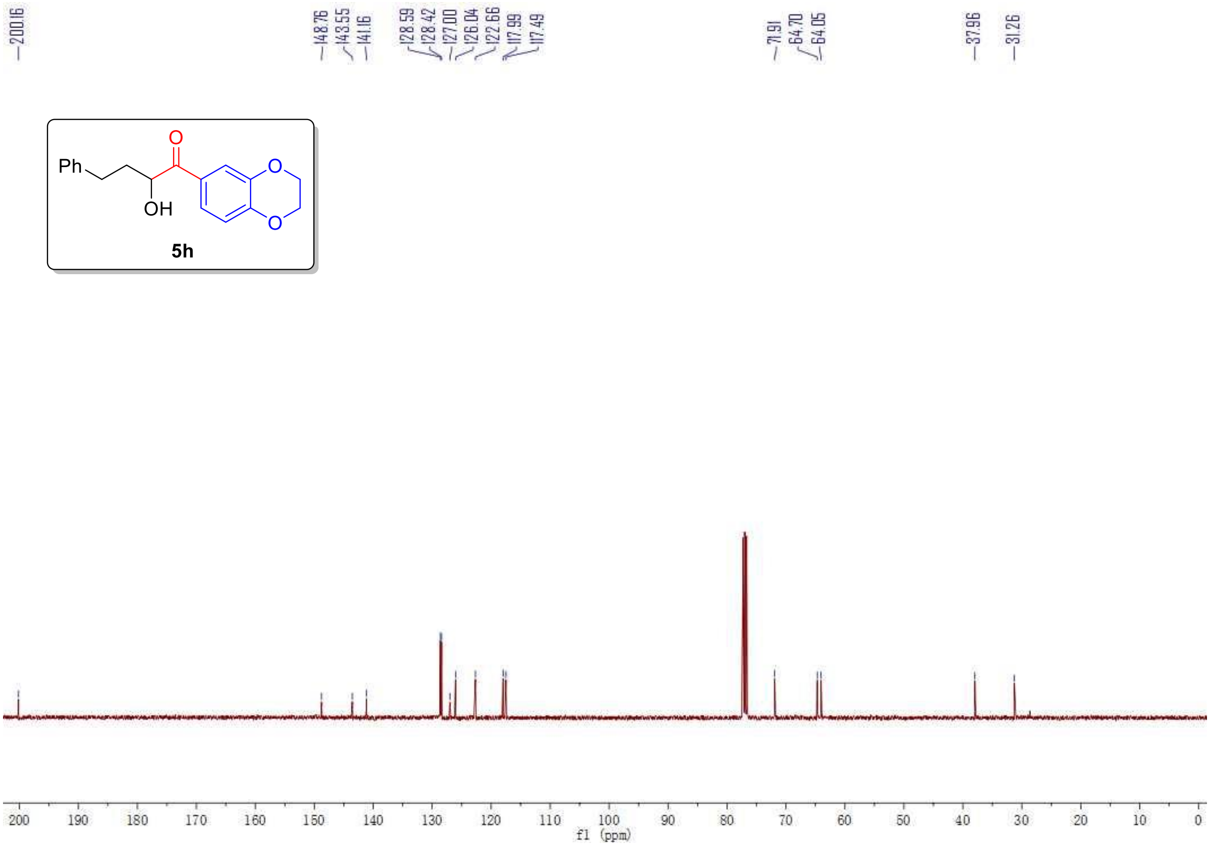

^{1}H NMR (400 MHz, $CDCl_3$) spectrum of 5i

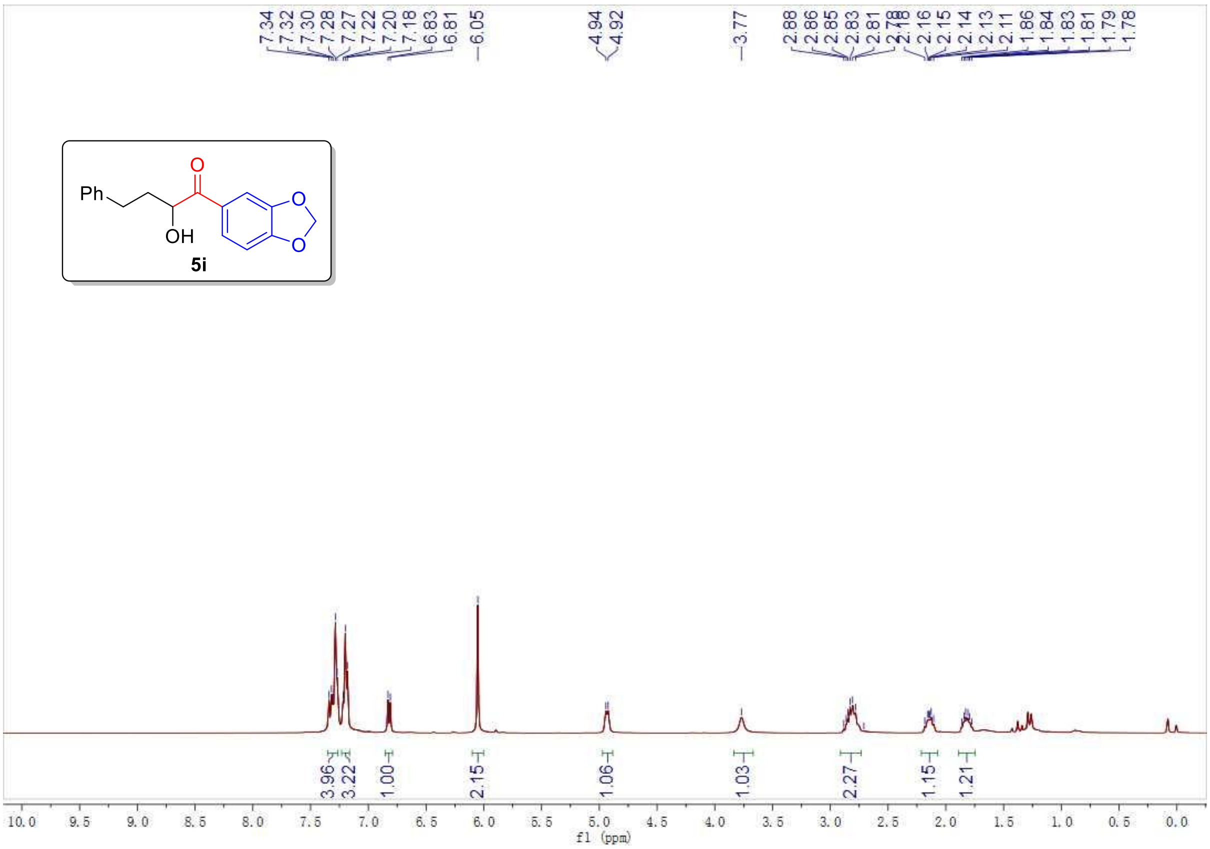

^{13}C NMR (101 MHz, $CDCl_3$) spectrum of 5i

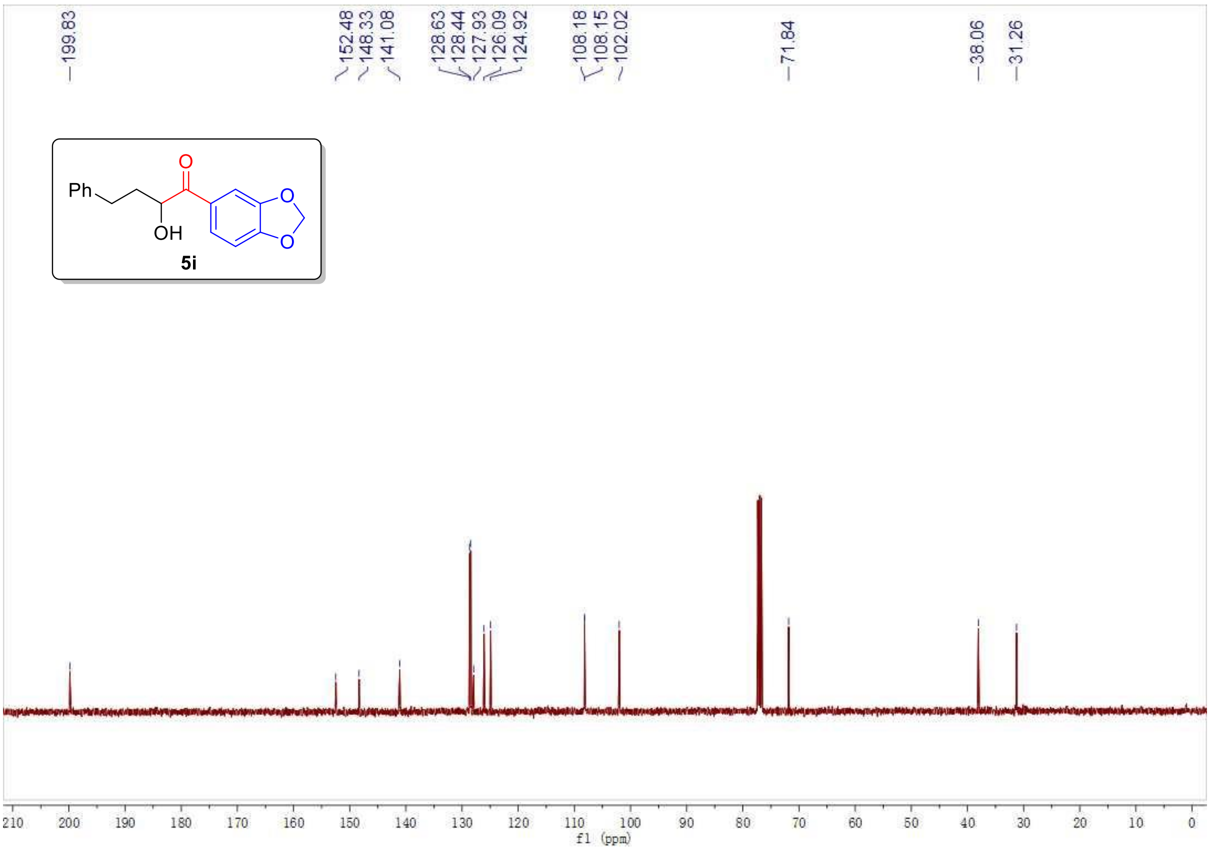

^{1}H NMR (400 MHz, $CDCl_3$) spectrum of 5j

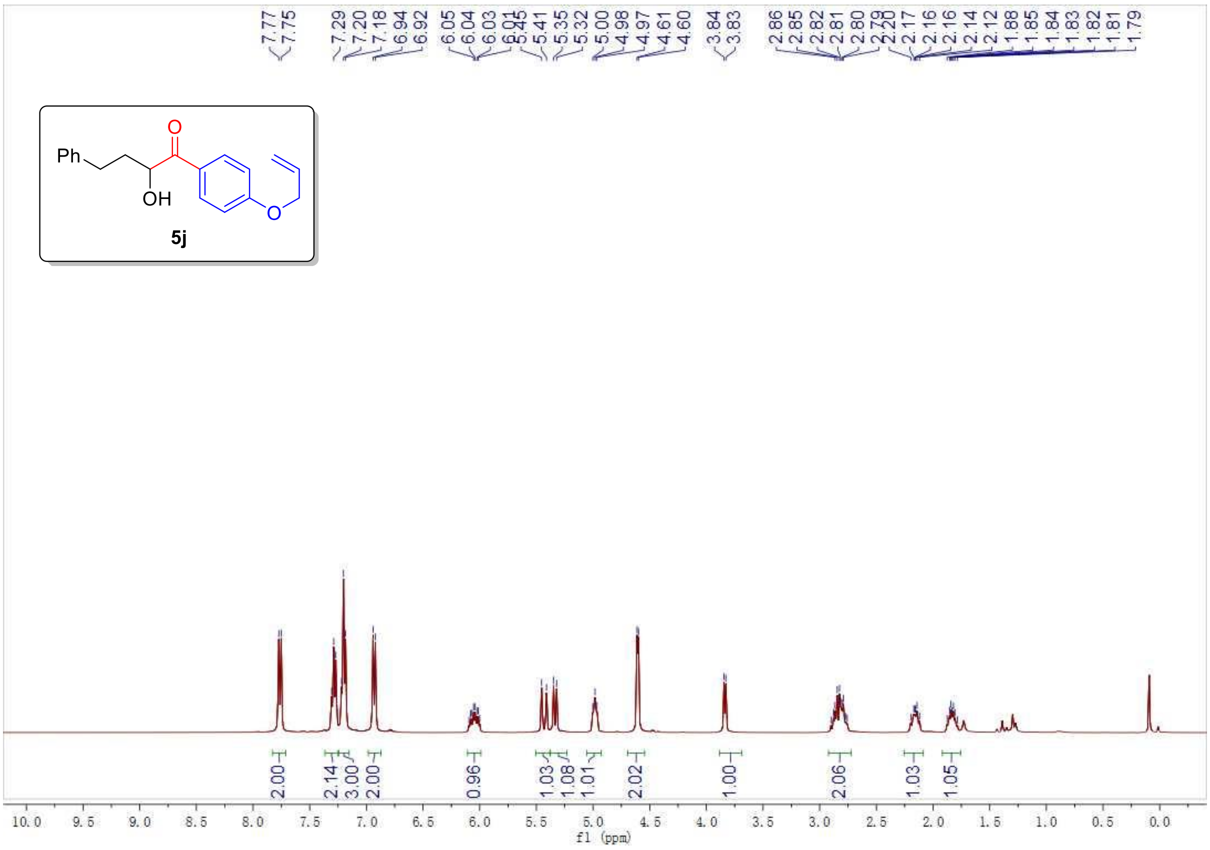

^{13}C NMR (101 MHz, $CDCl_3$) spectrum of 5j

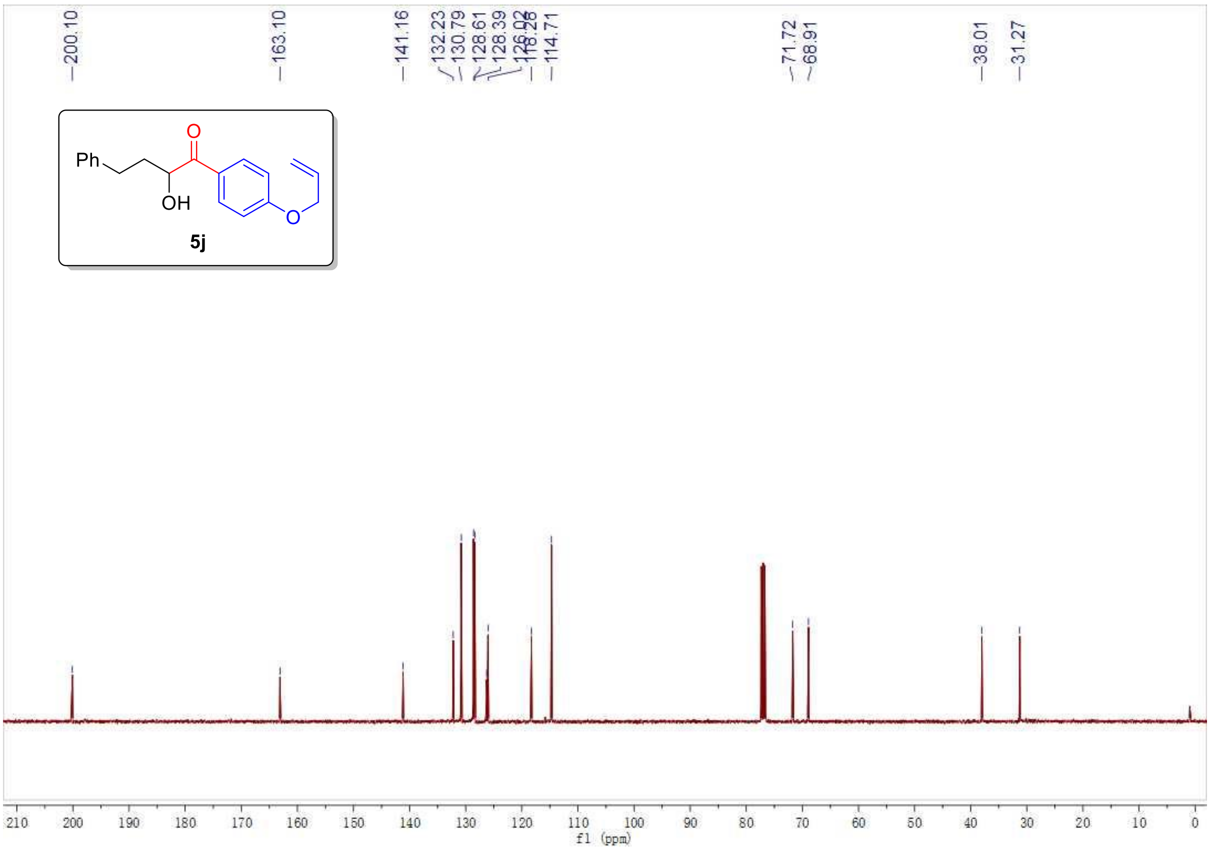

^{1}H NMR (400 MHz, $CDCl_3$) spectrum of 5k

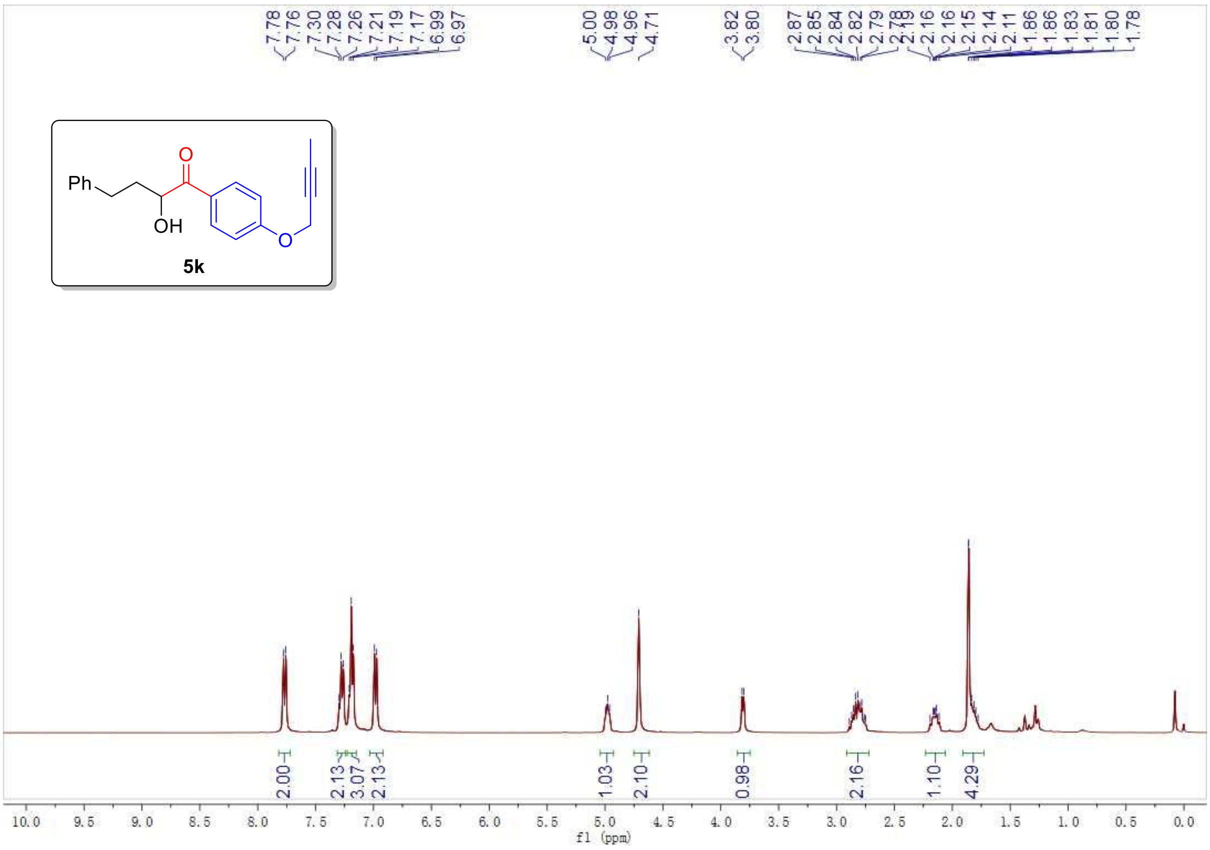

^{13}C NMR (101 MHz, $CDCl_3$) spectrum of 5k

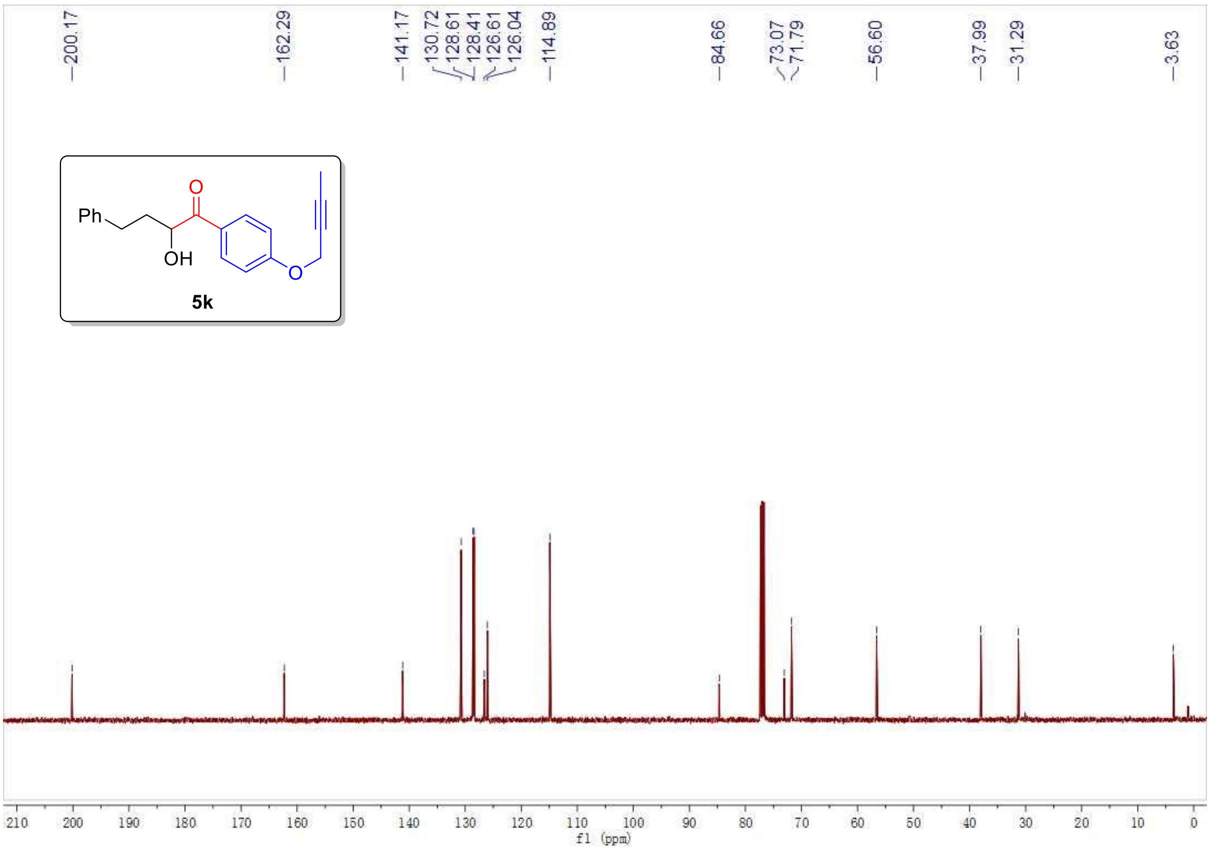

^{1}H NMR (400 MHz, $CDCl_3$) spectrum of 5l

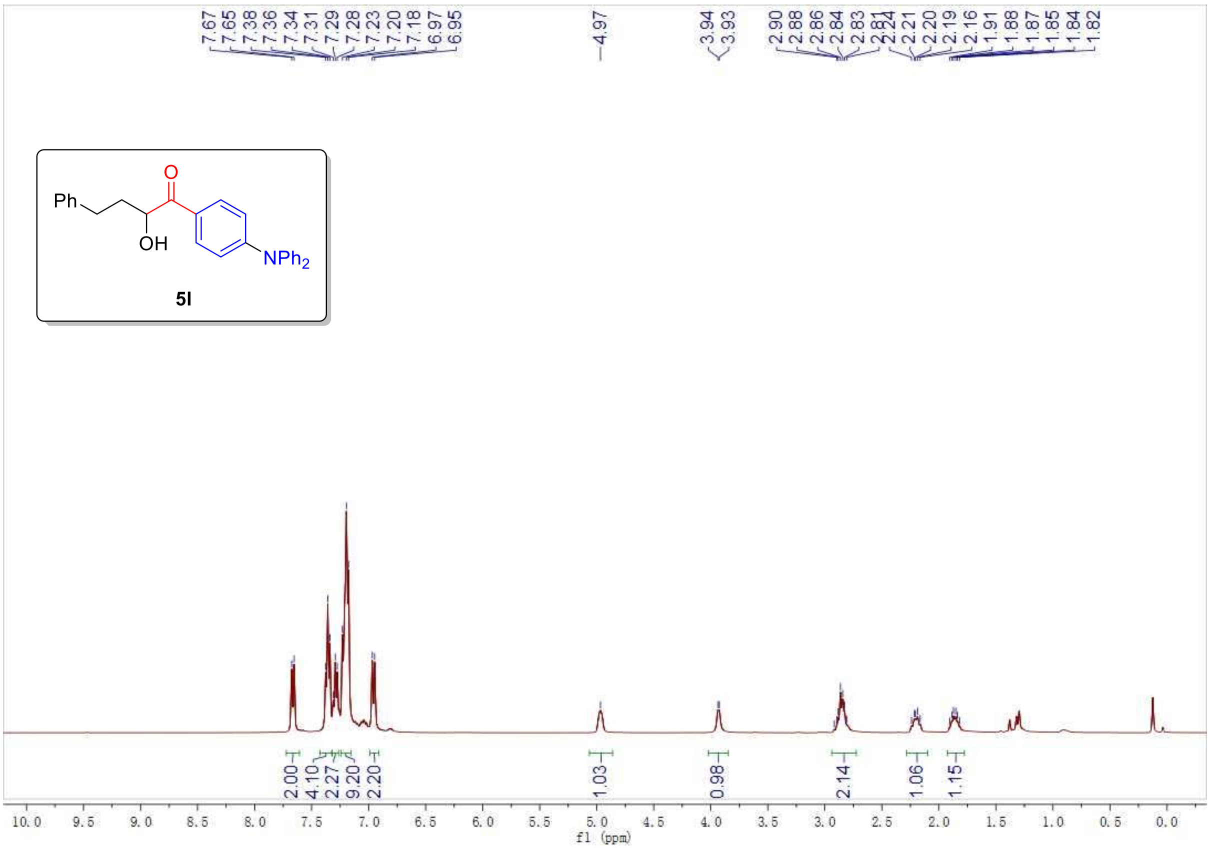

^{13}C NMR (101 MHz, $CDCl_3$) spectrum of 5l

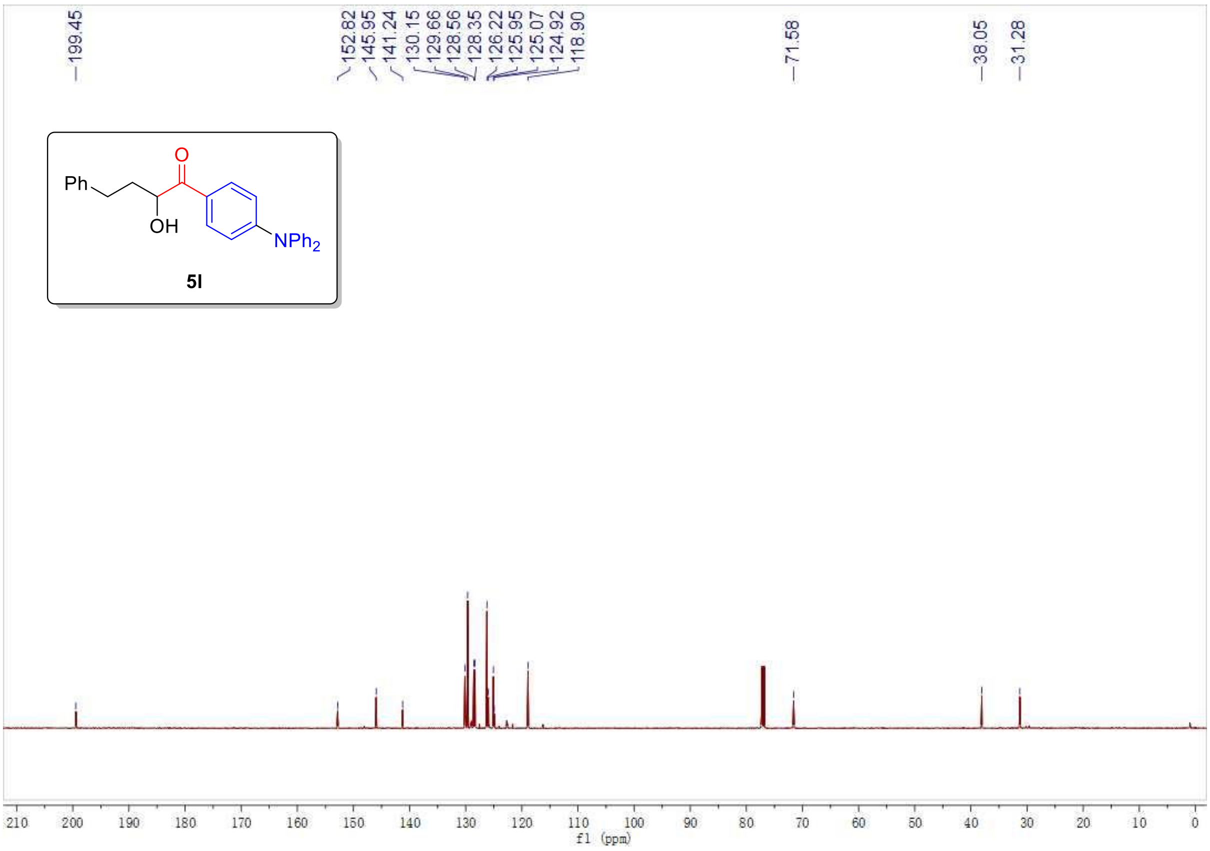

^{1}H NMR (400 MHz, $CDCl_3$) spectrum of 5m

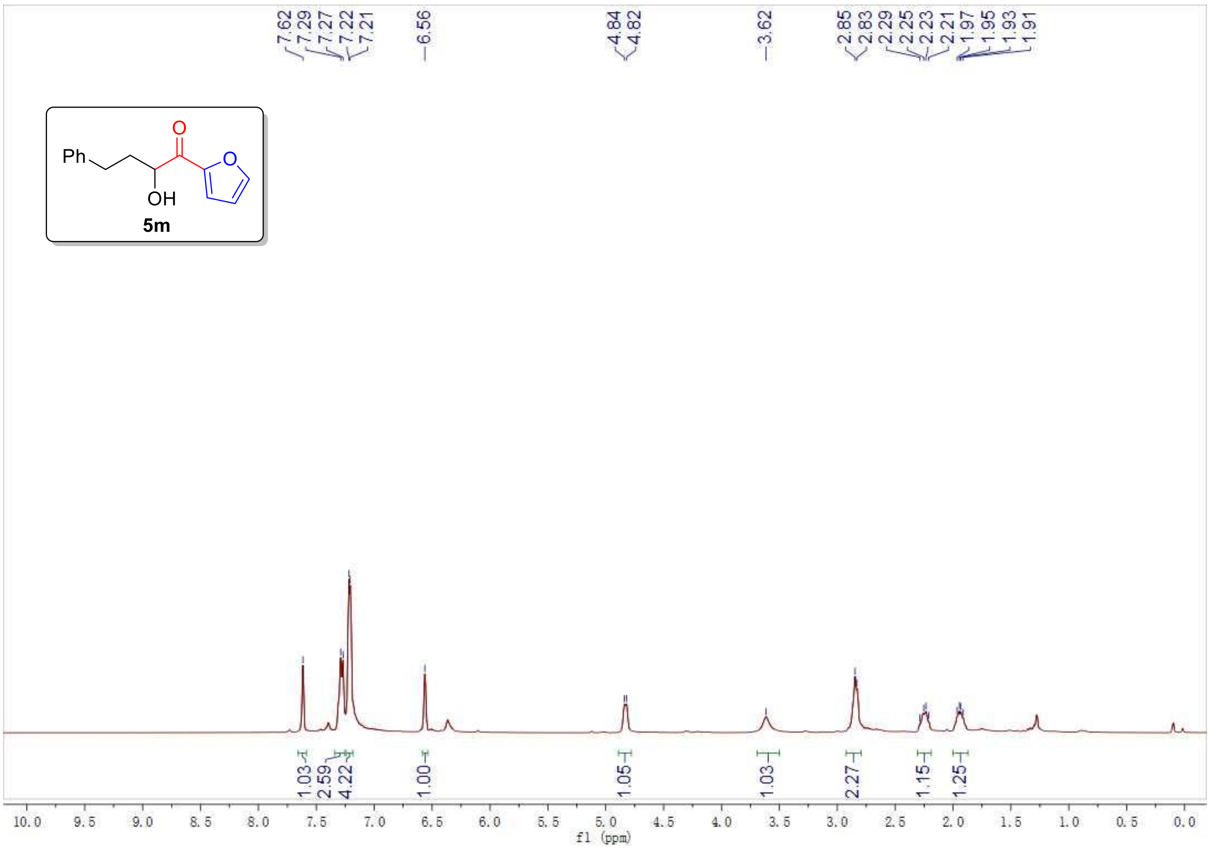

^{13}C NMR (101 MHz, $CDCl_3$) spectrum of 5m

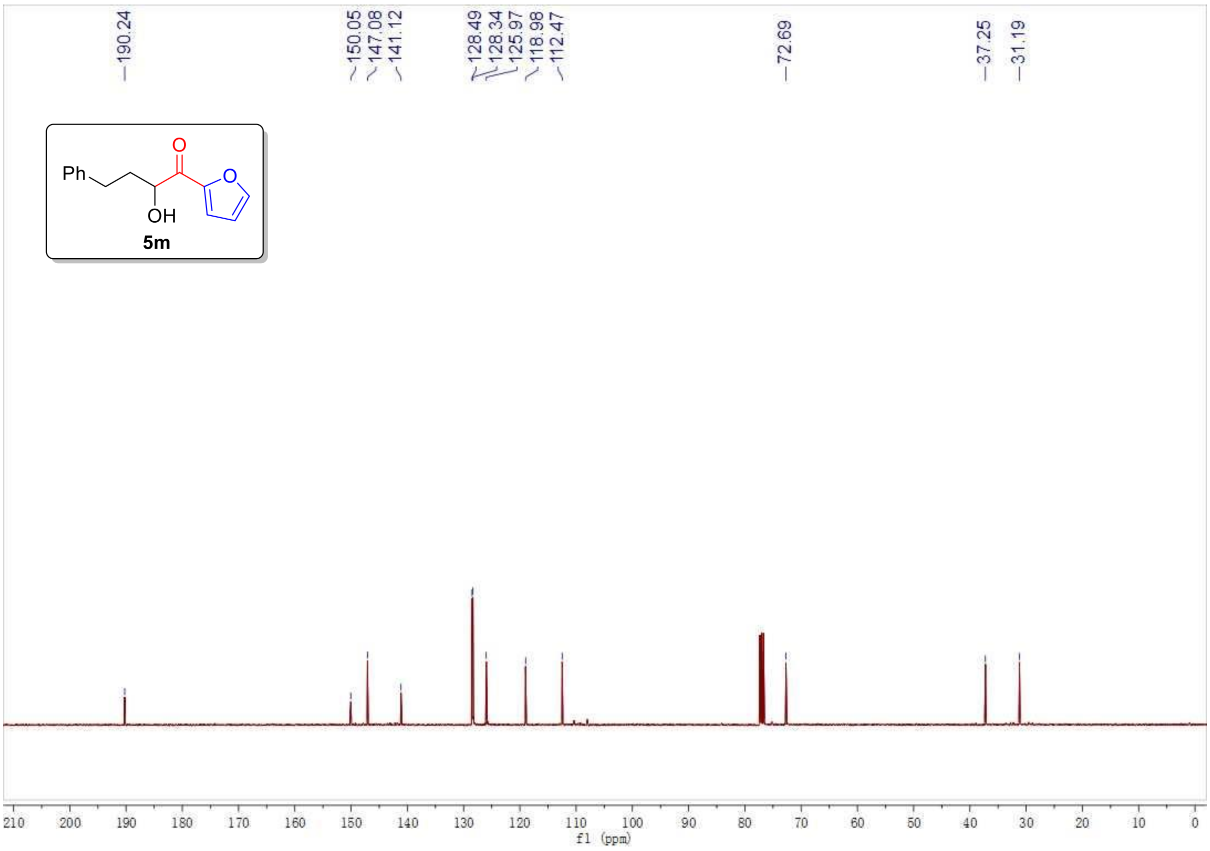

^{1}H NMR (400 MHz, $CDCl_3$) spectrum of 5n

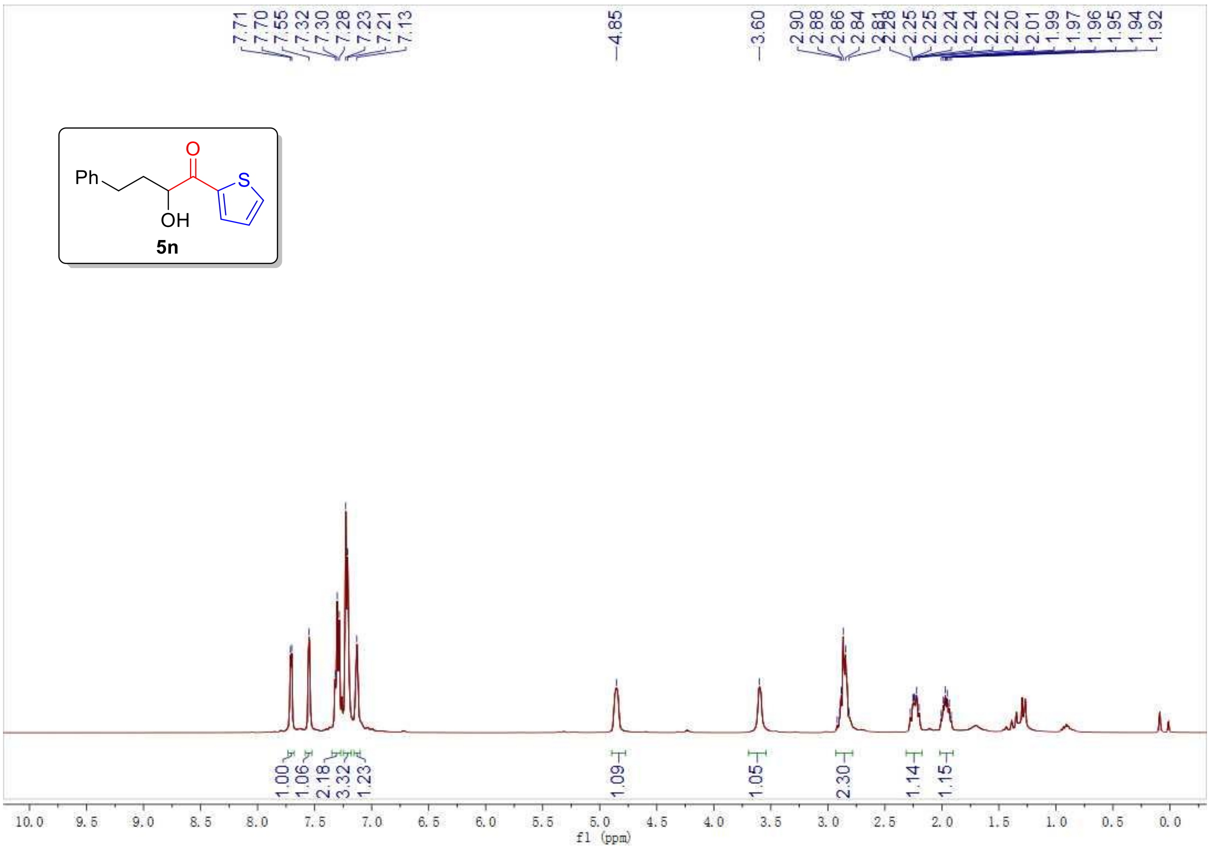

^{13}C NMR (101 MHz, $CDCl_3$) spectrum of 5n

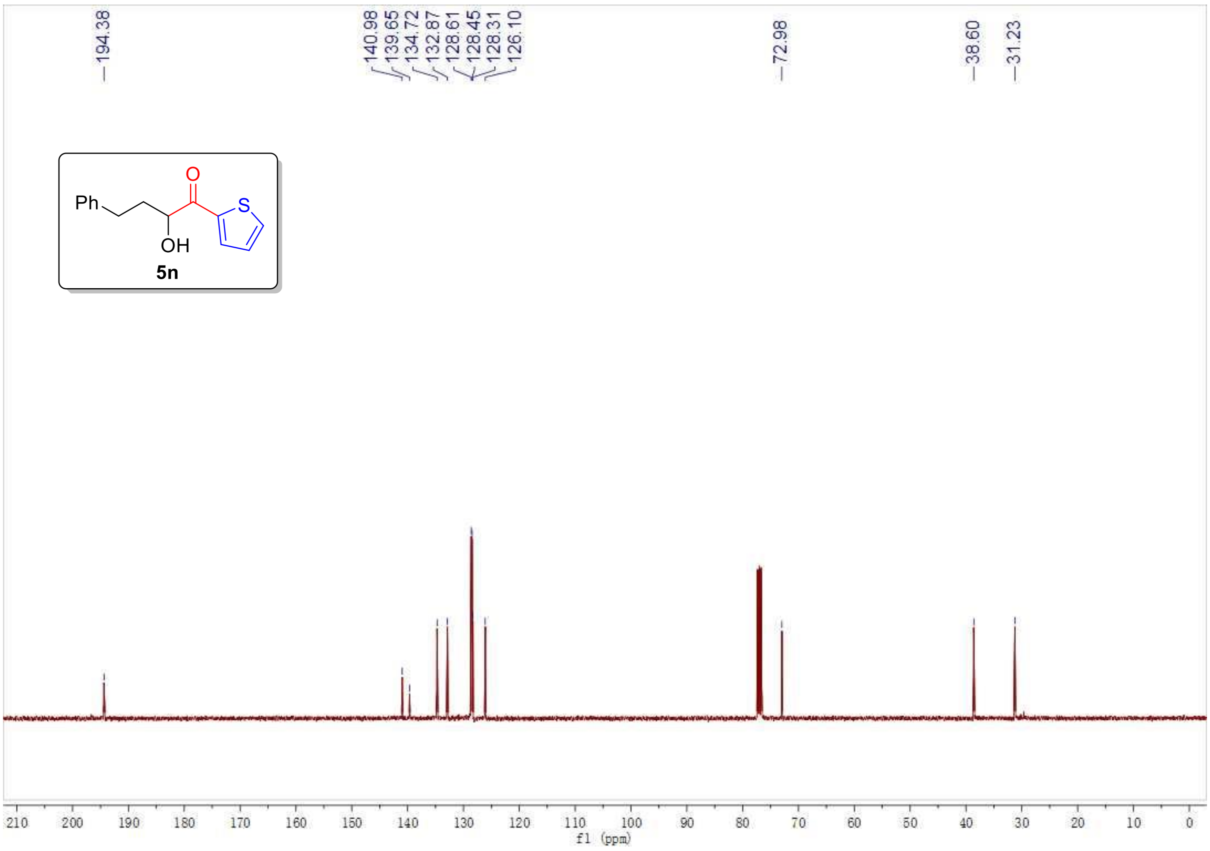

^{1}H NMR (400 MHz, $CDCl_3$) spectrum of 5o

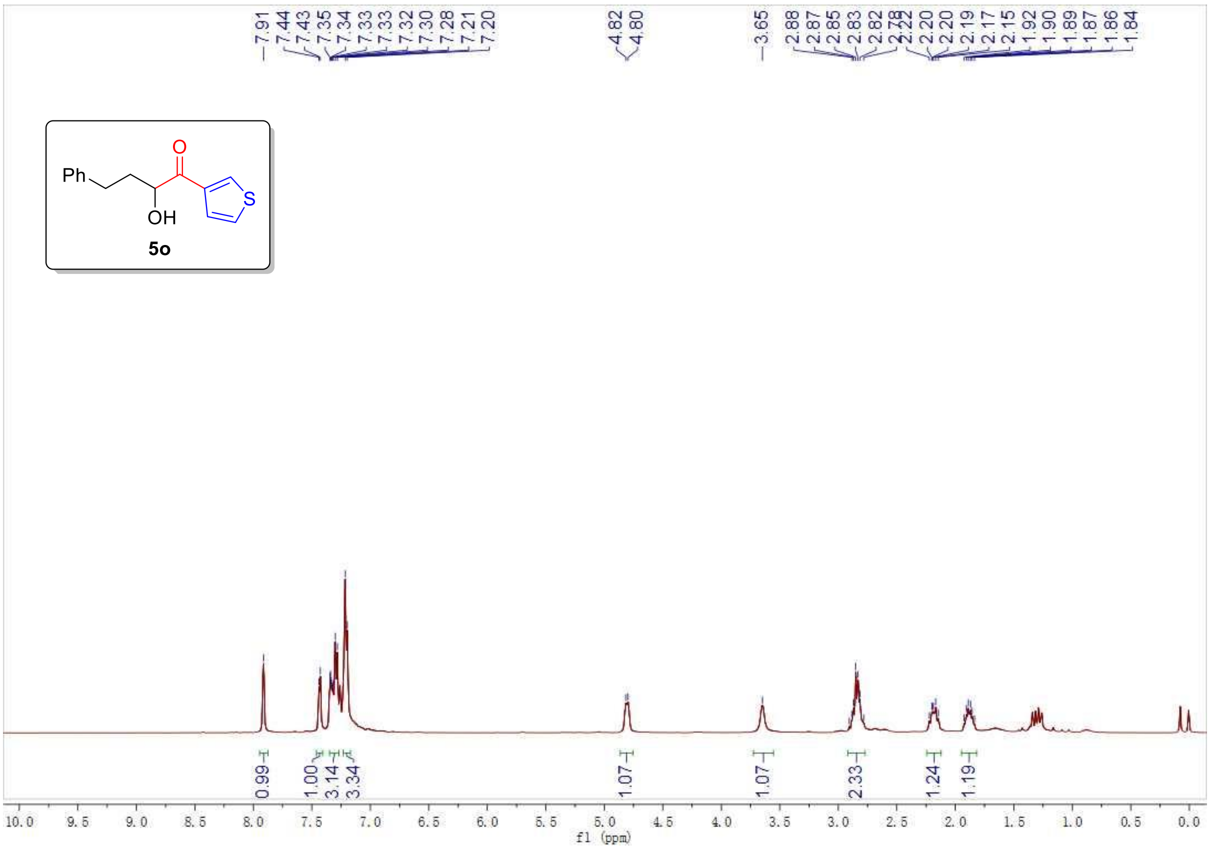

^{13}C NMR (101 MHz, $CDCl_3$) spectrum of 5o

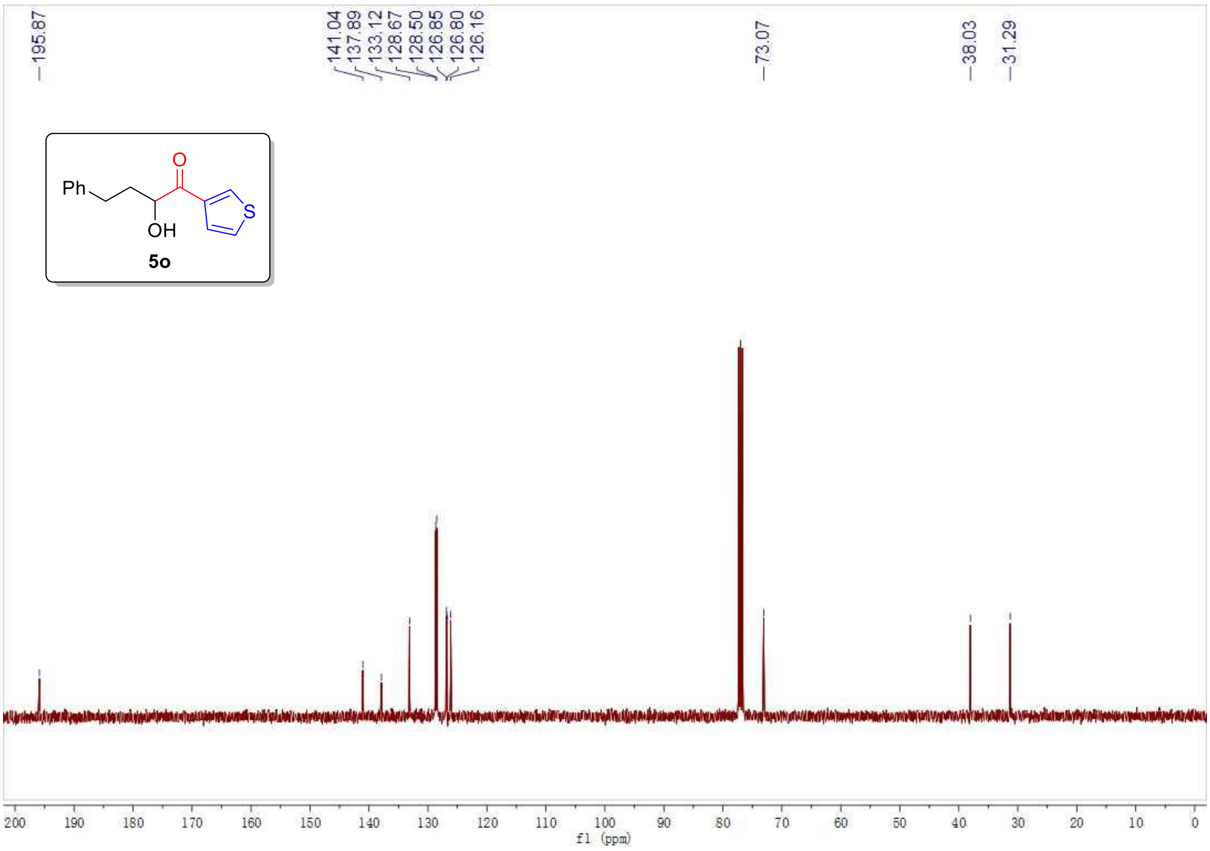

^{1}H NMR (400 MHz, $CDCl_3$) spectrum of 5

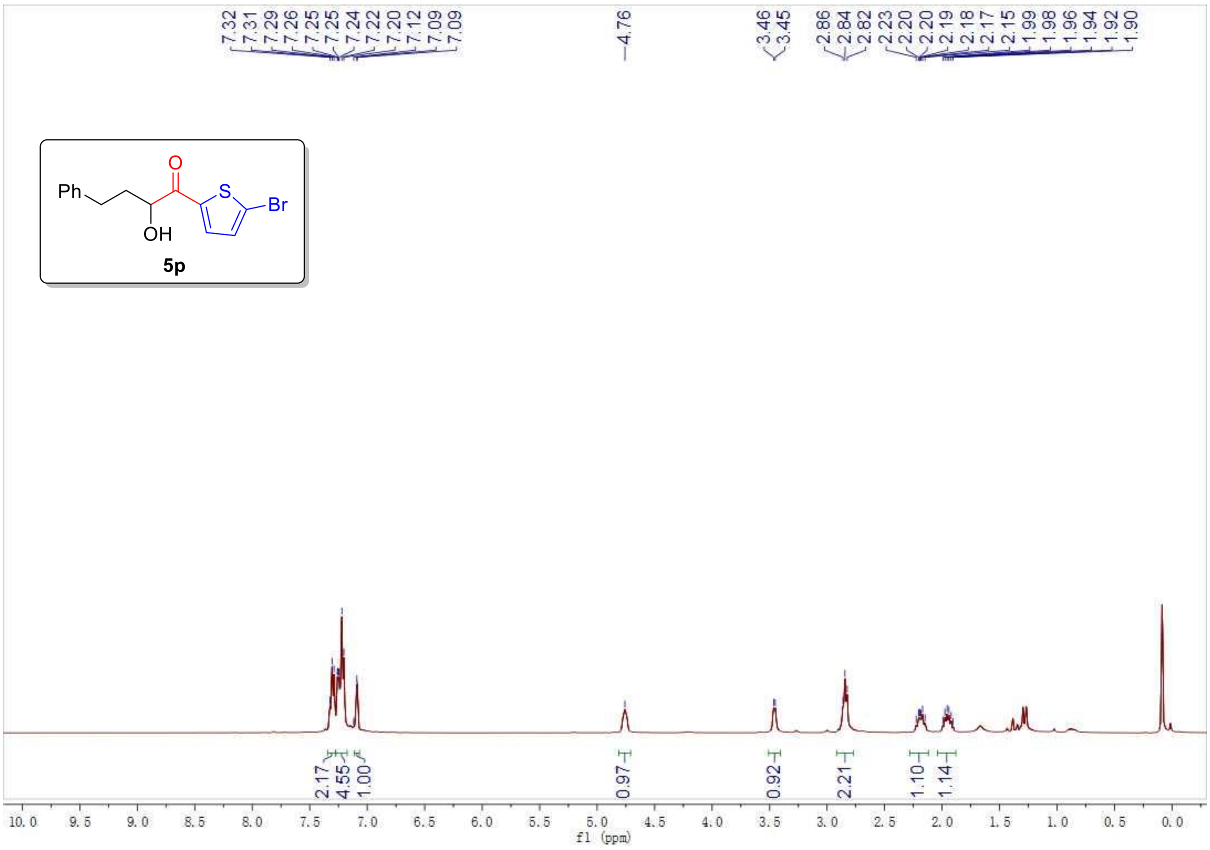

^{13}C NMR (101 MHz, $CDCl_3$) spectrum of 5p

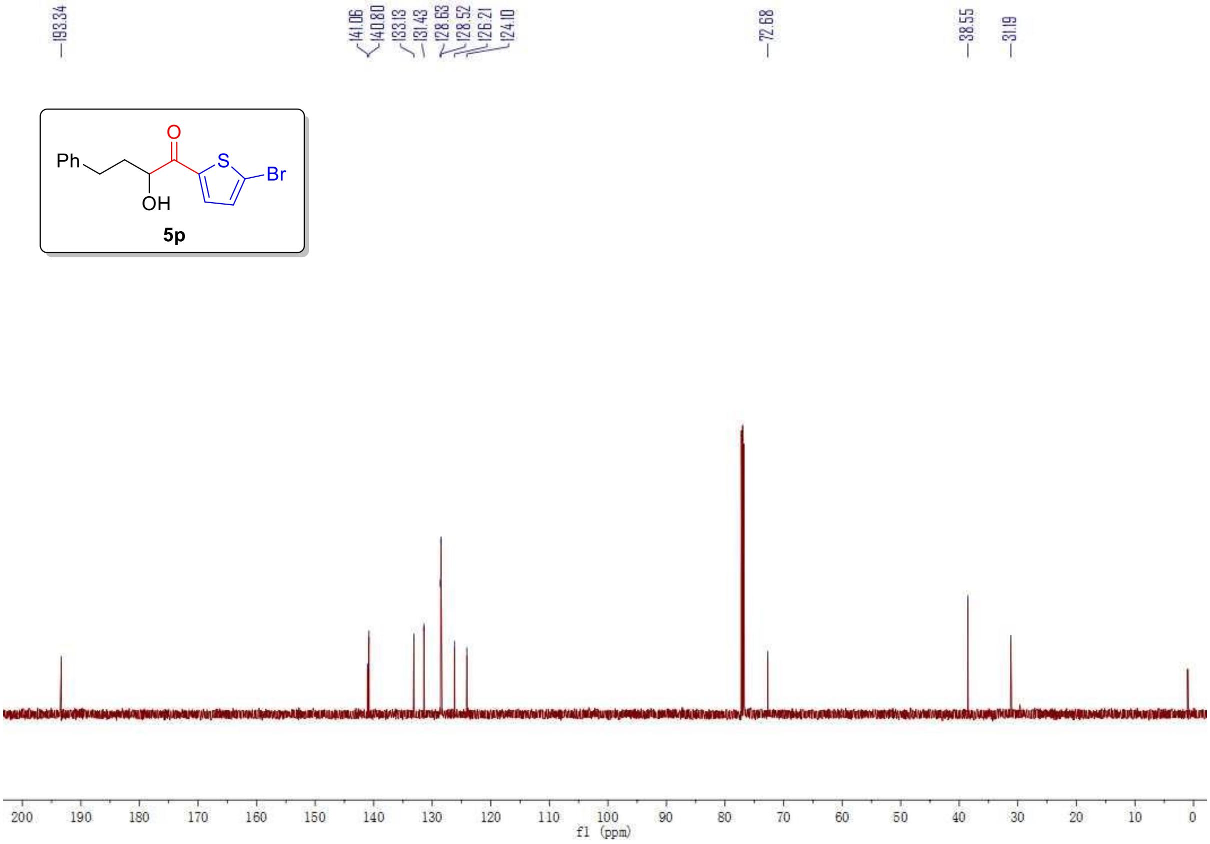

^{1}H NMR (400 MHz, $CDCl_3$) spectrum of 5q

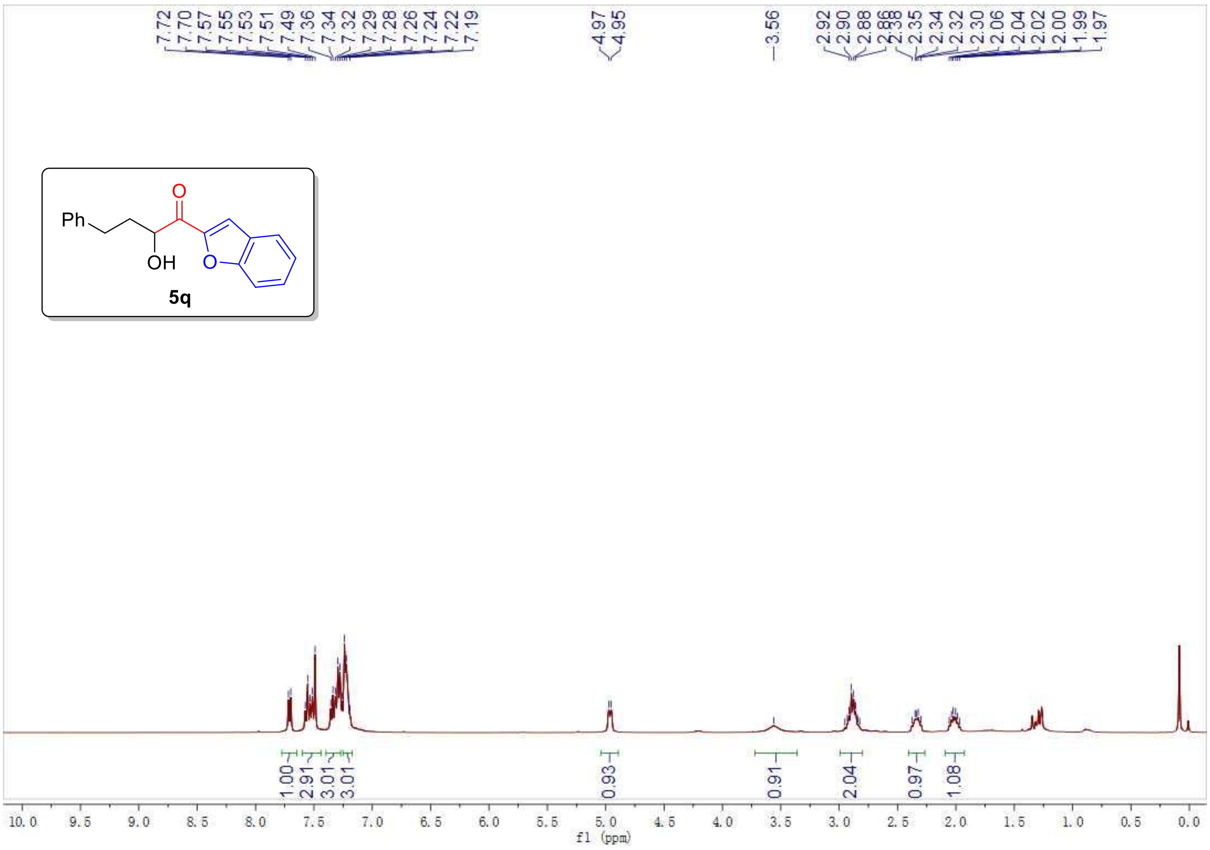

^{13}C NMR (101 MHz, $CDCl_3$) spectrum of 5q

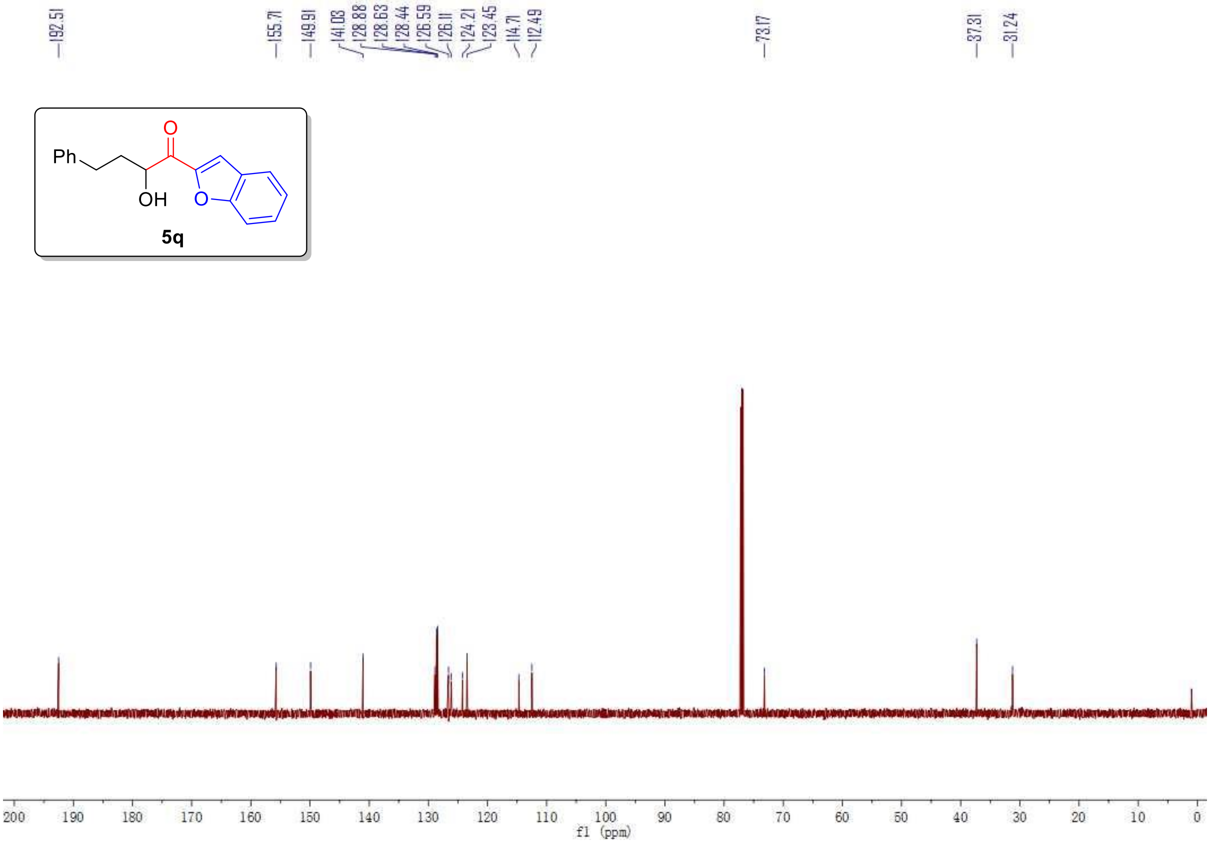

^{1}H NMR (400 MHz, $CDCl_3$) spectrum of 5r

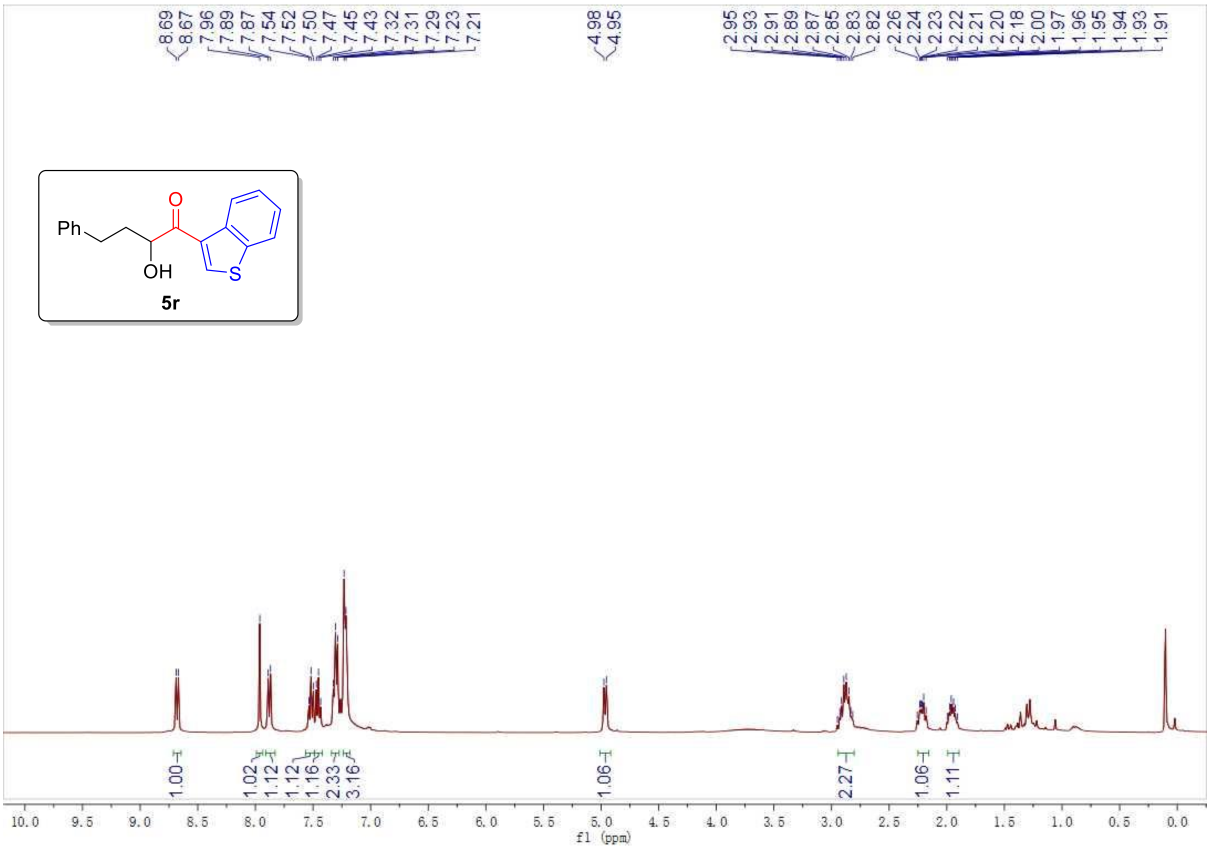

^{13}C NMR (101 MHz, $CDCl_3$) spectrum of 5r

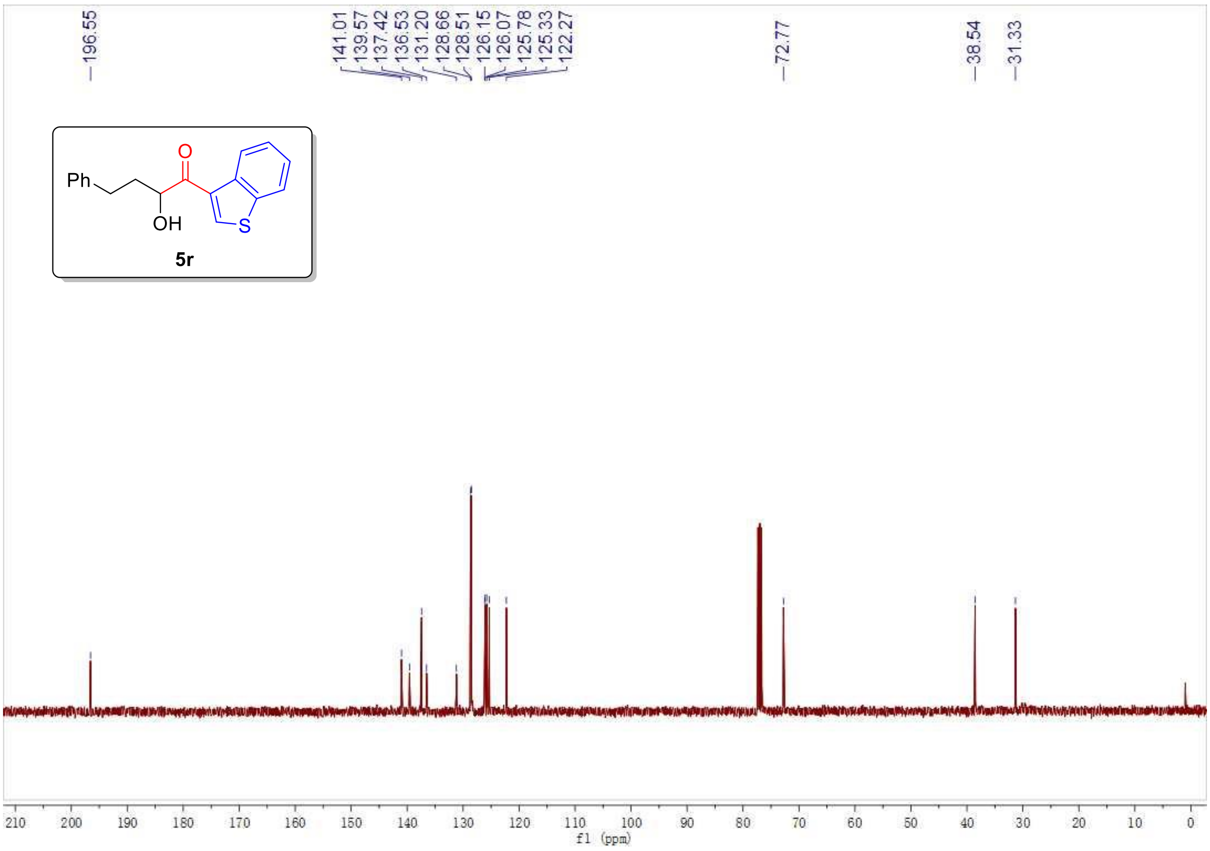

^{1}H NMR (400 MHz, $CDCl_3$) spectrum of 5s

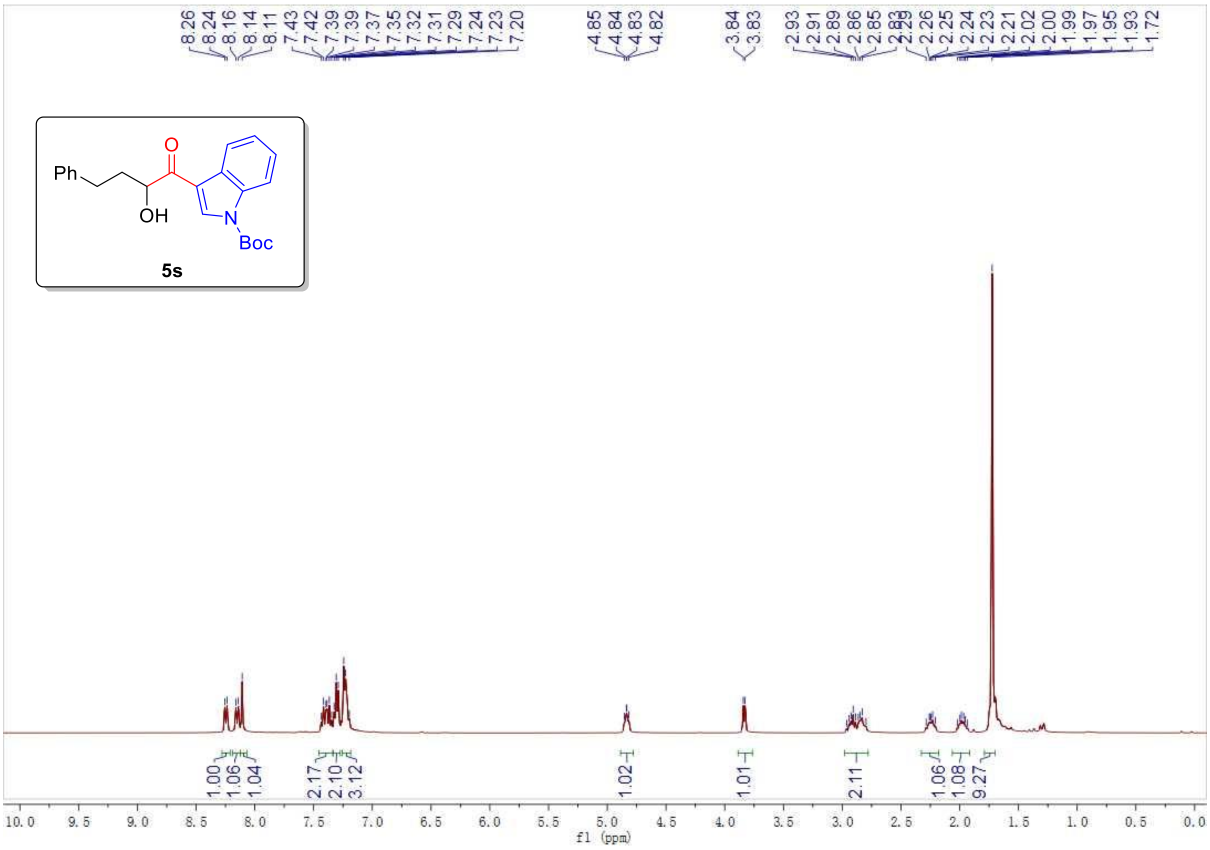

^{13}C NMR (101 MHz, $CDCl_3$) spectrum of 5s

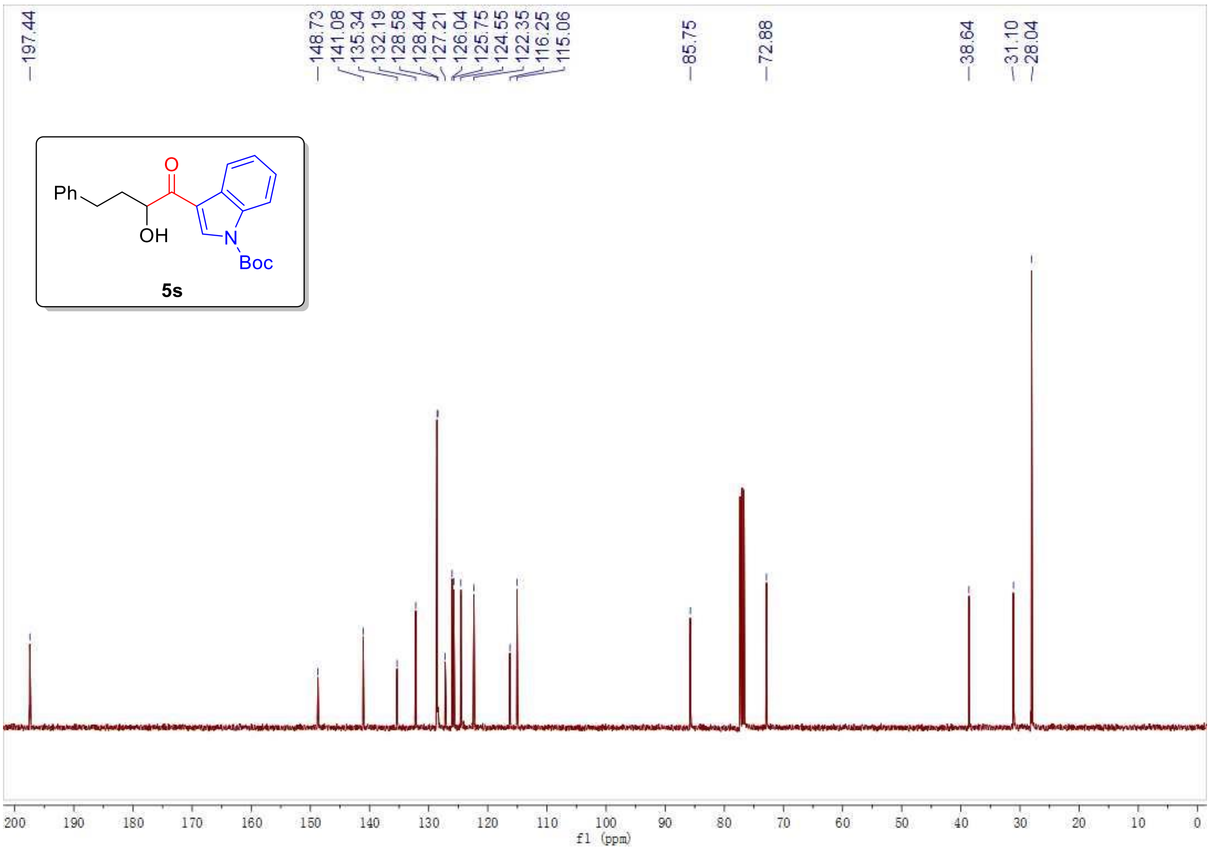

^{1}H NMR (400 MHz, $CDCl_3$) spectrum of 5t

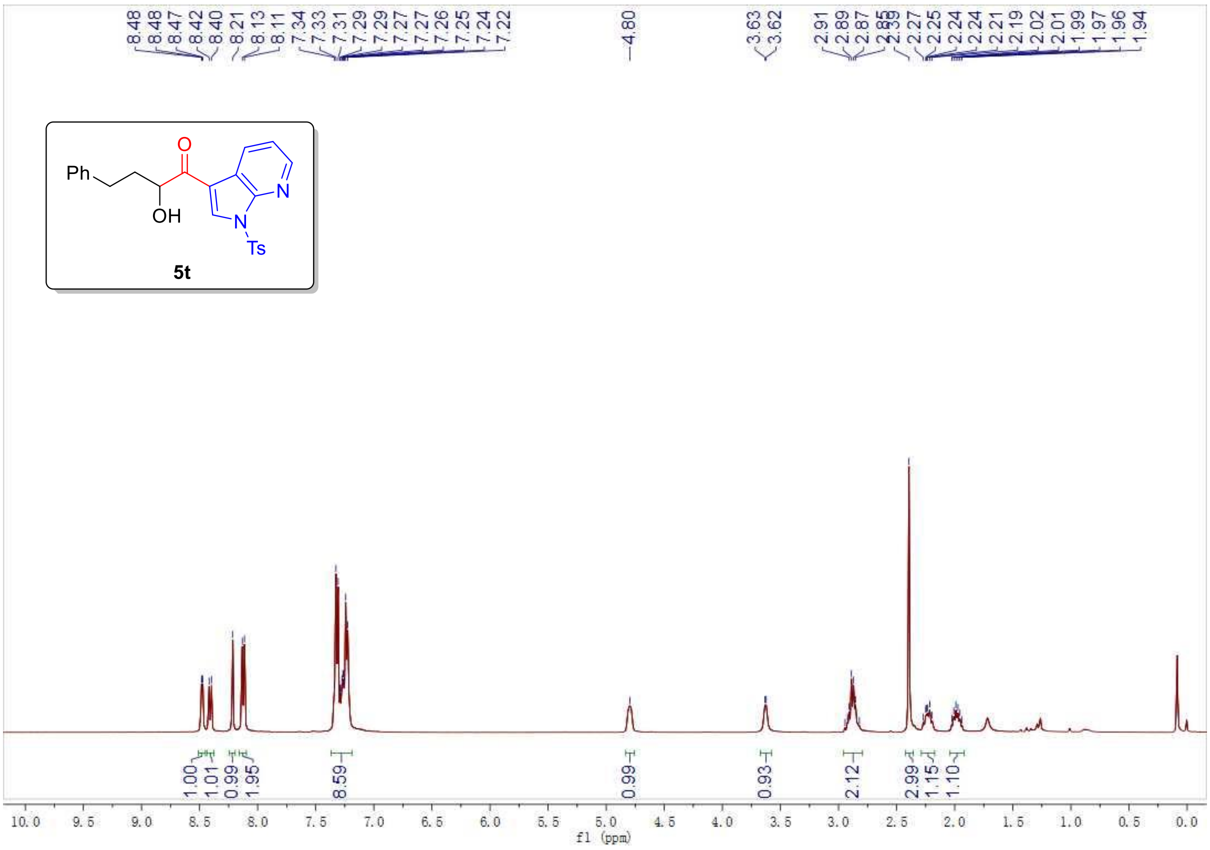

^{13}C NMR (101 MHz, $CDCl_3$) spectrum of 5t

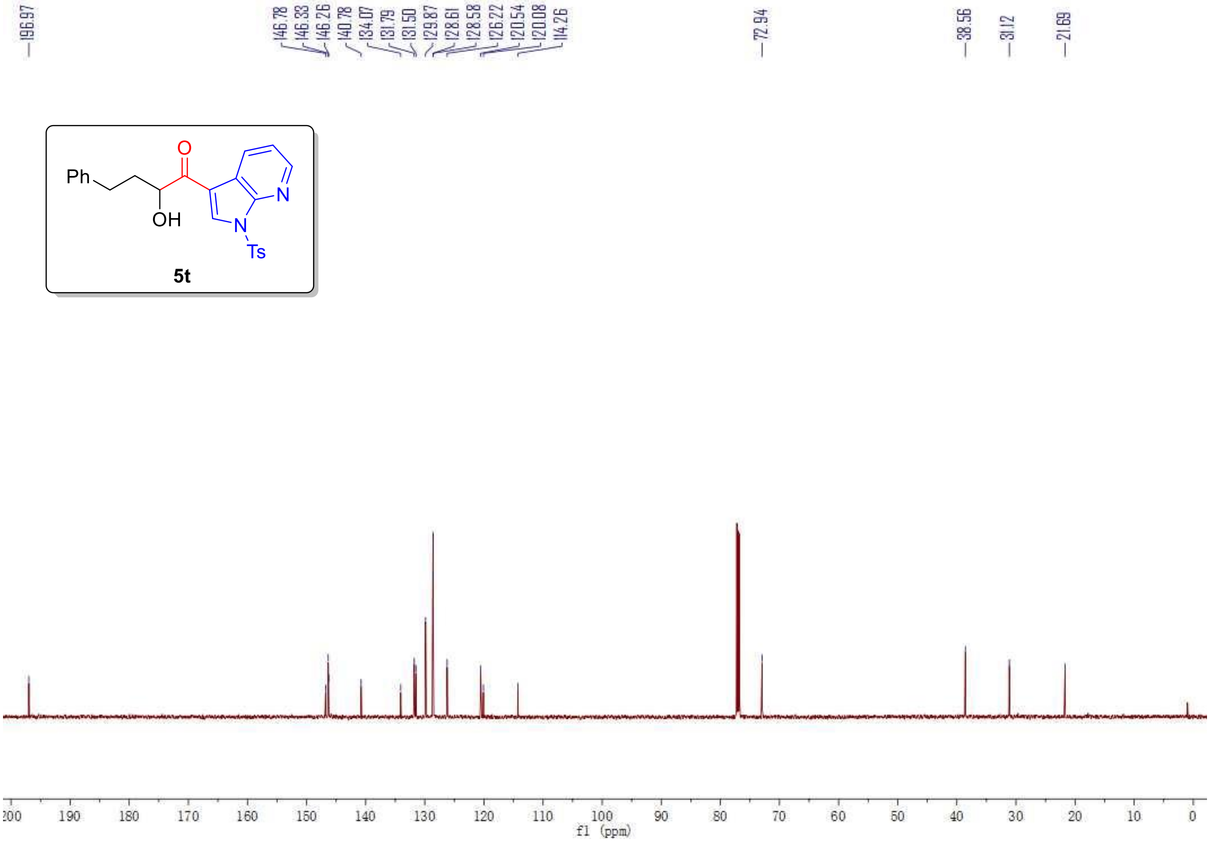

^{1}H NMR (400 MHz, $CDCl_3$) spectrum of 5u

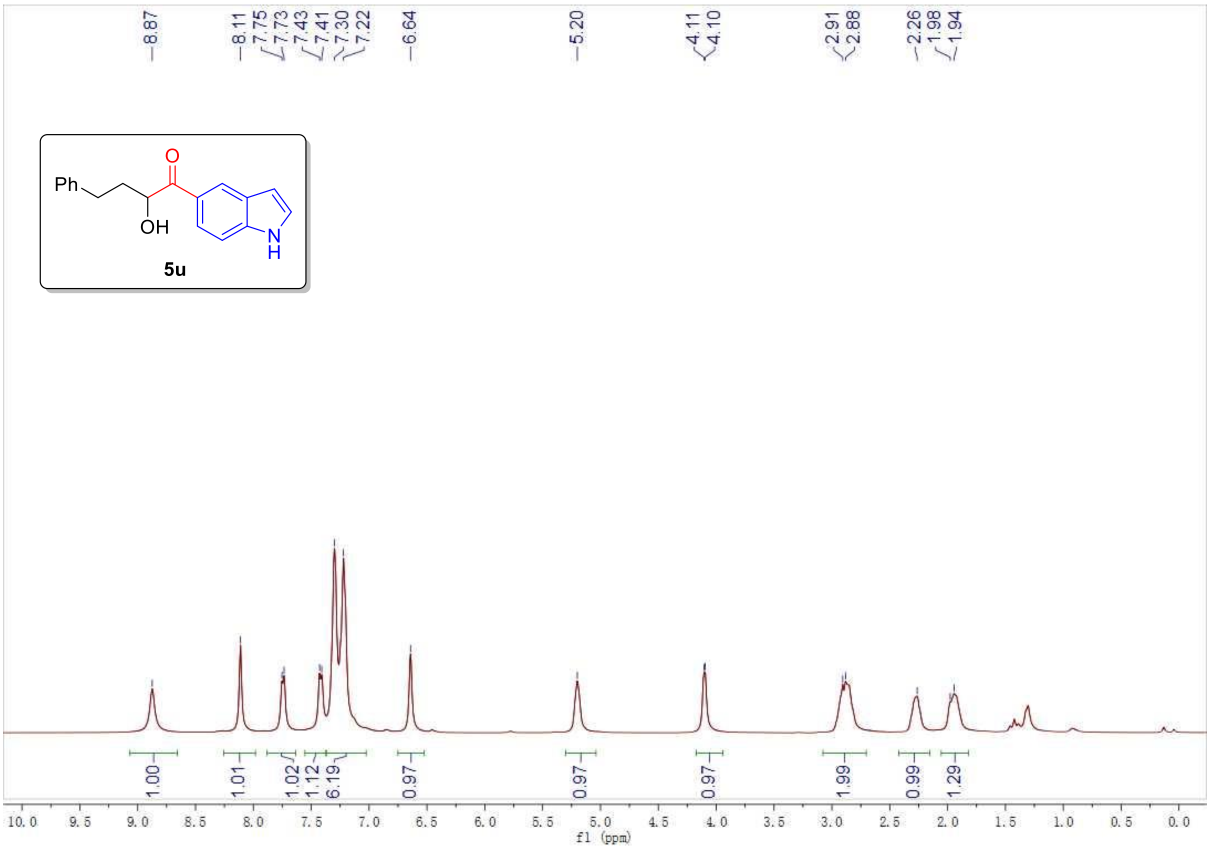

^{13}C NMR (101 MHz, $CDCl_3$) spectrum of 5u

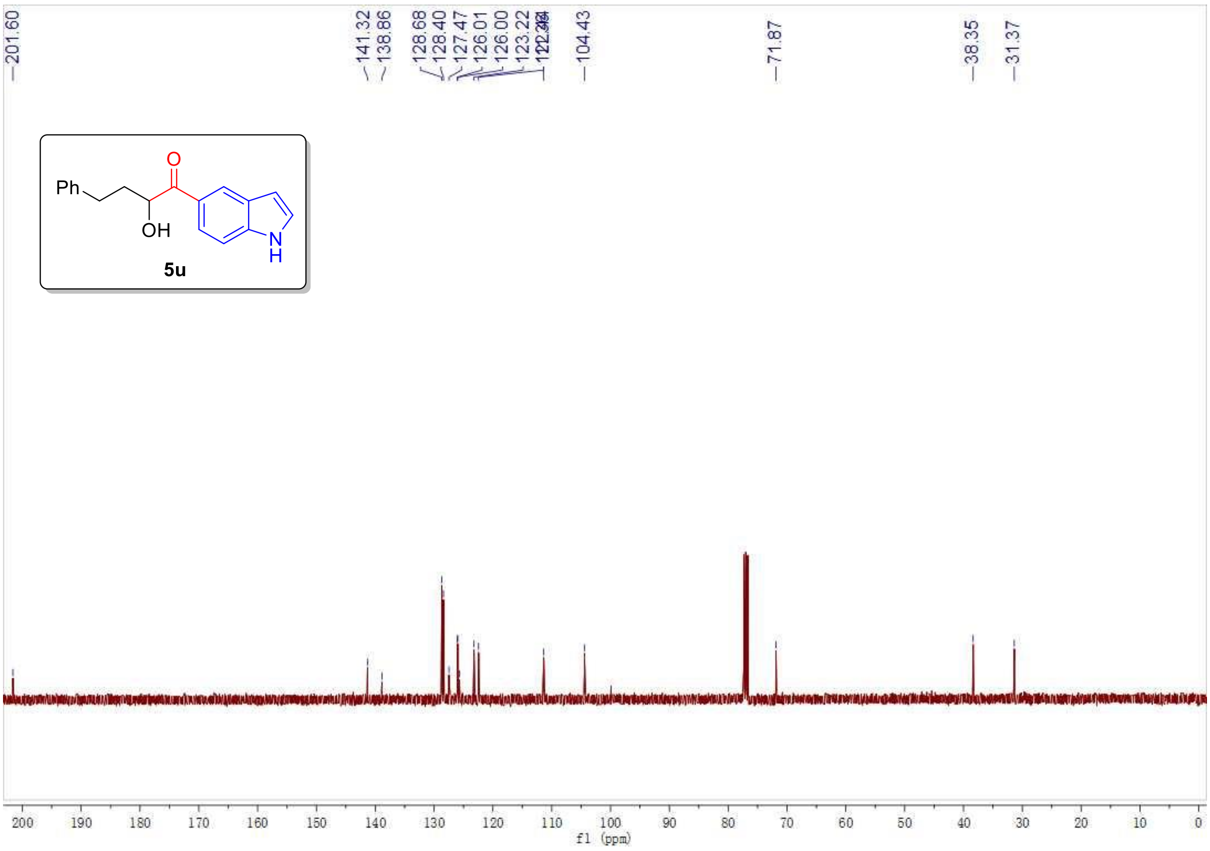

^{1}H NMR (400 MHz, $CDCl_3$) spectrum of 5v

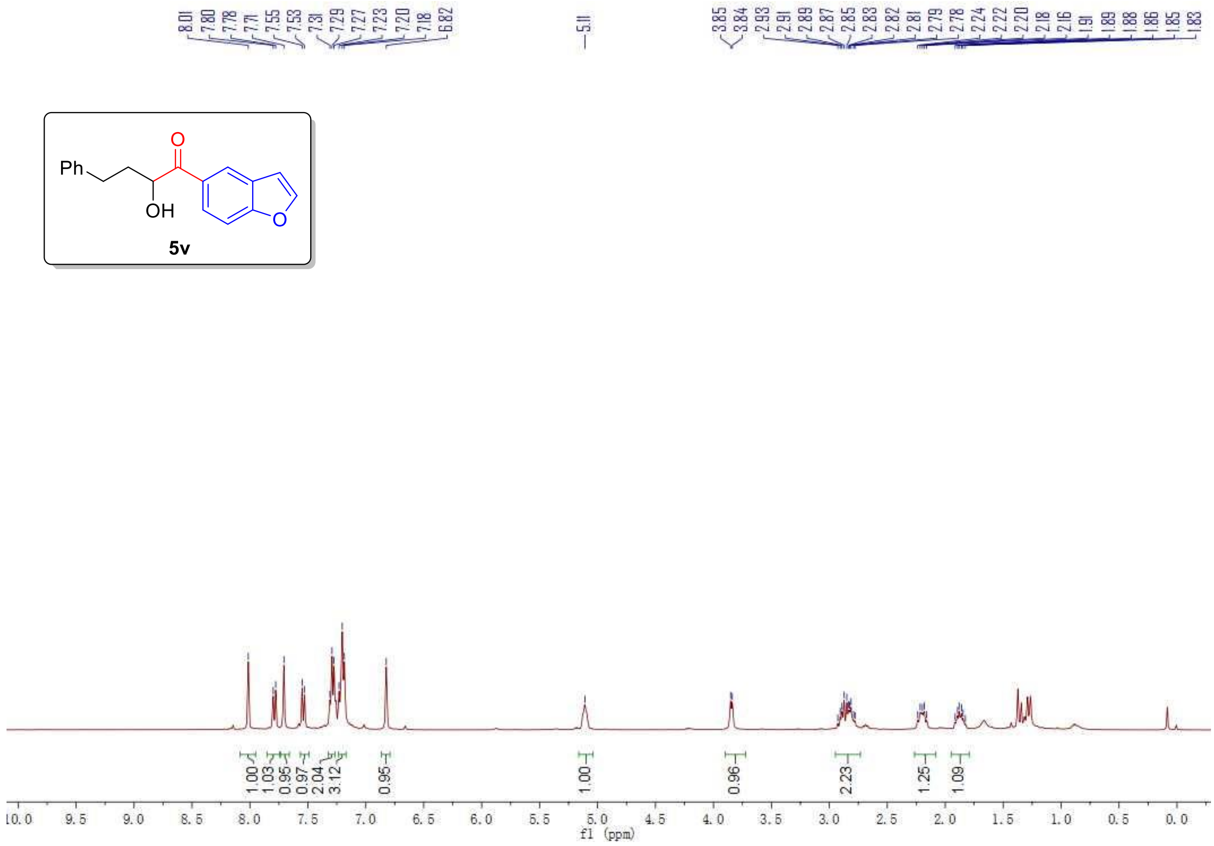

^{13}C NMR (101 MHz, $CDCl_3$) spectrum of 5v

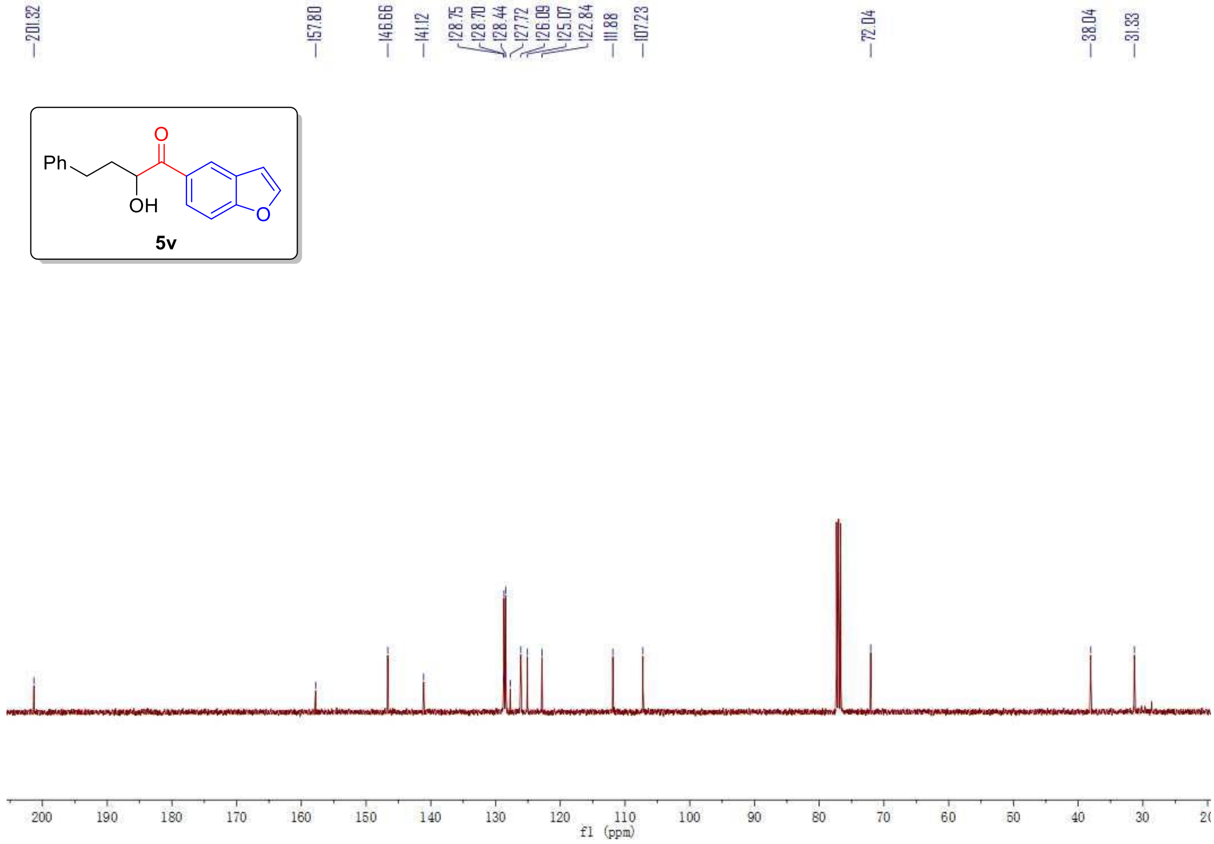

^{1}H NMR (400 MHz, $CDCl_3$) spectrum of 5w

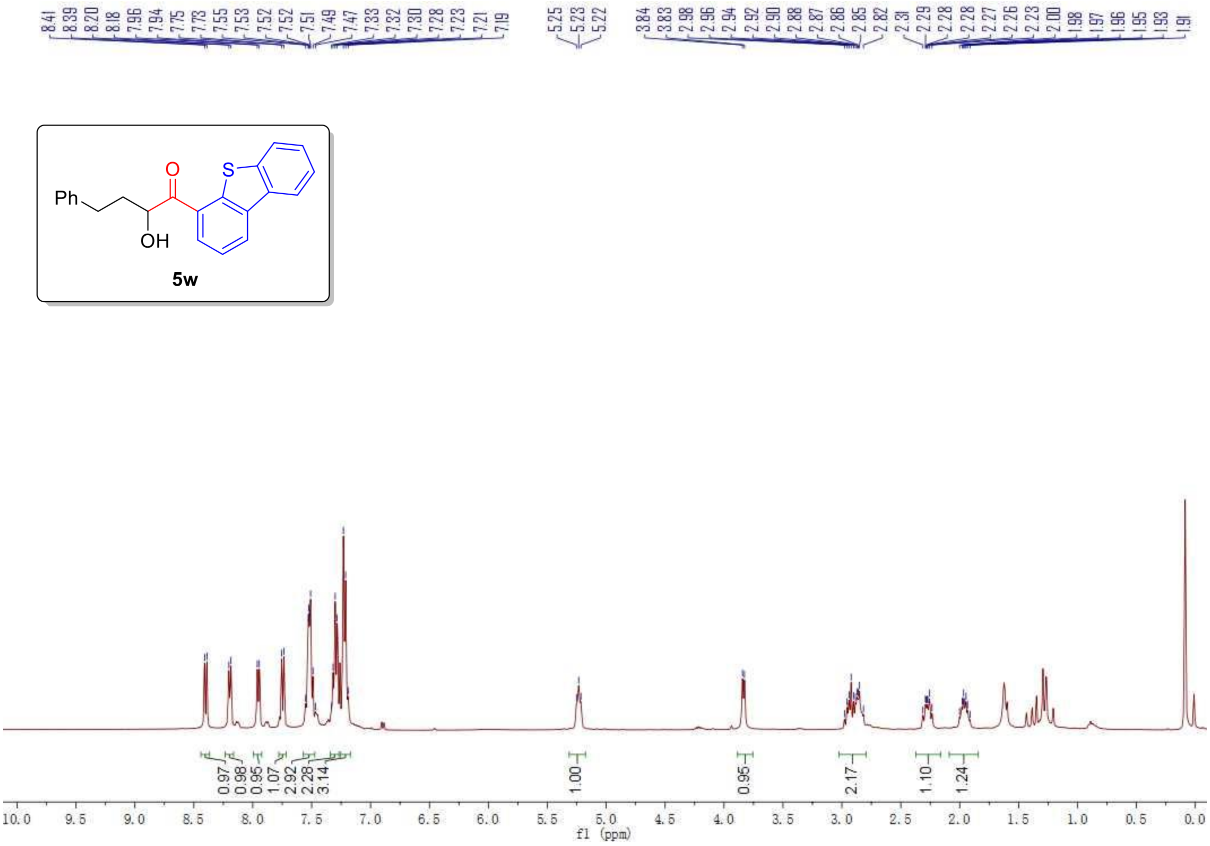

^{13}C NMR (101 MHz, $CDCl_3$) spectrum of 5w

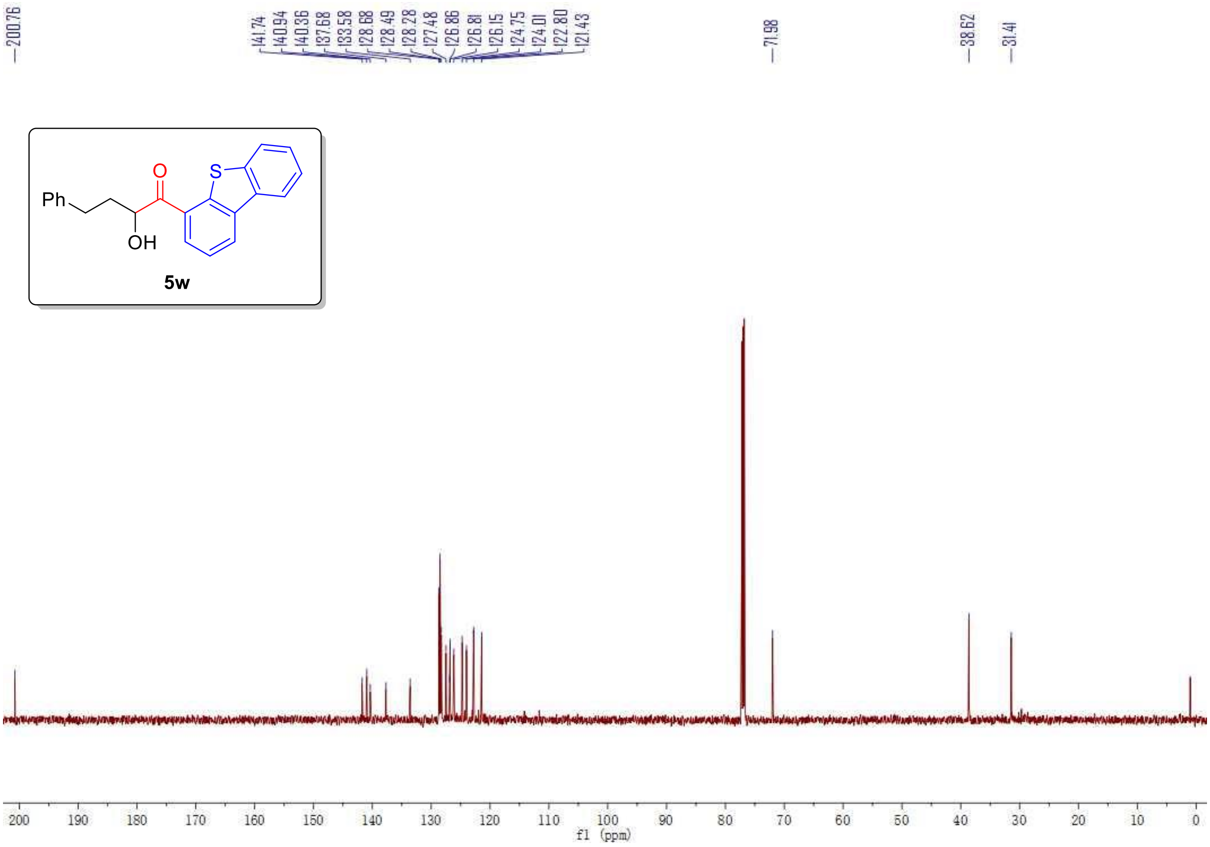

^{1}H NMR (400 MHz, $CDCl_3$) spectrum of 5x

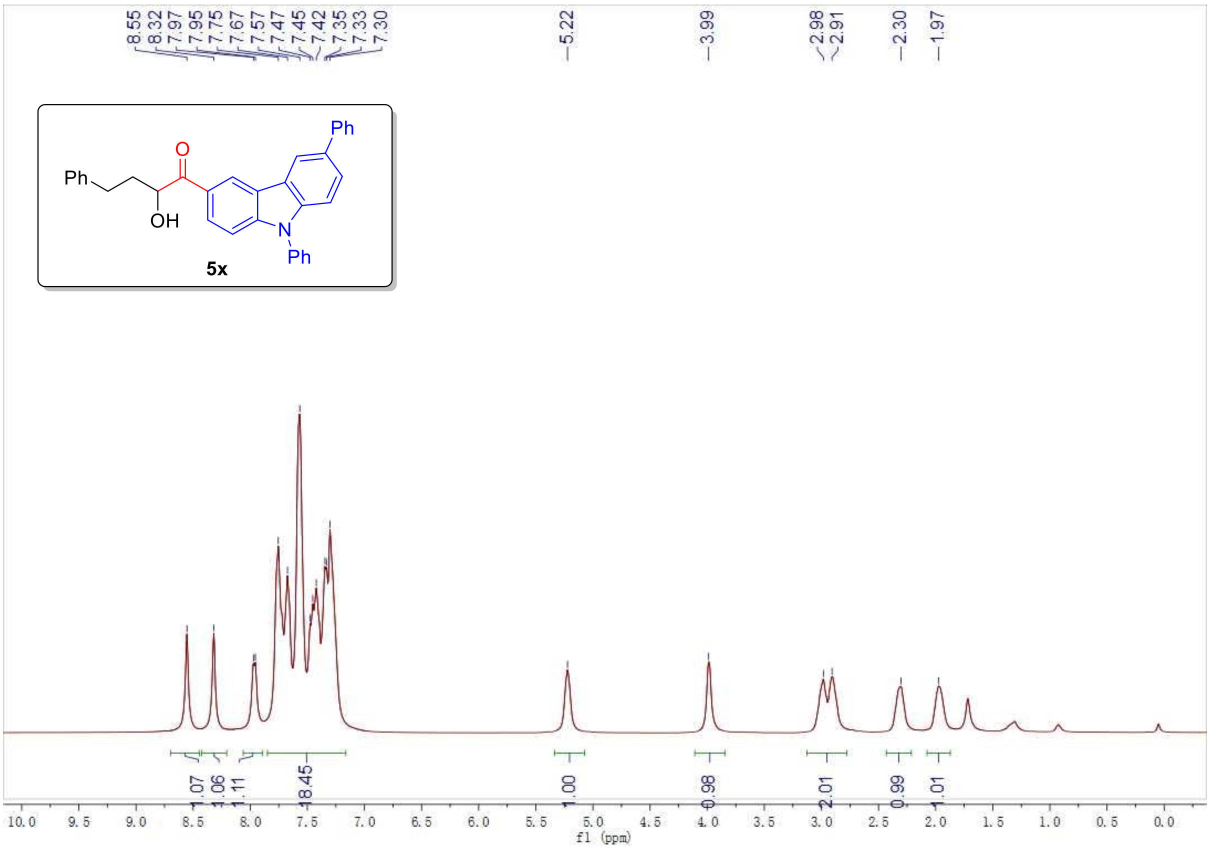

^{13}C NMR (101 MHz, $CDCl_3$) spectrum of 5x

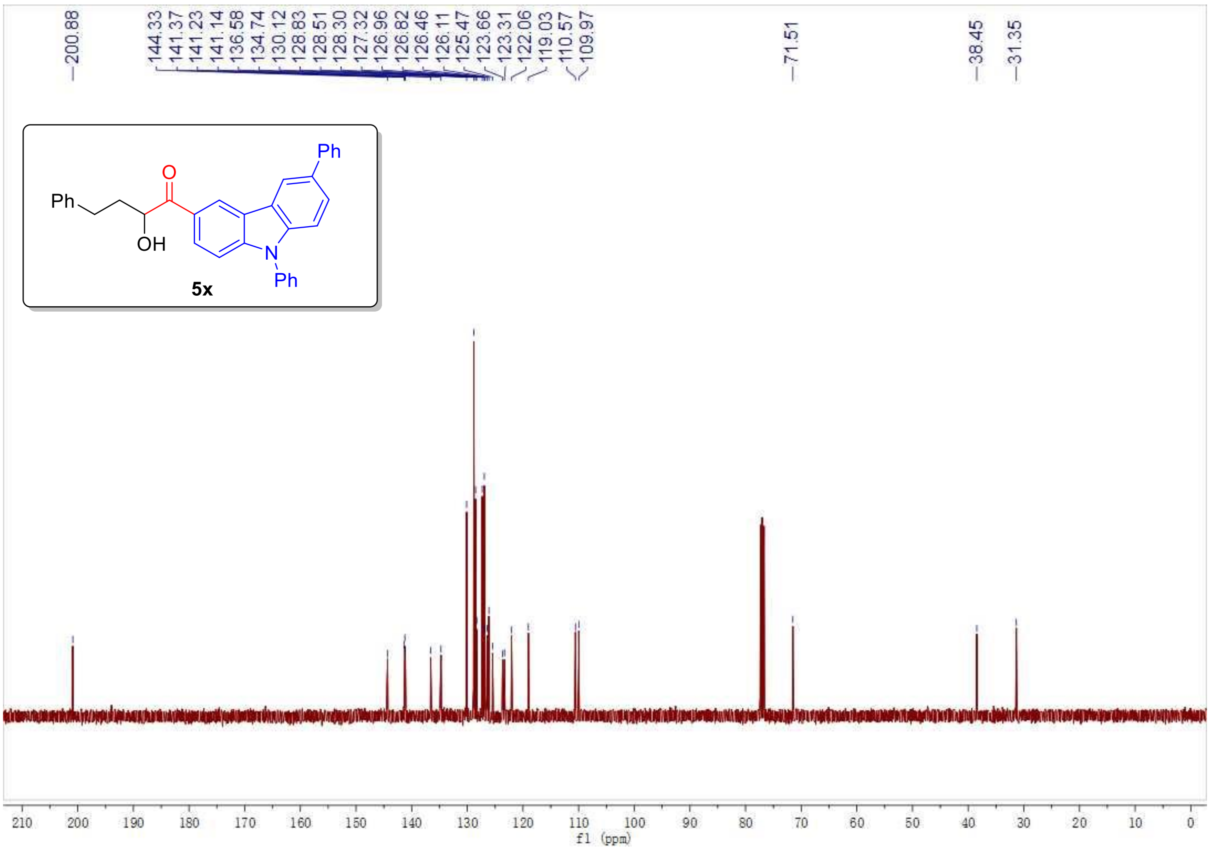

^{1}H NMR (500 MHz, $CDCl_3$) spectrum of 5y

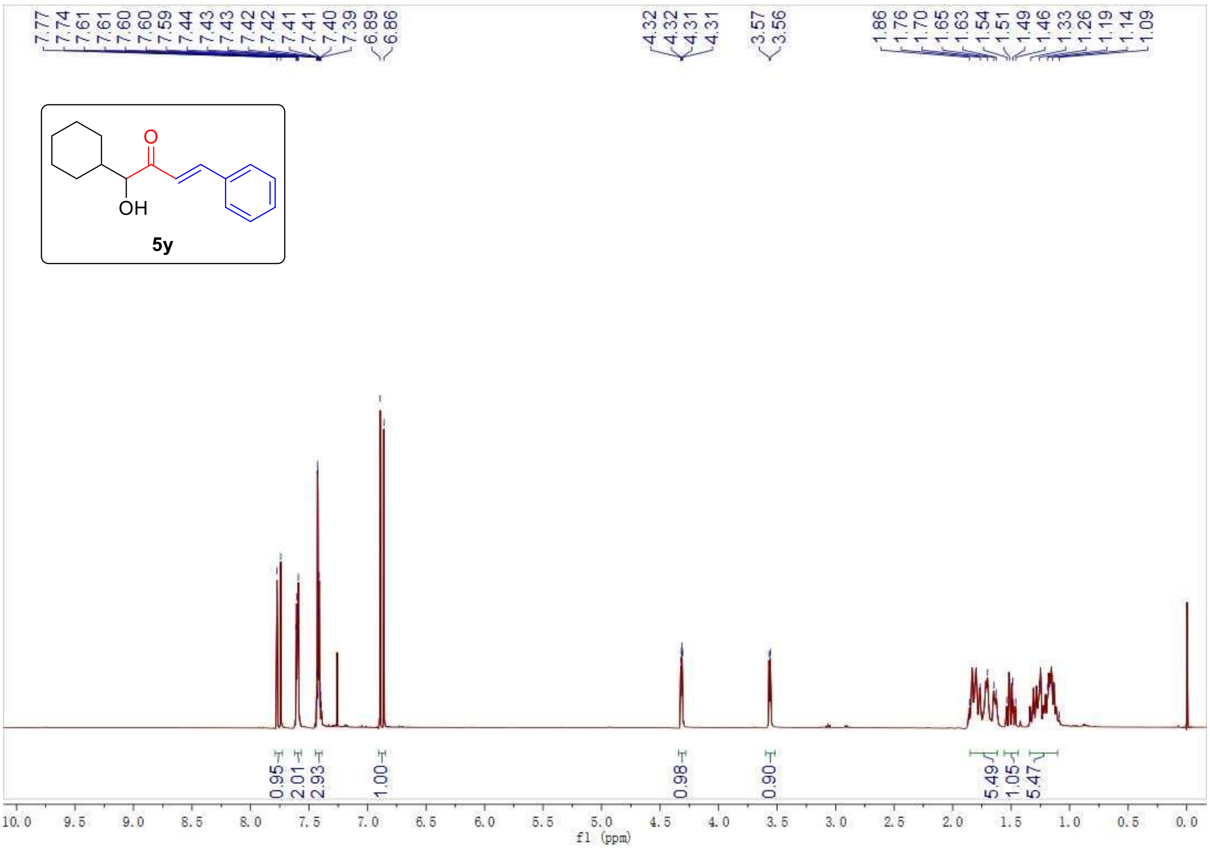

^{13}C NMR (126 MHz, $CDCl_3$) spectrum of 5y

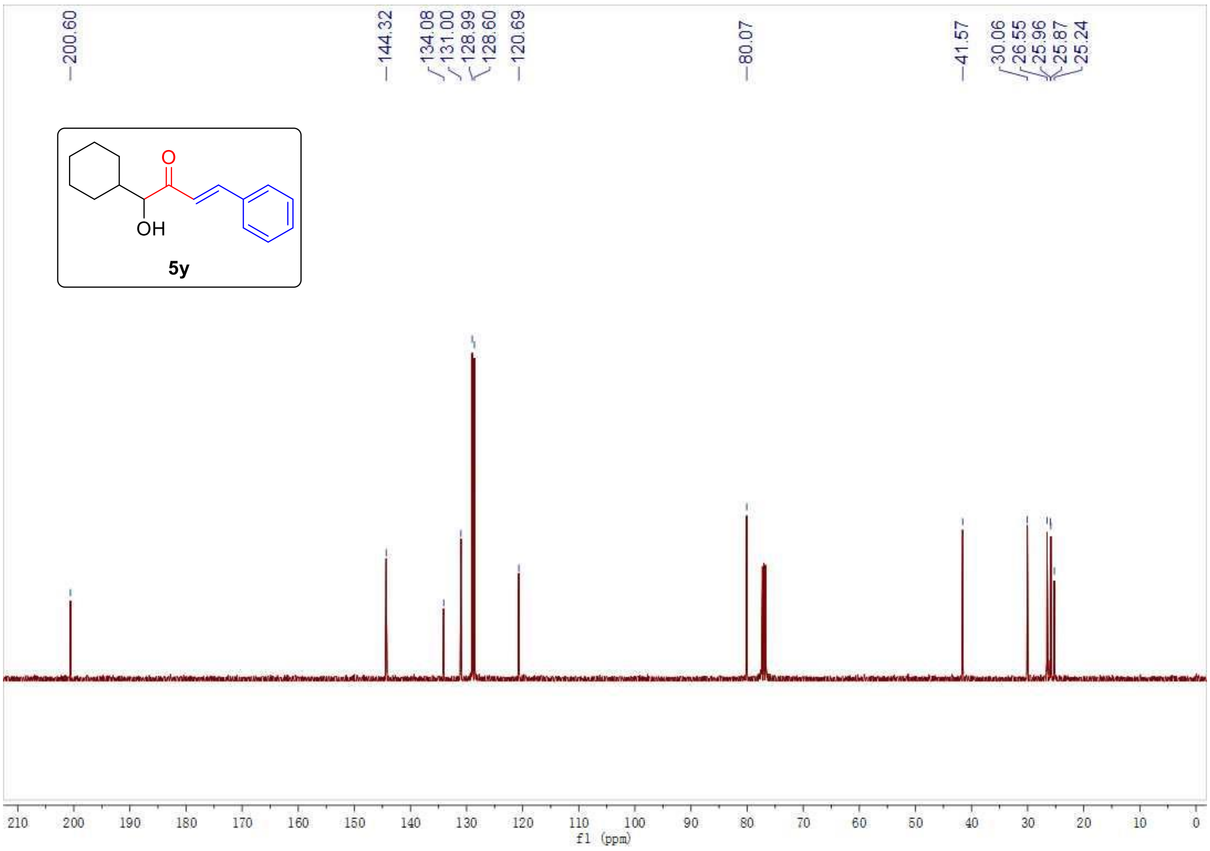

^{1}H NMR (500 MHz, $CDCl_3$) spectrum of 5z

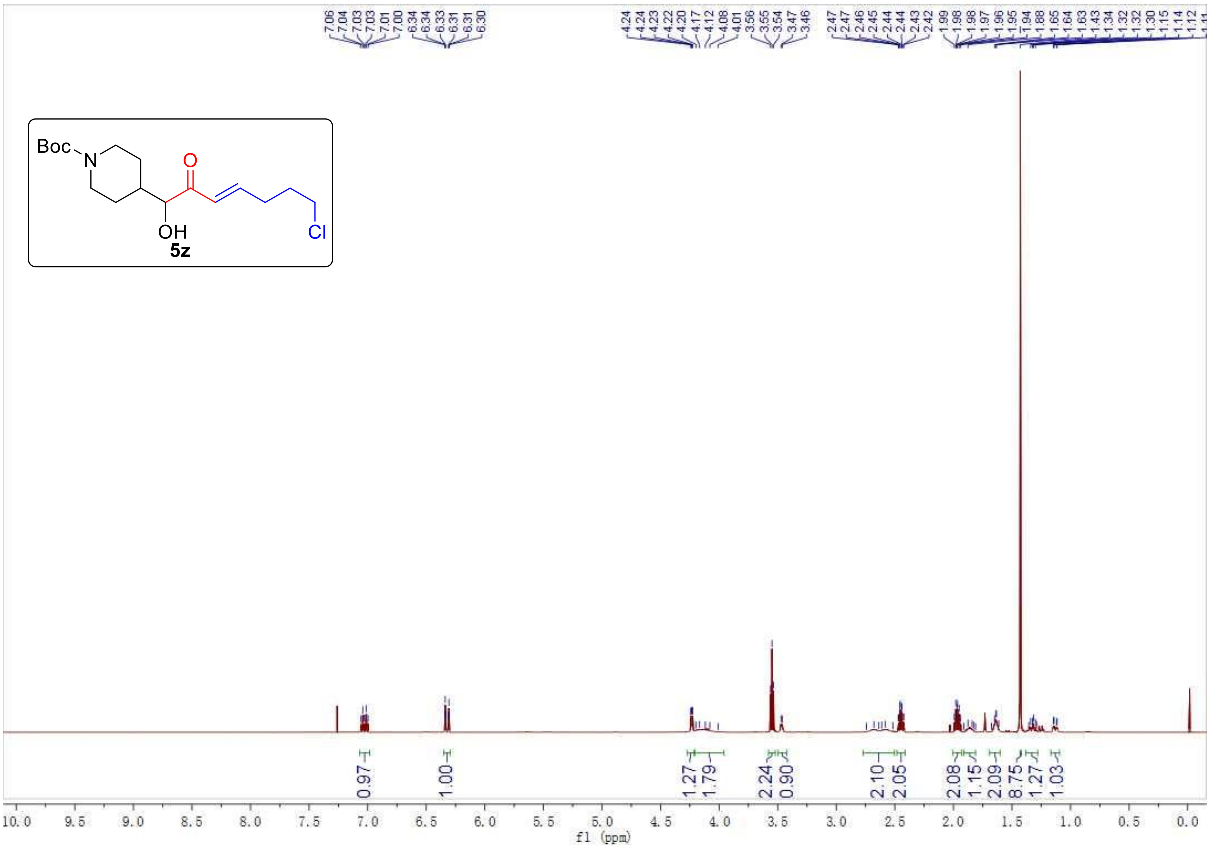

^{13}C NMR (126 MHz, $CDCl_3$) spectrum of 5z

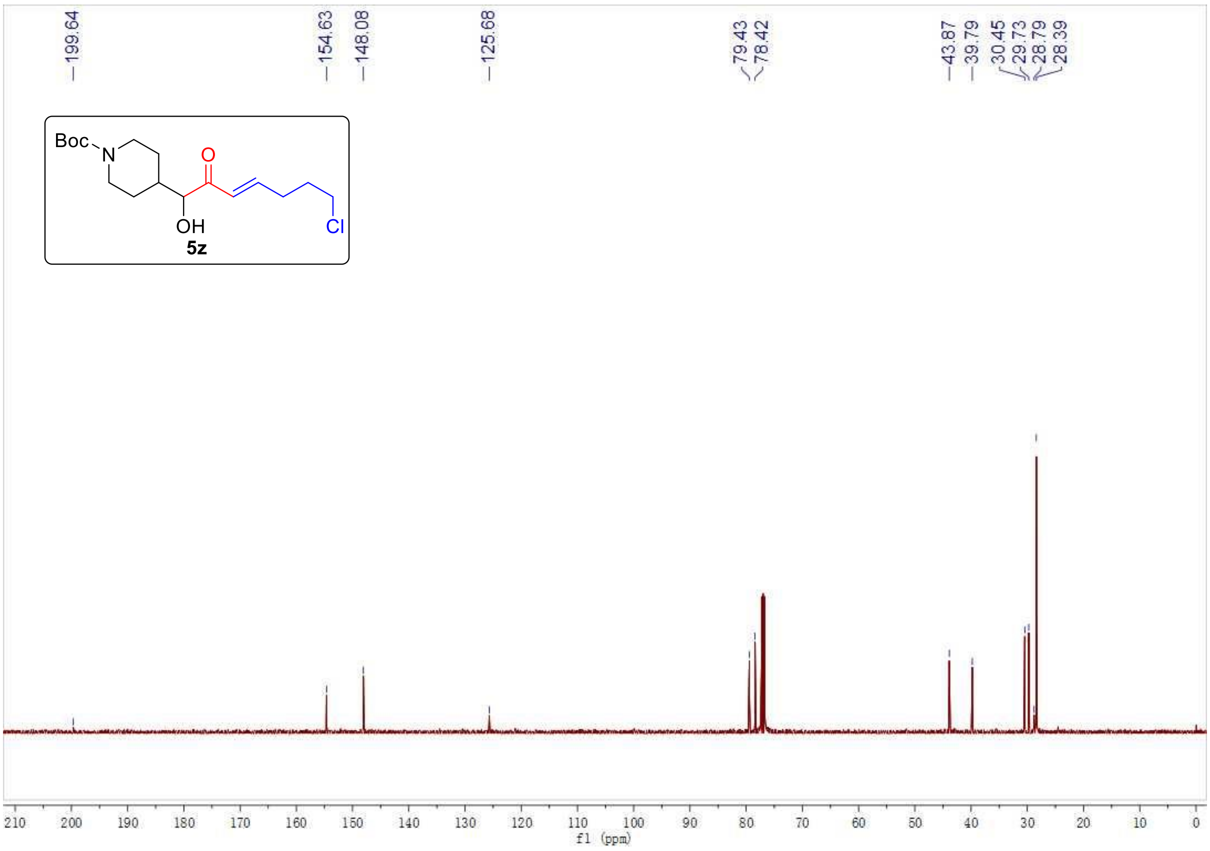

^{1}H NMR (500 MHz, $CDCl_3$) spectrum of 7a

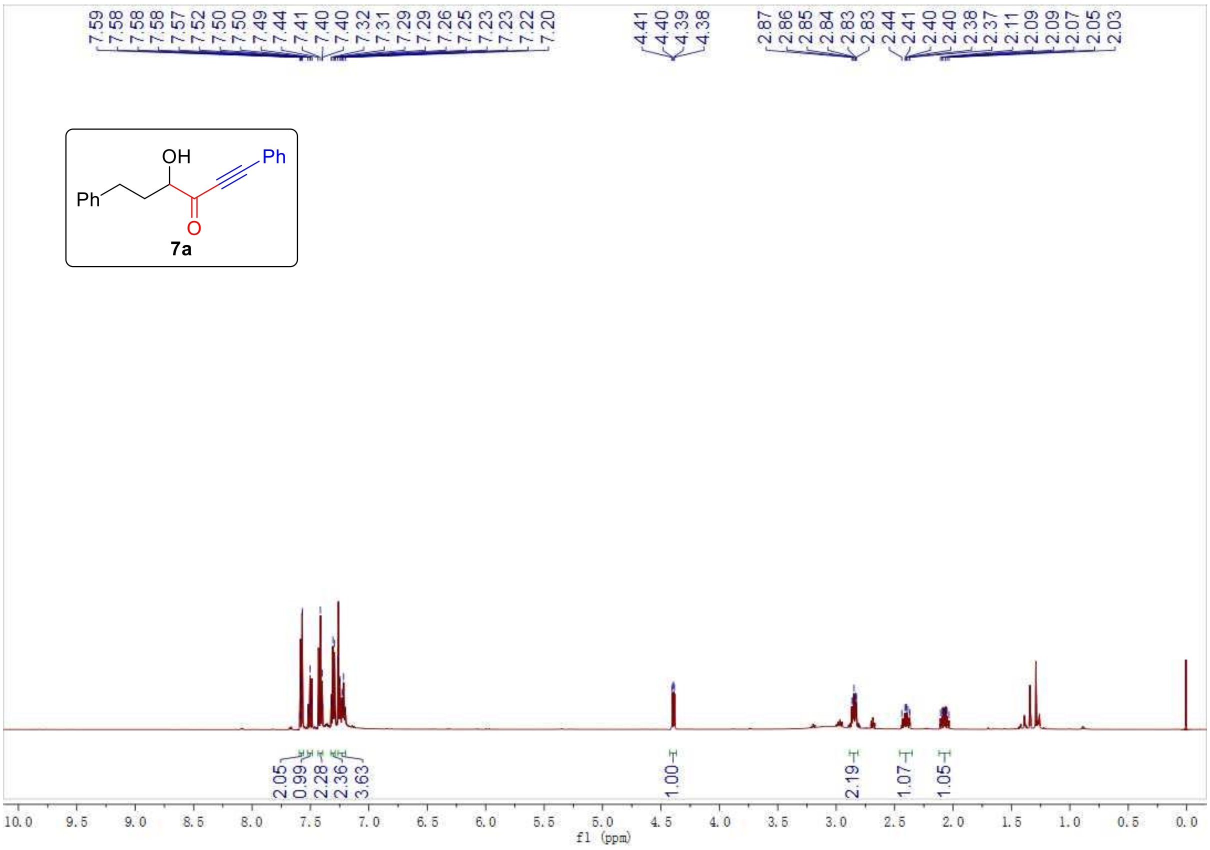

^{13}C NMR (126 MHz, $CDCl_3$) spectrum of 7a

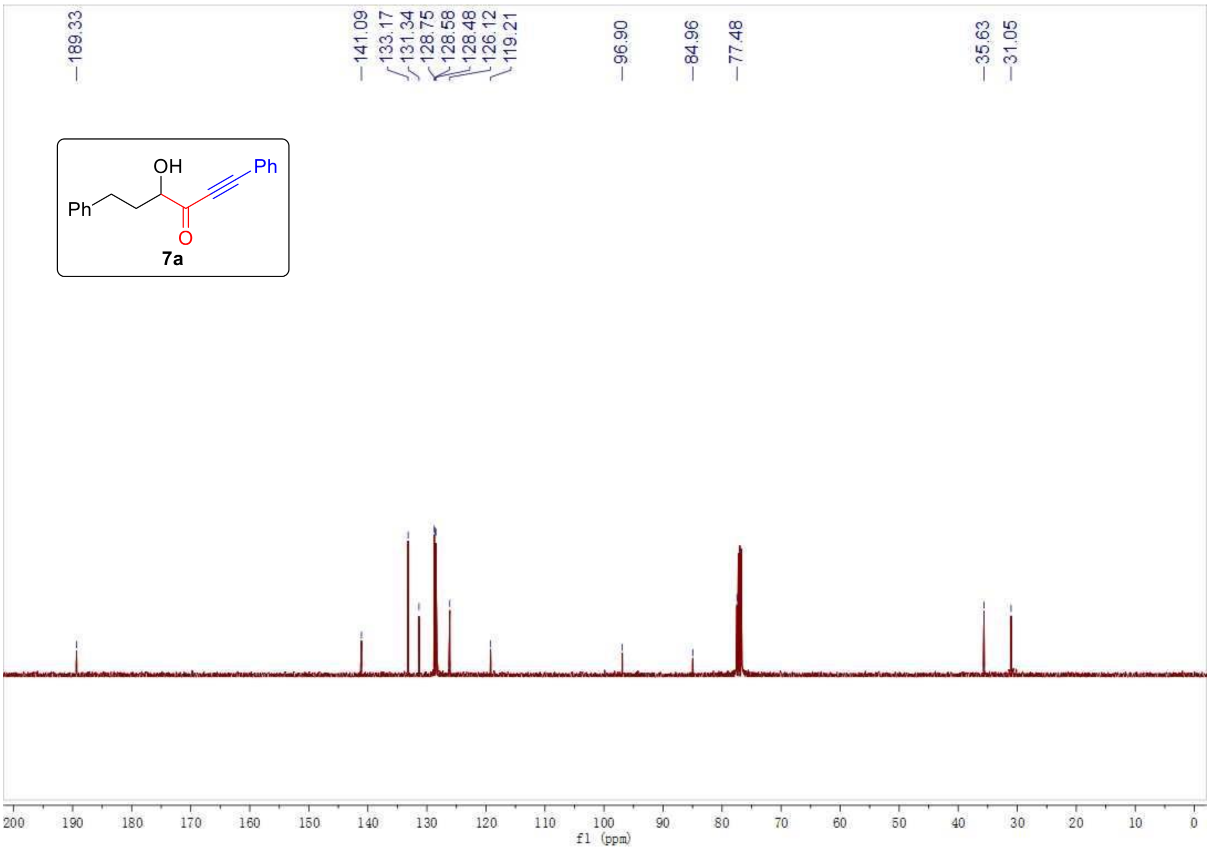

^{1}H NMR (500 MHz, $CDCl_3$) spectrum of 7b

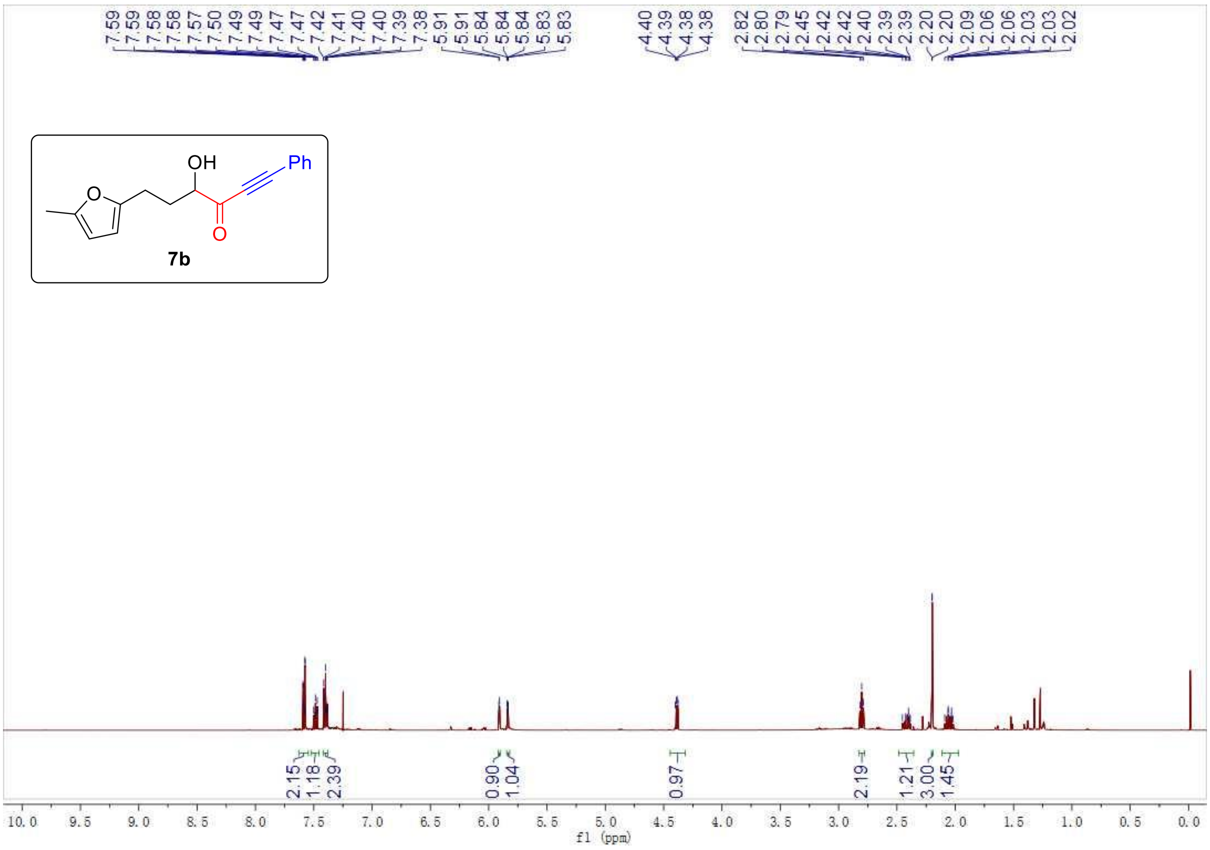

^{13}C NMR (126 MHz, $CDCl_3$) spectrum of 7b

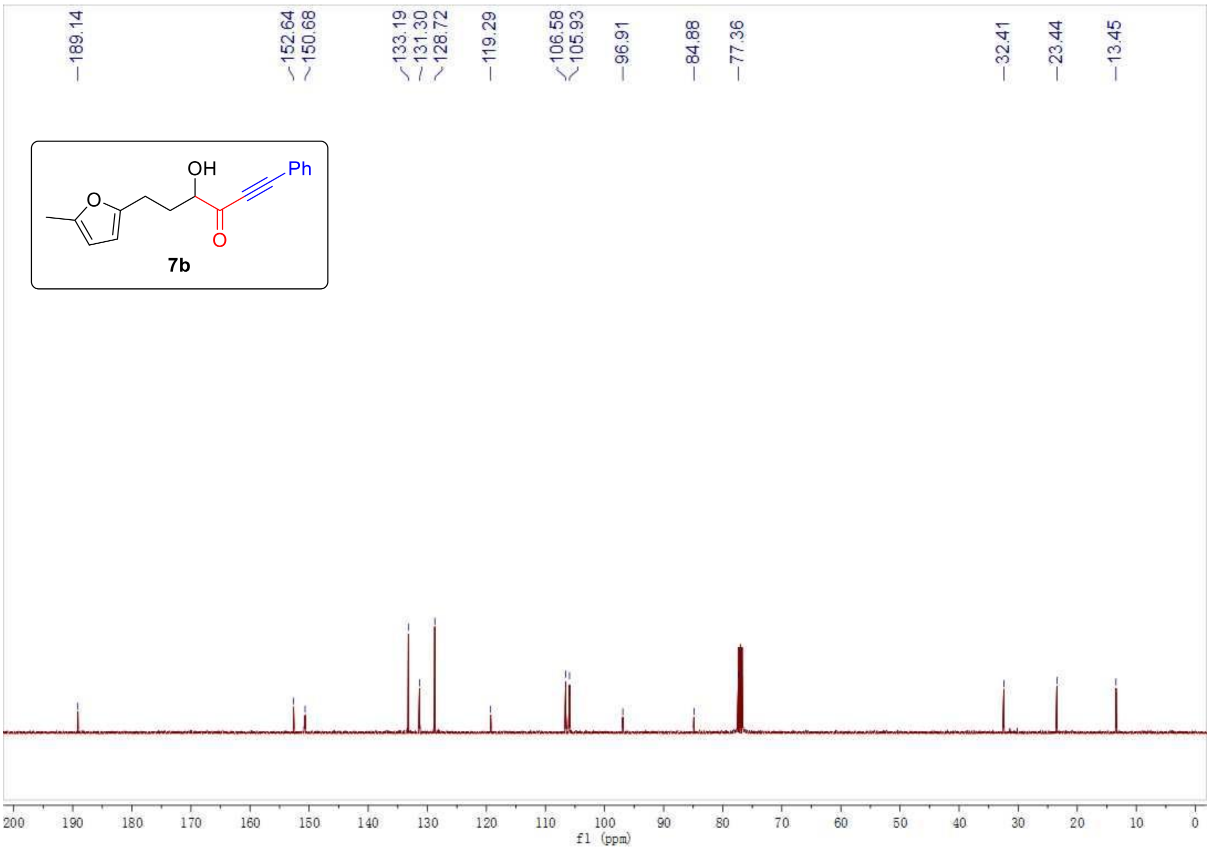

^{1}H NMR (500 MHz, $CDCl_3$) spectrum of 7c

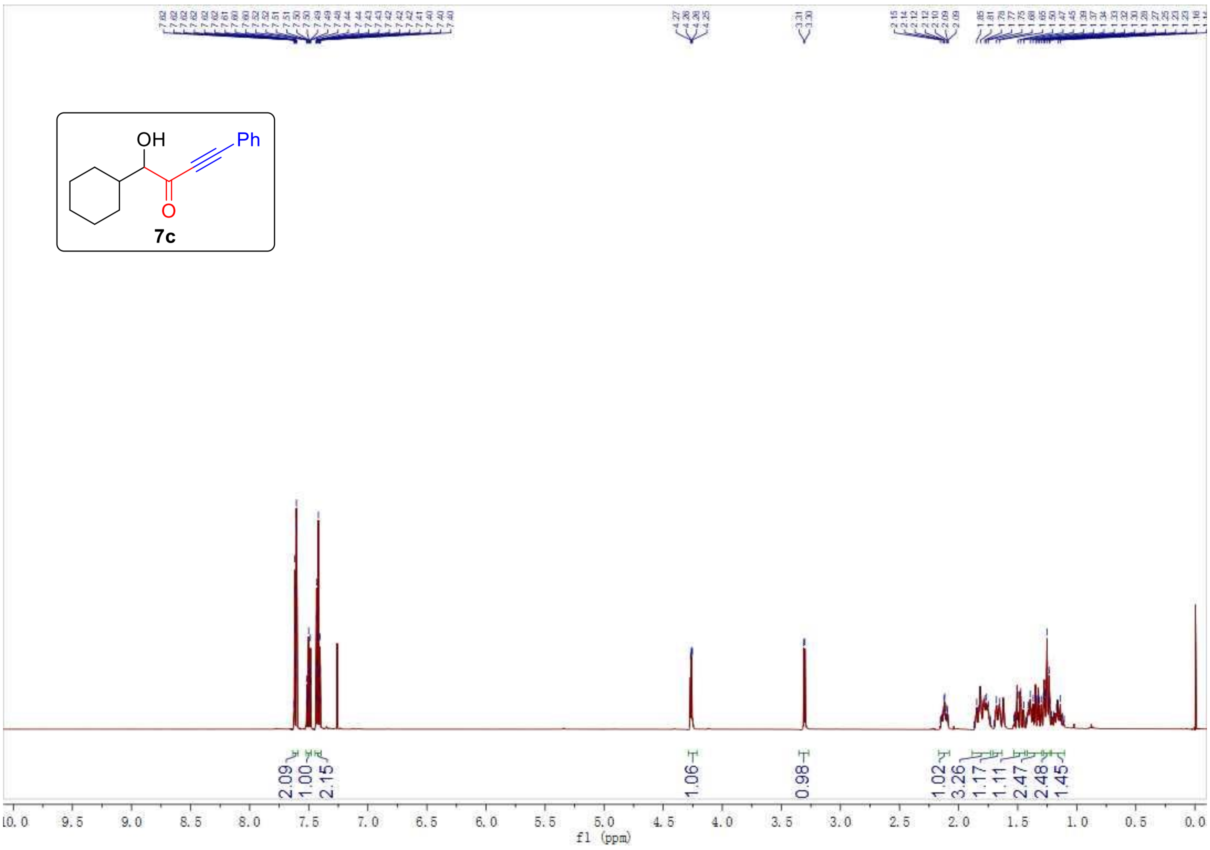

^{13}C NMR (126 MHz, $CDCl_3$) spectrum of 7c

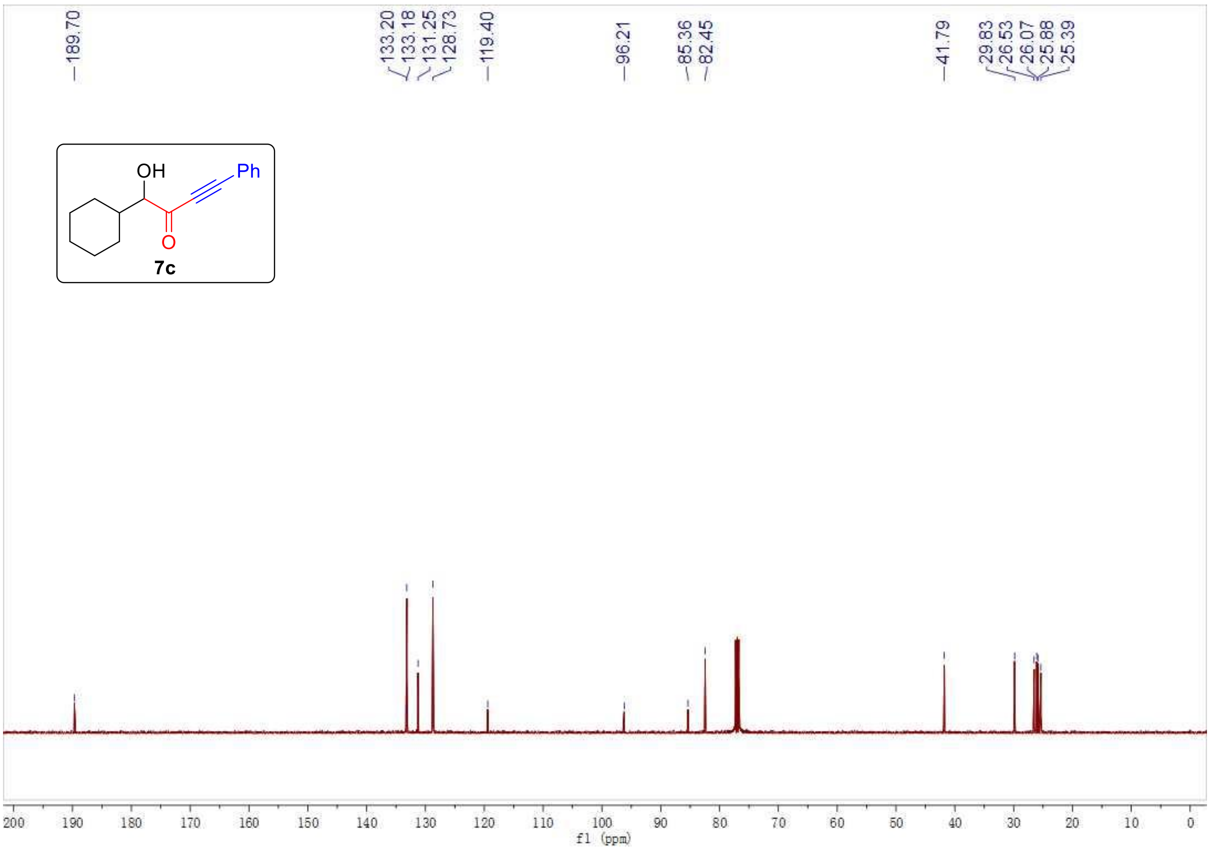

^{1}H NMR (500 MHz, $CDCl_3$) spectrum of 7d

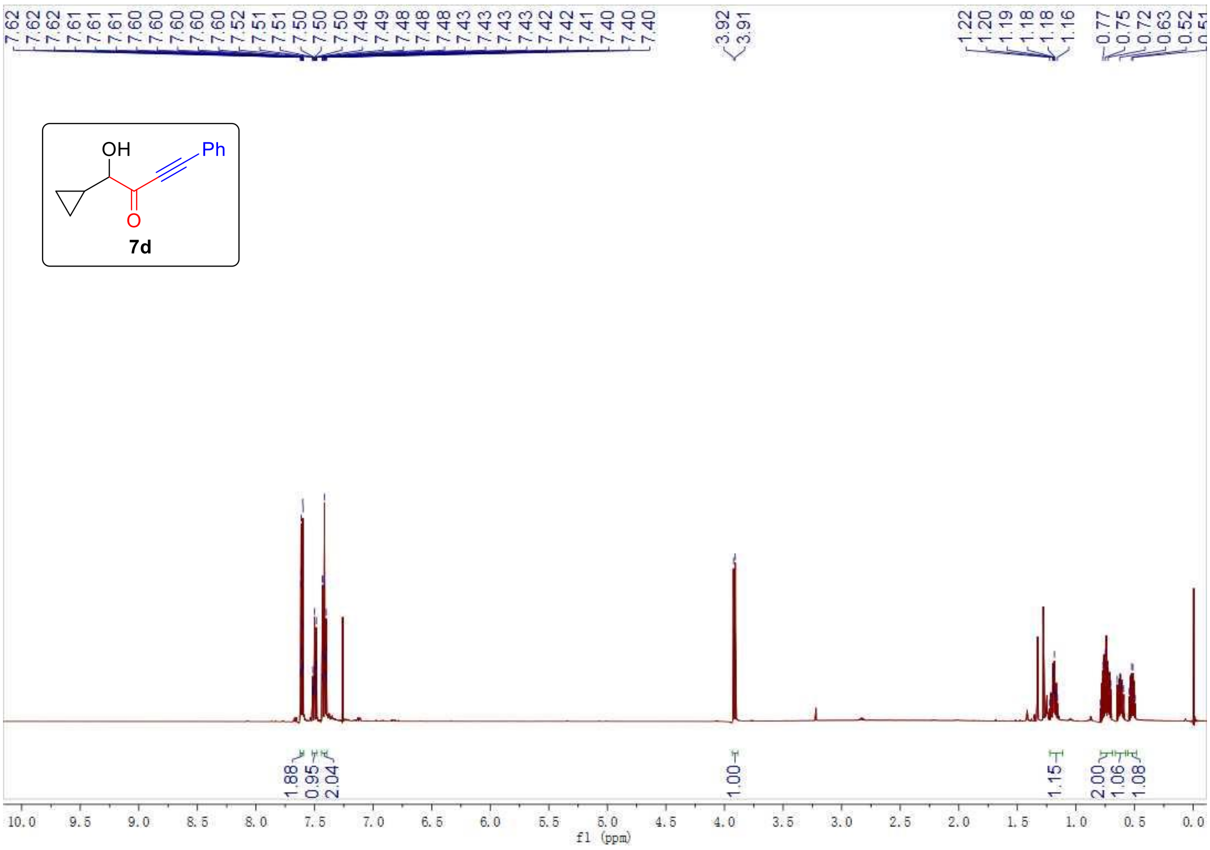

^{13}C NMR (126 MHz, $CDCl_3$) spectrum of 7d

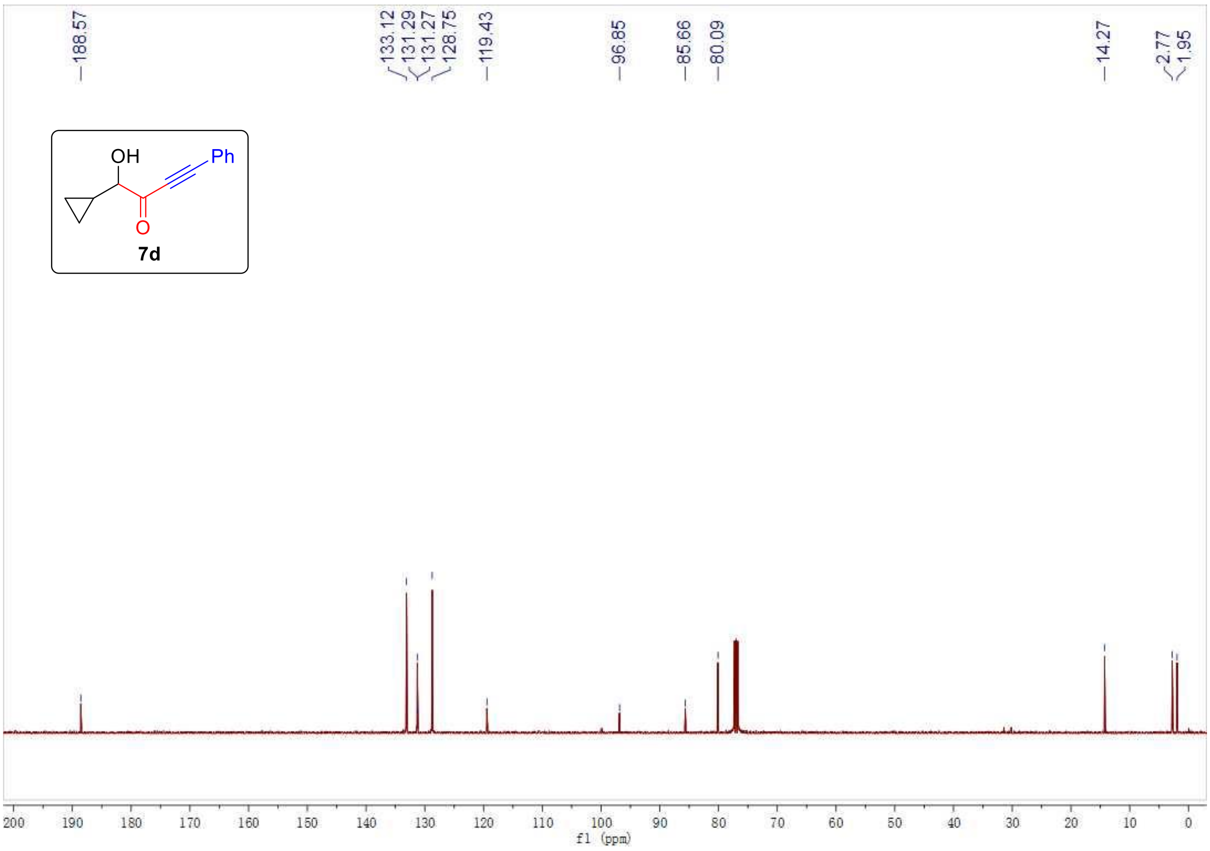

^{1}H NMR (400 MHz, $CDCl_3$) spectrum of 8a

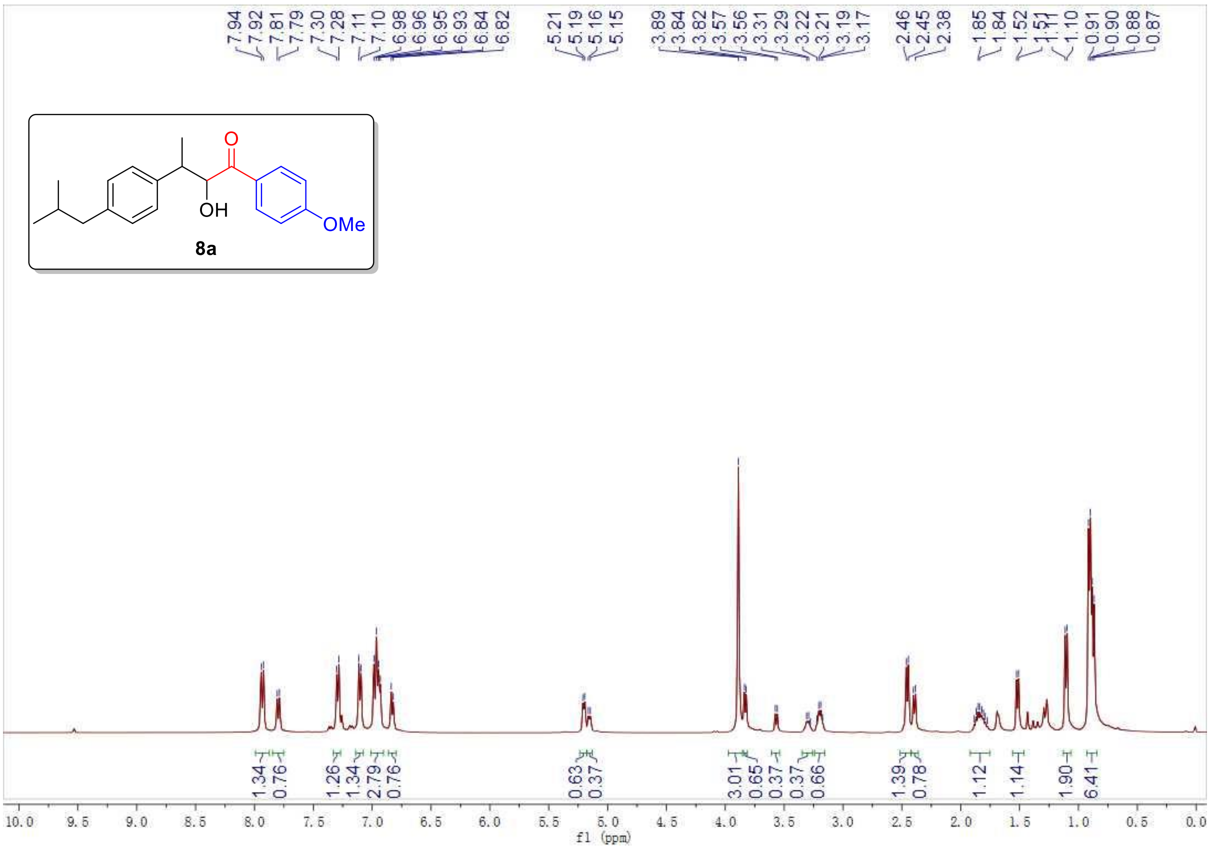

^{13}C NMR (101 MHz, $CDCl_3$) spectrum of 8a

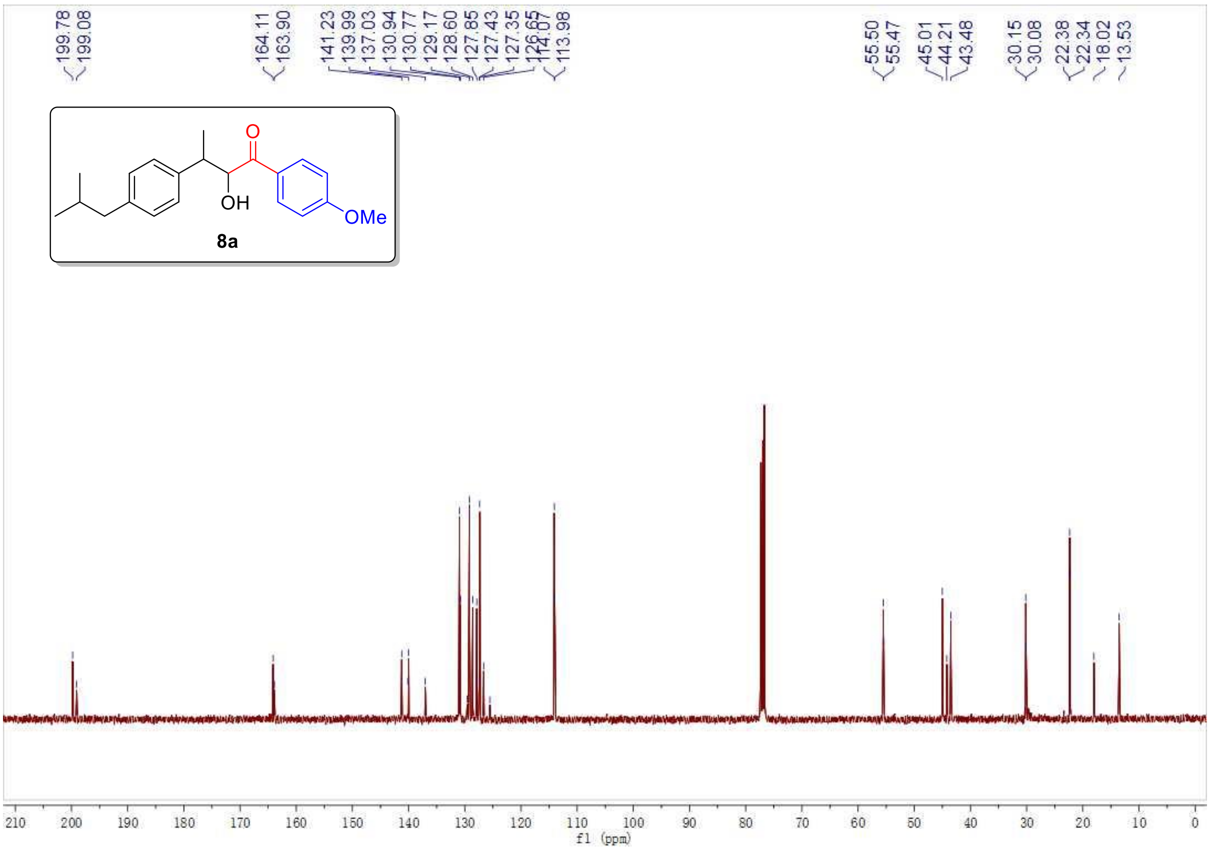

^{1}H NMR (400 MHz, $CDCl_3$) spectrum of 8b

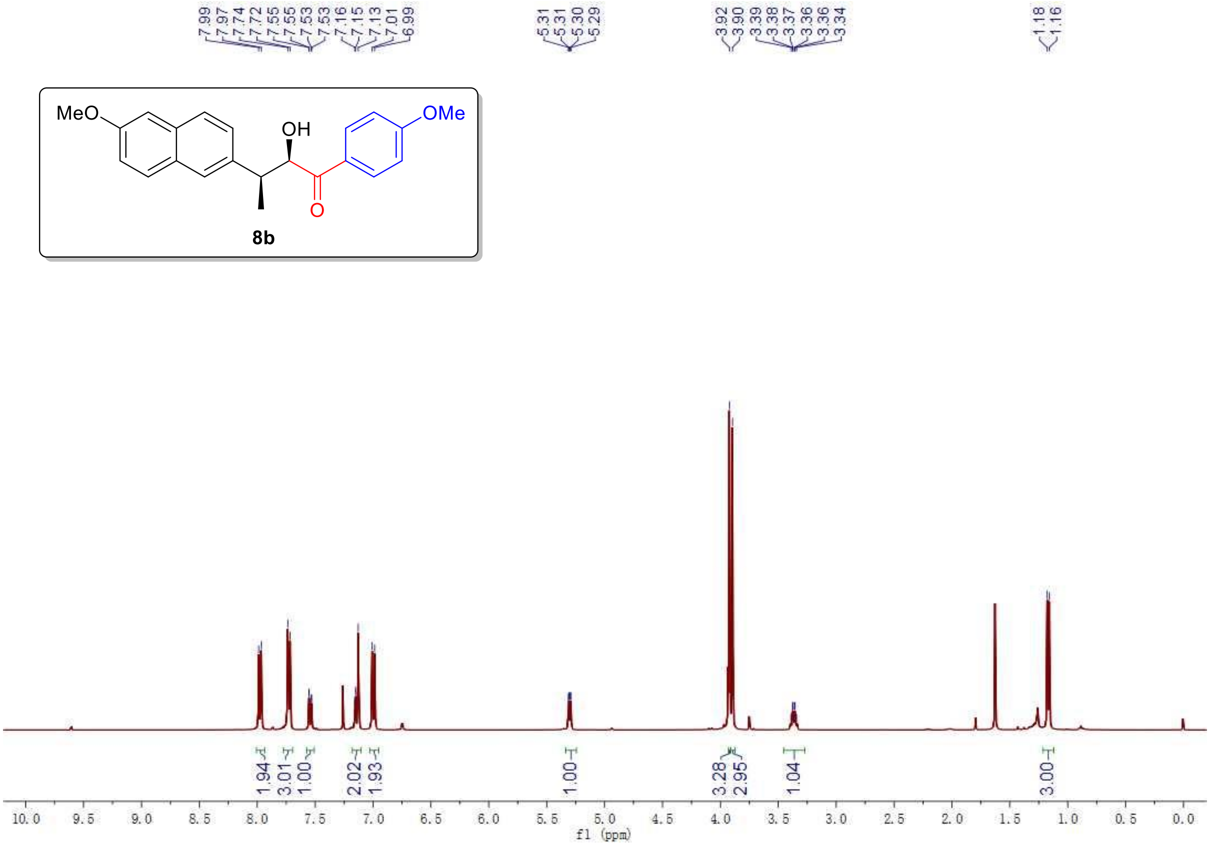

^{13}C NMR (101 MHz, $CDCl_3$) spectrum of 8b

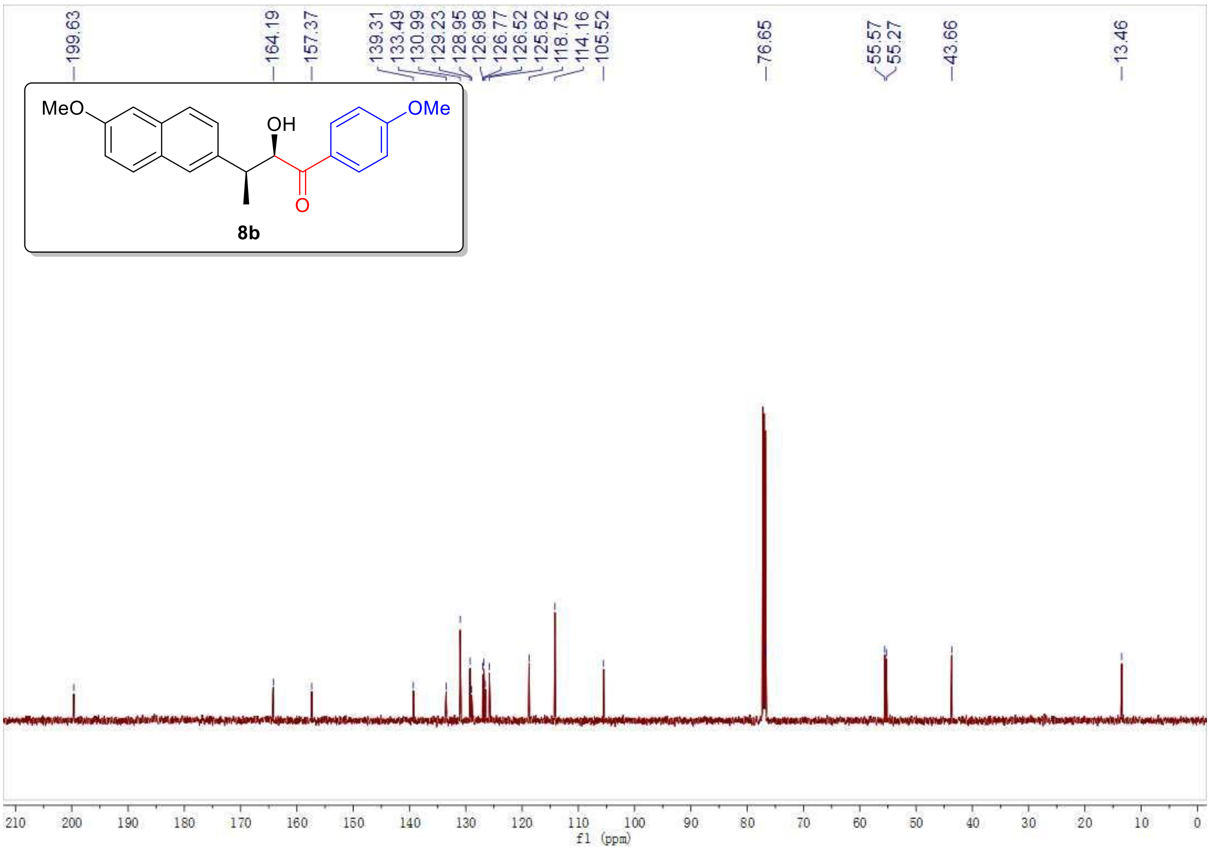

^{1}H NMR (400 MHz, $CDCl_3$) spectrum of 8c

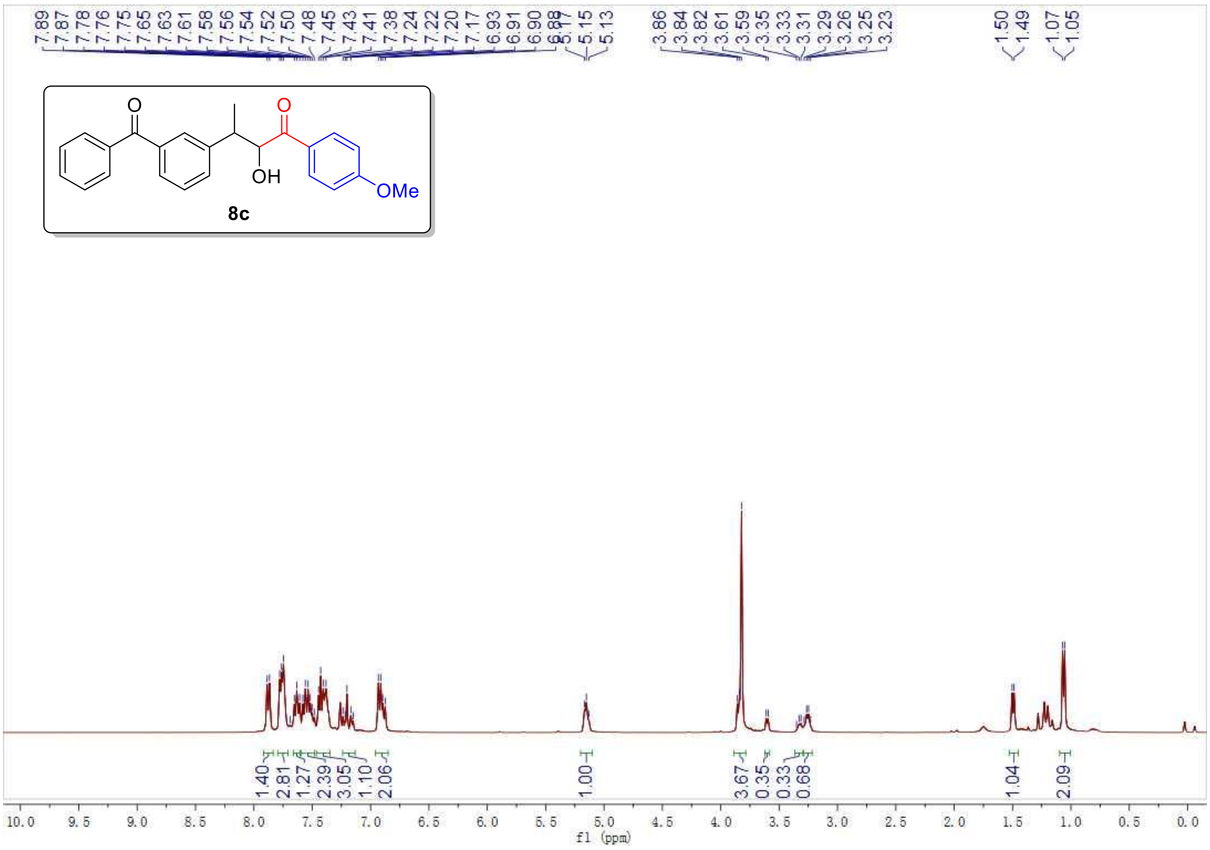

^{13}C NMR (101 MHz, $CDCl_3$) spectrum of 8c

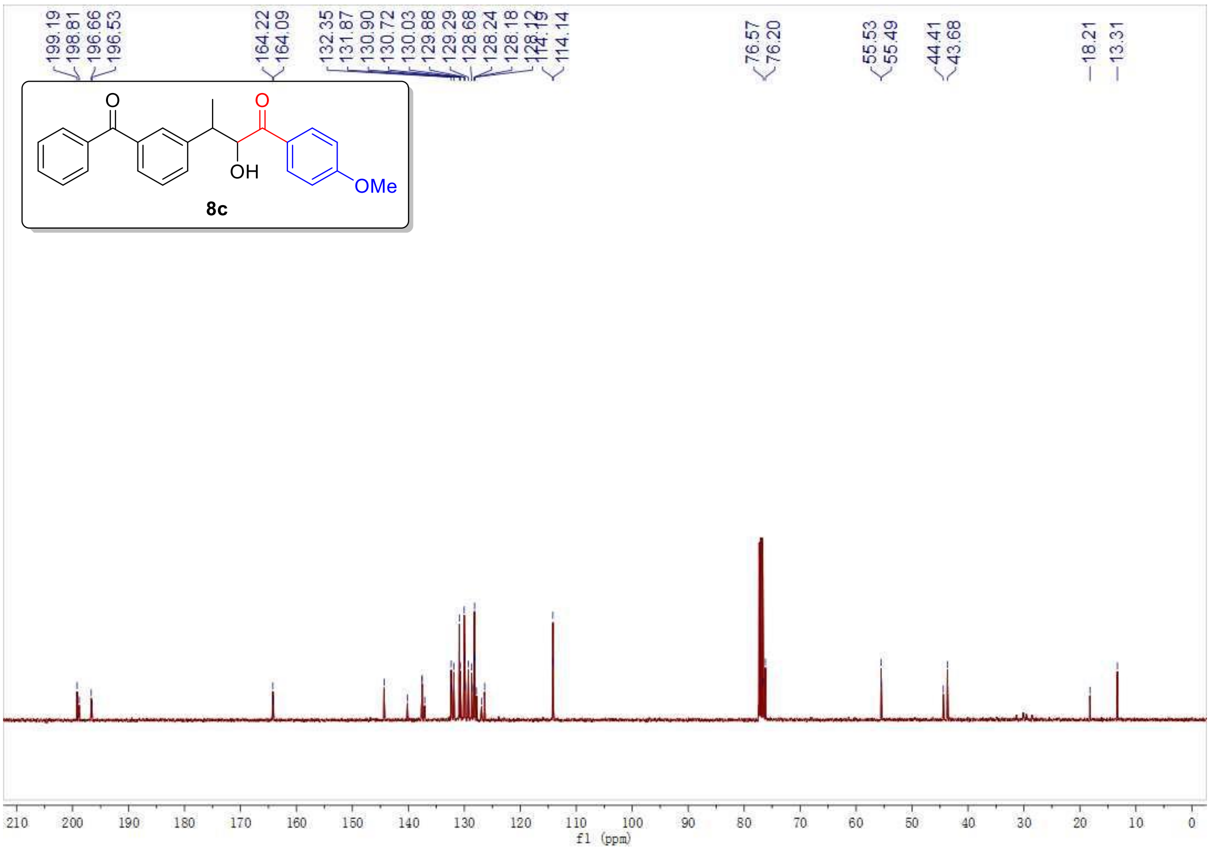

^{1}H NMR (400 MHz, $CDCl_3$) spectrum of 8d

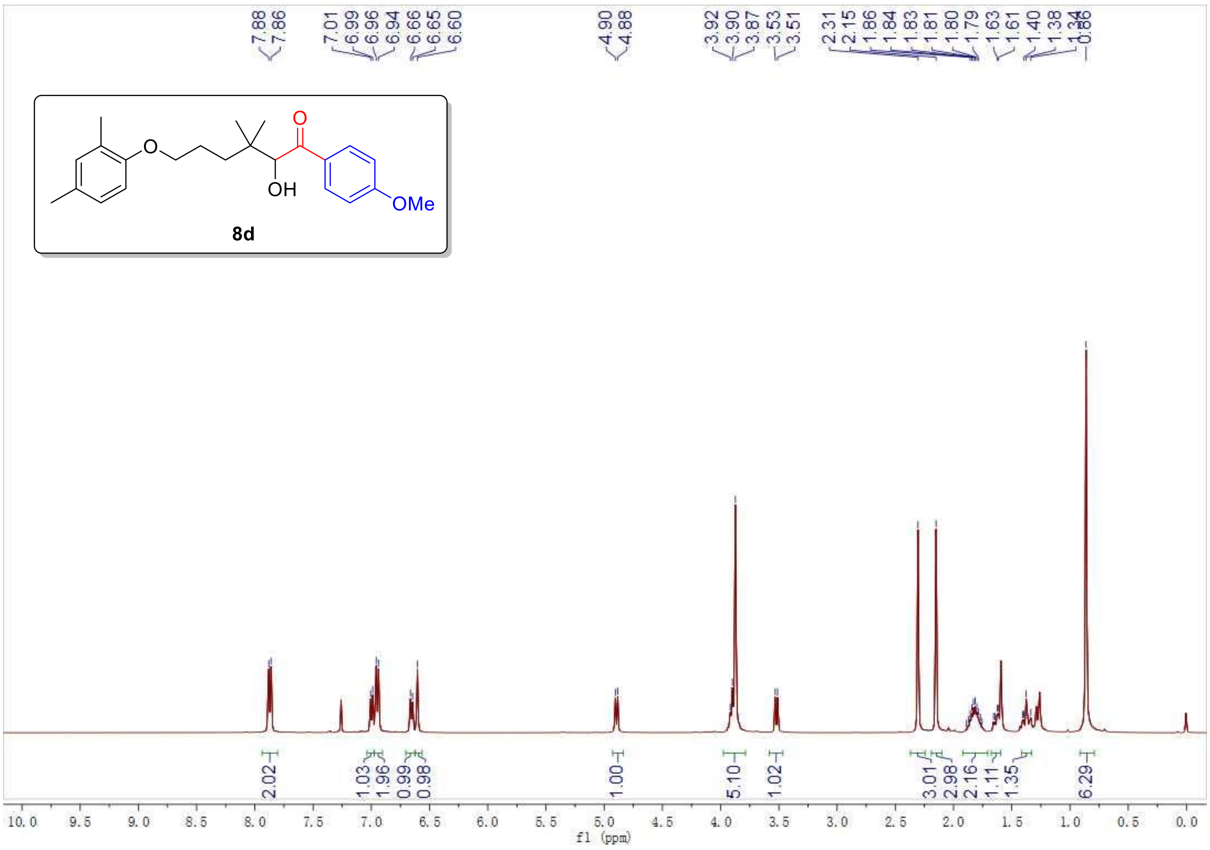

^{13}C NMR (101 MHz, $CDCl_3$) spectrum of 8d

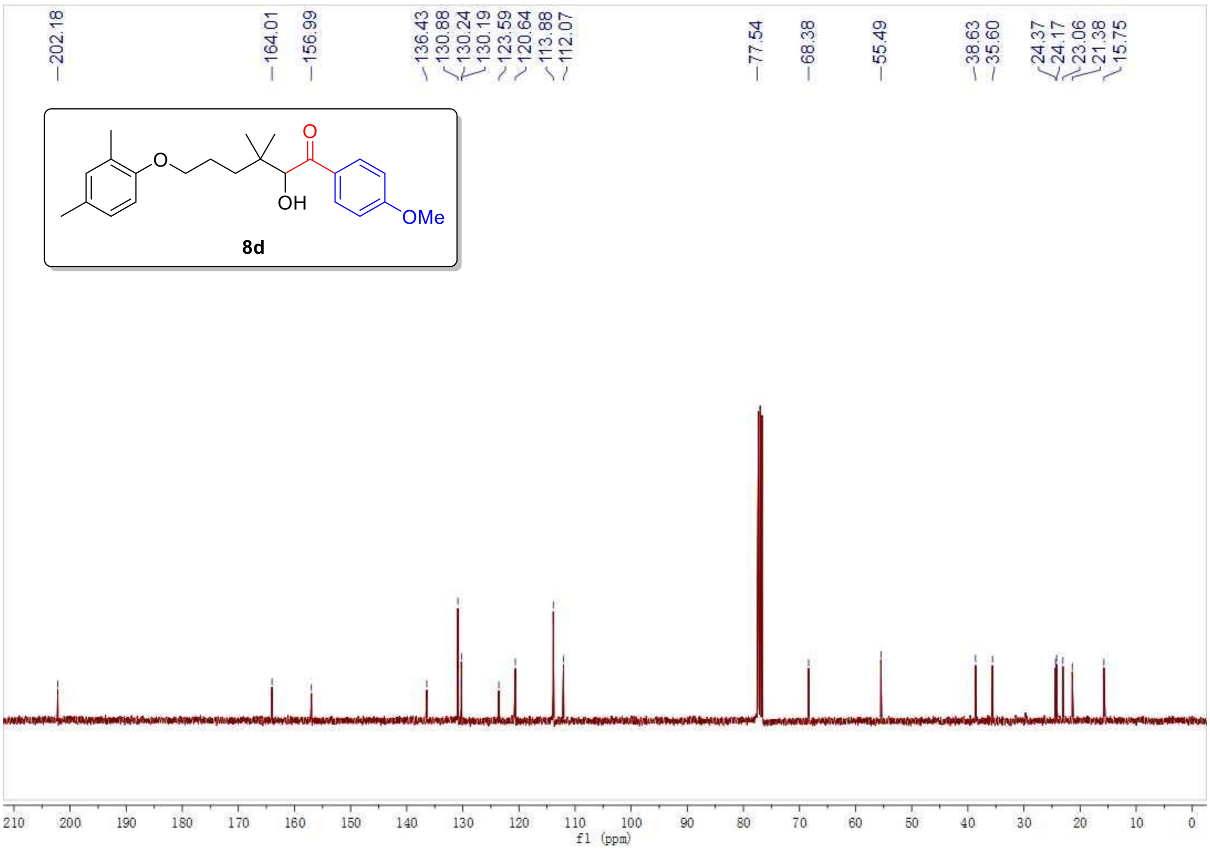

^{1}H NMR (400 MHz, $CDCl_3$) spectrum of 8e

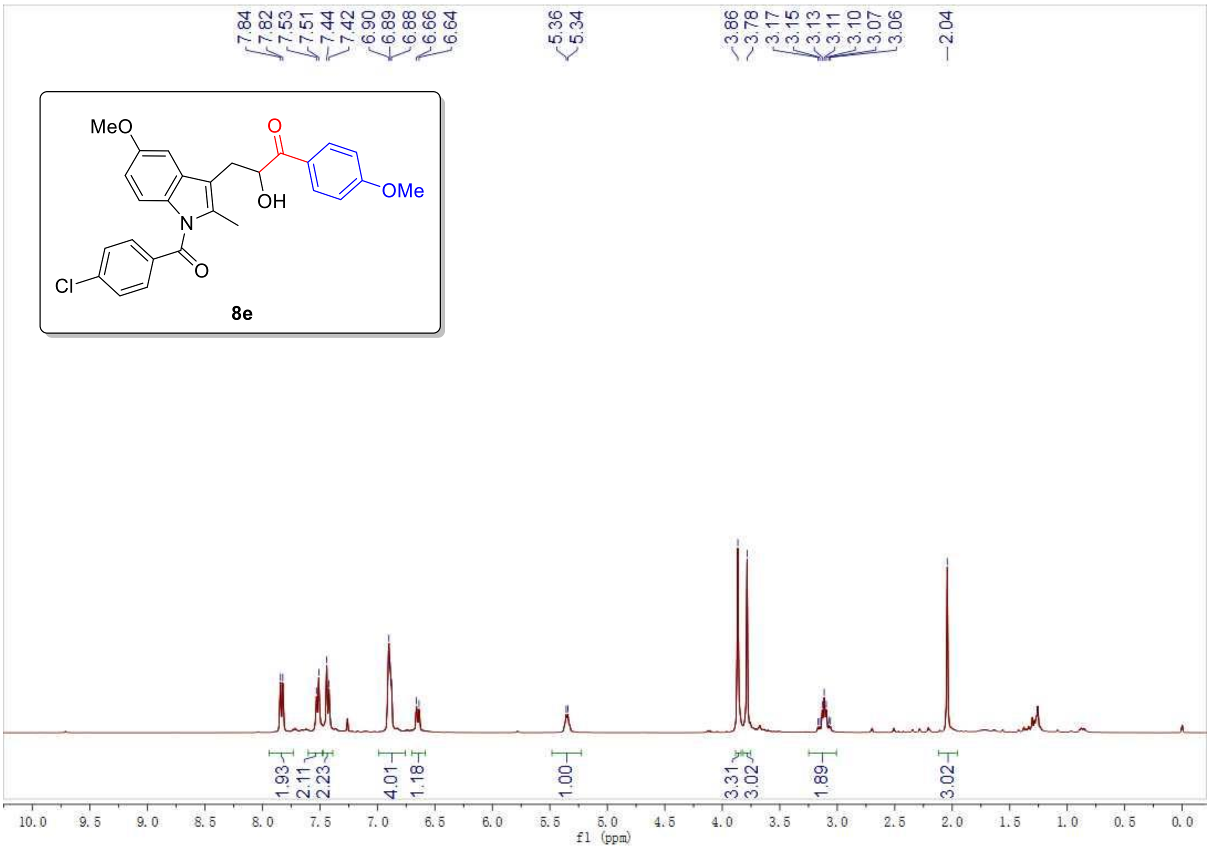

^{13}C NMR (101 MHz, $CDCl_3$) spectrum of 8e

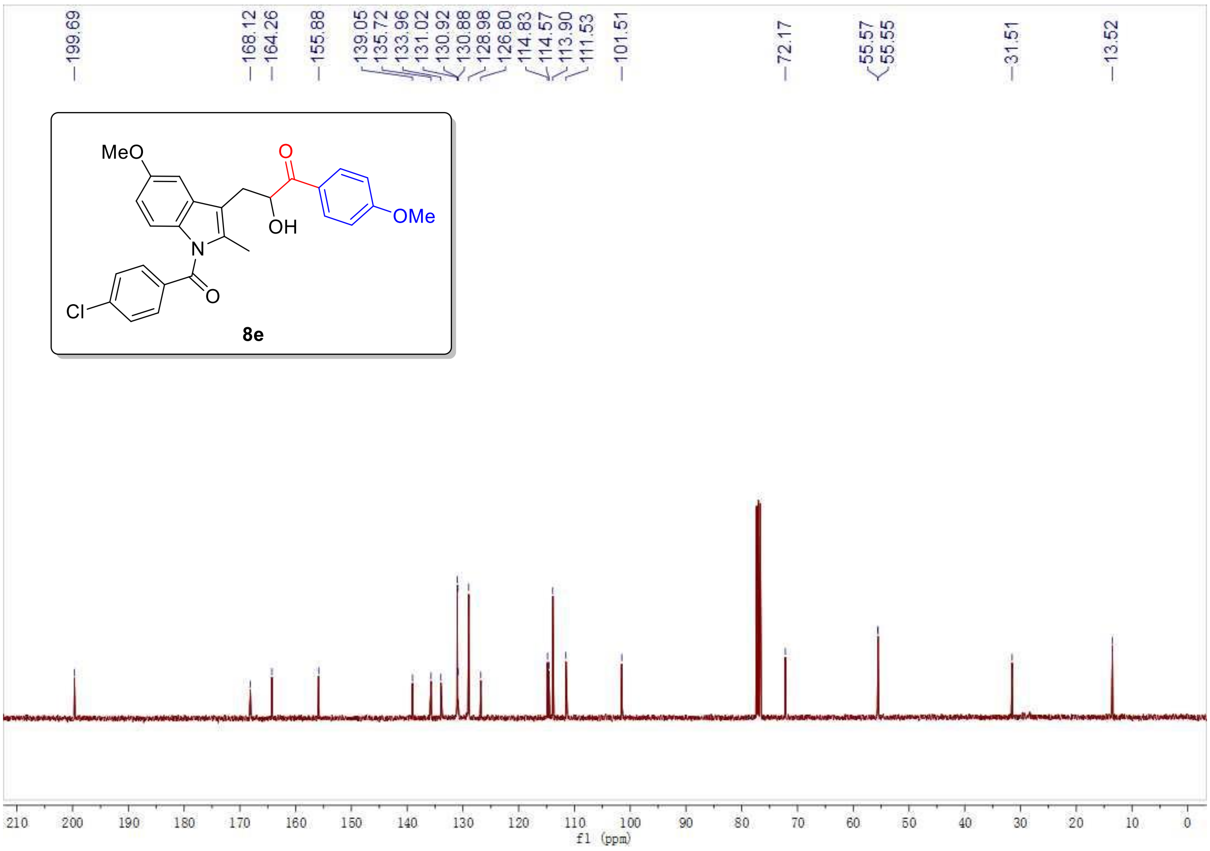

^{1}H NMR (400 MHz, $CDCl_3$) spectrum of 8f

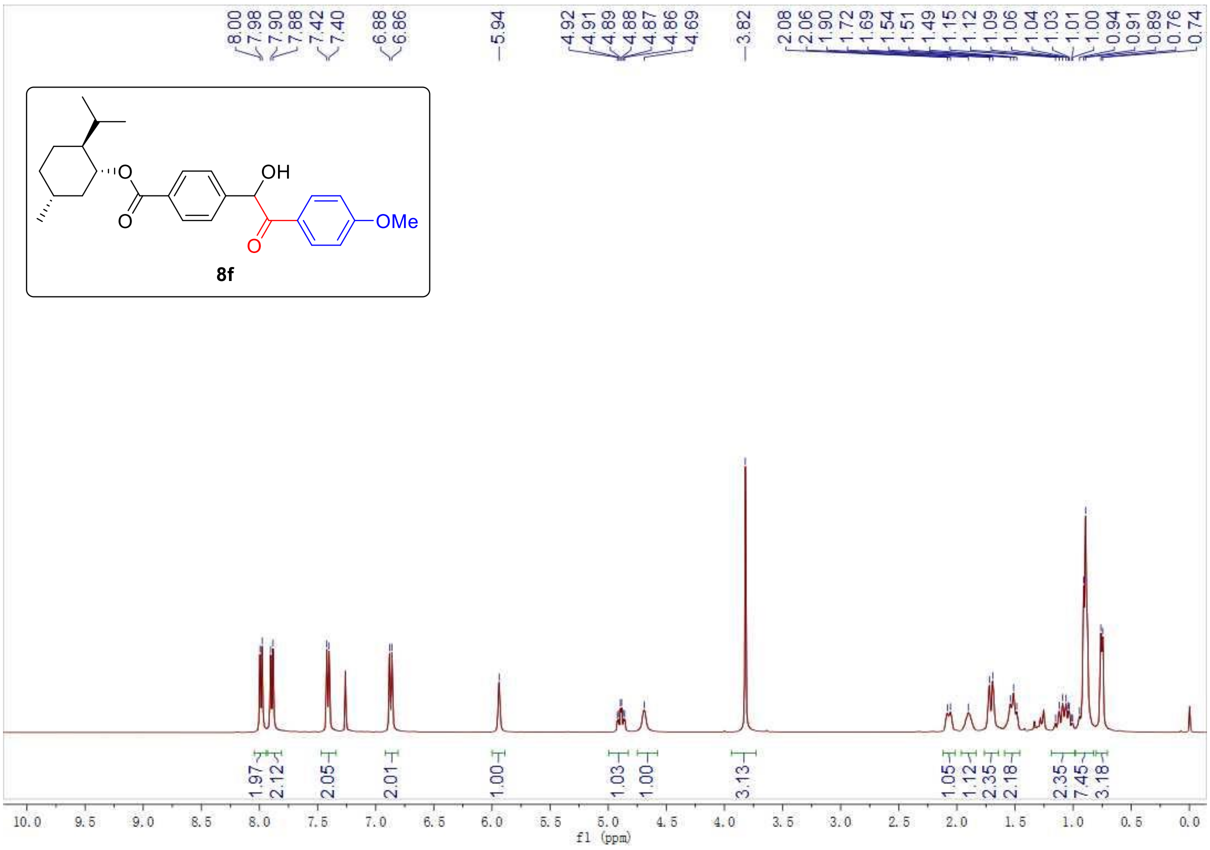

^{13}C NMR (101 MHz, $CDCl_3$) spectrum of 8f

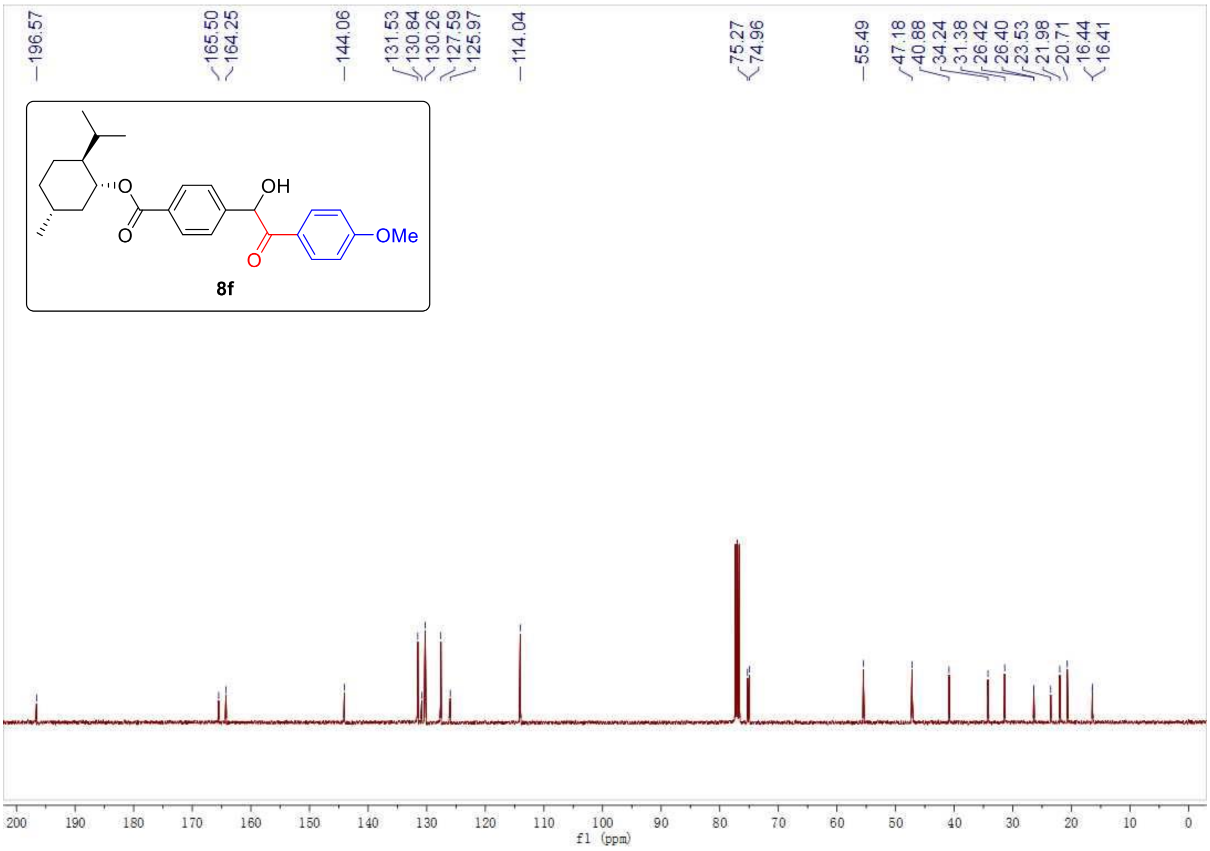

^{1}H NMR (400 MHz, $CDCl_3$) spectrum of 8g

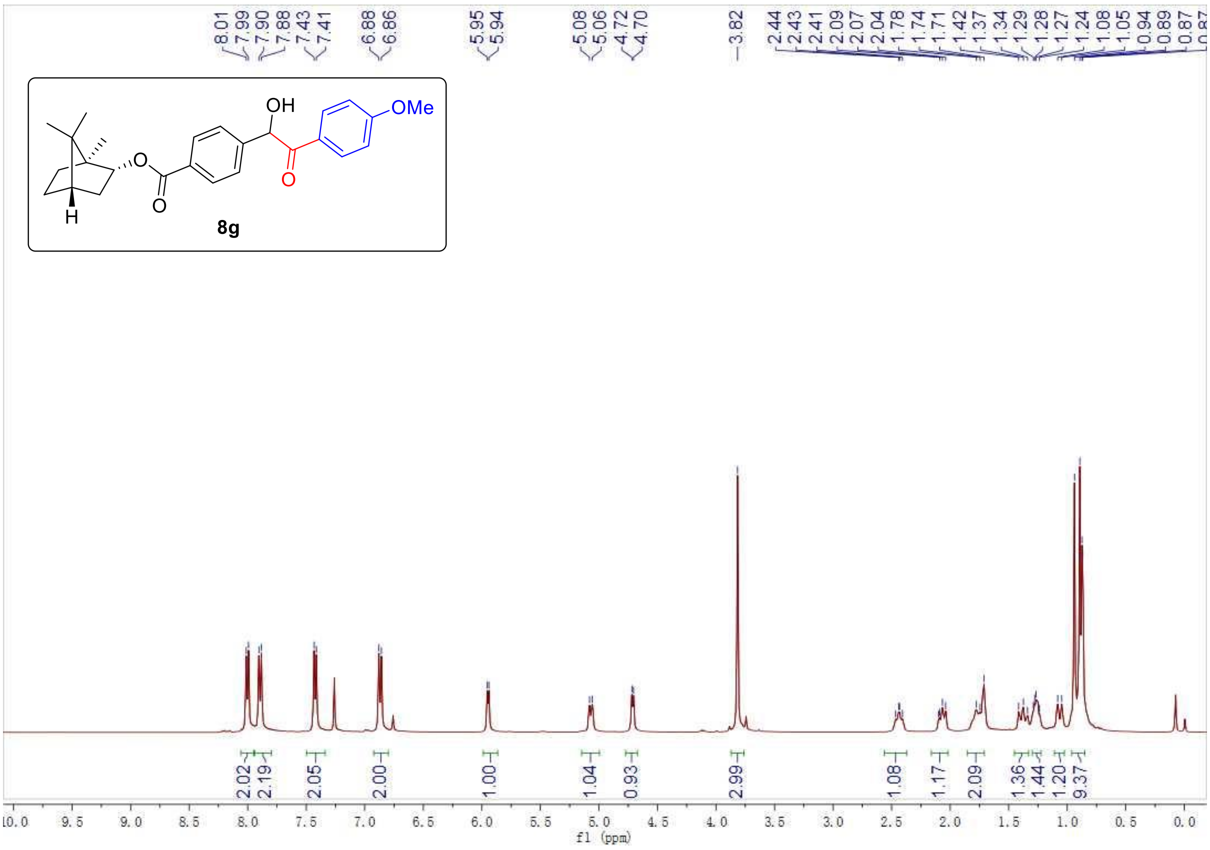

^{13}C NMR (101 MHz, $CDCl_3$) spectrum of 8g

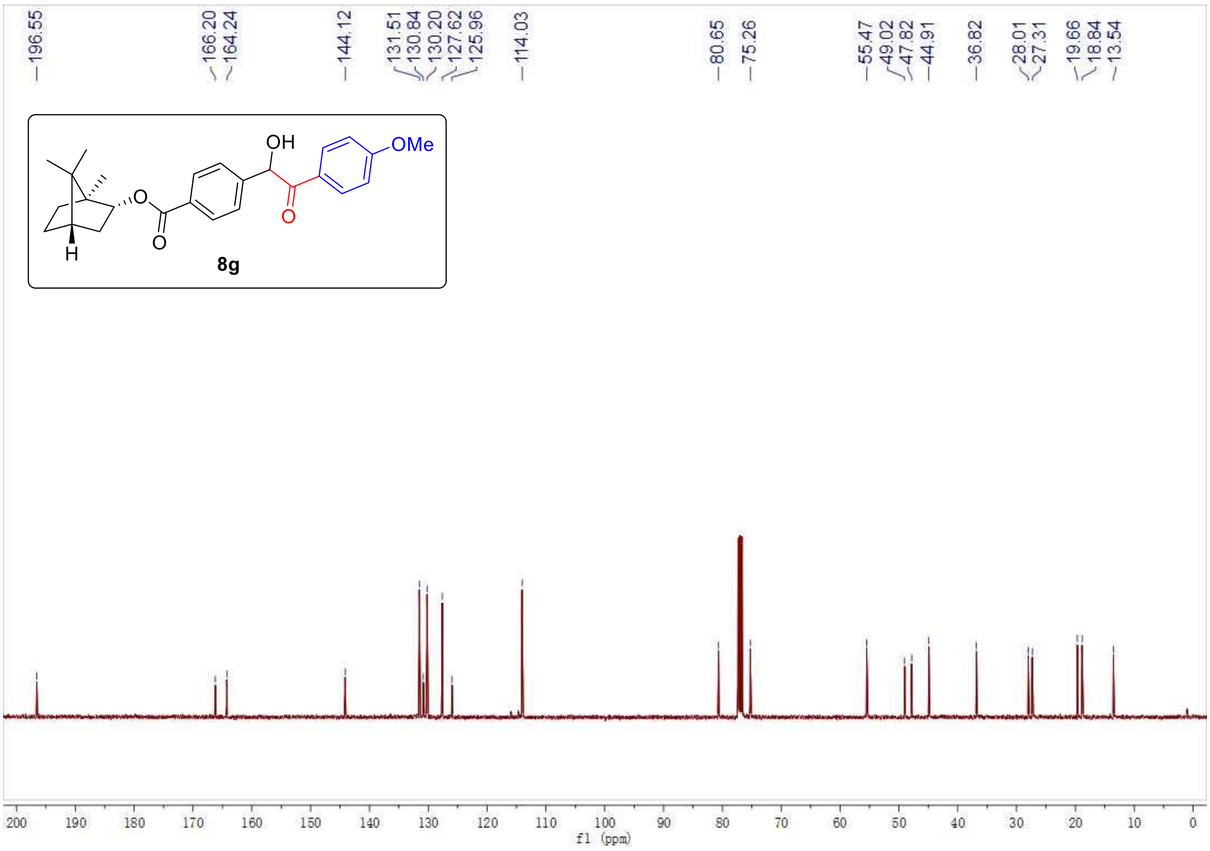

^{1}H NMR (400 MHz, $CDCl_3$) spectrum of 8h

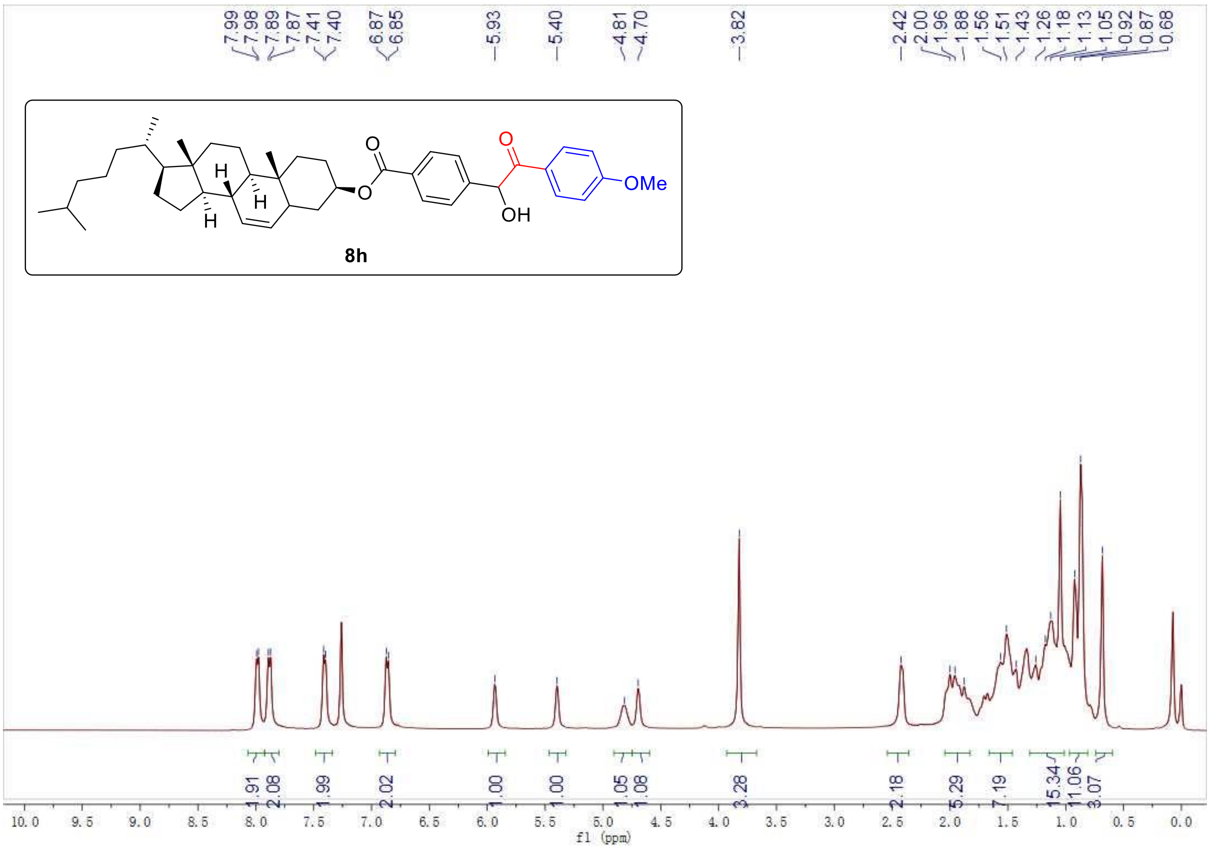

^{13}C NMR (101 MHz, $CDCl_3$) spectrum of 8h

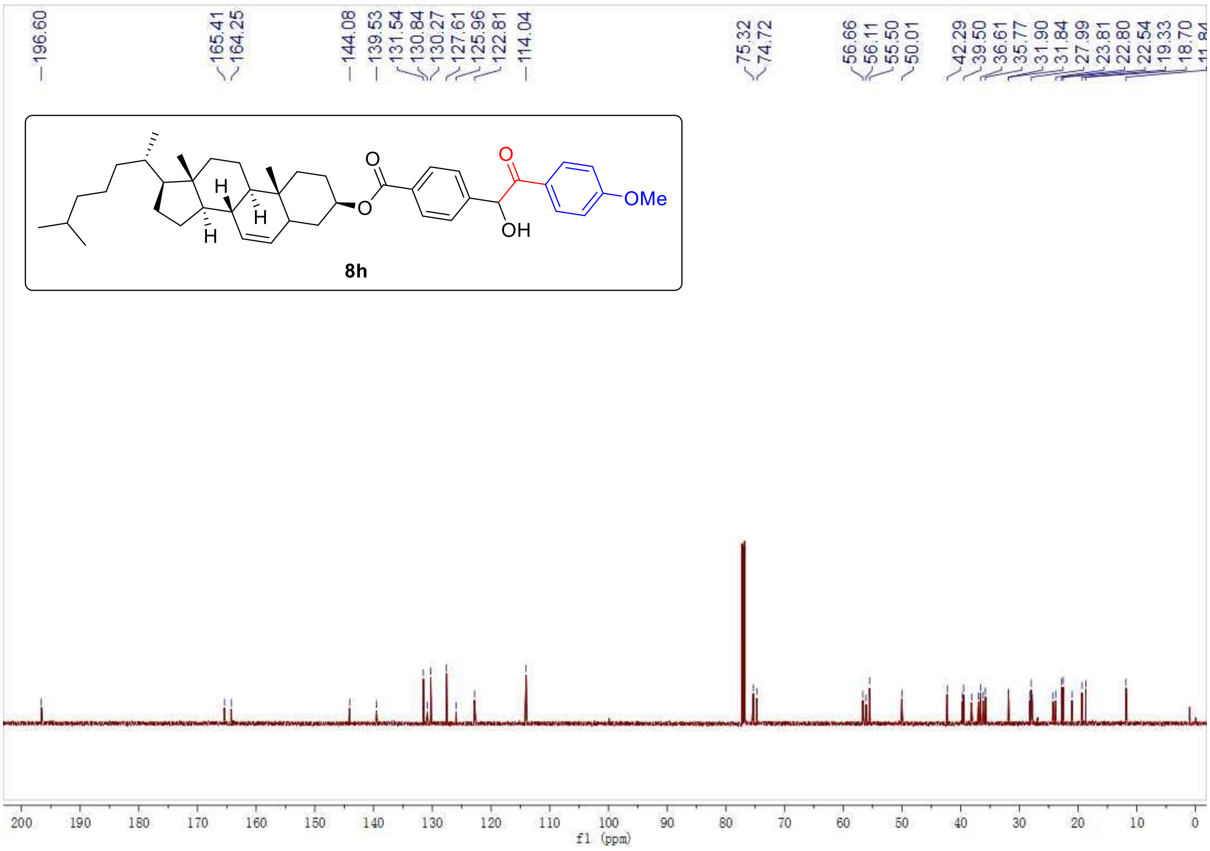

^{1}H NMR (400 MHz, $CDCl_3$) spectrum of 8i

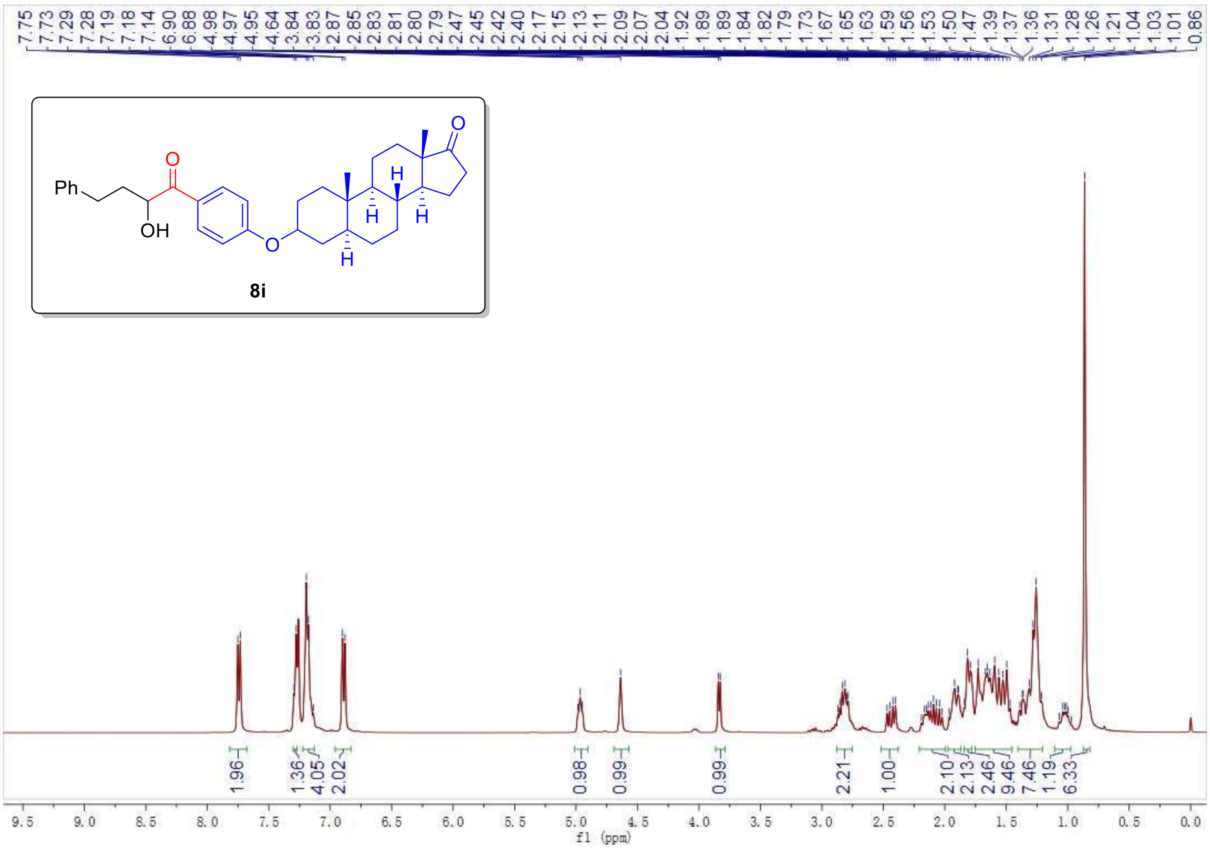

^{13}C NMR (101 MHz, $CDCl_3$) spectrum of 8i

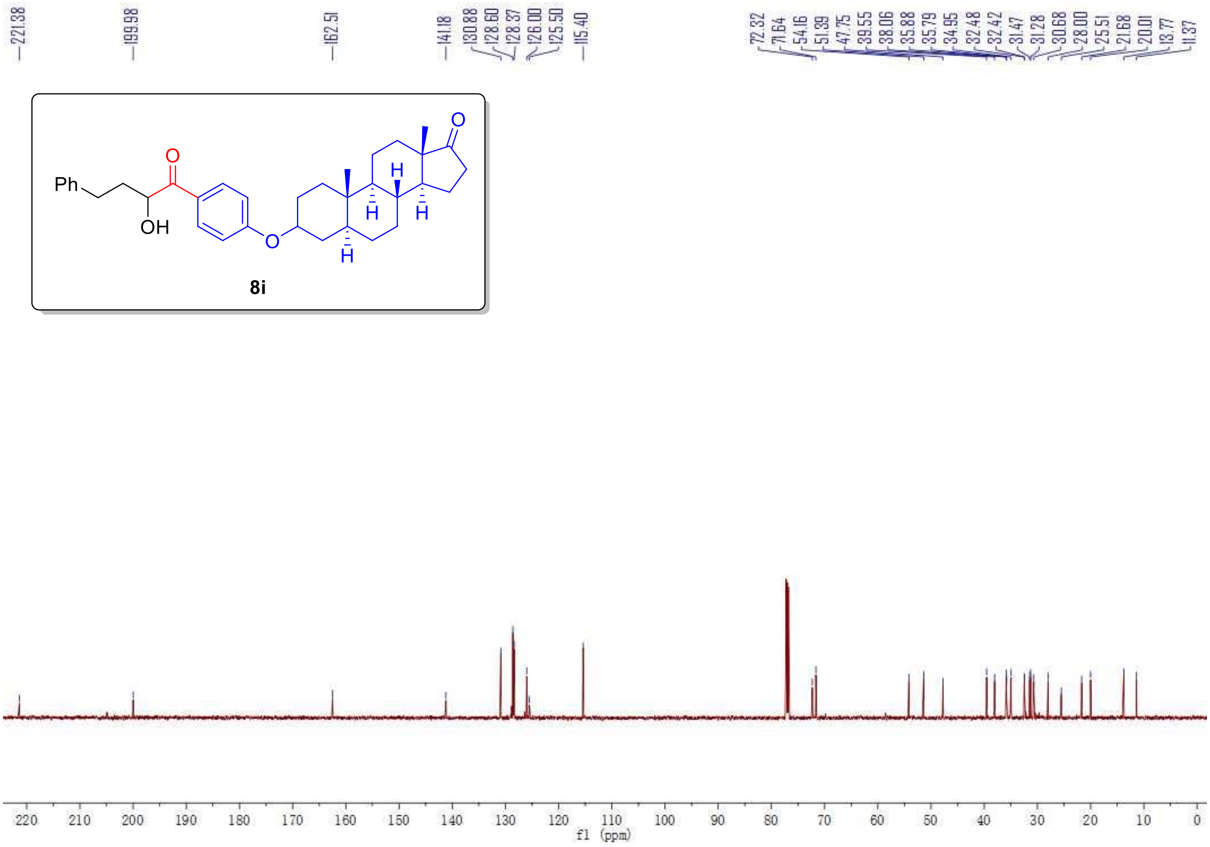

^{1}H NMR (400 MHz, $CDCl_3$) spectrum of 8j

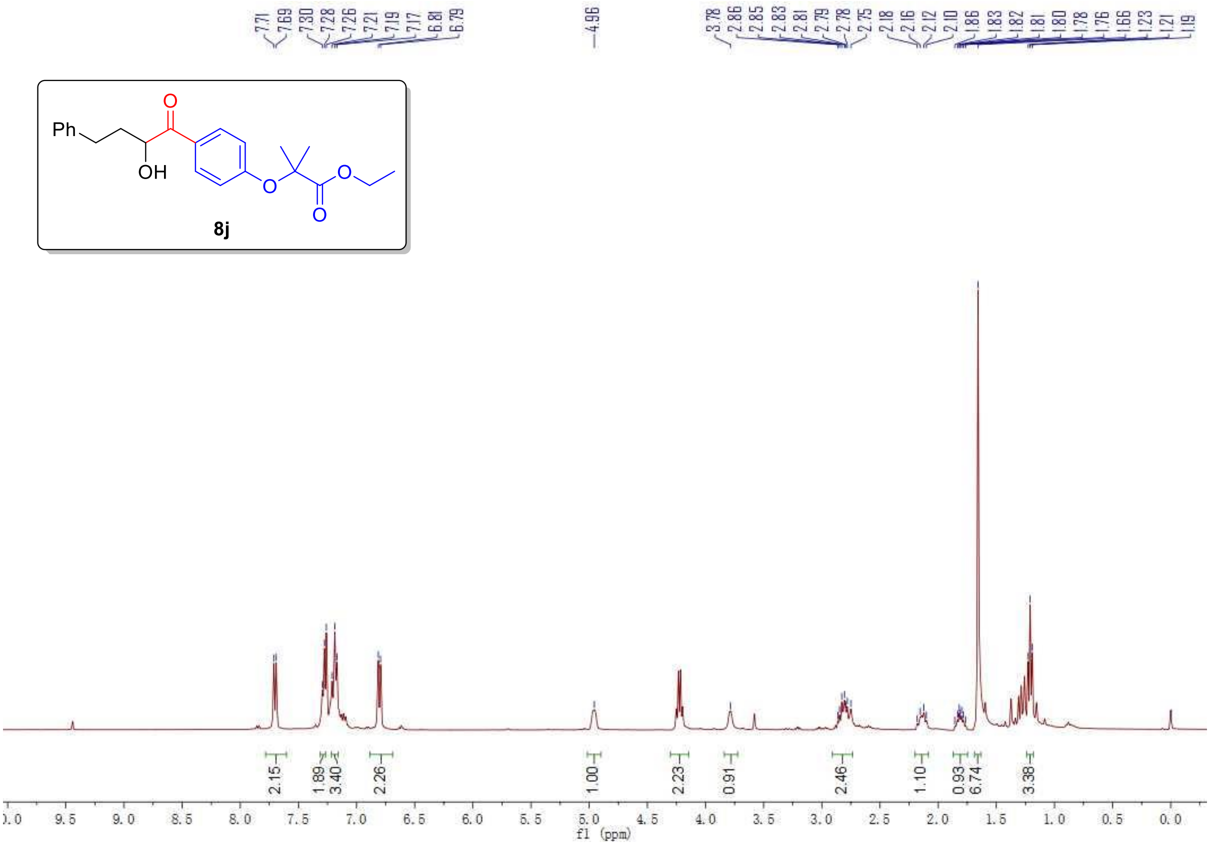

^{13}C NMR (101 MHz, $CDCl_3$) spectrum of 8j

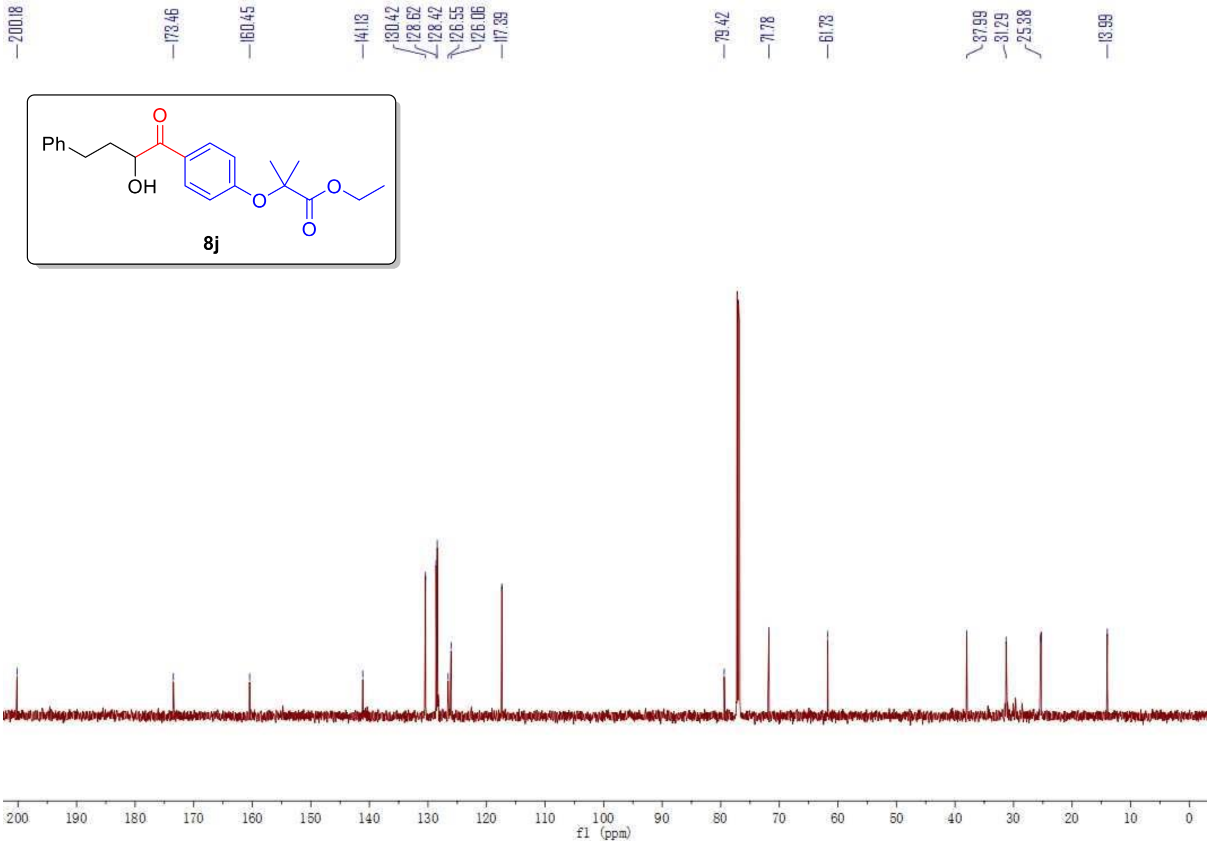

^{1}H NMR (400 MHz, $CDCl_3$) spectrum of 9

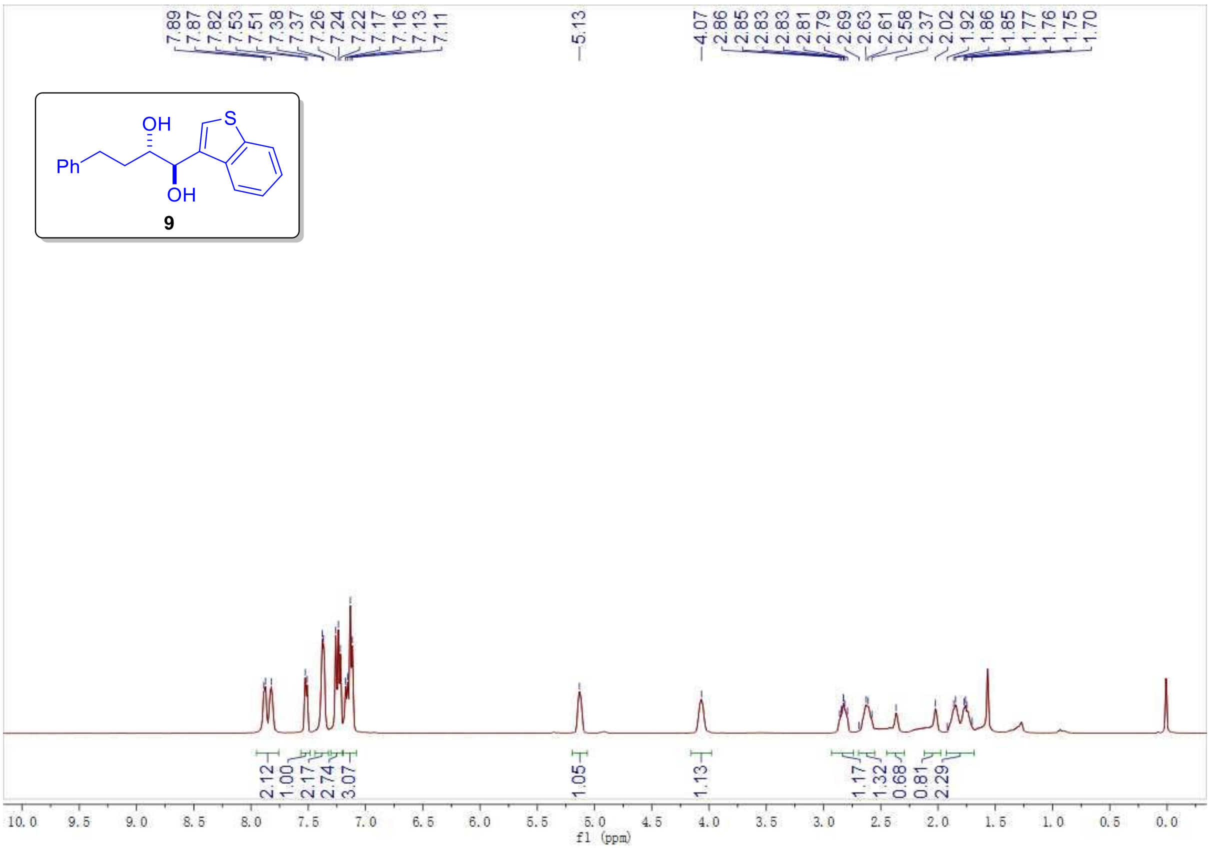

^{13}C NMR (101 MHz, $CDCl_3$) spectrum of 9

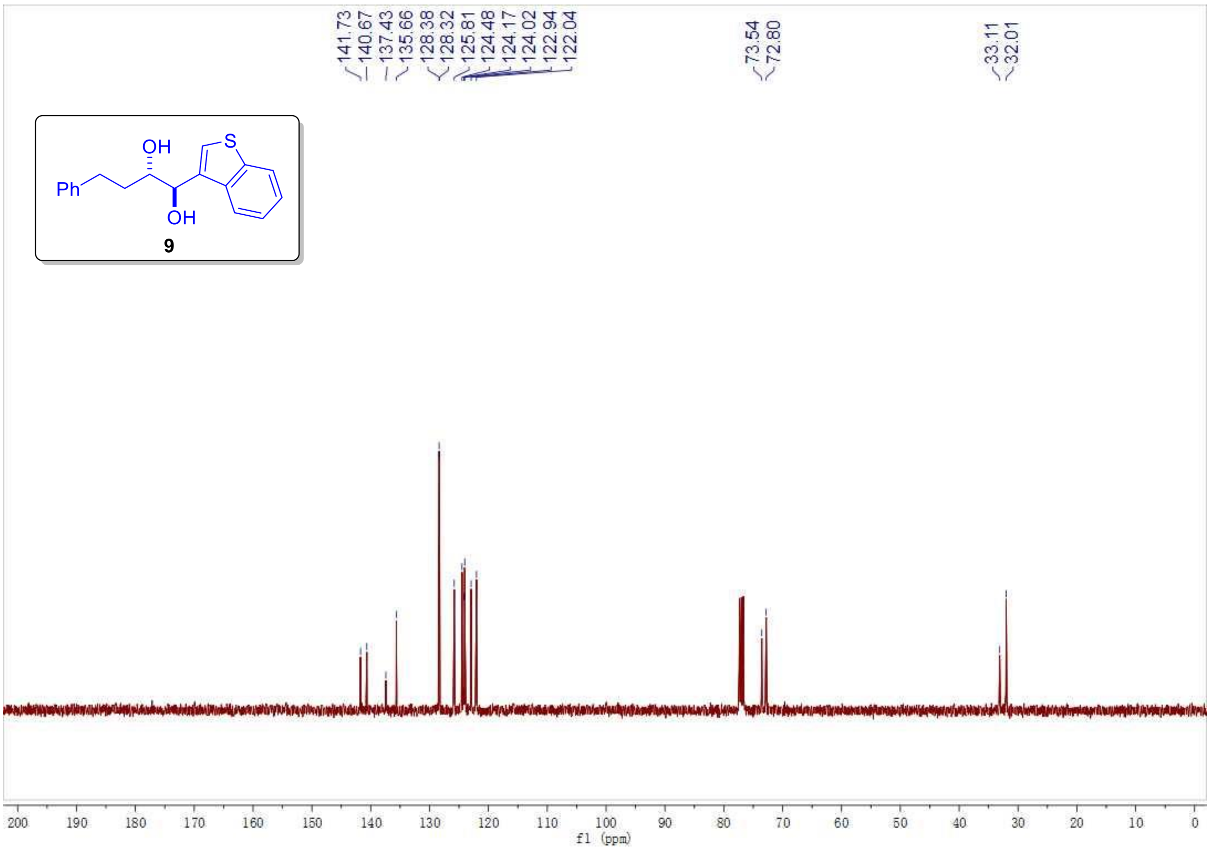

^{1}H NMR (400 MHz, $CDCl_3$) spectrum of 10

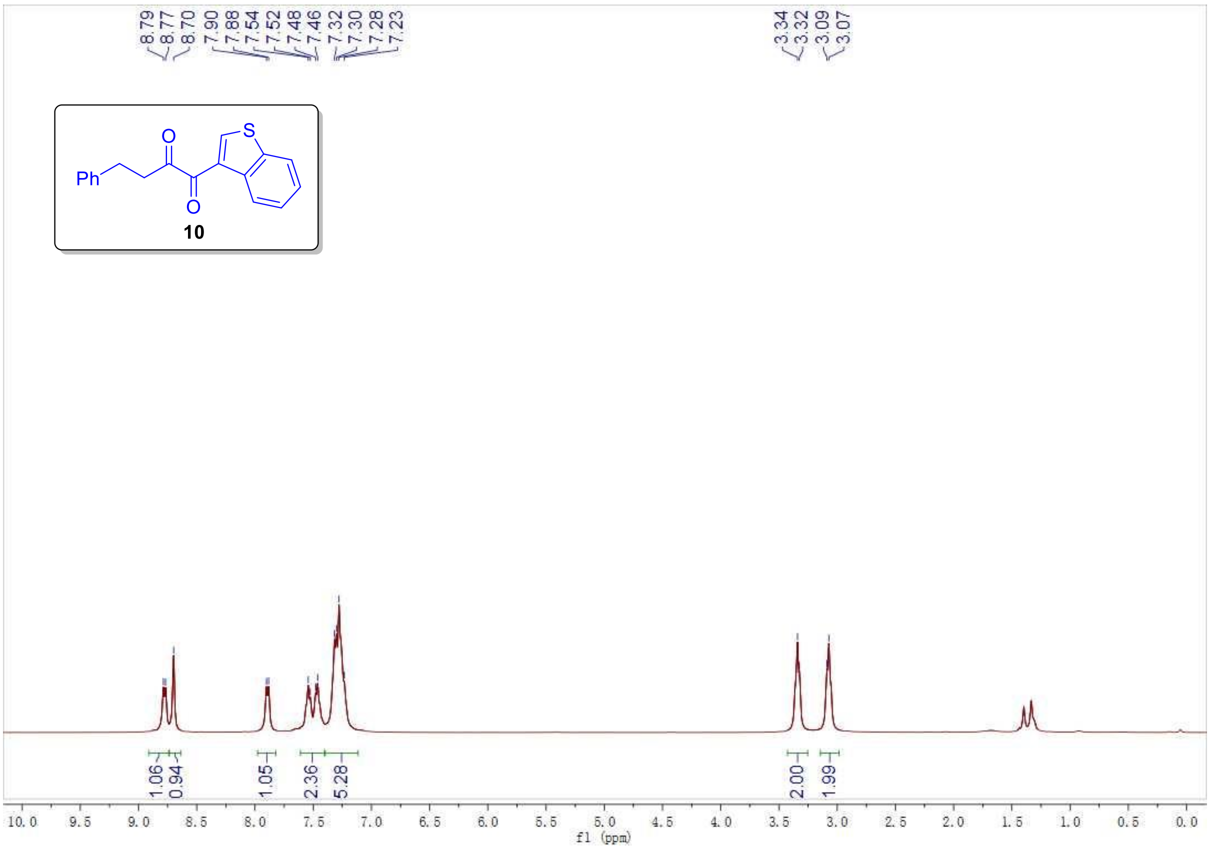

^{13}C NMR (101 MHz, $CDCl_3$) spectrum of 10

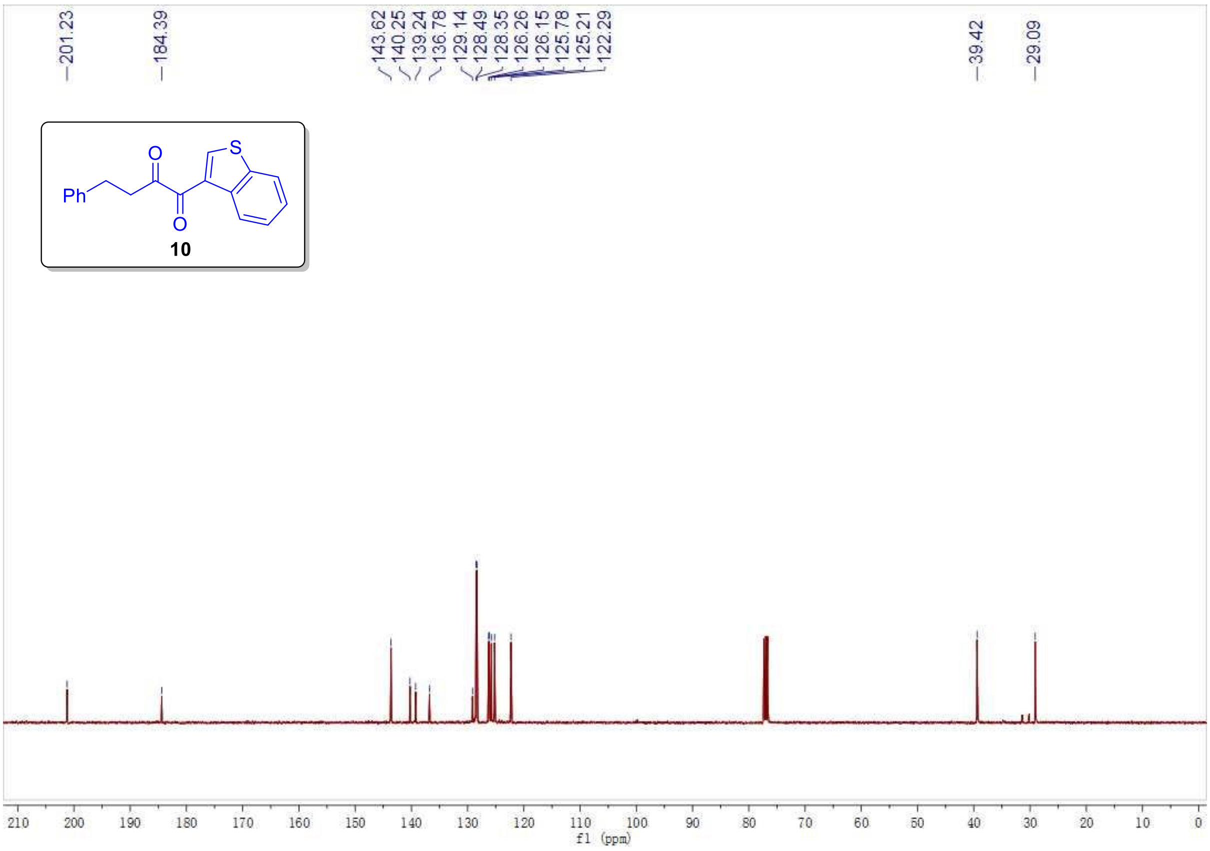

^{1}H NMR (400 MHz, $CDCl_3$) spectrum of 11

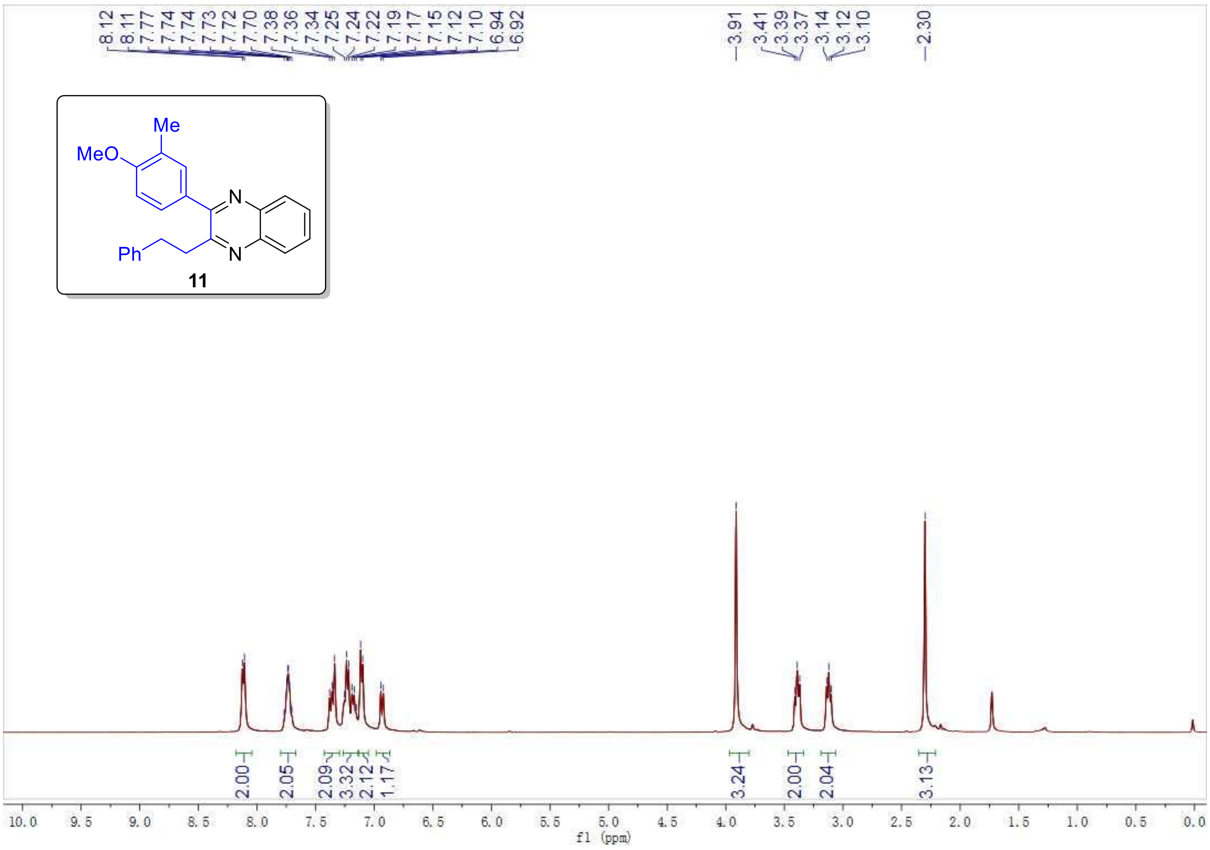

^{13}C NMR (101 MHz, $CDCl_3$) spectrum of 11

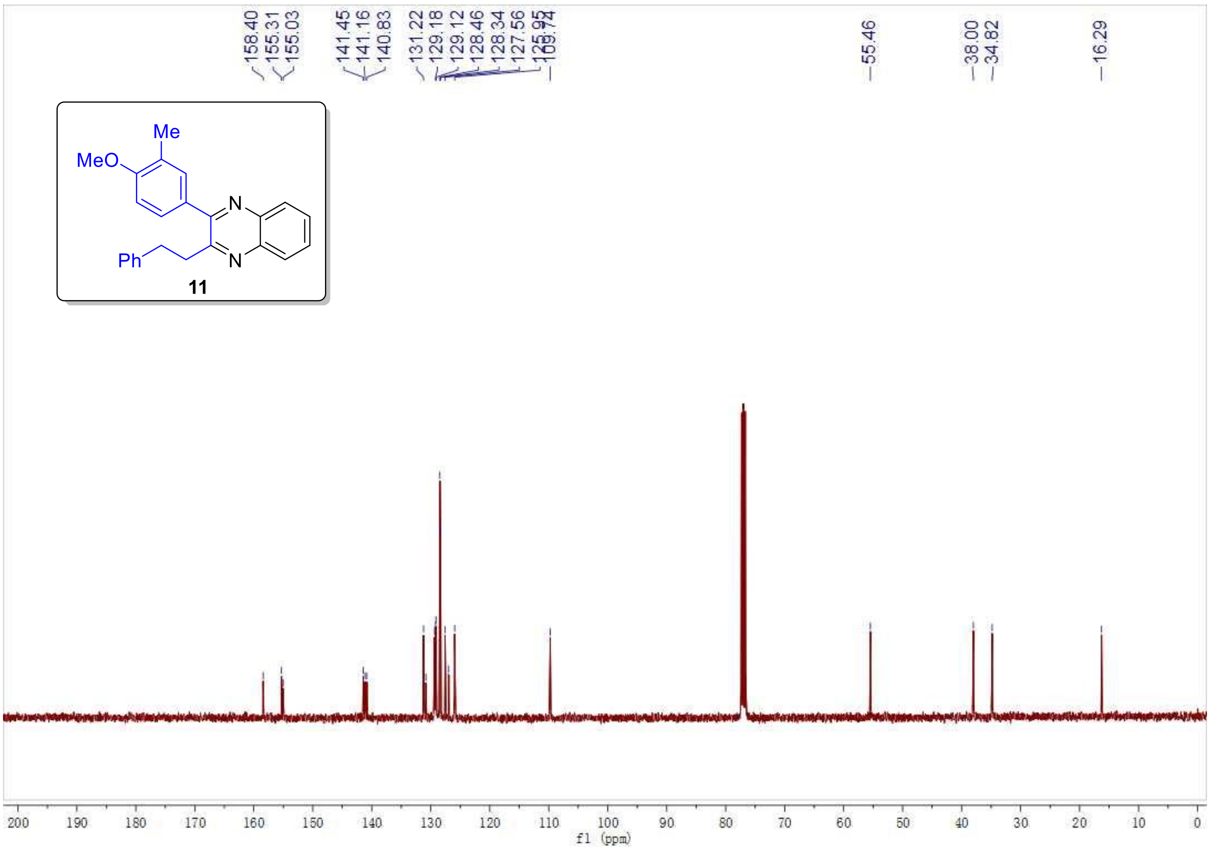

^{1}H NMR (400 MHz, $CDCl_3$) spectrum of 12

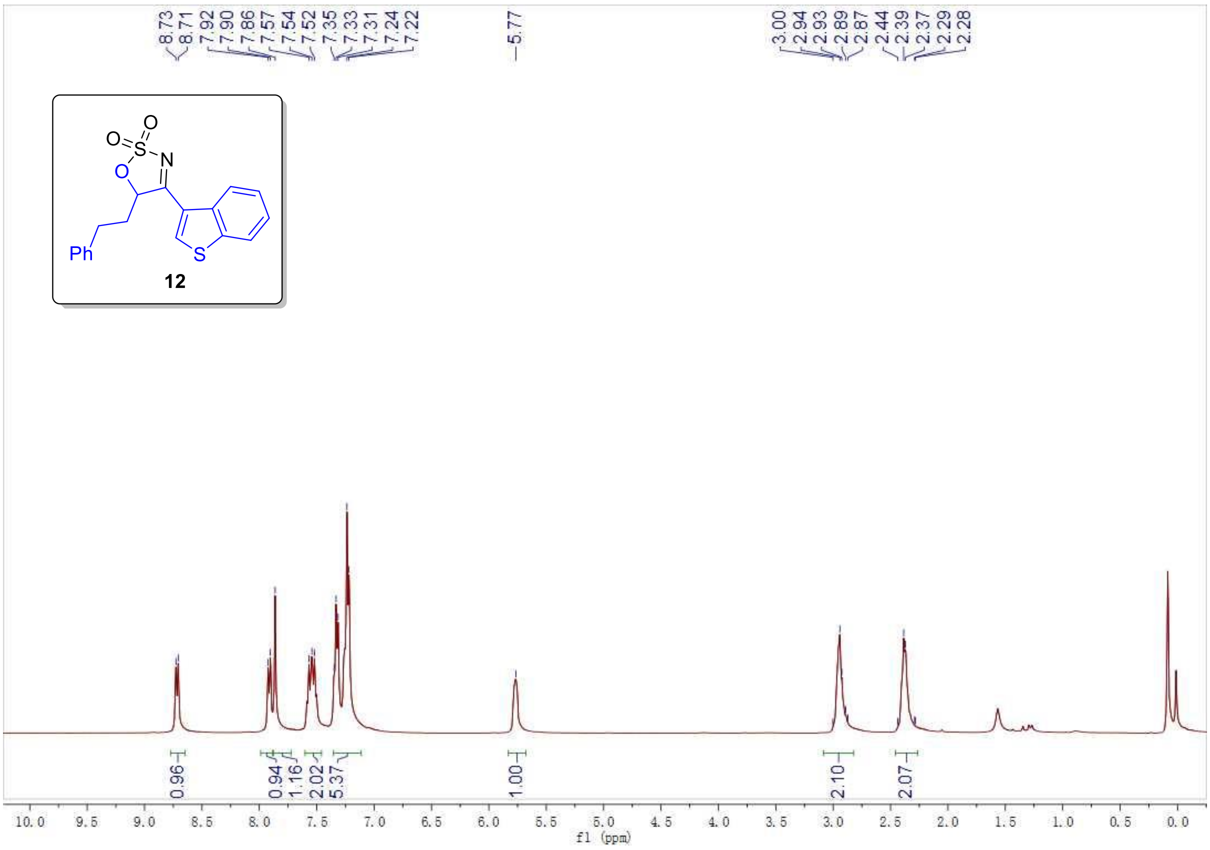

^{13}C NMR (101 MHz, $CDCl_3$) spectrum of 12

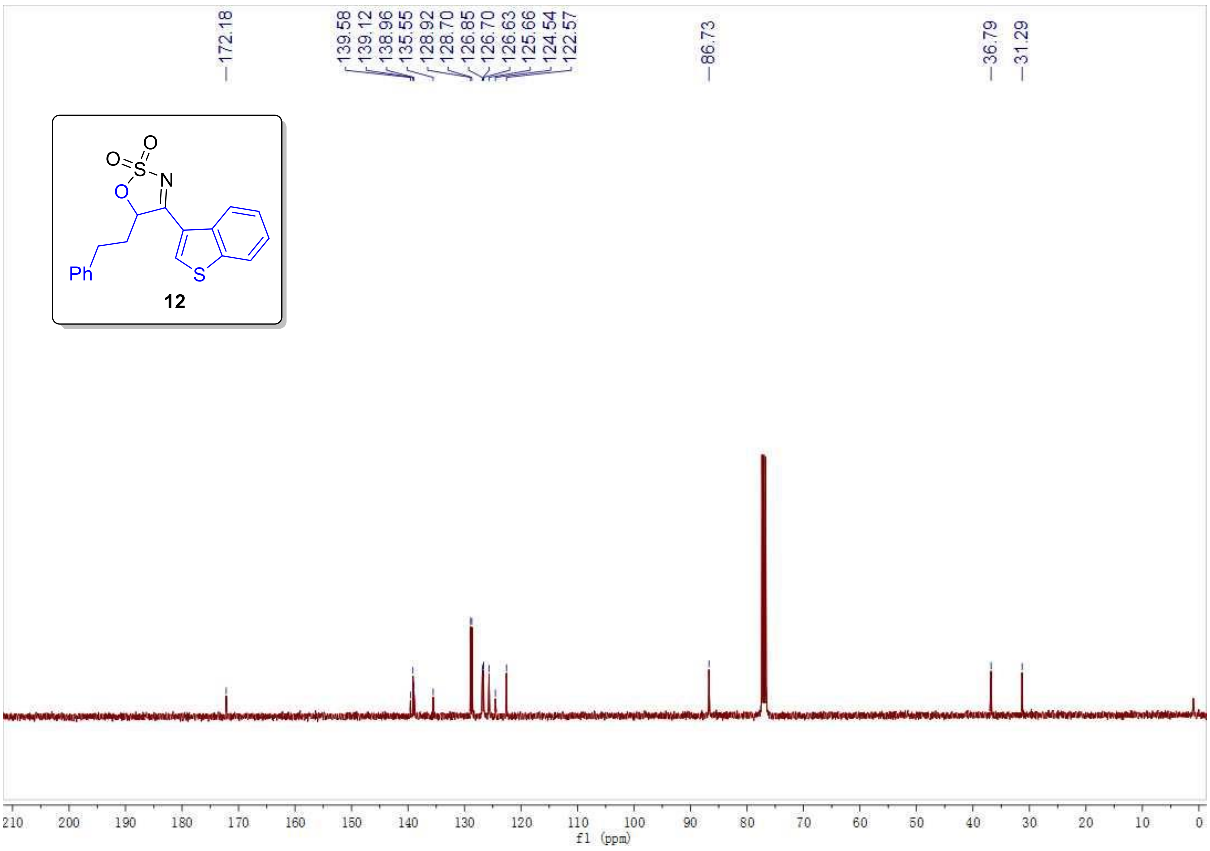

^1H NMR (400 MHz, $CDCl_3$) spectrum of 13

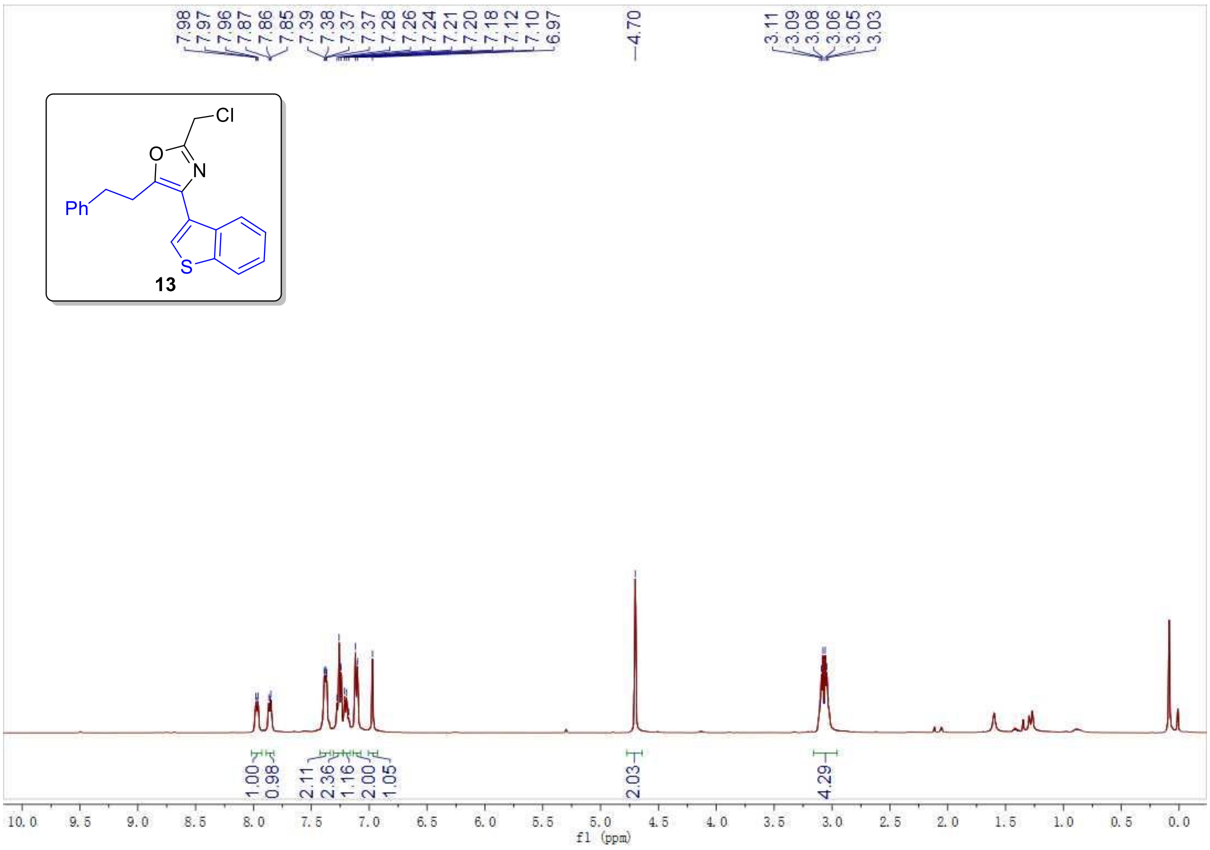

^{13}C NMR (101 MHz, $CDCl_3$) spectrum of 13

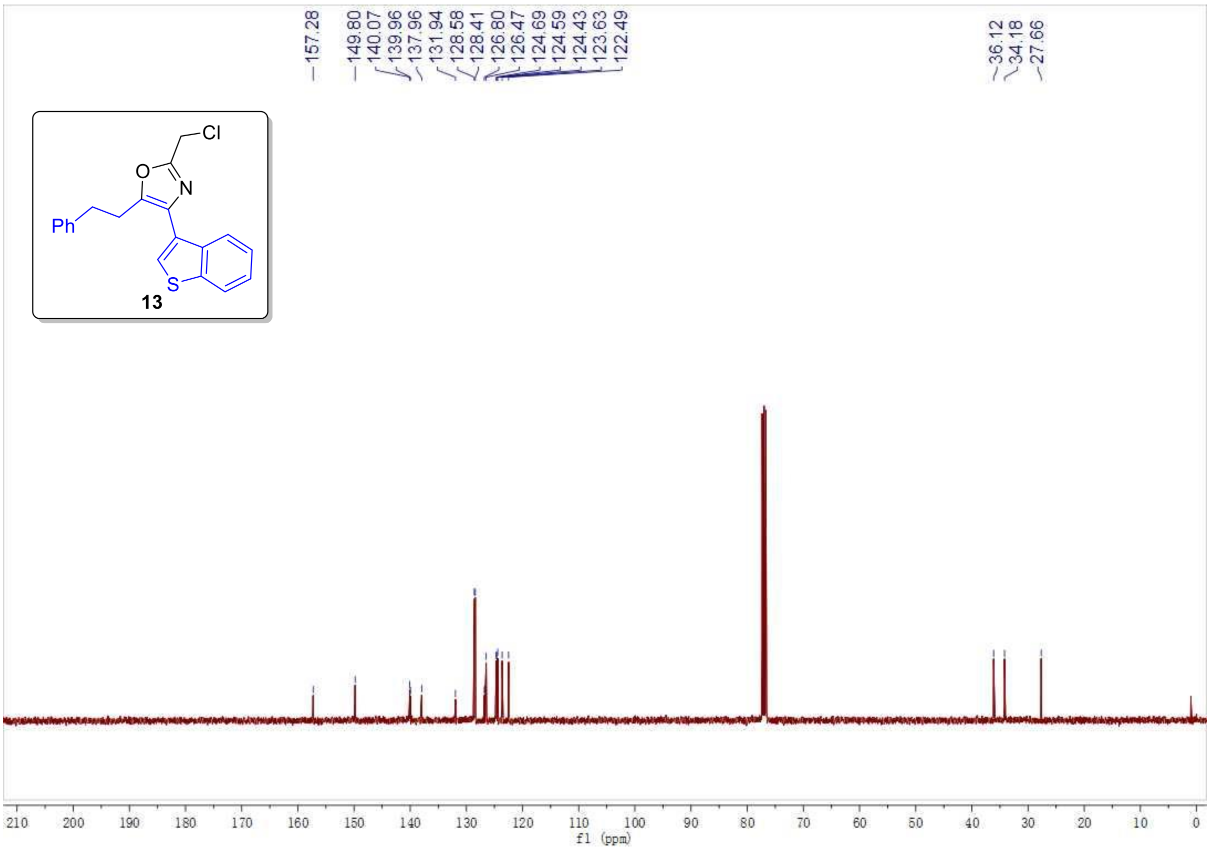

^{1}H NMR (400 MHz, $CDCl_3$) spectrum of 15

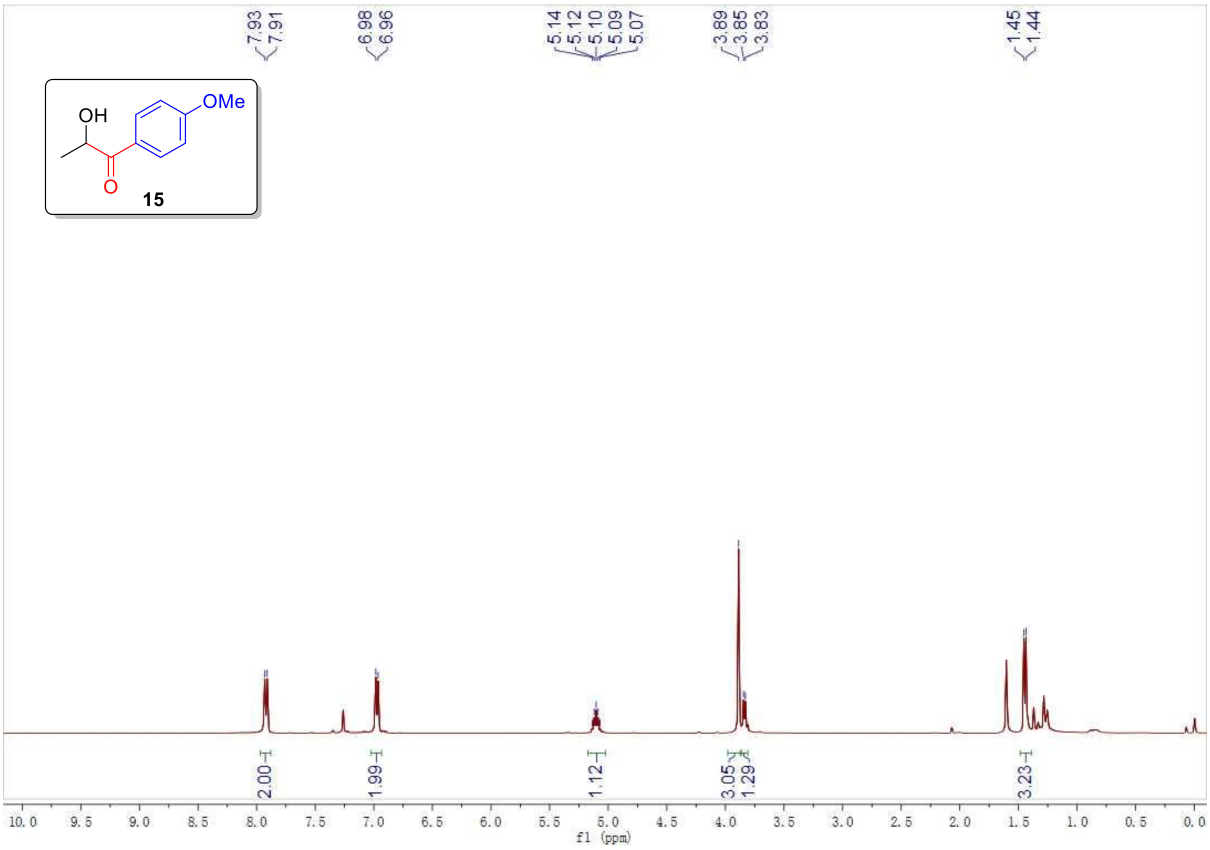

^{13}C NMR (101 MHz, $CDCl_3$) spectrum of 15

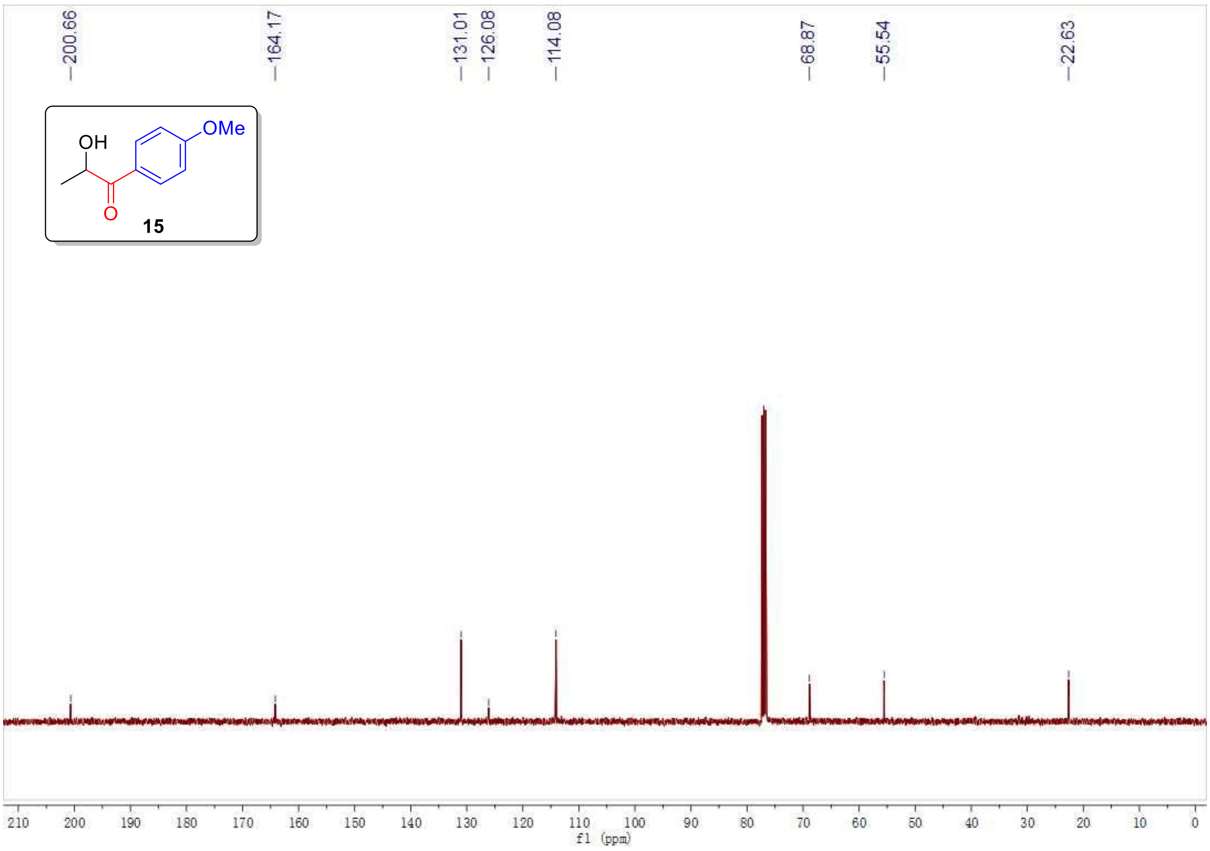

^{1}H NMR (400 MHz, CDCl$_3$) spectrum of 16

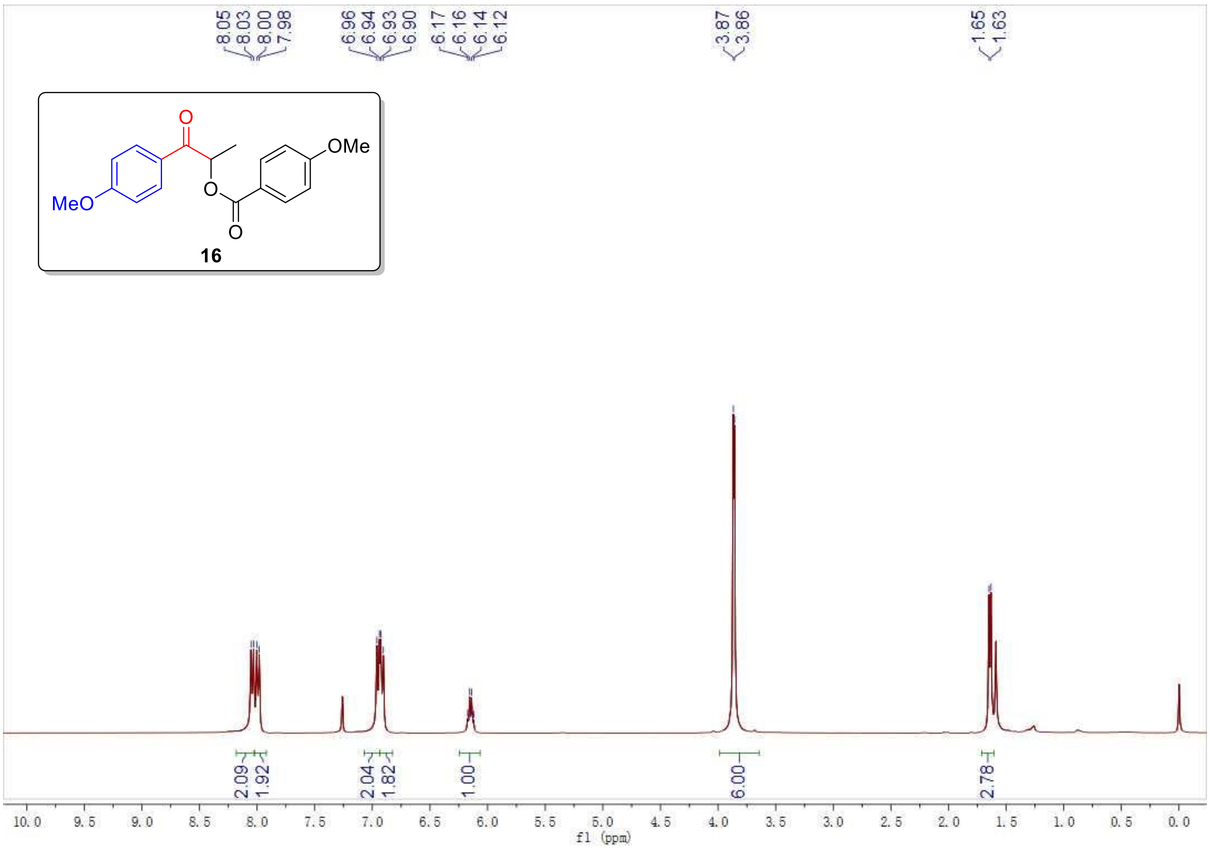

^{13}C NMR (101 MHz, CDCl$_3$) spectrum of 16

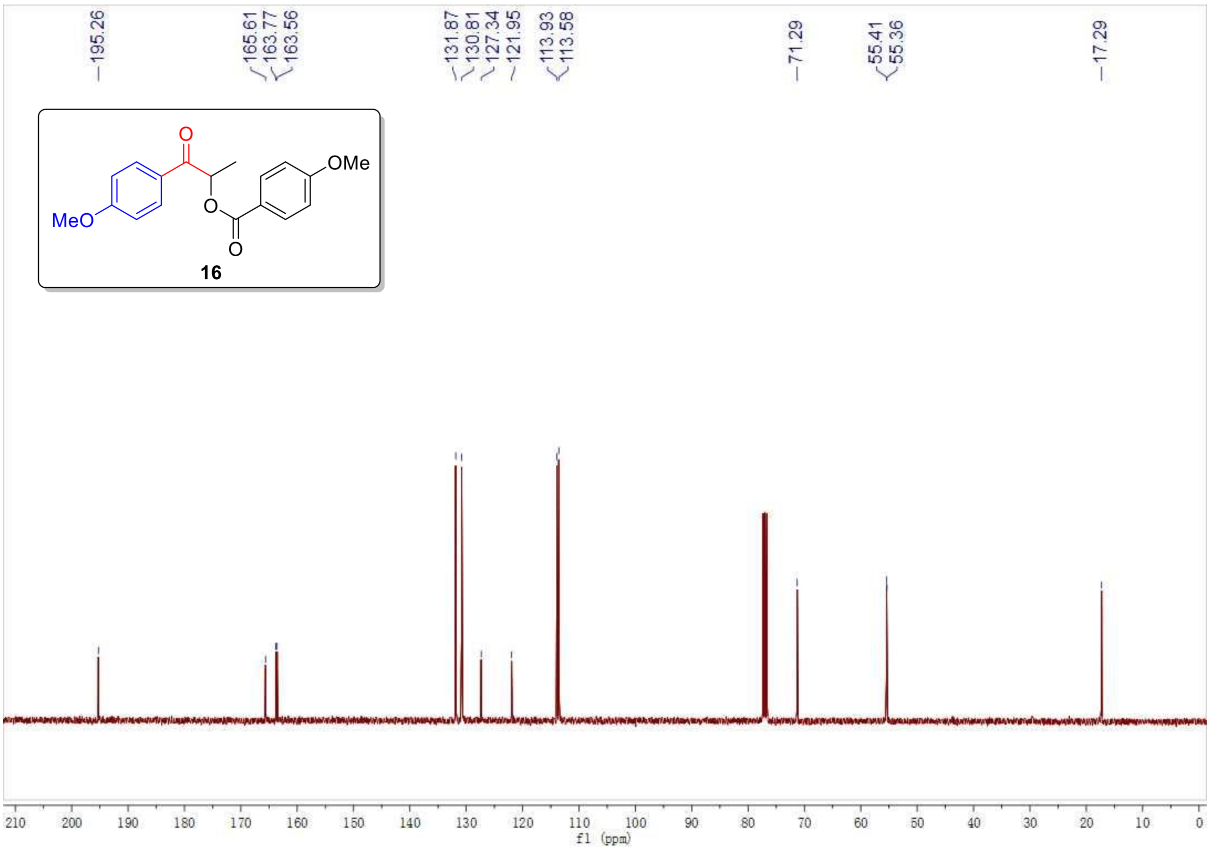

^{1}H NMR (400 MHz, $CDCl_3$) spectrum of 18

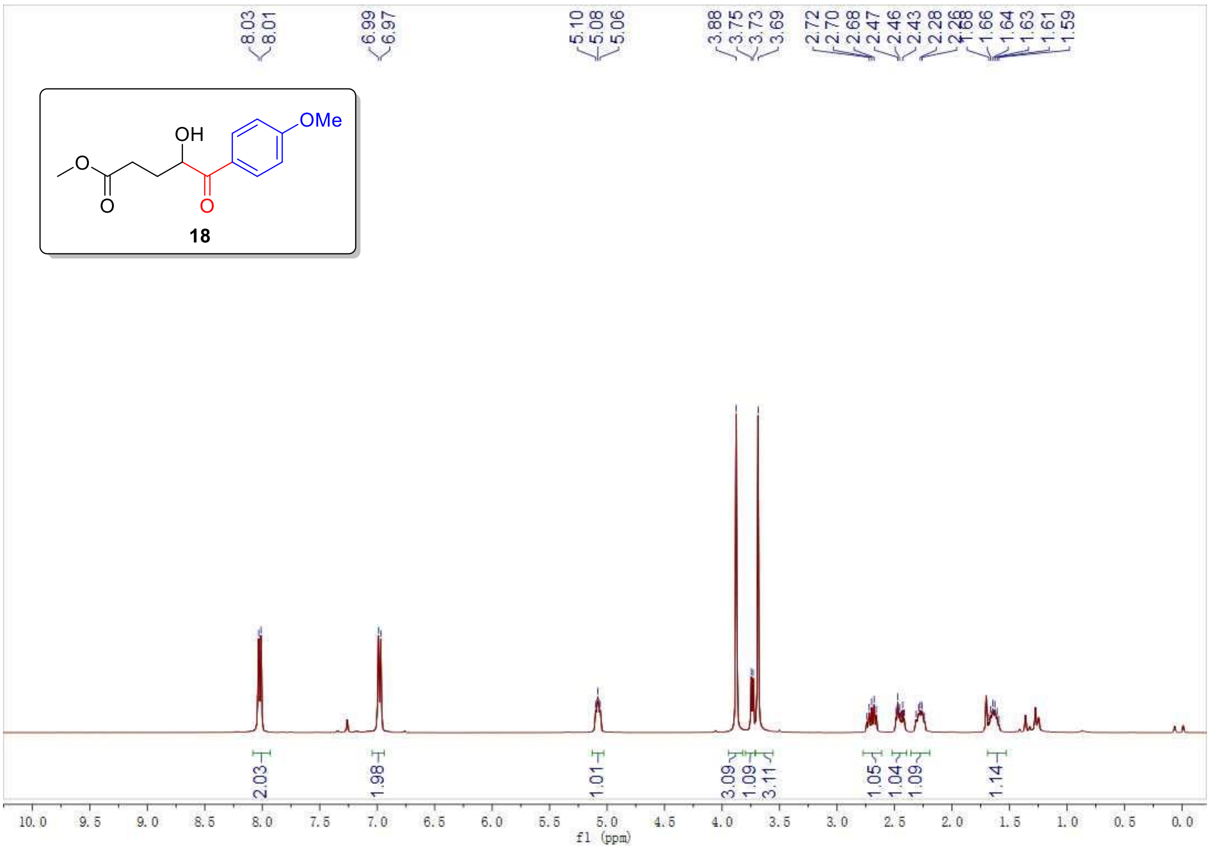

^{13}C NMR (101 MHz, $CDCl_3$) spectrum of 18

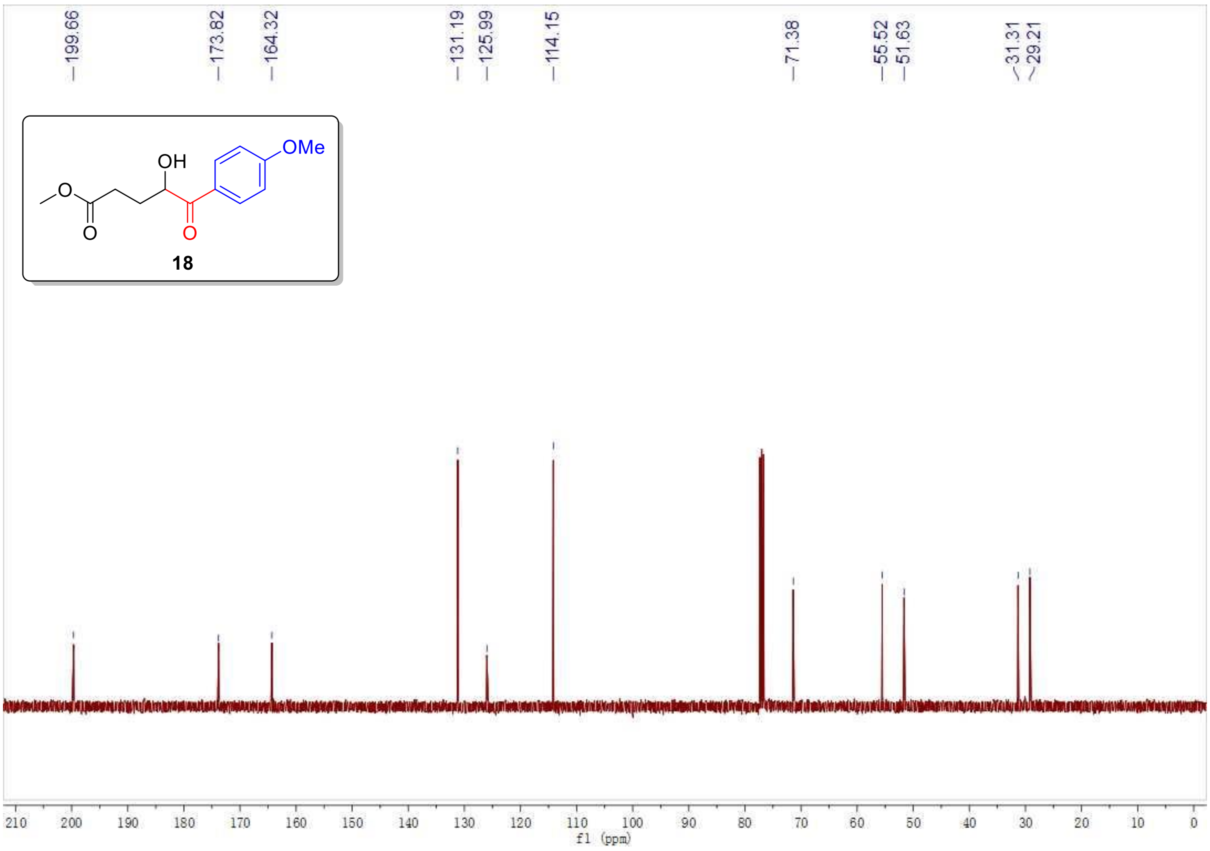

^{1}H NMR (400 MHz, $CDCl_3$) spectrum of 19

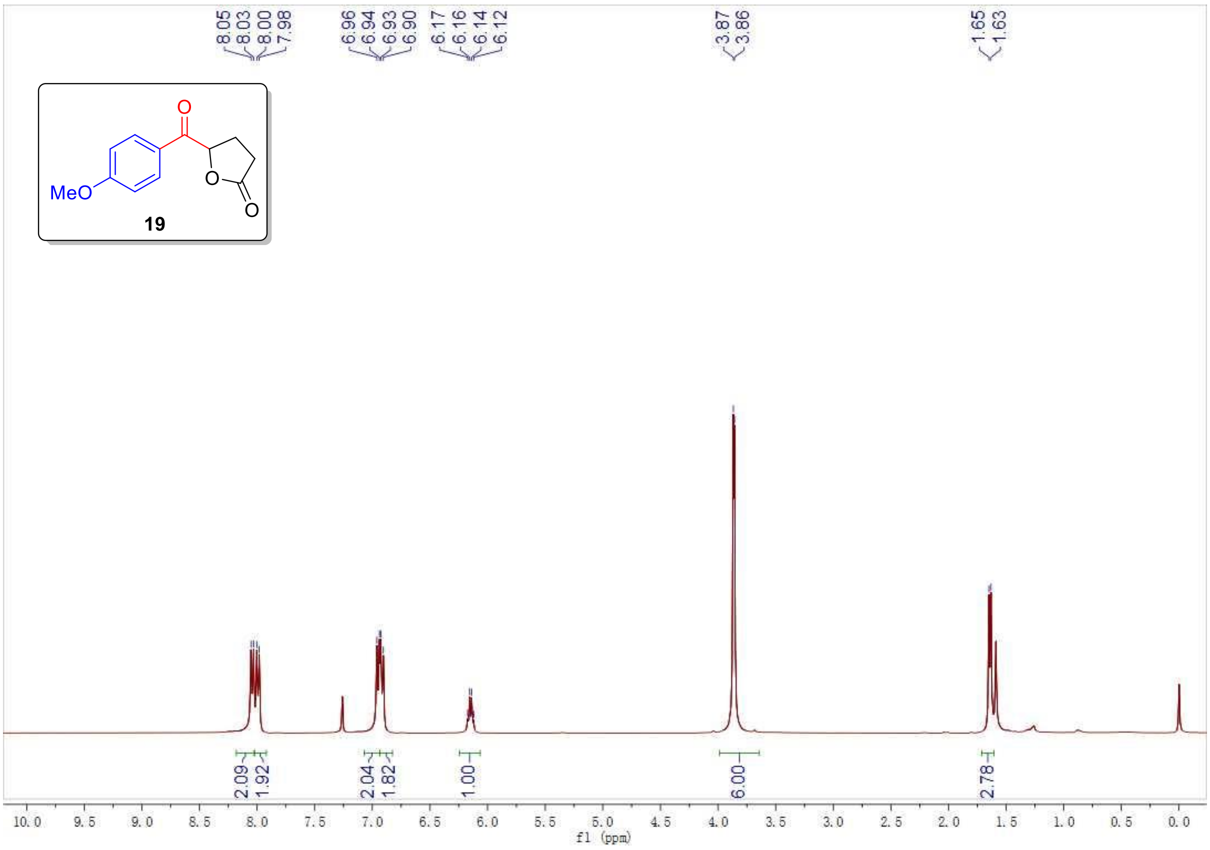

^{13}C NMR (101 MHz, $CDCl_3$) spectrum of 19

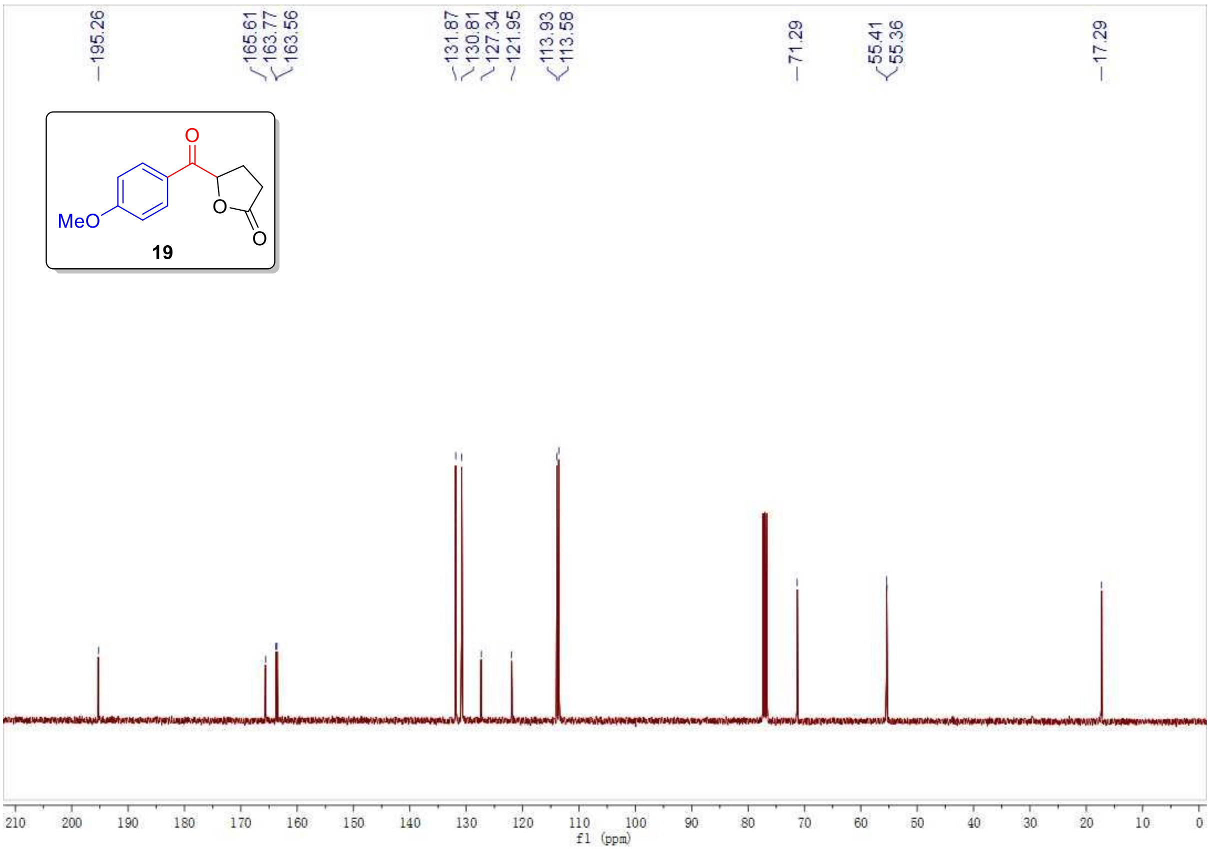

^{1}H NMR (400 MHz, $CDCl_3$) spectrum of 22

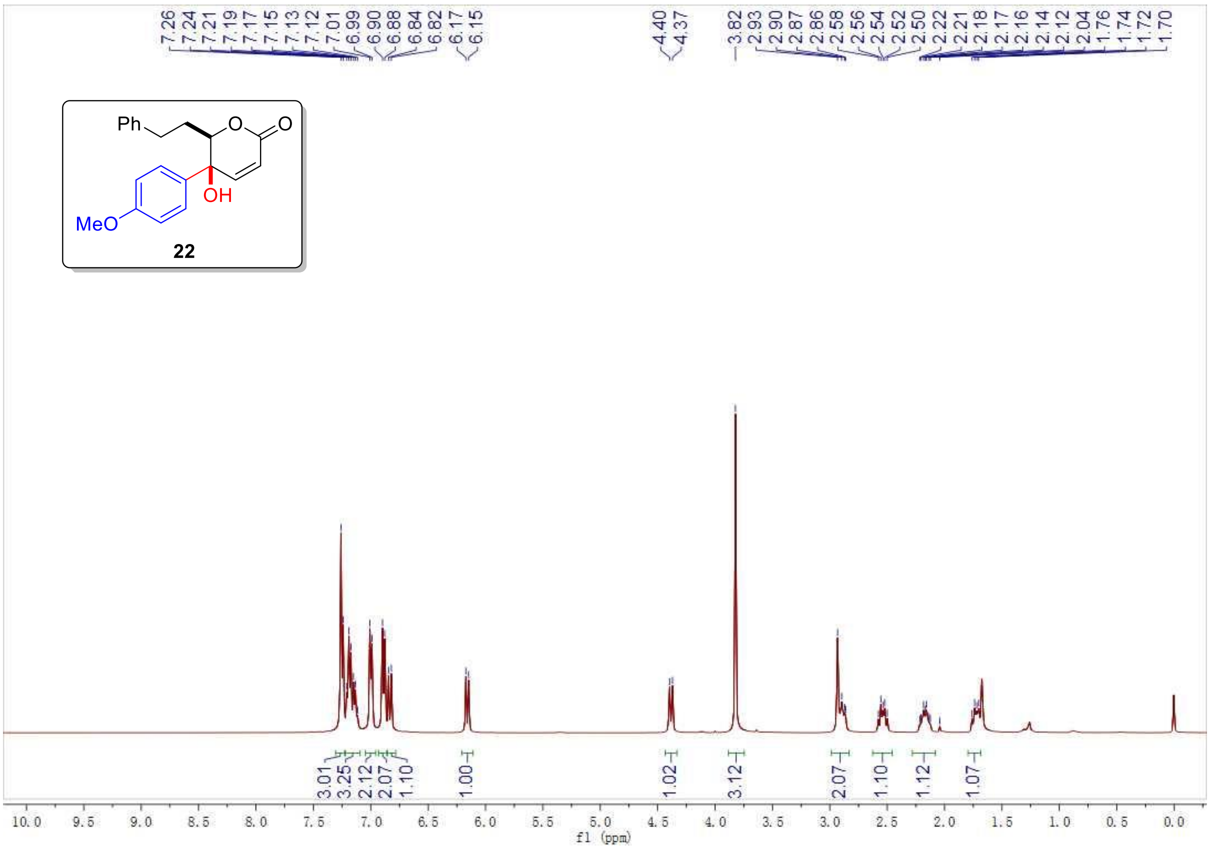

^{13}C NMR (101 MHz, $CDCl_3$) spectrum of 22

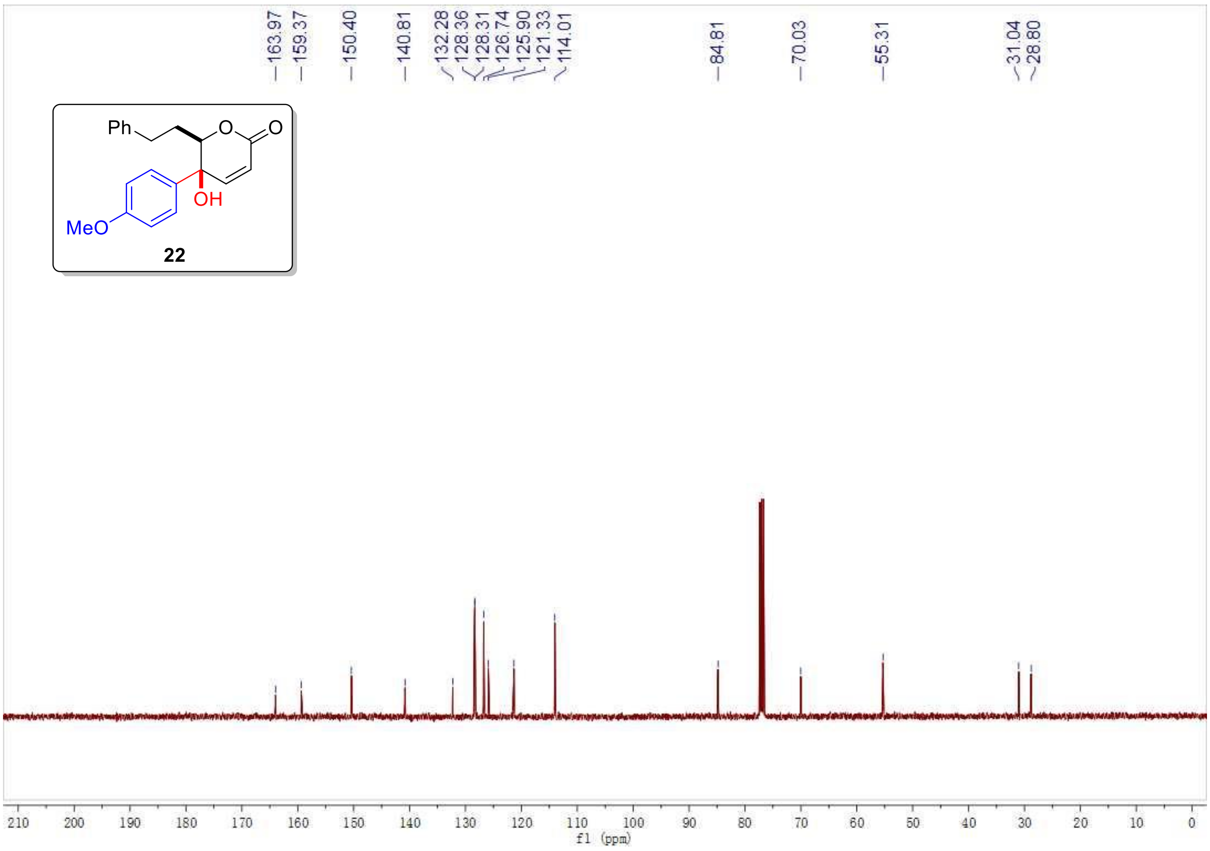

8 Supplementary references

1 Walsh, K., Sneddon, H. F. & Moody, C. J. Solar photochemical oxidations of benzylic and allylic alcohols using catalytic organo-oxidation with DDQ: application to lignin models. *Org. Lett.* **16**, 5224-5227 (2014).

2 Ghiringhelli, F., Nattmann, L., Bognar, S. & van Gemmeren, M. The direct conversion of α-hydroxyketones to alkynes. *J. Org. Chem.* **84**, 983-993 (2019).

3 Ooi, T., Uraguchi, D., Morikawa, J. & Maruoka, K. Unique synthetic utility of $BF_3 \cdot OEt_2$ in the highly diastereoselective reduction of hydroxy carbonyl and dicarbonyl Substrates. *Org. Lett.* **2**, 2015-2017 (2000).

4 Huang, Y.-W. & Frontier, A. J. Nazarov cyclization/internal redox cyclization sequence for the synthesis of N-heterocyclic bridged ring systems. *Org. Lett.* **18**, 4896-4899 (2016).

5 Li, G., Tang, L., Liu, H., Wang, Y., Zhao, G. & Tang, Z. Investigation and Application of amphoteric α-amino aldehyde: an in situ generated species based on heyns rearrangement. *Org. Lett.* **18**, 4526-4529 (2016).

6 Kim, H. R., Achary, R. & Lee, H.-K. DBU-promoted dynamic kinetic resolution in Rh-catalyzed asymmetric transfer hydrogenation of 5-akyl cyclic sulfamidate imines: stereoselective synthesis of functionalized 1,2-amino alcohols. *J. Org. Chem.* **83**, 11987-11999 (2018).

7 Patil, P. C., Luzzio, F. A. & Demuth, D. R. Oxazoles for click chemistry II: synthesis of extended heterocyclic scaffolds. *Tetrahedron Lett.* **56**, 3039-3041 (2015).

8 Liu, W., Chen, C. & Zhou, P. N,N-Dimethylformamide (DMF) as a Source of Oxygen To Access α-Hydroxy Arones via the α-Hydroxylation of Arones. *J. Org. Chem.* **82**, 2219-2222 (2017).

9 Jia, W.-G., Zhang, H., Li, D.-D. & Yan, L.-Q. One-pot synthesis of acyloxy carbonyl compounds from ketones using a Pybox–copper(II) catalyst. *RSC Adv.* **6**, 27590-27593 (2016).

10 Ding, Y. *et al.* DDQ-promoted dehydrogenation from natural rigid polycyclic acids or flexible alkyl acids to generate lactones by a radical ion mechanism. *Chem. Commun.* **47**, 9495-9497 (2011)..

11 Wildemann, H., Dünkelmann, P., Müller, M. & Schmidt, B. A short olefin metathesis-based route to enantiomerically pure arylated dihydropyrans and α,β-unsaturated δ-valero lactones. *J. Org. Chem.* **68**, 799-804 (2003).
